# Genome wide analysis of W-box element in Arabidopsis thaliana reveals TGAC motif with genes down regulated by heat and salinity

**DOI:** 10.1038/s41598-019-38757-7

**Published:** 2019-02-08

**Authors:** Pinky Dhatterwal, Samyadeep Basu, Sandhya Mehrotra, Rajesh Mehrotra

**Affiliations:** 1 Department of Biological Sciences, BITS Pilani, K. K Birla Goa Campus, Goa, India; 2 Department of Biological Sciences, Birla Institute of Technology and Science, Pilani, India

## Abstract

To design, synthetic promoters leading to stress-specific induction of a transgene, the study of *cis*-regulatory elements is of great importance. *Cis*-regulatory elements play a major role in regulating the gene expression spatially and temporally at the transcriptional level. The present work focuses on one of the important *cis*-regulatory element, W-box having TGAC as a core motif which serves as a binding site for the members of the WRKY transcription factor family. In the present study, we have analyzed the occurrence frequency of TGAC core motifs for varying spacer lengths (ranging from 0 to 30 base pairs) across the *Arabidopsis thaliana* genome in order to determine the biological and functional significance of these conserved sequences. Further, the available microarray data was used to determine the role of TGAC motif in abiotic stresses namely salinity, osmolarity and heat. It was observed that TGAC motifs with spacer sequences like **TGAC**CCATTT**TGAC** and **TGAC**CCATGAATTT**TGAC** had a significant deviation in frequency and were thought to be favored for transcriptional bindings. The microarray data analysis revealed the involvement of TGAC motif mainly with genes down-regulated under abiotic stress conditions. These results were further confirmed by the transient expression studies with promoter-reporter cassettes carrying TGAC and TGAC-ACGT variant motifs with spacer lengths of 5 and 10.

## Introduction

Plants being sessile organisms, encounter various biotic and abiotic stresses which greatly constraints their growth and productivity. Drought, salinity and high temperatures being the most important abiotic stresses, leading to an average yield loss of 50% in crop production worldwide^1,2^. Transgenic technology is being widely used to develop plants that can sustain under unfavorable environmental conditions with improved crop yield. The expression levels of a transgene depend on their promoter regions whose strength and specificity rely on their *cis*-regulatory element architecture^3,4^.

Generally, constitutive promoters are used to functionally characterize transgenes as they direct their expression in all tissues and throughout all developmental stages^3,5^. The problem with constitutive promoters, they drive constant gene expression irrespective of the necessity resulting in excessive energy and nutrient losses^6^. A plausible solution can be the development of stress-inducible promoters possessing an array of a specific type, copy number, order, position and combination of *cis*-acting regulatory elements positioned upstream of the core promoter sequence^7–12^. In order to develop stress-inducible promoters, sequence identification and functional characterization of different *cis*-acting regulatory motifs under different stress conditions is required^13^. The present paper is focused on one of the important *cis*-regulatory element; the W-box, as being widely reported to be responsible for inducing plant genes in response to pathogen attack^14–16^.

W-box has (C/TTGACT/C) as a core sequence and acts as a binding site for WRKY TFs^17^. Reports state that the tetramer sequence TGAC of W-box element is highly conserved. However, researchers like Ciolkowski *et al*.^18^ have shown that although TGAC core is essential for binding of WRKY family of transcription factors, adjacent sequences also play a critical role in determining the binding site preferences^18^. This is one of the major areas we are going to focus on in this paper, thus formulating some approaches to determine the specific spacer distance and spacer sequence required for selective binding of WRKY family of TF’s and how the gene expression is regulated under abiotic stress conditions. Along with TGAC as the core element, ACGT motif was also included in the analysis as known to be an important functional *cis*-regulatory element which generally acts synergistically with other motifs to regulate gene expression^19^. Computational and statistical approaches were used to analyze how different spacer sequences involving W-box elements and its variants might play a role in gene regulation. The publicly available microarray data for different stress conditions like salinity, osmotic, and heat were analyzed to study the regulatory roles of TGAC motif. Furthermore, transient expression studies were done to confirm the in-silico findings. The data generated in this work will be useful for designing abiotic stress-responsive promoters^20^.

## Results

### Spacer Frequency Comparison

**Figure 1:**
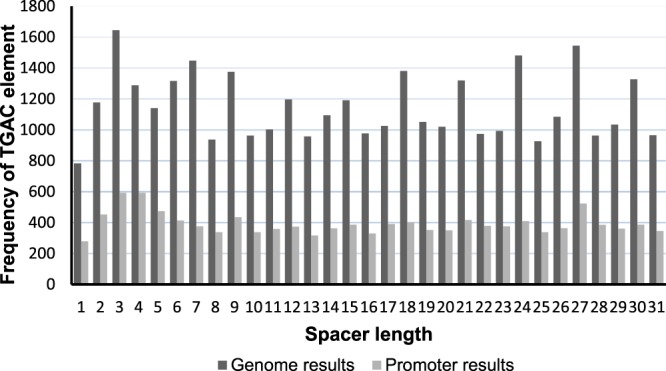
The Frequency of TGAC element vs. spacer length across *Arabidopsis thaliana* genome and promoter regions.

**Figure 2:**
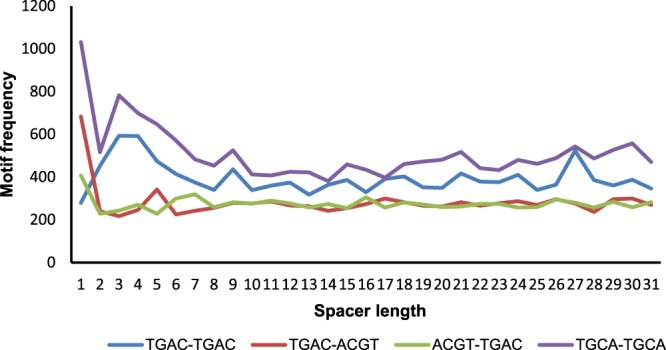
The Frequency of different motif combinations (TGAC-TGAC, TGAC-ACGT, ACGT-TGAC, TCA-TGCA) vs. spacer length.

The occurrence frequency of TGAC(N)TGAC motif (for N = 0 to N = 30) was searched (genome-wide & in promoter regions), the corresponding results were analyzed (Fig. 1). Since ACGT motif is known to be extremely important for transcription factor binding when present with other motifs like TGAC, hence the occurrence frequencies for TGAC(N)ACGT and ACGT(N)TGAC were also searched in the promoter regions. The occurrence frequencies for a variant of W-box element TGCA(N)TGCA were also looked for along with the above-mentioned motifs (Fig. 2). On comparing, it was seen that for almost all spacer lengths except 0, TGAC(N)TGAC overall had a higher frequency of occurrence in comparison to TGAC variants with ACGT motif (TGAC_N_ACGT and ACGT_N_TGAC). This indicates that the TGAC_N_TGAC as a core has more important regulatory role than TGAC_N_ACGT or any other variants. However, it was seen that of all the above motifs, TGCA(N)TGCA was found to have a higher frequency than other motifs throughout the 30 spacer lengths.

### Spacer Sequence Analysis

It was seen that for spacer length 6 in the genomic region, which had a total frequency of 1448, the spacer sequence ‘CCATTT’ comprised 582 out of the total. The probability of occurrence of CCATTT would have been (1/4^6) statistically, which signifies the presence of a huge deviation. Similar trends were observed for spacer sequences of length 10 (*TGAC****CCATGAATTT****TGAC*) in the promoter regions. The conclusion that can be drawn out of this data is that might be these sequences play some distinct regulatory role in governing the gene expression under stress conditions. This binding pattern even alludes in-phase binding of transcription factors to TGAC(N)TGAC motif.

### Microarray Data Analysis

WRKY TFs play a key role in regulating stress responses under both biotic and abiotic stresses and are also involved in various physiological and growth-related processes^21,22^. Until recently, research has been majorly focused on their biotic stress responses^23,24^. Here, we report the involvement of W-box in abiotic stress as revealed by microarray analysis. The frequency of motifs TGAC(N)TGAC, TGAC(N)ACGT and ACGT(N)TGAC in genes which are up-regulated or down-regulated during stresses namely salinity, heat and osmotic were analyzed (see Supplementary datasheet). The normalized frequency values for each motif were calculated as follows:

$$Normalized\,frequency=(total\,frequency\,of\,a\,motif)/(Gene\,count\,for\,the\,particular\,stress\,condition)$$

**Figure 3:**
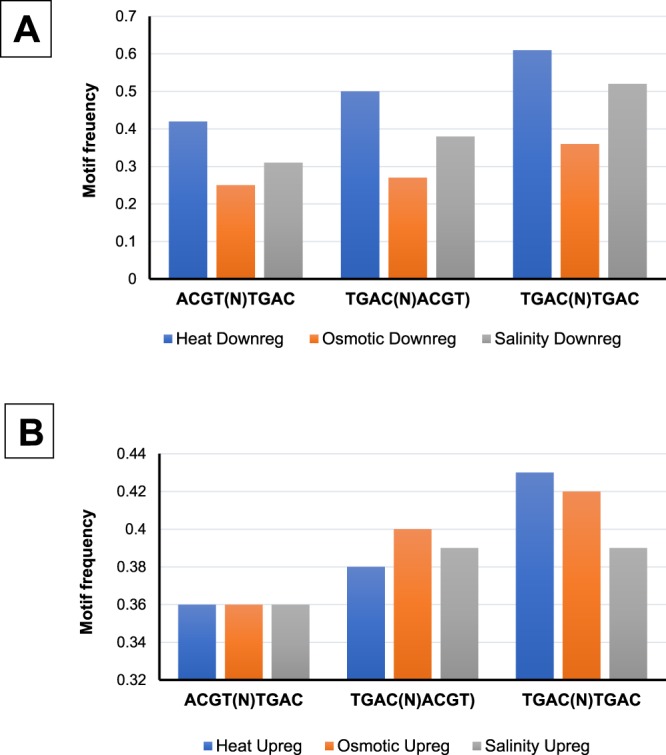
The Occurrence frequency of different TGAC motifs in genes (**A**) down-regulated and (**B**) up-regulated under heat, osmotic and salinity stresses.

Further, on comparing these normalized frequencies a threshold value was set as 0.45. Result unveils that frequency value for TGAC(N)TGAC motif in genes down-regulated during heat and salinity stress is 0.61 and 0.52 respectively (Fig. 3). This points out that TGAC(N)TGAC might play a major role in the promoter region of genes down-regulated during heat and salinity stress, as their observed frequency values are above the threshold by a significant margin. It can also be observed that the normalized frequency values for TGAC(N)TGAC is higher than TGAC(N)ACGT and ACGT(N)TGAC for both categories of genes which are up-regulated and down-regulated during heat stress. In case of salinity stress, TGAC(N)TGAC motif displayed a higher normalized value in genes which are down-regulated, this indicates that it might have some regulatory role in binding of transcription factors which cause down-regulation of genes during saline conditions. These observations suggest and justify the theoretical belief that W-Box elements are involved in the regulation of genes taking part in both biotic and abiotic stress conditions. From the Fig. 3, it can be deciphered that the normalized frequency of none of the motifs displayed any significant deviation in genes which are getting up-regulated during the heat, salinity and osmotic stresses. Also, none of the motifs has any role in the promoter region for up-regulation/down-regulation of genes involved in osmotic stress.

### Gene search for motif occurrence

The occurrence patterns of TGCA(N)TGCA and TGAC(N)TGAC motifs were searched across 25000 genes of *Arabidopsis thaliana*. The genes for which the peaks were observed were further analyzed. In a case of TGCA(N)TGCA motif, it was seen that the genes with peaks do not reveal high frequency for any particular spacer length, thus suggesting the peak could be due to certain random occurrences which do not play important regulatory roles. However, TGAC(N) TGAC analysis presented some interesting results. A peak was observed for gene AT1G56420 with a frequency of 19, out of which frequency for spacer 21 was 6 whereas frequency for spacer 22 was found to be 10. From this observation, it can be put forward that in the promoter of gene AT1G56420, two TGAC motifs with a spacing of around 20 bp is highly optimal. On looking at this gene more closely it was found that a long sequence of 50 bp is repeated multiple numbers of times in the promoter region. Similarly, the promoter of another gene AT2G20670 was found to have a repeating sequence of around 315 bp in length which contains flanking TGAC motifs. This sequence is repeated throughout the promoter of the gene. It has been seen that generally transcription factors tend to bind in groups and act synergistically to enhance the effect on one another. The occurrence pattern of W-box sequence in these promoters suggests that the WRKY TFs may also act cooperatively^25^. While analyzing the occurrence patterns of TGAC(N)TGAC motifs, it was noticed that consecutive TGAC’s are not preferred in the promoter region, rather a spacing of 3 or 4 was found to be optimal. However, this is in contrast with the result for TGAC(N)ACGT motif where a spacer length of zero is highly preferred. TGACACGT motif is highly preferred which indicates that it might have some important biological role.

### Similarity scoring between sequences

A similarity scoring mechanism as described in *Methods* was used to find similarity between different spacer sequences of genes containing multiple TGAC elements. An interesting result was obtained for genes which were down-regulated during osmotic stress conditions. It was observed that genes AT4G07450 and AT4G04830 were found to have sequences containing three TGAC’s along with a similarity score of 85.9% for their spacer sequences. Further for the genes AT2G27150, AT5G26340 and AT3G26830, which were up-regulated during osmotic stress, it was observed that the spacer sequences had a similarity score of 88.8% between AT2G27150 and AT3G26830 and 72.9% similarity between AT3G26830 and AT5G26340. All these sequences were observed to be present 50–120 bp upstream of their respective genes which therefore showed a structural similarity amongst the above-mentioned genes. Further analysis of the functionality of these genes was done and the results showed a striking similarity between them. AT3G26830 encodes a cytochrome p450 enzyme that catalyzes dihydro camalexin acid to camalexin. Camalexin is found to be cytotoxic which has a role in cell death. AT2G27150 encodes aldehyde oxidase delta isoform catalyzing final step in the abscisic acid synthesis. AT5G26340’s expression in mutants involved in Programmed Cell Death shows a high correlation between gene expression and Programmed Cell Death. It can be seen that all three genes with similar spacer sequences play analogous roles in Programmed Cell Death.

### Transient expression analysis of TGAC reporter cassette

The expression of the *gusA* gene in leaves bombarded with promoter-reporter cassette carrying a single TGAC motif or in tandem and separated by a spacer of 5 and10 nucleotides under abiotic stress (Salt, ABA) conditions was analyzed. The expression of the reporter gene driven by TGAC with ACGT motif separated by 5 and 10 nucleotides was also studied. The leaves bombarded with a 50 + Pmec reporter cassette without any TGAC motif were treated as a control. The data (as shown in Table 1) clearly shows a gradual reduction in the reporter gene expression, expressed under the effect of a minimal promoter (50 + Pmec) carrying TGAC activator motif as a single copy or two in tandem separated by a spacer length of 0, 5, 10. Under salt stress, the constructs carrying motifs (TGAC) (TGAC), (TGAC)_N5_ (TGAC), (TGAC)_N10_ (TGAC) reduced the reporter gene expression by 1.57, 4.30, 7.74 folds respectively as compared to the control construct. A similar pattern was observed for the ABA treatment, the gene expression got reduced to 1.46, 3.60, 5.15 folds with increased spacing between the two TGAC motifs. ACGT motif is widely known to drive the gene expression highly under abiotic stress conditions. However, ACGT motif when placed with TGAC motif separated by a spacer length of 5 and 10, did not significantly increased the gene expression under stress conditions.

**Table 1:** Transient expression data.

Promoter cassette	Uninduced (pmoles/min/mg protein ± s.d.)	Fold activity as compared to 50 + Pmec	NaCl (pmoles/min/mg protein ± s.d.)	Fold induction	p value	ABA (pmoles/min/mg protein ± s.d.)	Fold induction	p value
50 + Pmec	1807 ± 57.3	1	1827 ± 66.2	1.01	p = 0.7126	1872 ± 89.6	1.03	p = 0.3495
TGAC + 50 + Pmec	2209 ± 80.7	1.22	2330 ± 102.3	1.05	p = 0.1830	2347 ± 76.3	1.06	p = 0.0977
(TGAC) (TGAC) + 50 + Pmec	2670 ± 180.2	1.47	1760 ± 80.2	0.65	p = 0.0013	1952 ± 92	0.73	p = 0.0036
(TGAC) _N5_ (TGAC) + 50 + Pmec	3608 ± 160.7	1.99	812.2 ± 78	0.22	**p** < **0.0001**	1008.7 ± 92	0.27	**p** < **0.0001**
(TGAC) _N10_ (TGAC) + 50 + Pmec	4708 ± 287	2.60	608 ± 49.2	0.12	**p** < **0.0001**	940 ± 82	0.19	**p** < **0.0001**
(ACGT) _N5_ (TGAC) + 50 + Pmec	3872 ± 169.2	2.14	4782 ± 190.6	1.23	p = 0.0035	5008 ± 207.6	1.29	p = 0.0018
(ACGT) _N10_ (TGAC) + 50 + Pmec	4967 ± 228.6	2.74	5568 ± 230.2	1.12	p = 0.0326	6200 ± 290.8	1.24	p = 0.0045
(TGAC) _N5_ (ACGT) + 50 + Pmec	4387 ± 263	2.42	5300 ± 428	1.20	p = 0.0346	6120 ± 283	1.39	p = 0.0015
(TGAC) _N10_ (ACGT) + 50 + Pmec	4962 ± 310	2.74	6023 ± 217.8	1.21	p = 0.0083	6347 ± 312.6	1.27	p = 0.0055

## Discussion

Transgenic technology is being widely used to develop plants that can sustain under unfavorable environmental conditions without much loss in crop yield. For significant expression of a transgene, an efficient promoter is a necessity. Now-a-days, synthetic promoters are being preferred over the constitutive promoters as they offer for more defined and efficient spatial and temporal control of transgene expression^8^. The activity of a synthetic promoter relies on the type of *cis*-regulatory motifs included as well as on their positions, copy number, inter-motif distance and orientations^7,10,26–28^. So, in order to design synthetic promoters leading stress-specific induction of a transgene, the identification and functional characterization of different *cis*-acting regulatory motifs is of great importance. One such regulatory motif is W-box (TTGACC/T) to which WRKY TFs bind to regulate temporally and spatially gene’s expression under different stress conditions. Although, the TGAC core is essential for binding of WRKY TFs; the flanking sequences also play a key role in determining the binding site preferences as reported by Ciolkowski *et al*.^18^. Focusing on this point we used computational approaches to determine the specific spacer distance and spacer sequence of TGAC(N)TGAC [N = 0–30] required for selective binding of WRKY family of TF’s. It was seen that for almost all spacer lengths except 0 and 1, TGAC(N)TGAC had a higher frequency of occurrence than TGAC-ACGT variants. Indicating that TGAC_N_TGAC motif is preferred binding site for WRKY TFs. Further, we looked for the spacer sequences of TGAC(N)TGAC motif which showed more conservation at particular spacer lengths. As this spacer sequence analysis of w-box motif will be beneficial for designing synthetic plant promoters with defined regulatory elements to modulate gene expression under specific stress conditions^9^. Our methods churned out that certain spacer sequences lying between two TGAC motifs with spacer sequences **TGAC**CCATTT**TGAC** and **TGAC**CCATGAATTT**TGAC** had a significant deviation in frequency and were thought to be favored for transcriptional bindings. This binding pattern even suggests in-phase binding of transcription factors to TGAC(N)TGAC motif. WRKY TFs play a key role in regulating stress responses under both biotic and abiotic stresses and are also involved in various physiological and growth-related processes^22,23^. Until recently, research has been majorly focused on their biotic stress responses^24,25^. Here, we report the involvement of W-box in abiotic stress responses. On analyzing the microarray data, it hinted at the possibility of the role of TGAC(N)TGAC motif in the regulation of genes which are down-regulated during heat stress and salinity stress. To further confirm these in-silico findings, transient expression studies with 50 + Pmec reporter cassettes carrying TGAC motifs were performed. The reporter gene expression was analyzed under abiotic stress conditions by bombarding the tobacco leaves with the promoter-reporter cassettes. In correlation with the in-silico studies, the expression studies also presented similar results. The *gusA* gene expression was reduced gradually as the spacing between the two TGAC motifs was increased. TGAC motifs separated with a spacer length of 10 decreased the gene expression to around 7.74 and 5.15 folds under both the salt and ABA treatments respectively. The effect of promoter constructs carrying TGAC-ACGT variants with spacer distance of 5 and 10 nucleotides on the reporter gene expression was also analyzed. ACGT is known to induce gene expression under abiotic stress, however, only one fold increase was observed when coupled with TGAC motif.

The transient data generated using biolistics system strengthened our in-silico findings that the TGAC motif down-regulates the gene expression under abiotic stress conditions. The data also suggest that the TGAC motif might be acting as a negative regulator or repressor leading to reduced reporter gene expression in response to abiotic stress conditions. As the expression of a gene, majorly depends on the *cis*-regulatory elements arranged in their promoter regions. So, to control and modulate a transgene expression under abiotic stresses the role of TGAC motif as the negative regulator can be taken into consideration. Hence, for designing abiotic stress-inducible promoters these finding can be useful. This analysis is indicative of the results obtained. However, to make the analysis more robust stable transgenics need to be developed.

## Methods

### Data Extraction

For analysis, the genomic and promoter region sequences of *Arabidopsis thaliana* were retrieved from NCBI Reference Sequence Database and Arabidopsis Gene Regulatory Information Server (AGRIS) database respectively^29,30^. Further, the co-occurring frequency of TGAC elements was determined across the genome and in the promoter regions with spacer length ranging from 0 to 30. No computation over 30 spacer lengths was done, as transcription factors generally do not require more than 25 bp to bind (see Supplementary datasheet). The frequencies for spacers involving a) TGAC and ACGT motifs b) co-occurring TGCA elements for the promoter region were also computed (see Supplementary datasheet). The sequences of each spacer region (between two TGAC elements/between TGAC and ACGT elements/between two TGCA) were also extracted and the total numbers of occurrences for each spacer length were determined. In order to test the significance of these frequencies, we used four palindromic – AGCT, TGCA, CTAG, GATC and four non-palindromic – TAGC, CGTA, GCTA, ATGC, sequences as controls^19^.

### Spacer Sequence Analysis

Spacer sequences obtained for the TGAC(N)TGAC motif (N = 0 to N = 30) on analyzing the promoter regions were further examined to find a pattern which exists in their occurrence and to correlate them with a certain regulatory role if any. The searching technique used a modified version of the Knuth-Morris-Pratt algorithm which was able to search for all pattern occurrences of length n within a string of length k, O (n + k)^31,32^. Usage of this algorithm highly reduces the searching time over any naive method. Additionally, nucleotide preference for each position within a spacer sequence was calculated. The threshold occurrence percentage for C/G was taken as 25% and for A/T for a particular position was 40%.

### Functional Analysis

In this phase, the microarray data from EBI Gene Expression Atlas for *Arabidopsis thaliana* was used^33^. Using this data, we calculated whether genes containing multiple core TGAC *cis*-elements were up-regulated/down-regulated during various stress conditions like salinity, osmotic, and heat. We also compared the genes regulated under the given stress conditions with those genes containing multiple TGAC elements to find the likelihood of occurrence. The following statistical formula was used:

$$Likelihood\,of\,occurrence:\,(A\cap B)/(B\ast P(A))$$

A: Event that a given gene is up-regulated/down-regulated by a particular stress conditionB: Event that a given gene contains multiple TGAC elements separated by N base pairsLikelihood of occurrences for N = 0 to N = 30 was calculated using this method and analyzed.

### Sequence Similarity by Dynamic Programming

Dynamic programming was used to find out the similarity between the spacer sequences (up to 30 bps) of the genes containing multiple TGAC elements. The similarity was measured using a scoring mechanism as described:

$$For\,every\,nucleotide\,match:\,+\,2\,score\,was\,awarded$$

$$For\,every\,nucleotide\,mismatch:\,-\,2\,score\,was\,awarded$$

$$For\,every\,insertion/deletion:\,-\,1\,score\,was\,awarded$$

All those pairs of sequences which had a similarity score greater than 0.7 were further analyzed.

### Preparation of reporter cassette

**Figure 4:**
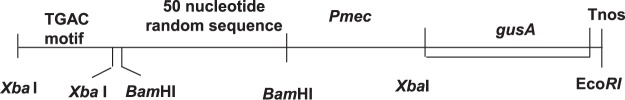
A layout of the promoter-reporter cassette to determine the effect of promoter architecture on *gusA* expression.

A minimal promoter *Pmec* sequence containing a TATA-box, a transcription start site, and the reporter gene *gusA* cloned in the plasmid pUC19 was used. A random sequence of 50 nucleotides (GGATCCGGCTATGGCGGAGCAAGATTCACTCTGC GAGGCCAAAGCTTACCCCGGAAGGATCC), was cloned at the *BamH1* site of the *Pmec*. Further, this promoter-reporter cassette was cloned in the pBluescript SK (+/−) Phagemid (Stratagene, USA). Upstream of the 50 random nucleotide sequence, different combinations of the TGAC motifs (TGAC (N) TGAC; N = 0, 5, 10), TGAC (N = 5,10) ACGT and ACGT (N = 5,10) TGAC were inserted at the XbaI site (Table 2, Fig. 4). The TGAC–Pmec-*gusA* cassettes were coated on gold microparticles and bombarded onto tobacco leaves at 1100 psi, using a biolistic gun (Bio-Rad PDS-1000/He).

**Table 2:** The sequences of TGAC motif separated with spacer sequences of varying lengths.

Motif sequences	Representation
TCTAGA**TGAC**TCTAGA	(TGAC)
TCTAGA**TGACTGAC**TCTAGA	(TGAC)_2_
TCTAGA**TGAC**ggcta**TGAC**TCTAGA	(TGAC)_N5_ (TGAC)
TCTAGA**TGAC**ggctatggcg**TGAC**TCTAGA	(TGAC)_N10_ (TGAC)
TCTAGA**ACGT**ggcta**TGAC**TCTAGA	(ACGT)_N5_ (TGAC)
TCTAGA**ACGT**ggctatggcg**TGAC**TCTAGA	(ACGT)_N10_ (TGAC)
TCTAGA**TGAC**ggcta**ACGT**TCTAGA	(TGAC)_N5_ (ACGT)
TCTAGA**TGAC**ggctatggcg**ACGT**TCTAGA	(TGAC)_N10_ (ACGT)

### Transient expression studies under abiotic stress conditions

To study the expression of the reporter *gusA* gene using different minimal promoter cassettes under abiotic stresses. The bombarded leaves were kept in the pertidishes with Hoagland solution supplemented with 400 mM NaCl for salt stress. After treatment, the plates were placed in the growth chamber maintained at temperature 25 °C, 16 h light/8 h dark period for 48 h. For abscisic acid treatment, bombarded leaves were placed in the Hoagland solution supplemented with 100 µM ABA. The transient expression studies using biolistic system were performed as described by Mehrotra *et al*. 2005. In brief, treated leaves were incubated at 25 °C and 16 h light/8 h dark photoperiod for 48 hrs. Subsequently, the leaves were immediately frozen, grounded in liquid nitrogen, and treated with GUS extraction buffer (50 mM Na2HPO4 pH 7.0, 1 mM EDTA, 0.1% v/v Triton X-100, 1.0 mM DTT and 0.1% SLS). The glucuronidase activity was assayed in cell-free extracts using 4-methyl umbelliferyl glucuronide^34^. Relative fluorescence of 4-methylumbelliferone (MU) was determined using Perkin Elmer Spectrofluorometer with excitation at 365 nm and emission at 455 nm. The expression data were analyzed statistically using t-test.

## Supplementary information

Datasheet 1

Datasheet 2

## Data Availability

The datasets generated during and/or analyzed during the current study are available in the supplementary information file.
