# Supplementary material for: Genome wide analysis of W-box element in Arabidopsis thaliana reveals TGAC motif with genes down regulated by heat and salinity
Source: Sci Rep. 2019 Feb 8;9:1681. doi: 10.1038/s41598-019-38757-7 (PMC6368537; doi:10.1038/s41598-019-38757-7)
Supplement: Supplementary file 2 — Datasheet 2 [file 41598_2019_38757_MOESM2_ESM.docx]

Genome wide analysis of W-box element in Arabidopsis thaliana reveals TGAC motif with genes down regulated by heat and salinity

Pinky Dhatterwal, Samyadeep Basu, Sandhya Mehrotra and Rajesh Mehrotra

Spacer sequence analysis for TGAC(N)TGAC motif for spacer length 0 to 30.

0 : 279

tgactgac 279

1 : 452

tgacatgac 158

tgacctgac 67

tgacgtgac 58

tgacttgac 169

2 : 593

tgacaatgac 74

tgacactgac 32

tgacagtgac 38

tgacattgac 63

tgaccatgac 41

tgaccctgac 8

tgaccgtgac 13

tgaccttgac 26

tgacgatgac 29

tgacgctgac 9

tgacggtgac 27

tgacgttgac 34

tgactatgac 33

tgactctgac 30

tgactgtgac 27

tgactttgac 109

3 : 592

tgacaaatgac 25

tgacaactgac 13

tgacaagtgac 16

tgacaattgac 32

tgacacatgac 12

tgacacgtgac 11

tgacacttgac 17

tgacagatgac 6

tgacagctgac 3

tgacaggtgac 4

tgacagttgac 7

tgacatatgac 15

tgacatctgac 2

tgacatgtgac 15

tgacatttgac 31

tgaccaatgac 14

tgaccactgac 12

tgaccagtgac 10

tgaccattgac 20

tgacccatgac 5

tgacccctgac 2

tgacccgtgac 2

tgacccttgac 1

tgaccgatgac 4

tgaccggtgac 5

tgaccgttgac 14

tgacctatgac 2

tgacctctgac 3

tgacctgtgac 4

tgacctttgac 11

tgacgaatgac 6

tgacgactgac 9

tgacgagtgac 6

tgacgattgac 7

tgacgcatgac 2

tgacgcctgac 1

tgacgcgtgac 1

tgacgcttgac 1

tgacggatgac 4

tgacggctgac 4

tgacgggtgac 1

tgacggttgac 2

tgacgtatgac 3

tgacgtgtgac 6

tgacgtttgac 9

tgactaatgac 22

tgactactgac 8

tgactagtgac 16

tgactattgac 18

tgactcatgac 15

tgactcctgac 3

tgactcgtgac 6

tgactcttgac 14

tgactgatgac 12

tgactgctgac 5

tgactggtgac 9

tgactgttgac 14

tgacttatgac 11

tgacttctgac 7

tgacttgtgac 17

tgacttttgac 35

4 : 474

tgacaaaatgac 5

tgacaaactgac 7

tgacaaagtgac 4

tgacaaattgac 14

tgacaacatgac 3

tgacaacgtgac 1

tgacaacttgac 9

tgacaagatgac 2

tgacaagctgac 1

tgacaaggtgac 1

tgacaagttgac 7

tgacaatatgac 5

tgacaatctgac 7

tgacaatgtgac 3

tgacaatttgac 12

tgacacaatgac 6

tgacacactgac 1

tgacacagtgac 1

tgacacattgac 5

tgacaccatgac 1

tgacaccgtgac 1

tgacacgatgac 1

tgacacggtgac 1

tgacacgttgac 2

tgacactatgac 3

tgacactgtgac 1

tgacactttgac 8

tgacagaatgac 2

tgacagactgac 3

tgacagagtgac 4

tgacagattgac 1

tgacagcttgac 1

tgacaggctgac 3

tgacaggttgac 3

tgacagtatgac 2

tgacagtctgac 4

tgacagtttgac 1

tgacataatgac 10

tgacatactgac 3

tgacatagtgac 4

tgacatattgac 11

tgacatcatgac 2

tgacatcttgac 1

tgacatgatgac 2

tgacatgctgac 2

tgacatgttgac 1

tgacattatgac 4

tgacattctgac 2

tgacattgtgac 2

tgacattttgac 8

tgaccaaatgac 4

tgaccaactgac 1

tgaccaagtgac 3

tgaccaattgac 6

tgaccacatgac 3

tgaccacttgac 5

tgaccagatgac 1

tgaccagctgac 2

tgaccagttgac 3

tgaccatatgac 1

tgaccatctgac 3

tgaccatgtgac 2

tgaccatttgac 7

tgacccaatgac 3

tgacccagtgac 2

tgacccattgac 3

tgaccccatgac 2

tgacccgatgac 5

tgacccgctgac 2

tgacccggtgac 3

tgaccctgtgac 2

tgaccctttgac 2

tgaccgaatgac 1

tgaccgattgac 5

tgaccgtgtgac 1

tgaccgtttgac 4

tgacctaatgac 1

tgacctactgac 1

tgacctattgac 2

tgacctcttgac 1

tgacctgttgac 2

tgaccttatgac 1

tgaccttttgac 2

tgacgaaatgac 2

tgacgaactgac 1

tgacgaagtgac 1

tgacgaattgac 2

tgacgacatgac 2

tgacgacctgac 1

tgacgacgtgac 2

tgacgacttgac 3

tgacgagctgac 1

tgacgagttgac 3

tgacgatatgac 1

tgacgatgtgac 1

tgacgatttgac 1

tgacgcactgac 2

tgacgcattgac 1

tgacgccttgac 1

tgacgcgatgac 1

tgacgcggtgac 2

tgacgcgttgac 1

tgacgctgtgac 1

tgacgctttgac 3

tgacggactgac 1

tgacggagtgac 1

tgacggattgac 1

tgacgggctgac 1

tgacggtttgac 1

tgacgtactgac 2

tgacgtagtgac 2

tgacgtcatgac 1

tgacgtcgtgac 3

tgacgtcttgac 2

tgacgtggtgac 3

tgacgtgttgac 4

tgacgttatgac 1

tgacgttgtgac 1

tgactaaatgac 4

tgactaagtgac 2

tgactaattgac 6

tgactacatgac 1

tgactacttgac 5

tgactagctgac 6

tgactagttgac 5

tgactatatgac 6

tgactatgtgac 3

tgactatttgac 3

tgactcaatgac 4

tgactcactgac 2

tgactcagtgac 2

tgactccatgac 1

tgactccttgac 2

tgactcgatgac 1

tgactcggtgac 1

tgactcgttgac 2

tgactctatgac 1

tgactctctgac 1

tgactctgtgac 1

tgactctttgac 7

tgactgactgac 8

tgactgattgac 2

tgactgcatgac 3

tgactgcctgac 2

tgactggatgac 3

tgactggctgac 1

tgactgggtgac 1

tgactggttgac 1

tgactgtatgac 1

tgactgtctgac 1

tgactgtgtgac 1

tgactgtttgac 5

tgacttaatgac 2

tgacttactgac 2

tgacttagtgac 3

tgacttattgac 4

tgacttcatgac 1

tgacttcttgac 2

tgacttgatgac 2

tgacttgctgac 1

tgacttggtgac 2

tgacttgttgac 9

tgactttatgac 5

tgactttctgac 2

tgactttgtgac 3

tgactttttgac 10

5 : 414

tgacaaaaatgac 5

tgacaaaagtgac 2

tgacaaaattgac 7

tgacaaacatgac 2

tgacaaatatgac 1

tgacaaatgtgac 2

tgacaaatttgac 4

tgacaacaatgac 1

tgacaacattgac 1

tgacaaccttgac 1

tgacaacgatgac 1

tgacaacggtgac 1

tgacaactctgac 1

tgacaactgtgac 1

tgacaactttgac 2

tgacaagagtgac 2

tgacaagattgac 2

tgacaaggatgac 1

tgacaagtatgac 1

tgacaagtttgac 1

tgacaatactgac 1

tgacaatattgac 1

tgacaatcatgac 2

tgacaatgatgac 3

tgacaatgctgac 1

tgacaattatgac 1

tgacaattctgac 1

tgacaattttgac 2

tgacacaaatgac 1

tgacacacatgac 1

tgacacagctgac 1

tgacacaggtgac 1

tgacacagttgac 1

tgacacatgtgac 6

tgacacatttgac 2

tgacaccaatgac 1

tgacacccatgac 1

tgacacctatgac 1

tgacacgtatgac 1

tgacacgtgtgac 1

tgacactactgac 1

tgacactgatgac 1

tgacagaagtgac 1

tgacagacctgac 1

tgacagaggtgac 1

tgacagatctgac 1

tgacagatttgac 2

tgacagcaatgac 2

tgacagcgatgac 1

tgacagcgttgac 1

tgacagctttgac 1

tgacaggcatgac 1

tgacaggtttgac 1

tgacagtgatgac 2

tgacagttttgac 1

tgacataaatgac 2

tgacataagtgac 2

tgacatacatgac 1

tgacatacgtgac 1

tgacatatatgac 4

tgacatatttgac 2

tgacatcattgac 2

tgacatccttgac 1

tgacatctatgac 1

tgacatctctgac 1

tgacatctttgac 1

tgacatgaatgac 1

tgacatgagtgac 1

tgacatgcatgac 1

tgacatggatgac 2

tgacatgggtgac 1

tgacatgtatgac 2

tgacatgtgtgac 1

tgacatgtttgac 2

tgacattaatgac 1

tgacattagtgac 1

tgacattattgac 1

tgacattcttgac 1

tgacattgatgac 5

tgacattgctgac 1

tgacattgttgac 1

tgacatttatgac 1

tgacatttgtgac 2

tgacatttttgac 1

tgaccaaattgac 1

tgaccaagatgac 1

tgaccaatatgac 1

tgaccaatgtgac 1

tgaccaatttgac 1

tgaccacactgac 1

tgaccacagtgac 1

tgaccacgatgac 1

tgaccactatgac 1

tgaccagaatgac 1

tgaccagagtgac 1

tgaccagtgtgac 1

tgaccagtttgac 1

tgaccatcctgac 1

tgaccatgatgac 1

tgaccattctgac 1

tgaccattttgac 1

tgacccaactgac 1

tgacccacctgac 1

tgacccacttgac 1

tgacccatgtgac 2

tgacccatttgac 2

tgacccctatgac 1

tgacccctttgac 1

tgacccggttgac 1

tgaccctgttgac 1

tgaccgagatgac 1

tgaccgagttgac 2

tgaccgctttgac 1

tgaccggagtgac 2

tgaccggcatgac 1

tgaccggtgtgac 1

tgaccgtaatgac 1

tgaccgttgtgac 1

tgaccgttttgac 1

tgacctaaatgac 1

tgacctaggtgac 1

tgacctagttgac 1

tgacctccatgac 1

tgacctccgtgac 1

tgacctcgttgac 1

tgacctgaatgac 1

tgacctgcatgac 1

tgaccttattgac 1

tgacctttgtgac 1

tgacctttttgac 2

tgacgaaaatgac 3

tgacgaaagtgac 1

tgacgaaattgac 3

tgacgaacttgac 1

tgacgaagttgac 1

tgacgaatatgac 1

tgacgaatttgac 3

tgacgacaatgac 1

tgacgacagtgac 1

tgacgacattgac 1

tgacgactatgac 1

tgacgagagtgac 1

tgacgagcatgac 2

tgacgagcgtgac 1

tgacgaggatgac 1

tgacgagtctgac 1

tgacgagtgtgac 1

tgacgatattgac 3

tgacgatgatgac 3

tgacgatggtgac 1

tgacgattctgac 2

tgacgattttgac 2

tgacgcacatgac 1

tgacgcacttgac 1

tgacgcagctgac 1

tgacgcgtttgac 2

tgacgctactgac 1

tgacgctcatgac 1

tgacgcttttgac 1

tgacggaattgac 1

tgacggagatgac 1

tgacggaggtgac 2

tgacggatatgac 1

tgacggatctgac 2

tgacggatttgac 1

tgacggcactgac 1

tgacggcggtgac 1

tgacggcgttgac 1

tgacggctgtgac 1

tgacggctttgac 1

tgacgggaatgac 1

tgacgggtgtgac 1

tgacgggtttgac 1

tgacggtcatgac 1

tgacggtgttgac 1

tgacggttatgac 1

tgacgtagatgac 1

tgacgtcattgac 6

tgacgtgagtgac 1

tgacgtggatgac 1

tgacgtgtatgac 1

tgacgtgtctgac 1

tgacgttaatgac 1

tgacgttcttgac 3

tgacgtttgtgac 1

tgacgtttttgac 1

tgactaaaatgac 2

tgactaacttgac 1

tgactaatatgac 1

tgactaatctgac 2

tgactacagtgac 1

tgactacattgac 1

tgactacgatgac 1

tgactacgttgac 1

tgactagaatgac 3

tgactagactgac 3

tgactagattgac 1

tgactagcatgac 1

tgactagcttgac 1

tgactagtgtgac 1

tgactagtttgac 1

tgactataatgac 1

tgactatactgac 1

tgactatagtgac 1

tgactatattgac 2

tgactatcatgac 1

tgactatcttgac 1

tgactatgatgac 3

tgactatggtgac 1

tgactatgttgac 1

tgactattatgac 1

tgactattgtgac 2

tgactattttgac 2

tgactcaaatgac 1

tgactcacgtgac 1

tgactcagttgac 1

tgactcatatgac 2

tgactcatgtgac 3

tgactcatttgac 3

tgactccagtgac 1

tgactccattgac 2

tgactccgatgac 1

tgactcgagtgac 1

tgactcgattgac 1

tgactcgcttgac 1

tgactctagtgac 1

tgactctgatgac 1

tgactctgctgac 1

tgactctgttgac 1

tgactcttgtgac 1

tgactcttttgac 1

tgactgaaatgac 2

tgactgagttgac 2

tgactgatatgac 1

tgactgatttgac 1

tgactgcactgac 1

tgactgcattgac 2

tgactgctgtgac 1

tgactggactgac 1

tgactggattgac 1

tgactggtatgac 1

tgactggtgtgac 1

tgactggtttgac 1

tgactgtaatgac 1

tgactgtattgac 1

tgactgtcatgac 2

tgactgttatgac 1

tgactgttgtgac 1

tgactgttttgac 2

tgacttaaatgac 4

tgacttaactgac 2

tgacttaattgac 3

tgacttacatgac 2

tgacttacttgac 4

tgacttaggtgac 1

tgacttagttgac 2

tgacttcaatgac 7

tgacttcactgac 2

tgacttcagtgac 2

tgacttcgttgac 1

tgacttctttgac 1

tgacttgaatgac 1

tgacttgagtgac 1

tgacttgcgtgac 1

tgacttgcttgac 2

tgacttgggtgac 1

tgacttggttgac 1

tgacttgtatgac 2

tgacttgtctgac 1

tgacttgtgtgac 1

tgactttaatgac 2

tgactttactgac 2

tgactttagtgac 1

tgactttattgac 1

tgactttcttgac 4

tgactttgatgac 4

tgactttgctgac 1

tgacttttatgac 2

tgacttttctgac 1

tgacttttgtgac 2

tgacttttttgac 6

6 : 375

tgacaaaaagtgac 2

tgacaaaagttgac 1

tgacaaacactgac 1

tgacaaaccatgac 1

tgacaaactatgac 2

tgacaaagaatgac 1

tgacaaagtttgac 1

tgacaaatactgac 1

tgacaaatattgac 1

tgacaaatgatgac 1

tgacaaattgtgac 1

tgacaaattttgac 1

tgacaacaagtgac 2

tgacaaccaatgac 3

tgacaacttatgac 1

tgacaacttgtgac 1

tgacaaggaatgac 1

tgacaaggcttgac 1

tgacaataattgac 1

tgacaatcactgac 1

tgacaatcgatgac 1

tgacaatgcatgac 1

tgacaatggatgac 1

tgacaattaatgac 1

tgacaatttatgac 1

tgacaatttctgac 1

tgacacaagatgac 1

tgacacaatatgac 1

tgacacacactgac 2

tgacacactttgac 3

tgacacatattgac 3

tgacacattttgac 4

tgacaccaagtgac 1

tgacaccatctgac 1

tgacacgccatgac 1

tgacacgctttgac 1

tgacacgtgttgac 1

tgacactatttgac 1

tgacacttcgtgac 1

tgacacttggtgac 1

tgacagaaattgac 1

tgacagaagatgac 1

tgacagaagttgac 1

tgacagaatttgac 1

tgacagattctgac 1

tgacagattttgac 1

tgacagcaattgac 2

tgacagcccttgac 1

tgacagcgagtgac 1

tgacagctaatgac 2

tgacaggttttgac 1

tgacagtaactgac 1

tgacagtatgtgac 1

tgacagtcggtgac 1

tgacagttcttgac 1

tgacagtttttgac 1

tgacataagctgac 1

tgacataagttgac 1

tgacatactatgac 1

tgacatatactgac 2

tgacatatagtgac 1

tgacatatattgac 2

tgacatcaattgac 1

tgacatcacatgac 1

tgacatcatttgac 1

tgacatcgcgtgac 1

tgacatgacatgac 1

tgacatgttttgac 2

tgacattaaatgac 1

tgacattaattgac 2

tgacattcaatgac 2

tgacattctttgac 1

tgacatttgatgac 1

tgacatttggtgac 1

tgaccaaaaatgac 2

tgaccaaagctgac 1

tgaccaaatctgac 1

tgaccaaatttgac 1

tgaccaacgttgac 1

tgaccaagcttgac 1

tgaccaatgatgac 1

tgaccaatgttgac 1

tgaccaccattgac 2

tgaccacttttgac 1

tgaccagcgatgac 1

tgaccaggattgac 1

tgaccagttttgac 1

tgaccatcgatgac 1

tgaccatgagtgac 1

tgaccatgattgac 1

tgaccatgtttgac 1

tgaccattactgac 2

tgaccattcctgac 1

tgaccattgctgac 1

tgacccaagatgac 1

tgacccaatctgac 1

tgacccatgctgac 1

tgacccattttgac 13

tgaccccacgtgac 1

tgacccgacctgac 1

tgacccgacttgac 1

tgacccgtgatgac 2

tgaccctccttgac 1

tgaccctctttgac 1

tgaccctgagtgac 1

tgacccttcttgac 1

tgaccctttttgac 1

tgaccgacgatgac 1

tgaccgagtctgac 2

tgaccgatcatgac 1

tgaccgattttgac 1

tgaccgcacgtgac 1

tgaccggagttgac 1

tgaccggcgttgac 1

tgaccggtgatgac 1

tgaccggtgttgac 2

tgaccgtcgatgac 1

tgacctaagatgac 1

tgacctaagttgac 1

tgacctaatctgac 1

tgacctagattgac 1

tgacctaggttgac 1

tgacctatcttgac 1

tgacctatgttgac 2

tgacctattttgac 1

tgacctcagttgac 1

tgacctgacctgac 1

tgacctgcattgac 2

tgacctgctatgac 1

tgacctggcatgac 1

tgacctggcttgac 1

tgacctgtgctgac 1

tgacctgttctgac 1

tgaccttccatgac 1

tgaccttccgtgac 1

tgaccttgaatgac 1

tgaccttttgtgac 1

tgacgaaacatgac 1

tgacgaaatctgac 1

tgacgaaatttgac 2

tgacgaagggtgac 1

tgacgaattgtgac 1

tgacgacctctgac 1

tgacgacctttgac 1

tgacgactcatgac 1

tgacgagacttgac 1

tgacgagatttgac 1

tgacgagcgatgac 1

tgacgagcggtgac 1

tgacgagctgtgac 2

tgacgagtattgac 1

tgacgatacatgac 1

tgacgatagatgac 1

tgacgatatctgac 1

tgacgatatttgac 1

tgacgatcggtgac 1

tgacgatctctgac 1

tgacgattaatgac 1

tgacgattattgac 1

tgacgatttttgac 1

tgacgccgactgac 1

tgacgccttttgac 1

tgacggagaatgac 1

tgacggatattgac 1

tgacggccggtgac 1

tgacggcgattgac 1

tgacggcgcgtgac 3

tgacggcttgtgac 1

tgacggggaatgac 1

tgacggtctttgac 1

tgacggttaatgac 1

tgacgtaacatgac 1

tgacgtacagtgac 1

tgacgtcaaatgac 2

tgacgtcaattgac 2

tgacgtcatctgac 1

tgacgtccgttgac 1

tgacgtcgtgtgac 1

tgacgtgacttgac 1

tgacgtgatatgac 1

tgacgtgctttgac 1

tgacgtggaatgac 2

tgacgtgtaatgac 2

tgacgtgtcgtgac 1

tgacgttaaatgac 1

tgacgttaattgac 1

tgacgttatctgac 1

tgacgttatgtgac 1

tgacgttttttgac 1

tgactaaaaatgac 1

tgactaacaatgac 1

tgactaagggtgac 1

tgactaatagtgac 1

tgactaatattgac 3

tgactaatgttgac 1

tgactaattgtgac 1

tgactaattttgac 1

tgactacaggtgac 1

tgactacccttgac 1

tgactactactgac 1

tgactacttgtgac 1

tgactagacttgac 1

tgactagatctgac 1

tgactagggctgac 1

tgactagggttgac 1

tgactaggtttgac 2

tgactagtactgac 1

tgactagtagtgac 1

tgactataattgac 1

tgactatagatgac 2

tgactatcattgac 1

tgactatgaatgac 1

tgactatgattgac 3

tgactattactgac 1

tgactattagtgac 1

tgactattattgac 1

tgactattcttgac 1

tgactatttgtgac 1

tgactcaacgtgac 1

tgactcaatatgac 1

tgactcacagtgac 1

tgactcagcgtgac 1

tgactcataatgac 1

tgactcatgatgac 1

tgactcattttgac 7

tgactccacgtgac 1

tgactccatatgac 1

tgactcgaattgac 1

tgactcgctctgac 1

tgactcggtttgac 1

tgactctcaatgac 1

tgactctctctgac 1

tgactctgtttgac 1

tgactcttagtgac 1

tgactcttgttgac 1

tgactctttttgac 1

tgactgaagatgac 1

tgactgaatttgac 1

tgactgactatgac 1

tgactgactgtgac 1

tgactgagactgac 1

tgactgaggctgac 1

tgactgataatgac 1

tgactgatggtgac 1

tgactgattttgac 1

tgactggaaatgac 1

tgactggtaatgac 1

tgactggtattgac 1

tgactggtgatgac 1

tgactgtaattgac 2

tgactgtctctgac 1

tgactgtctttgac 1

tgactgtgagtgac 1

tgactgtgattgac 1

tgactgtggatgac 1

tgactgtgtatgac 1

tgactgttgttgac 1

tgactgtttctgac 1

tgactgtttgtgac 1

tgacttaaagtgac 1

tgacttacattgac 1

tgacttagcatgac 1

tgacttatgatgac 2

tgacttatgttgac 1

tgacttattgtgac 1

tgacttcacatgac 1

tgacttcagatgac 2

tgacttcagctgac 1

tgacttcatatgac 1

tgacttcatctgac 1

tgacttccattgac 1

tgacttcctatgac 1

tgacttcctgtgac 1

tgacttcctttgac 1

tgacttctcgtgac 2

tgacttctgatgac 1

tgacttcttatgac 1

tgacttcttttgac 2

tgacttgacttgac 2

tgacttgagttgac 1

tgacttgctgtgac 3

tgacttggagtgac 1

tgacttggtatgac 1

tgacttgtcttgac 1

tgactttacatgac 1

tgactttatatgac 1

tgactttatttgac 1

tgactttcactgac 1

tgactttcattgac 1

tgactttccatgac 2

tgactttcgttgac 1

tgactttgaatgac 1

tgactttgactgac 1

tgactttgagtgac 1

tgactttggttgac 1

tgactttgtgtgac 1

tgactttgtttgac 2

tgacttttaatgac 1

tgacttttcatgac 1

tgacttttcttgac 1

tgacttttgttgac 3

tgactttttctgac 1

tgactttttgtgac 1

7 : 339

tgacaaaaccgtgac 1

tgacaaaagtctgac 1

tgacaaaagtttgac 1

tgacaaaataatgac 1

tgacaaaatactgac 1

tgacaaaatgatgac 1

tgacaaaattatgac 1

tgacaaacacttgac 1

tgacaaacgtgtgac 2

tgacaaactattgac 1

tgacaaactcgtgac 1

tgacaaagagctgac 1

tgacaaagcactgac 1

tgacaaagcagtgac 1

tgacaaagccgtgac 1

tgacaaatacatgac 1

tgacaaatagttgac 1

tgacaaattactgac 1

tgacaaattagtgac 1

tgacaaatttatgac 1

tgacaacaaagtgac 1

tgacaacaaattgac 1

tgacaacatgatgac 1

tgacaacattttgac 1

tgacaactttctgac 1

tgacaagactatgac 1

tgacaagagcatgac 1

tgacaagatcttgac 1

tgacaagctggtgac 1

tgacaaggaaatgac 2

tgacaaggctatgac 1

tgacaagtaaatgac 1

tgacaagtatatgac 1

tgacaagtatttgac 1

tgacaagtctatgac 1

tgacaagttcttgac 1

tgacaagttgatgac 2

tgacaagtttatgac 1

tgacaataaattgac 1

tgacaatcatgtgac 1

tgacaattctatgac 1

tgacaattgcttgac 1

tgacaattggttgac 1

tgacacaaatttgac 1

tgacacaagcatgac 1

tgacacaagtctgac 1

tgacacaccgttgac 1

tgacacagttgtgac 1

tgacacatctatgac 1

tgacacatgtttgac 1

tgacacattgatgac 1

tgacaccaccatgac 1

tgacaccatattgac 1

tgacacgtgcgtgac 1

tgacacgtgggtgac 1

tgacacgtgtctgac 1

tgacactactttgac 1

tgacactataatgac 1

tgacactatagtgac 1

tgacactcaaatgac 1

tgacactgaattgac 1

tgacactgacatgac 1

tgacagacacgtgac 1

tgacagagaggtgac 1

tgacagatgtttgac 1

tgacagattcatgac 1

tgacagcacattgac 1

tgacagcattttgac 1

tgacagcgacttgac 1

tgacagctcggtgac 1

tgacagctgtttgac 1

tgacaggacattgac 1

tgacagggttatgac 1

tgacagtaacatgac 1

tgacagtaacttgac 1

tgacagtactgtgac 1

tgacagtgaagtgac 1

tgacagtgatatgac 1

tgacagtgtagtgac 1

tgacagttggttgac 1

tgacagtttcgtgac 1

tgacagtttcttgac 1

tgacataaacatgac 1

tgacataaagatgac 1

tgacataacgatgac 1

tgacataactttgac 1

tgacataccaatgac 1

tgacatatctatgac 1

tgacatatgaatgac 1

tgacatattcatgac 1

tgacatattcttgac 1

tgacatattgctgac 1

tgacatcaccttgac 1

tgacatccaaatgac 1

tgacatcgttttgac 1

tgacatgaaactgac 1

tgacatgaatgtgac 1

tgacatgacaatgac 1

tgacatgacagtgac 1

tgacatgggtatgac 1

tgacatggtcttgac 1

tgacatgtcaatgac 1

tgacattaaaatgac 1

tgacattcatgtgac 1

tgacattccattgac 1

tgacattggtttgac 1

tgacatttgagtgac 1

tgacatttgtatgac 1

tgacatttgtttgac 1

tgacattttcatgac 1

tgacattttcttgac 1

tgacattttggtgac 1

tgaccaaactttgac 1

tgaccaaataatgac 2

tgaccaaattgtgac 1

tgaccaactagtgac 1

tgaccaacttttgac 1

tgaccaatagttgac 1

tgaccaatgggtgac 1

tgaccaatgtttgac 1

tgaccaattaatgac 1

tgaccaattgttgac 1

tgaccaatttctgac 1

tgaccacaacatgac 1

tgaccacagtttgac 2

tgaccacatcatgac 1

tgaccaccacttgac 1

tgaccagacagtgac 1

tgaccagtagttgac 1

tgaccagtgattgac 1

tgaccagttgctgac 1

tgaccataacatgac 1

tgaccataatgtgac 1

tgaccatgtattgac 1

tgaccatgtcatgac 1

tgaccattgattgac 1

tgaccattggttgac 1

tgaccatttcgtgac 1

tgacccaaatttgac 1

tgacccacttctgac 2

tgacccagatctgac 1

tgacccatacatgac 1

tgacccatatatgac 1

tgacccatccatgac 1

tgacccattcatgac 1

tgaccccattctgac 1

tgacccctcagtgac 1

tgacccgacaatgac 1

tgacccgcacgtgac 1

tgacccgttcctgac 1

tgaccgaccgttgac 1

tgaccgacttgtgac 1

tgaccgagtcgtgac 1

tgaccgatttctgac 1

tgaccgcatgctgac 1

tgaccggaagatgac 1

tgaccggaccatgac 1

tgaccggagactgac 1

tgaccggtgtgtgac 1

tgaccgtactctgac 1

tgaccgtgtggtgac 1

tgaccgttccttgac 1

tgacctaaagttgac 1

tgacctaattgtgac 1

tgacctagctttgac 1

tgacctatctttgac 1

tgacctcaaactgac 3

tgacctcacagtgac 1

tgacctctcaatgac 1

tgacctctctctgac 1

tgacctctctttgac 1

tgacctcttactgac 1

tgacctgtctttgac 1

tgaccttcaagtgac 1

tgaccttgaagtgac 1

tgaccttgtcgtgac 1

tgacctttccctgac 2

tgaccttttcttgac 1

tgacgaaagtttgac 1

tgacgaaattatgac 1

tgacgaacctttgac 1

tgacgaagacgtgac 1

tgacgaatctgtgac 1

tgacgacaaattgac 1

tgacgacgaattgac 1

tgacgactttatgac 1

tgacgactttctgac 1

tgacgagattttgac 1

tgacgaggtactgac 1

tgacgagttgatgac 1

tgacgagttgctgac 1

tgacgatccgttgac 1

tgacgatgaattgac 1

tgacgatgctgtgac 1

tgacgattaagtgac 1

tgacgattttgtgac 2

tgacgcaaacttgac 1

tgacgcaattctgac 1

tgacggaactatgac 1

tgacggatcgatgac 1

tgacggctctttgac 1

tgacgggcaagtgac 1

tgacgggctagtgac 1

tgacgggttcctgac 1

tgacggtgcagtgac 1

tgacggtggcgtgac 1

tgacggttctgtgac 1

tgacgtacgagtgac 1

tgacgtacgcgtgac 1

tgacgtatctttgac 1

tgacgtcacactgac 1

tgacgtcatcatgac 1

tgacgtcatcttgac 1

tgacgtcatgttgac 1

tgacgtcctggtgac 1

tgacgtcgacatgac 1

tgacgtcgttgtgac 1

tgacgtcttcttgac 1

tgacgtgaaaatgac 1

tgacgtgagcatgac 1

tgacgtggaagtgac 1

tgacgtgtcgatgac 1

tgacgtgtttatgac 1

tgacgttaaaatgac 1

tgacgttacggtgac 1

tgacgttgatttgac 1

tgacgttgccttgac 1

tgacgtttcattgac 1

tgacgtttgtttgac 1

tgacgttttcatgac 1

tgactaaaacgtgac 1

tgactaaacagtgac 1

tgactaaacgttgac 1

tgactaactactgac 1

tgactaacttgtgac 1

tgactaataaatgac 1

tgactaatagatgac 1

tgactaatagttgac 1

tgactaatatgtgac 1

tgactaatccatgac 1

tgactaatgaatgac 1

tgactaattgttgac 1

tgactaatttgtgac 1

tgactaatttttgac 1

tgactaccctctgac 1

tgactacctcttgac 1

tgactacgtcctgac 1

tgactagaagctgac 1

tgactagattgtgac 1

tgactagcttgtgac 1

tgactaggatctgac 1

tgactaggcaatgac 1

tgactatagattgac 1

tgactatatattgac 2

tgactatcaaatgac 2

tgactatgaagtgac 1

tgactatgagttgac 1

tgactatgtgatgac 1

tgactatgttatgac 1

tgactattcaatgac 1

tgactattgtctgac 1

tgactattttgtgac 1

tgactcagcattgac 1

tgactcagtcgtgac 1

tgactcattcttgac 1

tgactccaaactgac 1

tgactccacaatgac 1

tgactccgacatgac 1

tgactcctctgtgac 1

tgactcgcacgtgac 1

tgactcggtggtgac 1

tgactcgtatatgac 1

tgactcgtccgtgac 1

tgactcgtgactgac 1

tgactcgttcctgac 1

tgactctcctgtgac 1

tgactctcggctgac 1

tgactctgaaatgac 1

tgactctgcggtgac 1

tgactcttaaatgac 1

tgactcttctgtgac 1

tgactcttctttgac 1

tgactcttggatgac 1

tgactctttcatgac 1

tgactgaaatttgac 1

tgactgaataatgac 1

tgactgattattgac 1

tgactgatttgtgac 1

tgactgcagtttgac 1

tgactggtgaatgac 1

tgactggttaatgac 1

tgactgtactatgac 1

tgactgtagaatgac 1

tgacttaacactgac 1

tgacttaccaatgac 1

tgacttatatatgac 1

tgacttatctttgac 1

tgacttcaccttgac 1

tgacttccatctgac 1

tgacttcgatttgac 1

tgacttctgcatgac 1

tgacttgaacatgac 1

tgacttgaatttgac 1

tgacttgagtttgac 1

tgacttgatcatgac 1

tgacttgcctatgac 3

tgacttggatatgac 1

tgacttgtagctgac 2

tgacttgtccttgac 1

tgacttgtgagtgac 1

tgacttgttaatgac 1

tgactttatcttgac 1

tgactttatgttgac 1

tgactttattatgac 1

tgactttcgtatgac 1

tgactttcttatgac 1

tgactttgatttgac 1

tgactttgcgttgac 1

tgactttggtgtgac 1

tgactttgtaatgac 1

tgactttgtattgac 1

tgacttttcgttgac 1

tgactttttgatgac 1

tgactttttgttgac 1

8 : 436

tgacaaaaaatatgac 1

tgacaaaaaccatgac 1

tgacaaaaacgatgac 1

tgacaaaaacgttgac 1

tgacaaaaactatgac 1

tgacaaaacctttgac 1

tgacaaaagcaatgac 1

tgacaaaatatgtgac 2

tgacaaaatcgttgac 1

tgacaaaatttatgac 1

tgacaaacaaagtgac 1

tgacaaagaatttgac 1

tgacaaagcatatgac 1

tgacaaagtttatgac 1

tgacaaataaattgac 1

tgacaaatatcttgac 1

tgacaaatccggtgac 1

tgacaaattgtttgac 1

tgacaacaacaatgac 1

tgacaacaactatgac 1

tgacaacaatggtgac 1

tgacaacaccagtgac 1

tgacaacatatatgac 1

tgacaacatctttgac 1

tgacaacgaagatgac 1

tgacaacttgtgtgac 2

tgacaagaaatttgac 1

tgacaagaccaatgac 1

tgacaagatccctgac 1

tgacaaggttattgac 1

tgacaagtagattgac 1

tgacaagtatagtgac 1

tgacaagttagttgac 1

tgacaagtttgatgac 1

tgacaataaatatgac 1

tgacaataatgatgac 1

tgacaatagccgtgac 1

tgacaatataagtgac 1

tgacaatataattgac 1

tgacaatattattgac 1

tgacaatattcttgac 2

tgacaatccgactgac 1

tgacaatcgttttgac 1

tgacaatctctttgac 1

tgacaatgacaatgac 1

tgacaatgattttgac 1

tgacaatgcccatgac 1

tgacaatggcaatgac 1

tgacaatgtcattgac 1

tgacaattattttgac 1

tgacaattgacttgac 1

tgacacaattgatgac 1

tgacacaattgctgac 1

tgacacacaagctgac 1

tgacacagaacgtgac 1

tgacacagccactgac 1

tgacacataccatgac 1

tgacacatactttgac 3

tgacacattctctgac 1

tgacacattggatgac 1

tgacaccatatatgac 1

tgacaccattattgac 1

tgacaccctctatgac 1

tgacaccgcgtttgac 1

tgacacgatagttgac 1

tgacacgcataatgac 1

tgacacggtgcctgac 1

tgacactgtttgtgac 1

tgacacttggcgtgac 1

tgacagaaaaaatgac 1

tgacagaaaatctgac 1

tgacagaagcagtgac 1

tgacagaatatttgac 1

tgacagaccttgtgac 1

tgacagagaacttgac 1

tgacagatactttgac 1

tgacagatagattgac 1

tgacagcctggttgac 1

tgacagcgttattgac 1

tgacagctgagctgac 1

tgacaggaatactgac 1

tgacaggagaattgac 3

tgacaggtgatgtgac 1

tgacaggttacttgac 1

tgacagtaaaagtgac 1

tgacagtagcgctgac 1

tgacagtgatgatgac 1

tgacagttggtttgac 1

tgacataaaatctgac 1

tgacataacagctgac 1

tgacataatggatgac 1

tgacataattagtgac 1

tgacataatttttgac 1

tgacatactgtatgac 1

tgacataggaattgac 1

tgacatatacactgac 2

tgacatatatagtgac 1

tgacatatattttgac 1

tgacatatgcaatgac 1

tgacatattaattgac 1

tgacatattccctgac 1

tgacatcaagcatgac 1

tgacatcacatgtgac 1

tgacatcatatgtgac 1

tgacatcatccttgac 1

tgacatcatgcatgac 1

tgacatcatgtgtgac 1

tgacatgaaaggtgac 1

tgacatgacgtttgac 1

tgacatgagttatgac 2

tgacatggaggatgac 1

tgacatgggcaatgac 2

tgacatgtaaattgac 3

tgacatgtactatgac 1

tgacatgtatggtgac 1

tgacattacatttgac 1

tgacattataaatgac 1

tgacattatcagtgac 1

tgacattatttctgac 1

tgacattatttttgac 1

tgacattcaaaatgac 1

tgacattccacgtgac 1

tgacattccttatgac 1

tgacattcgtgctgac 1

tgacattgactttgac 1

tgacattgagcttgac 1

tgacattgatgttgac 1

tgacatttagggtgac 1

tgacatttctcttgac 1

tgacattttaattgac 1

tgacatttttgttgac 1

tgaccaaagcgttgac 1

tgaccaaatccatgac 1

tgaccaaatccttgac 1

tgaccaacaaactgac 1

tgaccaatcagatgac 1

tgaccaatctattgac 1

tgaccaatgatatgac 1

tgaccaatttagtgac 1

tgaccaatttattgac 1

tgaccacaatgatgac 1

tgaccacattattgac 1

tgaccaccgtactgac 1

tgaccagacaagtgac 1

tgaccagcacactgac 1

tgaccaggtttttgac 1

tgaccagtacactgac 1

tgaccagtgatgtgac 1

tgaccagttatatgac 1

tgaccatacttttgac 1

tgaccatcctcatgac 1

tgaccatctttttgac 1

tgaccatgcgtgtgac 1

tgaccatgtccatgac 1

tgaccattaacatgac 1

tgaccattaatgtgac 1

tgaccattgttgtgac 1

tgacccaatgattgac 1

tgacccaccaactgac 1

tgacccacgagttgac 1

tgacccactttttgac 1

tgacccagcgtatgac 1

tgacccatgagttgac 1

tgaccccaaatttgac 1

tgaccccgtcagtgac 1

tgacccgaaccgtgac 1

tgacccgactaatgac 1

tgacccgagccgtgac 2

tgacccgagtgttgac 1

tgacccgtaaaatgac 1

tgacccgtctaatgac 1

tgaccctaaaaatgac 1

tgaccctagggatgac 1

tgaccctgaagttgac 1

tgaccctgaatatgac 1

tgacccttgcgttgac 1

tgaccgagataatgac 1

tgaccggagcgttgac 1

tgaccggctatctgac 1

tgaccggttgagtgac 1

tgaccgtccatttgac 1

tgaccgtctagatgac 1

tgaccgtgactgtgac 1

tgaccgttgagatgac 1

tgacctaaacgatgac 1

tgacctaagatttgac 1

tgacctaagtaatgac 1

tgacctacaatctgac 1

tgacctagagagtgac 1

tgacctagcaaatgac 1

tgacctagtttttgac 1

tgacctatttattgac 1

tgacctcagcagtgac 1

tgacctcatttgtgac 1

tgacctcctacctgac 1

tgacctcggctttgac 1

tgacctgagtgatgac 1

tgacctgcaatatgac 1

tgacctgcgtattgac 1

tgacctggtacatgac 1

tgaccttaaccttgac 1

tgaccttcatactgac 1

tgaccttctaaatgac 1

tgaccttgaaggtgac 1

tgaccttgaccttgac 1

tgacctttctattgac 1

tgacctttgtgatgac 1

tgacctttgtgttgac 1

tgacgaaaataatgac 1

tgacgaaacgtatgac 1

tgacgaacacattgac 1

tgacgaagaagatgac 1

tgacgaagaagttgac 1

tgacgaagacgttgac 1

tgacgaagatggtgac 1

tgacgaagcagatgac 1

tgacgaatgcaatgac 1

tgacgacgagattgac 1

tgacgacgataatgac 1

tgacgactaagctgac 1

tgacgacttcagtgac 1

tgacgagagatttgac 1

tgacgagagcggtgac 1

tgacgagagtgctgac 1

tgacgagataattgac 1

tgacgaggtctctgac 1

tgacgataacgatgac 1

tgacgatatgtgtgac 1

tgacgatgaagatgac 1

tgacgatgacggtgac 1

tgacgatgaggatgac 1

tgacgatgatgatgac 1

tgacgatgcttttgac 1

tgacgatggccttgac 1

tgacgatggtgatgac 1

tgacgatgttgatgac 1

tgacgattatagtgac 1

tgacgattttgttgac 1

tgacgcaacccgtgac 1

tgacgcaagcaatgac 1

tgacgcagactctgac 1

tgacgcattcgatgac 1

tgacgccattcgtgac 1

tgacgccgttcttgac 1

tgacgcctaacctgac 1

tgacgcggatcgtgac 1

tgacgcggatggtgac 1

tgacggaagacttgac 1

tgacggaagcaatgac 1

tgacggatggtttgac 1

tgacggcaaaattgac 1

tgacggcgagcatgac 1

tgacggcggagatgac 1

tgacgggccatttgac 1

tgacgggtctcttgac 1

tgacggtacacgtgac 1

tgacggtcttgttgac 1

tgacggtgagagtgac 1

tgacgtaactattgac 1

tgacgtaagcaatgac 1

tgacgtaaggtctgac 1

tgacgtaccttctgac 1

tgacgtacttagtgac 1

tgacgtataaaatgac 1

tgacgtatggagtgac 1

tgacgtcagcattgac 1

tgacgtcatcgttgac 1

tgacgtcattcctgac 1

tgacgtcgacactgac 1

tgacgtcggcaatgac 1

tgacgtgaaccatgac 1

tgacgtgagttttgac 1

tgacgtggcatgtgac 2

tgacgtgtcgtctgac 1

tgacgtgttgcttgac 1

tgacgttaacgttgac 1

tgacgttatacttgac 1

tgacgttgaaaatgac 1

tgacgttgctaatgac 1

tgacgttggcaatgac 1

tgacgtttcagatgac 1

tgacgtttcctttgac 1

tgacgtttcggctgac 1

tgacgttttctttgac 1

tgactaaacaaatgac 1

tgactaaacacatgac 1

tgactaaactgttgac 1

tgactaaagacatgac 1

tgactaaagatttgac 1

tgactaaagctttgac 1

tgactaacaatttgac 1

tgactaacccaatgac 1

tgactaagggcttgac 1

tgactaagtgggtgac 1

tgactaataacatgac 1

tgactaattgtgtgac 1

tgactacaattttgac 1

tgactacatatgtgac 1

tgactacatcattgac 1

tgactacggaaatgac 1

tgactactagcatgac 1

tgactactcaaatgac 1

tgactacttcgctgac 1

tgactacttctctgac 1

tgactagagaaatgac 1

tgactagaggggtgac 1

tgactagccttttgac 1

tgactagtaaaatgac 1

tgactataaaagtgac 1

tgactataataatgac 1

tgactatatatatgac 1

tgactatattgctgac 1

tgactatgactgtgac 1

tgactatgcacatgac 1

tgactatgctaatgac 1

tgactatgctcatgac 1

tgactatgtgtatgac 1

tgactatgtttgtgac 1

tgactattttgctgac 1

tgactcacaatttgac 1

tgactcacataatgac 1

tgactcacgaaatgac 1

tgactcatcagatgac 1

tgactcatgtcgtgac 1

tgactcattgattgac 1

tgactcattgcttgac 1

tgactccaaaaatgac 1

tgactccggcactgac 1

tgactccggcgttgac 1

tgactcgaaaattgac 1

tgactcgaatgatgac 1

tgactcgctacatgac 1

tgactcggcttgtgac 1

tgactcggggattgac 1

tgactcggtgtttgac 1

tgactcgtcacgtgac 1

tgactcgtgagttgac 1

tgactcgttttatgac 1

tgactctacctgtgac 1

tgactctatctttgac 1

tgactctccctttgac 1

tgactctgactctgac 1

tgactctgattatgac 1

tgactctgcctgtgac 1

tgactctggtactgac 1

tgactctttccttgac 1

tgactgaaagtttgac 1

tgactgaatagttgac 1

tgactgactgactgac 1

tgactgagaggatgac 1

tgactgatttgttgac 1

tgactgctcttctgac 2

tgactgctcttgtgac 2

tgactggtcttttgac 1

tgactgtaaccatgac 1

tgactgtattagtgac 1

tgactgtattgctgac 1

tgactgtcagcatgac 1

tgactgtcctattgac 1

tgactgtctttgtgac 1

tgactgtgaccgtgac 1

tgactgtggtaatgac 1

tgactgttagtgtgac 1

tgactgttccgttgac 1

tgactgtttaagtgac 1

tgactgtttgcttgac 1

tgactgttttactgac 1

tgactgtttttatgac 1

tgacttaaaagctgac 1

tgacttaaagcttgac 1

tgacttaagtaatgac 1

tgacttaataaatgac 1

tgacttaatgtctgac 1

tgacttaattcttgac 1

tgacttacctcttgac 1

tgacttagatggtgac 1

tgacttagcgggtgac 1

tgacttaggtgatgac 1

tgacttattgtgtgac 1

tgacttatttggtgac 1

tgacttatttgttgac 2

tgacttcaattttgac 1

tgacttcacccctgac 1

tgacttcatagttgac 1

tgacttcatcactgac 1

tgacttcggcgttgac 2

tgacttctaacttgac 1

tgacttctacgttgac 1

tgacttctcctatgac 1

tgacttcttccttgac 1

tgacttcttggttgac 1

tgacttgacacatgac 1

tgacttgagtcctgac 1

tgacttgcaaggtgac 1

tgacttgccgattgac 1

tgacttggagagtgac 1

tgacttggagattgac 1

tgacttgggctttgac 1

tgacttggtgagtgac 1

tgacttgtagtatgac 1

tgacttgtcgattgac 1

tgacttgtttcttgac 1

tgactttaaatatgac 1

tgactttaattttgac 1

tgactttacacatgac 1

tgactttaccgttgac 1

tgactttcgacctgac 1

tgactttgaatttgac 1

tgactttgactatgac 1

tgactttgattatgac 1

tgactttggtattgac 1

tgacttttagtttgac 1

tgacttttctcttgac 1

tgacttttgaagtgac 1

tgacttttgagatgac 1

tgacttttgccgtgac 1

tgacttttgtcgtgac 1

tgactttttaagtgac 1

9 : 338

tgacaaaaaaaaatgac 1

tgacaaaaacaaatgac 1

tgacaaaaatacttgac 1

tgacaaaaatctctgac 1

tgacaaaaatggatgac 1

tgacaaaaattcatgac 1

tgacaaaaattcgtgac 1

tgacaaaaccatatgac 1

tgacaaaagtgtttgac 1

tgacaaaatacgatgac 1

tgacaaaatttgatgac 1

tgacaaacacagttgac 1

tgacaaagatcaatgac 2

tgacaaagatggttgac 1

tgacaaagcattctgac 1

tgacaaataaagatgac 1

tgacaaatattattgac 1

tgacaaatcttaatgac 1

tgacaaatgtatctgac 1

tgacaaattatgttgac 1

tgacaaattttagtgac 1

tgacaacaatttgtgac 1

tgacaacattacctgac 1

tgacaacggcatgtgac 1

tgacaacgttaattgac 1

tgacaacttgacatgac 1

tgacaacttggtctgac 1

tgacaagaaaagatgac 1

tgacaagaacttatgac 1

tgacaagaatgtttgac 1

tgacaagagaatttgac 1

tgacaagagcgtgtgac 1

tgacaagcaaatgtgac 1

tgacaagcctcagtgac 1

tgacaaggattattgac 1

tgacaaggctctatgac 1

tgacaagtcctagtgac 1

tgacaagtgtgtttgac 1

tgacaatacgttttgac 2

tgacaatagggattgac 1

tgacaatgactattgac 1

tgacaatgtttgttgac 1

tgacaattcattatgac 1

tgacaattgtttgtgac 1

tgacacaatgtattgac 1

tgacacacacttctgac 1

tgacacagagacatgac 1

tgacacagtccaatgac 1

tgacacatactcgtgac 1

tgacaccatcatctgac 1

tgacaccatggggtgac 1

tgacacctgtcgctgac 1

tgacacctttagttgac 1

tgacacgtctcactgac 1

tgacacgtggcagtgac 1

tgacacgtggtaatgac 1

tgacacgttggcatgac 1

tgacactaataattgac 1

tgacactgatggttgac 1

tgacacttacctgtgac 1

tgacacttccaaatgac 1

tgacacttgcagatgac 1

tgacagaaaaaaatgac 1

tgacagaaatctttgac 1

tgacagaaccaggtgac 1

tgacagaactaactgac 1

tgacagaagcatatgac 1

tgacagagagtcttgac 1

tgacagaggatggtgac 1

tgacagattggaatgac 1

tgacagctcattttgac 1

tgacaggattatatgac 1

tgacagggctagctgac 1

tgacagtggcgcgtgac 1

tgacagttttacctgac 1

tgacagttttgactgac 1

tgacataaaacattgac 1

tgacataattcaatgac 1

tgacataattttatgac 1

tgacatacatgaatgac 1

tgacatagatcgatgac 1

tgacatagtccattgac 1

tgacatatactgatgac 1

tgacatatataactgac 1

tgacatatctacttgac 1

tgacatatgattttgac 2

tgacatatgcatgtgac 1

tgacatatgctaatgac 1

tgacatatttacctgac 1

tgacatattttgttgac 1

tgacatctagcaatgac 1

tgacatcttttgatgac 1

tgacatgaacttttgac 1

tgacatgacattctgac 2

tgacatggaataatgac 1

tgacatggaatgatgac 2

tgacatgtaacactgac 1

tgacatgtcttgatgac 1

tgacatgttaagttgac 1

tgacatgttggtatgac 2

tgacattacataatgac 2

tgacattactagttgac 1

tgacattagatattgac 1

tgacattagttattgac 1

tgacattattatttgac 1

tgacattatttcctgac 1

tgacattcgattttgac 1

tgacattgattaatgac 1

tgacattgttcattgac 1

tgacattgttttctgac 1

tgacatttaatattgac 1

tgacatttatgattgac 1

tgacatttgatcatgac 1

tgacatttgttgttgac 1

tgacattttccattgac 1

tgacattttcttttgac 1

tgaccaaaaaaattgac 1

tgaccaaaagttttgac 1

tgaccaaacgttttgac 1

tgaccaaactatttgac 1

tgaccaaagaatttgac 1

tgaccaaagagactgac 1

tgaccaaagtgaatgac 1

tgaccaaataacttgac 1

tgaccaacatttatgac 1

tgaccaacgaggctgac 1

tgaccaagacaaatgac 1

tgaccaagcaatctgac 1

tgaccaagcacaatgac 1

tgaccaaggatgatgac 1

tgaccaagtattatgac 1

tgaccaatataattgac 1

tgaccaccttgtttgac 1

tgaccagctggtctgac 1

tgaccagtgtcattgac 1

tgaccatagtctttgac 1

tgaccatcattcttgac 1

tgaccatgaattttgac 1

tgaccatgacaaatgac 1

tgaccattaacaatgac 1

tgaccattgattttgac 1

tgaccattgctaatgac 1

tgaccattttctatgac 1

tgaccattttctttgac 1

tgaccattttggttgac 1

tgacccaatcccctgac 1

tgaccccaaaaaatgac 1

tgacccgagcgattgac 1

tgacccgattccttgac 1

tgaccctagtgtatgac 1

tgaccctctgtgttgac 1

tgacccttttctatgac 1

tgaccgaacaatgtgac 1

tgaccgaacactttgac 1

tgaccgaagaaattgac 1

tgaccgcgaactctgac 1

tgaccgctttcactgac 1

tgaccggtgaacatgac 1

tgaccggtgaacctgac 1

tgaccggtgattttgac 1

tgaccgtaataattgac 1

tgaccgtagtgggtgac 1

tgaccgtcgatgatgac 1

tgaccgtcttatgtgac 1

tgacctaattatttgac 1

tgacctataacaatgac 1

tgacctatattaatgac 1

tgacctatattgttgac 1

tgacctatctccgtgac 1

tgacctccacacttgac 1

tgacctcgtgttttgac 1

tgacctgaaccactgac 1

tgacctgaatggttgac 1

tgacctgagtaattgac 1

tgaccttcctcaatgac 1

tgacctttgaagctgac 1

tgaccttttggtctgac 1

tgacgaactaaaatgac 1

tgacgaagaagactgac 1

tgacgaagaattttgac 1

tgacgaagatccgtgac 1

tgacgacacattttgac 1

tgacgacatacattgac 1

tgacgacatgtcctgac 1

tgacgacgacgagtgac 1

tgacgacggagcttgac 1

tgacgagagtatttgac 1

tgacgaggagcagtgac 1

tgacgaggatcgatgac 1

tgacgagtagtagtgac 1

tgacgataaaagctgac 1

tgacgatacgttttgac 1

tgacgatagccactgac 1

tgacgatgcgtgatgac 1

tgacgattgagattgac 1

tgacgatttttactgac 1

tgacgatttttgttgac 1

tgacgcacacacgtgac 1

tgacgcatgatggtgac 1

tgacgccaagtagtgac 1

tgacgccgttacttgac 2

tgacgccttttgatgac 1

tgacgcgttttgttgac 1

tgacgcttcttcatgac 1

tgacgcttgtagttgac 1

tgacggcaaagattgac 1

tgacggcaagacgtgac 1

tgacggcggagcctgac 1

tgacgggttcctctgac 1

tgacggtgcgttttgac 1

tgacggtggacggtgac 1

tgacggtggttgctgac 1

tgacggtttgcgttgac 1

tgacgtaagagattgac 1

tgacgtatatgattgac 1

tgacgtatctttctgac 1

tgacgtcatgacgtgac 1

tgacgtccccacttgac 1

tgacgtgaacacgtgac 1

tgacgtgattgcatgac 1

tgacgtggattgatgac 1

tgacgtggcccattgac 1

tgacgtgtaaatgtgac 1

tgacgtgtaatcttgac 1

tgacgtgtgaatgtgac 1

tgacgttaaacgctgac 1

tgacgttcaatattgac 1

tgacgtttccttgtgac 1

tgacgtttgtgcttgac 1

tgacgttttagagtgac 1

tgacgttttattatgac 1

tgacgttttgttgtgac 1

tgactaaaattattgac 1

tgactaaacactttgac 1

tgactaaatgatgtgac 1

tgactaacattaatgac 1

tgactaacttcgttgac 1

tgactaagccgtttgac 1

tgactaattaaagtgac 1

tgactacaaatcttgac 1

tgactaccttaaatgac 1

tgactacgaacgatgac 1

tgactacgtaacttgac 1

tgactagatcaagtgac 1

tgactagatcttctgac 1

tgactagcttctttgac 1

tgactaggattgttgac 1

tgactaggcctaatgac 1

tgactaggtcaaatgac 1

tgactagtgaaattgac 1

tgactagttggcgtgac 1

tgactagtttttctgac 1

tgactatacaatgtgac 1

tgactatagcttttgac 1

tgactatataaactgac 1

tgactatcaaaattgac 1

tgactatcccaaatgac 1

tgactatgaatgttgac 1

tgactatgacaaatgac 1

tgactatgattgatgac 1

tgactattaactttgac 1

tgactattcaacttgac 1

tgactattggggttgac 1

tgactatttcttttgac 1

tgactatttttattgac 1

tgactcaaaattttgac 1

tgactcaaaggtttgac 1

tgactcaacaaagtgac 1

tgactcagccacttgac 1

tgactcaggaacctgac 1

tgactcatcatgctgac 1

tgactcatgtttgtgac 1

tgactccactctgtgac 1

tgactccatattttgac 1

tgactccccatcatgac 1

tgactcgaccttatgac 1

tgactcgtaacactgac 1

tgactcgttagcatgac 1

tgactctgaataatgac 1

tgactctgctagatgac 1

tgactctgctttctgac 1

tgactcttgtctctgac 1

tgactgaagatgctgac 1

tgactgacgaatatgac 1

tgactgactcgagtgac 1

tgactgaggataatgac 1

tgactgctttaggtgac 1

tgactggaactagtgac 1

tgactggaatatgtgac 1

tgactggaatgcatgac 1

tgactgtgatgagtgac 1

tgacttaaacttatgac 1

tgacttaaccacttgac 1

tgacttaagactttgac 1

tgacttaatgacttgac 1

tgacttagctctgtgac 1

tgacttatagttttgac 1

tgacttatgctattgac 1

tgacttcaaaatttgac 1

tgacttcaaattatgac 1

tgacttcaacatatgac 1

tgacttcactctttgac 1

tgacttcattgtctgac 1

tgacttcgatcgatgac 1

tgacttcgtgaaatgac 1

tgacttctcctcctgac 1

tgacttctctccgtgac 1

tgacttgaacaaatgac 1

tgacttgacaggttgac 1

tgacttggagtgatgac 1

tgacttgtcgctttgac 1

tgacttgttcttttgac 1

tgactttacatagtgac 1

tgactttaggagatgac 1

tgactttatctcatgac 1

tgactttcaacattgac 1

tgactttcacctttgac 1

tgactttcattgatgac 1

tgactttcgtgtctgac 1

tgactttgaaacctgac 1

tgactttgaccagtgac 1

tgactttgacttttgac 1

tgactttgatgtatgac 1

tgactttgcacagtgac 1

tgactttgtttgatgac 1

tgacttttagtcatgac 1

tgacttttatttttgac 1

tgacttttcttggtgac 1

tgacttttgtcgttgac 1

tgactttttctattgac 1

10 : 359

tgacaaaaaccaaatgac 1

tgacaaaaagtacatgac 1

tgacaaaaataaattgac 1

tgacaaaagtcgcatgac 1

tgacaaaatatatttgac 1

tgacaaaatcttattgac 1

tgacaaaattaatgtgac 1

tgacaaaattgatatgac 1

tgacaaacacatggtgac 1

tgacaaacataatttgac 1

tgacaaacatcagttgac 1

tgacaaaccatccgtgac 1

tgacaaacttgtagtgac 1

tgacaaagaatgaatgac 1

tgacaaagactatgtgac 1

tgacaaattatcaatgac 1

tgacaaattgacaatgac 1

tgacaaattttagatgac 1

tgacaaattttctttgac 1

tgacaacaaaacagtgac 1

tgacaacaacaacatgac 1

tgacaacaacccaatgac 1

tgacaacataattttgac 1

tgacaacatttctatgac 1

tgacaacgacatattgac 2

tgacaacgtcgtgttgac 1

tgacaagaagacagtgac 1

tgacaagagaatgttgac 1

tgacaagagataagtgac 1

tgacaagatttgcatgac 1

tgacaagcgacgagtgac 1

tgacaagtcatatatgac 1

tgacaagtgaaagttgac 1

tgacaagttgacaatgac 1

tgacaagtttttgttgac 1

tgacaatattgtcttgac 1

tgacaatgatgatgtgac 1

tgacaatgattcggtgac 1

tgacaatgtgacaatgac 1

tgacaatgtgagagtgac 1

tgacaattctttcatgac 1

tgacaattgacaaatgac 1

tgacaatttagagttgac 1

tgacaatttctaattgac 1

tgacaatttctgtgtgac 1

tgacaatttggatatgac 1

tgacacaaaaataatgac 2

tgacacaaaactcttgac 1

tgacacaacaaacttgac 2

tgacacaaggggtttgac 1

tgacacaatttccctgac 1

tgacacactccttttgac 1

tgacacacttcgtttgac 1

tgacacagcacctgtgac 1

tgacacatatagtttgac 1

tgacacattacatgtgac 1

tgacacattatttctgac 1

tgacaccaacctaatgac 1

tgacaccaagattctgac 1

tgacaccctgaagatgac 1

tgacacgtgtacggtgac 1

tgacacgtgtatattgac 1

tgacacgtgtctgatgac 1

tgacacgttggagctgac 1

tgacactaaattattgac 1

tgacactaataaattgac 1

tgacactacattcttgac 1

tgacactagcattgtgac 1

tgacactggggtcatgac 1

tgacacttcgtcgatgac 1

tgacagaaagactgtgac 1

tgacagacacagactgac 1

tgacagacgatgcatgac 1

tgacagactgacagtgac 1

tgacagcaaaaacttgac 1

tgacagctctgcggtgac 1

tgacagctgaacaatgac 1

tgacaggaggtaaatgac 2

tgacaggtgggtagtgac 1

tgacagtaacaatctgac 1

tgacagtaactgactgac 1

tgacagtcacatggtgac 1

tgacagtgataaactgac 1

tgacagttaaaccttgac 1

tgacagttttgagttgac 1

tgacataaaaccactgac 1

tgacataaaagacatgac 1

tgacataaaccttttgac 1

tgacataaattgagtgac 1

tgacataacatatttgac 1

tgacataactcttatgac 1

tgacataactttcctgac 1

tgacataagaaacatgac 1

tgacataagtaaaatgac 1

tgacataagttaaatgac 1

tgacataatcggaatgac 1

tgacatactaaagatgac 2

tgacatatatatcgtgac 1

tgacatatcaggtgtgac 1

tgacatatcatacatgac 1

tgacatatgacaaatgac 1

tgacatattatggatgac 1

tgacatattcatcgtgac 1

tgacatattgacattgac 1

tgacatcaatagtttgac 1

tgacatcatatgcatgac 1

tgacatcatctcactgac 1

tgacatcgtcattttgac 1

tgacatctttggattgac 1

tgacatgacaaaagtgac 1

tgacatgactttgatgac 1

tgacatgagatctttgac 1

tgacatgatgatgatgac 1

tgacatgcttgagttgac 1

tgacatggattttatgac 1

tgacatgtattgattgac 1

tgacattaacacattgac 2

tgacattaagaagttgac 1

tgacattagaacagtgac 1

tgacattccatacttgac 1

tgacattctctctttgac 1

tgacattgacacaatgac 1

tgacattgctaatttgac 1

tgacattgttattttgac 2

tgacatttcaaagctgac 1

tgaccaaaaacatttgac 1

tgaccaaaagaccatgac 1

tgaccaaaagccattgac 1

tgaccaaaagccgttgac 1

tgaccaaaatcctttgac 1

tgaccaaaatgccctgac 1

tgaccaaaccaatttgac 1

tgaccaaatcggtatgac 1

tgaccaacattgcttgac 1

tgaccaaccaagcttgac 1

tgaccaactccacatgac 1

tgaccaagaaaacatgac 1

tgaccaagagatattgac 1

tgaccaaggaaccatgac 1

tgaccaattgttggtgac 1

tgaccacaattatttgac 1

tgaccacattattatgac 1

tgaccactgaccagtgac 1

tgaccacttcgatttgac 1

tgaccagaaaaagctgac 1

tgaccagagttgattgac 1

tgaccagcacataatgac 1

tgaccataagtttgtgac 1

tgaccatatcattatgac 1

tgaccatcgaccattgac 1

tgaccatgaccatttgac 1

tgaccattgtctattgac 1

tgaccatttggacttgac 2

tgaccattttctgttgac 1

tgacccaataaatatgac 1

tgacccaattatcttgac 1

tgacccaccacacgtgac 1

tgacccataaattttgac 1

tgacccatgaattttgac 10

tgacccatgcatgatgac 1

tgacccatgtaaaatgac 1

tgacccgatcctcgtgac 1

tgacccgtgggtattgac 1

tgaccctgcaattgtgac 1

tgacccttccacgatgac 1

tgaccctttttccgtgac 1

tgaccgaaatttactgac 1

tgaccgaatcaatttgac 1

tgaccgattaatgctgac 1

tgaccgctgatttttgac 1

tgaccggagtgacctgac 1

tgaccggtggcttttgac 1

tgaccgttgatttatgac 1

tgacctaattggcctgac 1

tgacctagcaaatatgac 1

tgacctcttctacttgac 1

tgacctggcagtgttgac 1

tgacctgggattgttgac 1

tgaccttaaaaaattgac 1

tgaccttcgggtcctgac 1

tgacctttgttgcctgac 1

tgacctttgttttttgac 1

tgacctttttattttgac 1

tgacgaacaagctgtgac 1

tgacgaagtgatcatgac 1

tgacgacaattcagtgac 1

tgacgacgaaagtttgac 1

tgacgactatgacttgac 1

tgacgagcgacgagtgac 4

tgacgagtgaatgatgac 1

tgacgatataaagatgac 1

tgacgatattttagtgac 1

tgacgatcctaaattgac 1

tgacgattctttcttgac 1

tgacgcaaaatgcgtgac 1

tgacgcataccagctgac 1

tgacgccacgtctatgac 1

tgacgcgtgacgactgac 1

tgacgctagaatgatgac 1

tgacggaaattctgtgac 2

tgacggaaattggatgac 1

tgacggatccgaattgac 1

tgacggatgattaatgac 1

tgacggcgttacggtgac 1

tgacggctgatggttgac 1

tgacgggacgagtttgac 2

tgacggtgacggagtgac 1

tgacggtgcattattgac 1

tgacggtggctctgtgac 1

tgacgtaaaaagcgtgac 1

tgacgtaacgatgatgac 1

tgacgtaccaaacatgac 1

tgacgtagaggaaatgac 1

tgacgtatagatgatgac 1

tgacgtcactgctgtgac 1

tgacgtcatccatttgac 1

tgacgtcatgaatatgac 1

tgacgtccgcctgatgac 1

tgacgtcttcttcttgac 1

tgacgtggaacatgtgac 1

tgacgtggcacttatgac 1

tgacgtggtgactatgac 1

tgacgtgtgagtcgtgac 1

tgacgtgtgtcctttgac 1

tgacgttaacaacatgac 1

tgacgttatacaattgac 1

tgacgttccaactctgac 1

tgacgttgaccacttgac 1

tgacgtttatgttttgac 1

tgacgttttgtttttgac 1

tgactaaaccatcatgac 1

tgactaaccaagcatgac 1

tgactaaccatcagtgac 1

tgactaagggtatgtgac 1

tgactaagttgctttgac 1

tgactaatgactattgac 1

tgactaatgatatgtgac 1

tgactaatgtgaagtgac 1

tgactaattgacattgac 1

tgactacaattctatgac 1

tgactacatgcatatgac 1

tgactactatagtatgac 1

tgactacttgagcgtgac 1

tgactagagacagttgac 1

tgactagcatttcctgac 1

tgactagtgacgattgac 1

tgactagtgacttatgac 1

tgactagtggttgctgac 1

tgactataaaatattgac 1

tgactataagtttatgac 1

tgactatatgtgcctgac 1

tgactatattaaactgac 1

tgactatgattgcctgac 1

tgactattactgactgac 1

tgactattagtttttgac 1

tgactattgagtattgac 1

tgactattgattggtgac 1

tgactattttgaattgac 1

tgactcaaaacatatgac 1

tgactcacattgcttgac 1

tgactcactgatgatgac 1

tgactcatgatatttgac 1

tgactcattcatgatgac 1

tgactcgcataatatgac 1

tgactctccatagttgac 1

tgactctcttttattgac 1

tgactctgactatatgac 1

tgactctgagattgtgac 1

tgactctgtgactctgac 1

tgactcttcacctctgac 1

tgactcttctcccttgac 1

tgactgaaattttttgac 1

tgactgactgactgtgac 1

tgactgatggaagttgac 1

tgactgattggttttgac 1

tgactgatttgtgatgac 1

tgactgcacgaggatgac 1

tgactgcattctagtgac 1

tgactgctgaatcctgac 1

tgactggtgactggtgac 1

tgactggtgttcgttgac 1

tgactggttctatttgac 1

tgactggttgaatttgac 1

tgactgtatgagtgtgac 1

tgactgtgcatgggtgac 1

tgactgttacttgatgac 1

tgacttaaaaaccatgac 1

tgacttaacgcaaatgac 1

tgacttaagagttttgac 1

tgacttaagccaaatgac 1

tgacttaagtaatctgac 1

tgacttaatggtgctgac 1

tgacttagagtgattgac 1

tgacttagtcatgttgac 1

tgacttataatctttgac 1

tgacttatgacttatgac 1

tgacttattagtgttgac 1

tgacttattgttattgac 2

tgacttatttgagttgac 1

tgacttcaatgacttgac 1

tgacttcccggggttgac 1

tgacttccggattatgac 1

tgacttcgagtgggtgac 1

tgacttctaatgtatgac 1

tgacttctcaaaaatgac 1

tgacttgaaaacaatgac 1

tgacttgataactctgac 1

tgacttgataagattgac 1

tgacttgcgagttatgac 1

tgacttgtgaatgctgac 1

tgacttgtgagttctgac 1

tgacttgtgagttgtgac 2

tgacttgtttttattgac 1

tgactttaaagatttgac 1

tgactttaatgtgatgac 1

tgactttacgatggtgac 1

tgactttcattaaatgac 1

tgactttctgtgattgac 1

tgactttcttattttgac 1

tgactttctttgattgac 1

tgactttgaaaccgtgac 1

tgactttgagtctttgac 1

tgactttgtacccttgac 1

tgactttgttattgtgac 1

tgacttttaaactttgac 1

tgacttttagcttttgac 1

tgacttttatcatttgac 1

tgacttttcaatcctgac 1

tgacttttctttagtgac 1

tgacttttgacatatgac 1

tgacttttgagttttgac 1

tgacttttgatgaatgac 1

tgacttttggagtttgac 1

tgactttttgtgtttgac 1

tgacttttttagtatgac 1

11 : 374

tgacaaaaaagaaaatgac 1

tgacaaaaaatgtcttgac 1

tgacaaaaagagctctgac 1

tgacaaaactggacctgac 1

tgacaaaagaaacggtgac 1

tgacaaaagcaccggtgac 1

tgacaaaatgaccgatgac 1

tgacaaagcttttgttgac 1

tgacaaaggataacatgac 1

tgacaaagtgacgagtgac 1

tgacaaagtttttgttgac 3

tgacaaatcagtatttgac 1

tgacaaatgggtggatgac 1

tgacaaattatagtgtgac 1

tgacaaattggatattgac 1

tgacaacgaagatgatgac 1

tgacaactatgctcatgac 1

tgacaactggttcggtgac 1

tgacaagaaaagttttgac 1

tgacaagagttattatgac 2

tgacaagcacactcttgac 1

tgacaagcatgggggtgac 1

tgacaaggtttgtgatgac 1

tgacaagtgttggactgac 1

tgacaataattatattgac 1

tgacaatagaaaagatgac 1

tgacaatataaagcatgac 1

tgacaatatccaaaatgac 1

tgacaatattaccaatgac 1

tgacaatattacccctgac 2

tgacaatattaggcatgac 1

tgacaatgactcatttgac 1

tgacaatggaaaccctgac 1

tgacaatgtataaggtgac 1

tgacaatgtccaaattgac 1

tgacaatgtcttggctgac 2

tgacaatgttaatcatgac 1

tgacaatttcaatgttgac 1

tgacacaaaattaattgac 1

tgacacaagttagtatgac 1

tgacacacgaaataatgac 1

tgacacagaagtacttgac 1

tgacaccaatggatctgac 2

tgacaccttaaacaatgac 1

tgacacgcaagtaggtgac 1

tgacacgtggtttggtgac 1

tgacacgttggtcaatgac 1

tgacactataaaaaatgac 1

tgacactcaataacatgac 1

tgacactcgttttcgtgac 1

tgacactgccctaattgac 1

tgacactgtcattcatgac 1

tgacacttaaaaggttgac 1

tgacagacgcatcgatgac 1

tgacagagacactcatgac 1

tgacagagacggggttgac 1

tgacagataatcttgtgac 1

tgacagcaaccactatgac 1

tgacagccattcaggtgac 1

tgacagccctcgagctgac 1

tgacaggtgagttggtgac 1

tgacagtaatgttagtgac 1

tgacagtaattattttgac 2

tgacataaaaagtattgac 1

tgacataaatacccttgac 1

tgacataagctaaattgac 1

tgacatacagtttcttgac 1

tgacatactacggtatgac 1

tgacatagtaagatatgac 1

tgacatagttttcgttgac 1

tgacatatataactgtgac 1

tgacatatgatactttgac 1

tgacatcaatctgtttgac 1

tgacatcacaaaacttgac 1

tgacatcatacaatatgac 1

tgacatcattgatgatgac 2

tgacatccaagtttatgac 1

tgacatctatgggtttgac 1

tgacatctgtgatcttgac 1

tgacatctttttttctgac 1

tgacatgaacatatatgac 1

tgacatgaagaggtttgac 1

tgacatgaataatagtgac 1

tgacatgccataccctgac 1

tgacattaaaagtattgac 1

tgacattaatacttttgac 1

tgacattaatccgattgac 1

tgacattaattaaactgac 1

tgacattaattgacttgac 1

tgacattaggatttttgac 1

tgacattattgggattgac 1

tgacattcaaggatttgac 1

tgacattccaacatgtgac 1

tgacattcttttagttgac 1

tgacattgacaaaaatgac 1

tgacattgcactaactgac 1

tgacattgcgagtattgac 1

tgacattgtgaccactgac 1

tgacatttaacaacgtgac 1

tgacatttaccttgttgac 1

tgacatttgactatatgac 1

tgacatttgagtttttgac 1

tgacatttgtaaaagtgac 1

tgacatttgttttattgac 1

tgacattttttcttctgac 1

tgaccaaaaaagatttgac 1

tgaccaaaatgaacgtgac 1

tgaccaaaattccattgac 1

tgaccaaaatttatgtgac 1

tgaccaaccatatattgac 1

tgaccaactcactattgac 1

tgaccaagcccaaagtgac 1

tgaccaataaaaaattgac 1

tgaccaataaaaatgtgac 1

tgaccaataccagtgtgac 1

tgaccaatcagttcatgac 1

tgaccaatcatattttgac 1

tgaccaatgacttgatgac 1

tgaccaattaaaacttgac 1

tgaccacaaagaagttgac 1

tgaccacgacacttctgac 2

tgaccactcagtagttgac 2

tgaccacttcaagaatgac 1

tgaccagtgaggcagtgac 1

tgaccataaaaggtttgac 1

tgaccataacgattatgac 1

tgaccataatgtgactgac 1

tgaccatataccatatgac 1

tgaccatatattgcttgac 1

tgaccatcatcttaatgac 1

tgaccatgaaaagactgac 1

tgaccatgaggccaatgac 1

tgaccatgtcagtgatgac 1

tgaccatgttcgatctgac 1

tgaccattaatgaaatgac 1

tgaccattacaaccatgac 1

tgaccattacgctcatgac 1

tgaccattcataatttgac 1

tgaccattgaaaaattgac 1

tgaccattgatatattgac 1

tgacccaaaacaaaatgac 1

tgacccaagggcttctgac 1

tgacccaagtgtatatgac 1

tgacccacaaacacgtgac 1

tgacccactcaaaattgac 1

tgacccactctttaatgac 1

tgacccatgaaatgttgac 1

tgacccatgagtccatgac 1

tgaccccaacgatgttgac 1

tgaccccatttaaattgac 1

tgacccgcaaaagtgtgac 1

tgacccgtctcgtgctgac 1

tgaccctactgtttatgac 1

tgaccctagcatttgtgac 1

tgacccttctgttgttgac 1

tgaccgatgaatgaatgac 1

tgaccggtgaatttctgac 1

tgaccgtccgatgaatgac 1

tgaccgttagagatttgac 1

tgacctaatctttgatgac 1

tgacctaatgatgcttgac 1

tgacctagcaggtcgtgac 1

tgacctataccttattgac 1

tgacctatctgcacttgac 1

tgacctatgagtctatgac 1

tgacctatttattactgac 1

tgacctcaaaatttttgac 1

tgacctcatatctcctgac 1

tgacctccaaaaccgtgac 1

tgacctcgatcgtgatgac 1

tgacctctatagttttgac 1

tgacctctatttgtttgac 1

tgacctgactacccttgac 1

tgacctgagcaatgttgac 1

tgacctgcacttctttgac 1

tgacctgccaattcctgac 1

tgacctgttgagtgttgac 1

tgaccttaggctggttgac 1

tgaccttcgaaccgctgac 1

tgaccttcgggcctttgac 1

tgaccttcttccaggtgac 1

tgaccttgctggacgtgac 1

tgaccttgttcgtcttgac 1

tgacctttaaatccttgac 1

tgacctttacggtggtgac 1

tgacctttataagtttgac 1

tgacctttataattctgac 1

tgacctttatgtatttgac 1

tgacgaagtttctgttgac 1

tgacgacaataattttgac 1

tgacgacacatgatgtgac 1

tgacgacagatccattgac 1

tgacgacatttaagctgac 1

tgacgacgaaaataatgac 1

tgacgacgcattaattgac 1

tgacgacgtctgatctgac 1

tgacgactatacccttgac 1

tgacgacttactatttgac 2

tgacgagagatgctttgac 1

tgacgagcaaagtcttgac 1

tgacgagcgaactcttgac 1

tgacgaggctttcattgac 1

tgacgataacggagatgac 1

tgacgatgaagatgatgac 1

tgacgatgacgtgagtgac 1

tgacgatgatgattctgac 1

tgacgatgctgaaagtgac 3

tgacgatggatgagatgac 1

tgacgattttgcccctgac 1

tgacgcaaaagagagtgac 1

tgacgcaatctcgtttgac 1

tgacgccaccaatgatgac 1

tgacgcccctcaaattgac 1

tgacgcgatccatattgac 1

tgacgcttaatcttctgac 1

tgacgcttcgtcttctgac 1

tgacggacacgtaaatgac 1

tgacggagaagatggtgac 1

tgacggatcttttcttgac 1

tgacggattcaaatttgac 2

tgacggcaaccctagtgac 1

tgacggcggcgaaagtgac 1

tgacggtaaaatctgtgac 1

tgacggtgaggatgatgac 1

tgacggtggctgtaatgac 1

tgacggttattagattgac 1

tgacgtaaagaaagatgac 1

tgacgtagacgaacgtgac 1

tgacgtagatctctatgac 1

tgacgtagtacccgatgac 1

tgacgtcatatctcttgac 1

tgacgtcatccatcatgac 1

tgacgtcgaagaagctgac 1

tgacgtcggcggcggtgac 1

tgacgtcgttatatatgac 1

tgacgtgcaaacaattgac 1

tgacgtggaagtgagtgac 1

tgacgtggaggataatgac 1

tgacgtggatatatttgac 1

tgacgtgtcaaactgtgac 2

tgacgtgttaagtcgtgac 1

tgacgttaagtattttgac 1

tgacgttaattaatctgac 1

tgacgttttacactctgac 1

tgacgttttttcttttgac 1

tgactaaaaatatgttgac 1

tgactaaaaattgtatgac 1

tgactaaaagattattgac 1

tgactaaaataagtttgac 1

tgactaaacttggggtgac 1

tgactaacgagctcttgac 1

tgactaactacttattgac 1

tgactaagcgcactttgac 1

tgactaataatgtattgac 1

tgactaatatggttgtgac 1

tgactaatgagaatttgac 1

tgactaatgggccagtgac 1

tgactaattcgaatttgac 1

tgactacagctgcattgac 1

tgactagagagaacatgac 1

tgactagagtagtagtgac 1

tgactagatattcattgac 1

tgactagatgtattttgac 1

tgactagtcacgtcatgac 1

tgactagtcttcttctgac 1

tgactatactggtaatgac 3

tgactatataatgtttgac 1

tgactatattaaccttgac 1

tgactatcctcaaattgac 1

tgactatctggaggctgac 1

tgactatgcagccaatgac 1

tgactattatttttgtgac 1

tgactatttccttgttgac 1

tgactcaaacagtgttgac 1

tgactcaacctccattgac 1

tgactcaatcaccattgac 1

tgactcaatgactcatgac 1

tgactcagctgccgttgac 1

tgactcatgggaacctgac 1

tgactccaaagacagtgac 1

tgactccaagttcaatgac 1

tgactccaatacatttgac 1

tgactcccatgcacttgac 1

tgactcgctgtcttgtgac 1

tgactcggaaccctgtgac 1

tgactcggtaaaagttgac 1

tgactcgtgactcagtgac 1

tgactcgttctagtttgac 1

tgactctataatctgtgac 1

tgactctgggaaatgtgac 1

tgactcttggggaggtgac 1

tgactgacgtacgagtgac 1

tgactgactctcggctgac 1

tgactgatgggaagatgac 1

tgactgatttacctttgac 1

tgactgccaagtggctgac 1

tgactgctataaaattgac 1

tgactgctgagaaaatgac 1

tgactggcaagagtctgac 1

tgactgggtcactgatgac 1

tgactgtaaaagctttgac 1

tgactgtggtggtgatgac 1

tgactgttattctgttgac 1

tgacttaaaagtttttgac 1

tgacttaaactaaattgac 1

tgacttaccagaccatgac 1

tgacttagcttaatgtgac 1

tgacttagcttccggtgac 1

tgacttatagtcatgtgac 1

tgacttatcaatatgtgac 1

tgacttatggttgcgtgac 1

tgacttcaagcaggttgac 1

tgacttcacctccggtgac 1

tgacttcagaaaatttgac 1

tgacttccacgagtctgac 1

tgacttcctcagttttgac 1

tgacttctccttgtgtgac 1

tgacttgaaacatcatgac 1

tgacttgaaacgtcatgac 2

tgacttgaaacgtcgtgac 1

tgacttgaaatgtcatgac 1

tgacttgaaggaggatgac 1

tgacttgagtcctagtgac 1

tgacttgatatttgttgac 1

tgacttgctcgattctgac 1

tgacttgtaacaatgtgac 1

tgacttgtacatatgtgac 1

tgacttgtgaggtcgtgac 1

tgacttgttcttacgtgac 1

tgacttgttggtcgatgac 1

tgactttaaaactagtgac 1

tgactttaaatctcatgac 1

tgactttaatttgcgtgac 1

tgactttgaaagctttgac 1

tgactttgacgcgtttgac 1

tgactttgagcgtcatgac 1

tgactttgagtcaaatgac 1

tgactttgatcacgttgac 1

tgactttgatctttatgac 1

tgactttgtaagcaatgac 1

tgactttgtctttgttgac 1

tgactttgtgaattctgac 1

tgacttttaaatggatgac 2

tgacttttaacatattgac 1

tgacttttacacacttgac 1

tgacttttacagggttgac 1

tgacttttaggtgcatgac 1

tgacttttatgagtctgac 1

tgacttttccgaaagtgac 1

tgacttttgagtttctgac 1

tgacttttgcacccttgac 1

tgacttttgtacgtgtgac 1

tgactttttaattattgac 1

tgactttttgacttttgac 1

tgactttttttattttgac 1

12 : 317

tgacaaaaaaaaataatgac 1

tgacaaaaaacgtatatgac 1

tgacaaaaacaatttctgac 1

tgacaaaaatgttttttgac 1

tgacaaaactgagccatgac 1

tgacaaaagaaaagattgac 1

tgacaaaagcttataatgac 1

tgacaaacatatattttgac 1

tgacaaaccgggtcgttgac 1

tgacaaactaatttggtgac 1

tgacaaactacacagttgac 1

tgacaaacttgtccgttgac 1

tgacaaagactcgagttgac 1

tgacaaagtgcaagattgac 1

tgacaaataagattgatgac 1

tgacaaatctctctcttgac 1

tgacaaattaaaaagttgac 1

tgacaacaccagtgactgac 1

tgacaacagctttttctgac 1

tgacaacattaactcctgac 1

tgacaaccatttttagtgac 1

tgacaactaaaacttatgac 1

tgacaactaaaccagttgac 1

tgacaacttatgactgtgac 1

tgacaacttcaaaagttgac 1

tgacaagaaattatcttgac 1

tgacaagcggctgtaatgac 1

tgacaaggaggtcagttgac 1

tgacaataaaataatatgac 1

tgacaataaatcacaatgac 1

tgacaatatttgaaagtgac 1

tgacaatctgacgtactgac 2

tgacaattatactgcatgac 1

tgacaattccagctaatgac 1

tgacaatttaaccacttgac 1

tgacaattttaccaactgac 1

tgacaattttttaaaatgac 1

tgacaattttttggtatgac 1

tgacacaaacactaactgac 1

tgacacaaattttgtgtgac 1

tgacacaattaaatcttgac 1

tgacacatacacattttgac 1

tgacacatatcaacattgac 1

tgacaccatatatttttgac 1

tgacacccaaaactagtgac 1

tgacaccgtaggatgttgac 1

tgacaccgttaaaatttgac 1

tgacaccgttttctgttgac 1

tgacacgaggcaatactgac 1

tgacacggttacaaactgac 1

tgacacgtccggccgttgac 1

tgacacgtggacatgctgac 1

tgacacgtgtcactcatgac 1

tgacactacatctttgtgac 1

tgacacttgattttggtgac 1

tgacactttcttgttctgac 1

tgacagacgtcagacgtgac 1

tgacagagactttatatgac 1

tgacagagatcttcagtgac 1

tgacagagtctcgggctgac 1

tgacagatagtcaaaatgac 1

tgacagcataaaggattgac 1

tgacagcatataattctgac 1

tgacaggatgtgcatgtgac 1

tgacaggcaaagacattgac 1

tgacagggacacaacctgac 1

tgacaggtccaattcctgac 1

tgacaggtgacggatttgac 1

tgacagtaagagtctttgac 1

tgacagtaatatcttttgac 1

tgacagtcatgattcatgac 1

tgacagtcgtcactggtgac 1

tgacagtgatgagtagtgac 1

tgacagttaatgtgaatgac 1

tgacataaattgatattgac 1

tgacataacacatgcttgac 1

tgacataagtagcttttgac 1

tgacataagttaaaattgac 1

tgacataatcaacatttgac 1

tgacatagattcaatctgac 1

tgacatcatttgagtttgac 1

tgacatcgttttggtttgac 1

tgacatctacagcagttgac 1

tgacatctcaatattttgac 1

tgacatgaaagaaaattgac 1

tgacatggatagatattgac 1

tgacatggtcaaaatttgac 1

tgacatggtgatacagtgac 1

tgacatgtcttgttcttgac 1

tgacatgttgtttgtttgac 1

tgacatgttttgactttgac 1

tgacattaactttggctgac 1

tgacattgaattttaatgac 1

tgacattgagagtctttgac 1

tgacattgatggcttttgac 1

tgacattgattcctagtgac 1

tgacatttaaatgtagtgac 1

tgacatttagtttttgtgac 1

tgacatttataatacatgac 1

tgacatttatattagttgac 1

tgacatttgatttaggtgac 1

tgacatttgtttctggtgac 1

tgacatttgttttgtttgac 1

tgacattttaaacaaatgac 1

tgacattttatctttttgac 1

tgacattttcaatttctgac 1

tgacattttcagtttttgac 1

tgacattttgaagatatgac 1

tgacatttttttaatgtgac 1

tgacattttttttagctgac 1

tgaccaaaaacgagcatgac 1

tgaccaaagttttggttgac 1

tgaccaaatcgacatttgac 1

tgaccaaattaatgtttgac 1

tgaccaaccttgaagatgac 1

tgaccaagccaaagagtgac 1

tgaccaagcccaaaagtgac 1

tgaccaagtccaagagtgac 1

tgaccaagtgtctgcatgac 1

tgaccaagttcaagagtgac 1

tgaccaatacaaaaaatgac 1

tgaccaatatcttggatgac 1

tgaccaatgtatgtggtgac 3

tgaccaattgtgttagtgac 1

tgaccacagacttcagtgac 1

tgaccacattttatagtgac 1

tgaccactaaagatattgac 1

tgaccacttgaaaacatgac 1

tgaccactttagattttgac 1

tgaccagactctctaatgac 1

tgaccagctcaatctctgac 1

tgaccagtgaagaaaatgac 1

tgaccagttgtccatttgac 1

tgaccataggtaaacctgac 1

tgaccatagttttatgtgac 1

tgaccatattttaagctgac 1

tgaccatccaggccgctgac 1

tgaccattccttagcttgac 1

tgaccattctctaaagtgac 1

tgaccattgaaccaagtgac 1

tgaccattgaatgttttgac 1

tgaccattgctgacattgac 1

tgaccattttgttttatgac 1

tgacccaaatataatttgac 1

tgacccactggtgagttgac 1

tgaccccaaataaaggtgac 1

tgacccgatgacccagtgac 1

tgacccgatgacccggtgac 1

tgacccggtgacccggtgac 1

tgaccctaagcaaaaatgac 1

tgaccgaggatacatatgac 1

tgaccgagtagctacttgac 1

tgaccgagtcttttattgac 1

tgaccgatcgaccggctgac 1

tgaccggacaagccggtgac 1

tgaccgggacacaatctgac 1

tgaccgggtacgtagatgac 1

tgaccgtcgctatcggtgac 1

tgaccgtgcggcgaattgac 1

tgaccgtgtttgctgctgac 1

tgacctaacacattaatgac 1

tgacctaatacttaggtgac 1

tgacctaattagagactgac 1

tgacctattcgaggattgac 1

tgacctctatcttctctgac 1

tgacctgactttatcttgac 1

tgacctgtaccaacactgac 1

tgacctgtattttaagtgac 1

tgaccttacactcatgtgac 1

tgaccttacatgttcatgac 1

tgaccttactagtatctgac 1

tgaccttagctttagatgac 1

tgaccttctaatgtcttgac 1

tgaccttgaagccacttgac 1

tgaccttgtcaacgtctgac 1

tgaccttttacatttctgac 1

tgacgaaacatttatttgac 1

tgacgaaacttacaagtgac 1

tgacgacaatataggttgac 1

tgacgacccaaaaatttgac 1

tgacgagacaaaattctgac 1

tgacgatagtctatagtgac 1

tgacgcacaaaaaaattgac 1

tgacgcacgtgagccatgac 1

tgacgctgagaagctgtgac 1

tgacggcgctggtggttgac 1

tgacgggaacgtcacatgac 1

tgacgggatatttttatgac 1

tgacgggtcaccaaattgac 1

tgacgggttagttgcttgac 1

tgacggtaaacccctttgac 1

tgacggtatgtcaatttgac 1

tgacggtccacaagcctgac 1

tgacggtgaactacgttgac 1

tgacggtgatggtctctgac 1

tgacggttaaaatttttgac 1

tgacgtaataatccattgac 1

tgacgtacataaaatttgac 1

tgacgtacgaagaatatgac 1

tgacgtagaaagaaggtgac 1

tgacgtagtttggctttgac 1

tgacgtatcgaattagtgac 1

tgacgtattcttctcatgac 1

tgacgtcaccaaagtttgac 2

tgacgtcaggtgagcctgac 1

tgacgtcctgcactattgac 1

tgacgtcggttcttattgac 1

tgacgtcgtcaagctttgac 1

tgacgtggcaatactatgac 1

tgacgtggtgactagttgac 1

tgacgtgtccatatgttgac 1

tgacgtgtgaatgtgatgac 1

tgacgtgtggctgttctgac 2

tgacgttaagaaaacgtgac 1

tgacgttaataatcaatgac 1

tgacgttagtttcacctgac 1

tgacgttatcgactaatgac 1

tgacgttgaatgattttgac 1

tgacgtttccatttgttgac 1

tgactaaaaatcaagatgac 1

tgactaaaatcgatggtgac 1

tgactaaaccggaccatgac 1

tgactaacagcttgattgac 1

tgactaacccagactatgac 1

tgactaactccgccattgac 1

tgactaaggaccacattgac 1

tgactaagtatagttctgac 1

tgactaatatttaaattgac 1

tgactaattagtagtttgac 1

tgactacactgatcattgac 1

tgactactaatataattgac 1

tgactacttctacctctgac 1

tgactagagatcaaattgac 1

tgactagggccacaagtgac 1

tgactaggggagatgatgac 1

tgactagtatgtgaggtgac 1

tgactatcaatcacaatgac 1

tgactatcatttgatttgac 1

tgactatctatctttttgac 1

tgactatgaagcaagttgac 1

tgactatgaatgtgggtgac 1

tgactatgtggttccatgac 1

tgactattagataaaatgac 1

tgactattcatatgattgac 1

tgactcaacttgtatttgac 1

tgactcacagtgagtgtgac 1

tgactcatcttcttcatgac 1

tgactcatgaatctagtgac 1

tgactccaaaaatttgtgac 1

tgactccaatgagtgctgac 1

tgactccatttagcaatgac 1

tgactccatttctttttgac 1

tgactccgcttataattgac 1

tgactcctcaagtggttgac 1

tgactcgaaatctaattgac 1

tgactcgattacccagtgac 1

tgactcgggatcttcttgac 2

tgactcgtatttgatttgac 1

tgactcgtcgtaattttgac 1

tgactcgtctcgctcgtgac 1

tgactcgtgaaacttttgac 1

tgactcgttgagtccttgac 1

tgactctaatacacattgac 1

tgactctgccttcagatgac 1

tgactctgtccccagatgac 1

tgactgaacaaattattgac 1

tgactgaatcaccacatgac 1

tgactgaattttgaaatgac 1

tgactgatccgtggcttgac 1

tgactgatgagcgttttgac 1

tgactgcgtttgtcattgac 1

tgactgctttacaaaatgac 1

tgactggccaatttaatgac 1

tgactgggtaaaaagttgac 1

tgactgggttagatggtgac 1

tgactggtaaaaatattgac 1

tgactgtgtcggaaactgac 1

tgactgttagtatttttgac 1

tgacttaagatgtttttgac 1

tgacttaaggtagatgtgac 1

tgacttaattactagttgac 1

tgacttagagtattcatgac 2

tgacttagtcactctatgac 1

tgacttagtttgtcgttgac 1

tgacttatcattaacttgac 1

tgacttcacttactgttgac 1

tgacttctatggagattgac 1

tgacttgaaaggcagatgac 1

tgacttgcttcaatcttgac 1

tgacttgctttttcattgac 1

tgacttgtcagtagattgac 1

tgacttgttctgaaattgac 1

tgacttgtttaaattatgac 1

tgacttgtttattatgtgac 1

tgactttatttcttgttgac 1

tgactttcacgtctcctgac 1

tgactttcgaaataaatgac 4

tgactttcgttaccaatgac 1

tgactttctaaataaatgac 2

tgactttctatataaatgac 1

tgactttgaaagaaactgac 1

tgactttgcgttcagatgac 1

tgacttttcaatttgatgac 1

tgacttttgaaagatgtgac 1

tgactttttcaactgttgac 1

tgactttttctaattatgac 1

13 : 363

tgacaaaaaaaaaatgttgac 1

tgacaaaaaaaaagacatgac 1

tgacaaaaaactgaacatgac 1

tgacaaaaacgataccgtgac 1

tgacaaaaatgtaccattgac 1

tgacaaaacaggaactttgac 1

tgacaaaaccgggaaaatgac 1

tgacaaaacttttccggtgac 1

tgacaaacataaattattgac 1

tgacaaaccgatgaaaatgac 1

tgacaaacgtatgcgtttgac 1

tgacaaactaatggcattgac 1

tgacaaagtaaaagaggtgac 1

tgacaaatacgaactagtgac 1

tgacaaatatgcaattgtgac 1

tgacaaatatgttaatatgac 1

tgacaaatcatacttggtgac 1

tgacaaattaaccactttgac 1

tgacaacaattagatgatgac 1

tgacaacagatttgagttgac 1

tgacaacatatgtattatgac 1

tgacaacccataacgcatgac 2

tgacaaccttggtcctttgac 1

tgacaactacagtttgatgac 1

tgacaactacgaatccatgac 1

tgacaacttgacaacattgac 1

tgacaagaaaatcattgtgac 1

tgacaagaacatgaaaatgac 1

tgacaagaactaagcaatgac 1

tgacaagataacacgcgtgac 1

tgacaagcaaatgcatttgac 1

tgacaagctaaggattgtgac 1

tgacaagtaaagagtcgtgac 1

tgacaagtatattcttttgac 1

tgacaagtggaaatatgtgac 1

tgacaagttagcttctttgac 1

tgacaataacatctttctgac 1

tgacaataatgtgaacgtgac 1

tgacaatacgagctccttgac 1

tgacaatatgggtgtattgac 1

tgacaatgatgtcagcatgac 1

tgacaatgtagtatttgtgac 1

tgacaatgtttagtcagtgac 1

tgacaattttcttgtagtgac 1

tgacaatttttattatgtgac 1

tgacacatatatgtatgtgac 1

tgacacatattcattcatgac 1

tgacacatcaccataaatgac 1

tgacacatctatcctcgtgac 1

tgacacatgatacattttgac 1

tgacaccaaaactatagtgac 1

tgacacccaaaccaacatgac 2

tgacaccgagtctcacgtgac 1

tgacacctaaatgattatgac 1

tgacacctgcgcaggattgac 1

tgacacgccttacgatgtgac 1

tgacacggtcataagtttgac 1

tgacacgtcatgcggcatgac 1

tgacacgtctcttactatgac 1

tgacactagtttacatttgac 1

tgacactagttttcatttgac 1

tgacactatcgtgcatgtgac 1

tgacactattattaacttgac 1

tgacactctgattatagtgac 1

tgacacttaacacatgttgac 1

tgacacttataagaatttgac 1

tgacacttcaacttgaatgac 1

tgacagaccgcgattgctgac 1

tgacagacttccacaattgac 1

tgacagatatgagtttgtgac 1

tgacagattcactttcttgac 1

tgacagccacgcgcttttgac 1

tgacagcccaagctcgatgac 1

tgacagcttatatatactgac 2

tgacagctttgatcgattgac 1

tgacagggagtggatgttgac 1

tgacagtagatttcatgtgac 1

tgacagtgaagaacacgtgac 1

tgacagtgtcggttctctgac 1

tgacagtgttgcgttattgac 1

tgacataaactaagatttgac 1

tgacatactacctaatttgac 1

tgacatagacttgtctttgac 1

tgacatatgtatatttatgac 1

tgacatatgttttttaatgac 1

tgacatattcgagaaagtgac 1

tgacatcaacatctagctgac 1

tgacatcaagttccaaatgac 1

tgacatcatacatgtgatgac 1

tgacatcatcgataagttgac 1

tgacatcattaactttgtgac 1

tgacatcgcagacgaaatgac 1

tgacatctagtcatatttgac 1

tgacatctctattactttgac 1

tgacatgaacaattatatgac 1

tgacatgatatatggtttgac 2

tgacatgccgttgttgatgac 1

tgacatgctgctgctggtgac 1

tgacatgctgttaaatctgac 1

tgacatggctgctggtctgac 1

tgacatgtcaagggaagtgac 1

tgacatgttgtaataattgac 1

tgacattaagttttggatgac 1

tgacattaatacttttttgac 1

tgacattacgatgataatgac 1

tgacattagtttgttgttgac 1

tgacattcaaaaagaagtgac 1

tgacattcgtatttttttgac 1

tgacattctttattcggtgac 1

tgacattgaattggtcatgac 1

tgacattgttgtttttctgac 1

tgacatttgatctatagtgac 1

tgaccaaaaggaatatgtgac 1

tgaccaaacgtatctgttgac 1

tgaccaaactattttgttgac 1

tgaccaaagactaacggtgac 1

tgaccaaagctgtagcttgac 1

tgaccaaagttaacatttgac 1

tgaccaaatctgcaaattgac 1

tgaccaaatgttctacttgac 1

tgaccaaattttccggatgac 1

tgaccaaccaatatttttgac 1

tgaccaactttcttttgtgac 1

tgaccaagacactagtctgac 1

tgaccaagagagagcattgac 1

tgaccaagtactcgttttgac 1

tgaccaagtatggaacgtgac 1

tgaccaatagttttatgtgac 1

tgaccaatataataatctgac 1

tgaccaatggtagcacgtgac 1

tgaccaattaaaccgagtgac 1

tgaccacaaatattgtttgac 1

tgaccacaacctgttcgtgac 1

tgaccacccttcttcaatgac 1

tgaccacgaccaggccatgac 1

tgaccactacttagcgttgac 1

tgaccactccaccttggtgac 1

tgaccactctaacaagttgac 1

tgaccagctcctgtgtttgac 1

tgaccagggaaattttttgac 1

tgaccataaatagctaatgac 1

tgaccatacatgcatggtgac 1

tgaccatatcttctatatgac 1

tgaccatcaaaataaggtgac 1

tgaccatgaattacatctgac 1

tgaccatgtggaccacgtgac 1

tgaccattaattacaattgac 1

tgaccattcttatcaactgac 1

tgaccattgactcttgttgac 1

tgaccattgagccacaatgac 1

tgacccaactcacattttgac 1

tgacccaagaacacccttgac 1

tgacccaataactcatttgac 4

tgacccaataactcgtttgac 1

tgacccatagctttgcttgac 1

tgacccatatgtatacctgac 1

tgacccatcaactcatttgac 1

tgacccctgtgcaacgttgac 1

tgaccccttcccctgattgac 1

tgacccgaaaacccgaatgac 1

tgaccctaccttcactttgac 2

tgacccttattacgtactgac 1

tgaccctttcgccttattgac 2

tgaccgagatcttccggtgac 1

tgaccgattaattcgactgac 1

tgaccgcggttactaagtgac 1

tgaccggactaactcgatgac 1

tgaccggtaaaggagtttgac 1

tgaccggtcatgtcgtttgac 1

tgaccggtctactcgattgac 1

tgaccgtacaccagctgtgac 4

tgaccgtgccgacattttgac 1

tgaccgtttgcactacgtgac 1

tgacctagacctaatcttgac 1

tgacctatgtgagactgtgac 1

tgacctcagtttcattttgac 1

tgacctcttatttattatgac 1

tgacctcttcgcctagttgac 1

tgacctgatccacaagttgac 1

tgacctgcctcctccattgac 1

tgacctggacgcacagttgac 1

tgaccttattcttatcatgac 1

tgaccttcaagcggggctgac 1

tgaccttgagagaagggtgac 1

tgaccttgccatcttggtgac 1

tgacgaaaaagtaaatatgac 1

tgacgaaaagtcttcattgac 1

tgacgaaaatacccttttgac 1

tgacgaaactagccctttgac 1

tgacgaatgacagaagatgac 1

tgacgaccgagactctgtgac 1

tgacgaccttgtggagatgac 1

tgacgagaaatagtccatgac 1

tgacgagcctaccctcttgac 3

tgacgaggaagcggtcatgac 1

tgacgatctgttctcgatgac 1

tgacgatgacgtggaagtgac 1

tgacgatggtaagattctgac 1

tgacgatttgcacaaaatgac 1

tgacgattttcagaatatgac 1

tgacgcaagcgtgcgtttgac 1

tgacgcattgtgataattgac 1

tgacgcccgttgcaatatgac 1

tgacgcccgttggattatgac 1

tgacgccggcgagaagatgac 2

tgacgcttagagctagttgac 1

tgacggaaccacaatcatgac 1

tgacggacaccaaccaatgac 1

tgacggactcttaacgatgac 1

tgacggatcgaagaacctgac 1

tgacggatttgatttggtgac 1

tgacggcatgtcgagactgac 1

tgacggtgtgttgaaagtgac 1

tgacggtttggccatcatgac 1

tgacgtaagtaaaatagtgac 1

tgacgtatccacgaccttgac 1

tgacgtcaccaagaaaatgac 1

tgacgtcagcagcggcgtgac 2

tgacgtcgaatttaaattgac 1

tgacgtctatatgcttatgac 1

tgacgtctcccagaatatgac 1

tgacgtgaattcgaagttgac 1

tgacgtgagattacgattgac 1

tgacgtggcgctttcgttgac 1

tgacgtggtccagccaatgac 1

tgacgtgtcacgtcaattgac 1

tgacgttaaaagccgagtgac 1

tgacgttatcctcccgttgac 1

tgacgtttgtgtttttgtgac 1

tgacgttttattcaaagtgac 1

tgacgtttttttacccctgac 1

tgactaaaaatccgggttgac 1

tgactaaaagatatgtatgac 1

tgactaaaattattatctgac 2

tgactaaagttaacaaatgac 1

tgactaacaaaagatcgtgac 1

tgactaaccattggatgtgac 1

tgactaacccaaaatgatgac 1

tgactaagaattaaaaatgac 1

tgactaaggcgattggttgac 1

tgactaatcaaagaagatgac 1

tgactaatgaatcataatgac 1

tgactaatttgttgtgctgac 1

tgactacatctatcacttgac 1

tgactacattacccttttgac 1

tgactagaatgatttagtgac 1

tgactagagtctagatatgac 1

tgactagatgtccaccttgac 1

tgactagcagctgccaatgac 1

tgactagctttataatttgac 1

tgactagtaaatagatttgac 1

tgactagtagtgacagctgac 1

tgactagtcaactgtgttgac 1

tgactagtcgtctagcctgac 2

tgactagtgtgggtctctgac 1

tgactagttttctttgatgac 1

tgactatacaaatatagtgac 1

tgactatatatgaataatgac 1

tgactatattatcaagatgac 1

tgactatattcaagttgtgac 1

tgactatgttgcgtaattgac 1

tgactattattgcatgttgac 2

tgactattgataacaactgac 1

tgactcactttccaagatgac 1

tgactcagctttgaatgtgac 1

tgactcagtattaaagctgac 1

tgactcatcaaaccaattgac 2

tgactcatcaacccatttgac 1

tgactcatcaactcatttgac 7

tgactcatcaacttatttgac 1

tgactcatgagtcatgatgac 1

tgactcatgcaaatgaatgac 1

tgactcattgcttgacttgac 1

tgactccacacgtcaagtgac 1

tgactccacgtatggcctgac 1

tgactccttcttttctatgac 1

tgactccttgttttaaatgac 1

tgactcgcccatactcatgac 1

tgactcgcccatgcgcttgac 1

tgactcggctacattggtgac 1

tgactcggttgcttatctgac 1

tgactcgtcttgttgcctgac 1

tgactcgttcatatatttgac 1

tgactctactacccctatgac 2

tgactctgagaaggagctgac 1

tgactctgtgtaaaatgtgac 1

tgactctttgatctacatgac 1

tgactcttttcaacttctgac 1

tgactggaataagcatctgac 1

tgactggatctcttaggtgac 1

tgactgtataaaaccaatgac 1

tgactgtctttagtctctgac 1

tgactgttccaatctcttgac 1

tgacttaagagagaatatgac 1

tgacttacttcgtgctttgac 1

tgacttaggattattattgac 1

tgacttataacaaccattgac 1

tgacttataatgccacgtgac 1

tgacttatcaatatgtttgac 1

tgacttatcaatgcttgtgac 1

tgacttatccataatgttgac 1

tgacttatctcagccactgac 1

tgacttatgtataaaaatgac 1

tgacttcaaactagaagtgac 1

tgacttcaatgatccagtgac 1

tgacttcactattgatttgac 1

tgacttcatgcgactattgac 1

tgacttcattttgtgtgtgac 1

tgacttccacatatatttgac 1

tgacttcgggcatgatgtgac 1

tgacttcgttttgctggtgac 1

tgacttctatttaaaactgac 1

tgacttctttccgattttgac 1

tgacttgaaacttaaactgac 1

tgacttgaacaaagaaatgac 1

tgacttgactcattgcttgac 1

tgacttgggataacacatgac 1

tgacttgtaaaaatatttgac 1

tgacttgtgaggtgaaatgac 1

tgacttgttaatggcattgac 1

tgacttgttatctttgctgac 1

tgacttgttcctccccttgac 1

tgacttgtttaccgaattgac 1

tgactttaattaattaatgac 1

tgactttacatgagctatgac 1

tgactttattttttgtttgac 1

tgactttgacaagttcttgac 1

tgactttggtcaaagtttgac 1

tgacttttagggacttgtgac 1

tgacttttatctcattttgac 1

tgacttttcctttgctttgac 1

tgacttttggattcttgtgac 1

tgactttttatccggattgac 2

tgactttttcgtggaaatgac 1

tgactttttttcttcaatgac 1

14 : 386

tgacaaaaaaaaaacctatgac 1

tgacaaaaaaaaaacttttgac 1

tgacaaaaaatataagtgtgac 1

tgacaaaaaatataataatgac 1

tgacaaaaactccaaatatgac 1

tgacaaaacatggttagttgac 1

tgacaaaagaaattaaactgac 1

tgacaaacattaaatatatgac 1

tgacaaacctaggtaatatgac 1

tgacaaacctaggtagtatgac 1

tgacaaactgaagaacactgac 1

tgacaaagaaaactttattgac 1

tgacaaagatgccattgttgac 1

tgacaaagatttctcttttgac 1

tgacaaaggactcaactttgac 1

tgacaaagtatttgttattgac 1

tgacaaagttgtctttggtgac 1

tgacaaagtttacaaagttgac 1

tgacaaataatcagcaagtgac 1

tgacaaatccatgtgatgtgac 1

tgacaaattacaacataatgac 1

tgacaaattagaaaaacatgac 1

tgacaaattgttggtcagtgac 1

tgacaacaacatgtccgttgac 1

tgacaacatgtcaacatgtgac 1

tgacaacgcatgtctatgtgac 1

tgacaactaacgtatgggtgac 1

tgacaactatcggatcgttgac 1

tgacaactgggatagaagtgac 1

tgacaacttgatggataatgac 1

tgacaacttgtaaaattatgac 1

tgacaagaagcttgttgatgac 1

tgacaagactagactgcatgac 1

tgacaagagcaccaagtgtgac 1

tgacaagatgttaactaatgac 1

tgacaagcatatattagatgac 1

tgacaaggaacaaaaaaatgac 1

tgacaagttaacaaaacttgac 1

tgacaataacacaatcactgac 1

tgacaataacatatccgttgac 1

tgacaataacgaagaaggtgac 1

tgacaatagcaaaaaatttgac 1

tgacaatatgatttcagatgac 1

tgacaatatttggatatttgac 1

tgacaatcatgattttcctgac 1

tgacaatctgacattctttgac 1

tgacaatctttgttcctttgac 1

tgacaatgaaatatgggttgac 1

tgacaatgacgatggccttgac 1

tgacaatggaagctccggtgac 1

tgacaatgtttcttttgttgac 1

tgacaattaaccacatattgac 1

tgacaattactcaattagtgac 1

tgacaattctgagtggcttgac 1

tgacacaaagtactcaaatgac 1

tgacacaattaacaagaatgac 1

tgacacaccaaaagcagttgac 1

tgacacacctaatcccgctgac 1

tgacacagccactgacattgac 1

tgacacatgtgactagtttgac 1

tgacacattggatgattatgac 1

tgacaccatatatacttttgac 1

tgacaccttcttaaagtctgac 1

tgacacgcaacagaagtttgac 1

tgacacgtggaacgtagatgac 1

tgacacgtgtattgcacgtgac 1

tgacacgttggcaaatcatgac 1

tgacactagggtttattctgac 1

tgacactatactaagaaatgac 1

tgacactgctcgtgctggtgac 1

tgacactgtcgcaacgattgac 1

tgacacttaccgtgatgttgac 1

tgacagaaaatgggacggtgac 1

tgacagaaccaggtgacctgac 1

tgacagaagatatgatggtgac 1

tgacagacgcaaaaacattgac 1

tgacagaggatgaagagttgac 1

tgacagccaagtactacatgac 1

tgacagccaataagattatgac 1

tgacagcctcctcgaaaatgac 1

tgacaggatcagtgaggctgac 1

tgacaggattatatgacatgac 1

tgacaggtacgccttccatgac 1

tgacaggtatgcccggtatgac 1

tgacaggtatgcctggtatgac 1

tgacagtagcaaactacatgac 1

tgacagtcctgctagtggtgac 1

tgacagtgaataggattatgac 1

tgacagtgatattaccattgac 1

tgacagtgatgatagcagtgac 2

tgacagtgttttgagttttgac 1

tgacataacacagcccattgac 1

tgacataacctagagttgtgac 1

tgacataacggagaattttgac 1

tgacataagcccaatggttgac 1

tgacataaggcaagaatctgac 1

tgacataattatgcatcatgac 1

tgacatacaaaaaagttttgac 1

tgacatacccaaaattcttgac 1

tgacatatcattatagtatgac 1

tgacatatttcacaattatgac 1

tgacatcaaaaaacaaattgac 1

tgacatcaaagaattttgtgac 1

tgacatcacctcaaaagatgac 1

tgacatcatataattaggtgac 1

tgacatcatcaattggcttgac 1

tgacatcgttcccattgctgac 1

tgacatctgtcacaattatgac 1

tgacatctgtcaccattatgac 1

tgacatcttcggcaatgttgac 1

tgacatctttcttagtcatgac 1

tgacatgcatgggagaattgac 1

tgacatgcctacacacattgac 1

tgacatgggcacagttagtgac 1

tgacatgtatgcgaaatgtgac 1

tgacatgttatacttcagtgac 1

tgacattaatgatggtattgac 1

tgacattagaatataaaatgac 1

tgacattcaaacaaatgttgac 1

tgacattgatatactctttgac 1

tgacattgatatgtttgttgac 1

tgacattgtacatgcccttgac 1

tgacattgtttattacagtgac 1

tgacatttacataatttatgac 1

tgacatttatataagttttgac 1

tgacatttattgagagtgtgac 2

tgacatttctcttgacaatgac 1

tgaccaaaaaaacataaatgac 1

tgaccaaaaaatattcaatgac 1

tgaccaaaaagtataacatgac 1

tgaccaaaaagttgaaaatgac 1

tgaccaaaaattgggtattgac 1

tgaccaaactaaatacattgac 1

tgaccaaatccaaccaattgac 1

tgaccaaccaaaagaaattgac 2

tgaccaactcttataatgtgac 1

tgaccaagatataacttgtgac 1

tgaccaataacttgtttatgac 1

tgaccaatattcaccgcctgac 1

tgaccaatgtgatgatgatgac 1

tgaccaatttaaaacatatgac 1

tgaccaatttatataaaatgac 1

tgaccacaatttaaaacttgac 3

tgaccacaccgaattgcttgac 1

tgaccacgtatttgaaattgac 1

tgaccagactgatcatgatgac 1

tgaccagatgtatctcgttgac 1

tgaccagctccttttcattgac 1

tgaccatcacggaagcgatgac 1

tgaccatcatattcatactgac 1

tgaccatgatatggacaatgac 1

tgaccatggtgatcgttttgac 1

tgaccattatagtgtgtgtgac 1

tgaccattgtttcaattttgac 1

tgaccatttttctgttaatgac 1

tgacccagcgtctggttttgac 1

tgacccattcacacacactgac 1

tgacccctacccgctccttgac 1

tgacccgtattttatacttgac 1

tgaccctaagcgttggaatgac 1

tgaccctattgttgagtatgac 1

tgaccctgcgatcgaatgtgac 1

tgacccttattagatccatgac 1

tgacccttttggccattttgac 1

tgaccgaaacgatgtcgttgac 1

tgaccgcgggggaaatgctgac 1

tgaccggaggagccgccgtgac 1

tgaccggatggcccaaactgac 1

tgaccggtcgaaccggattgac 1

tgaccgtgaccatctttttgac 1

tgacctaaaagaatggtttgac 1

tgacctaactagcttgattgac 1

tgacctaactgatctgtgtgac 1

tgacctaatctgttcttatgac 1

tgacctacgtggttacggtgac 1

tgacctacttttgtcatatgac 1

tgacctatacaaaaataatgac 1

tgacctatgccaataagatgac 1

tgacctattgtgtatctatgac 1

tgacctcatgatttttattgac 1

tgacctccgctatgaaattgac 1

tgacctcttctacgcttttgac 1

tgacctgattagatagtgtgac 1

tgaccttcatatatgctttgac 1

tgaccttcgctgcaagtttgac 1

tgaccttgaagatgatgatgac 1

tgacctttggtaccgaagtgac 1

tgacctttgtcttattgctgac 1

tgaccttttgaccgcacgtgac 1

tgacgaaatcagattacgtgac 1

tgacgaacaggccacgcgtgac 1

tgacgaacttgaccactatgac 1

tgacgaagaataaatgtttgac 1

tgacgaagactcgaactttgac 1

tgacgaagggactagatatgac 1

tgacgacaaaaagtaaaatgac 1

tgacgacgaagtttccgttgac 1

tgacgacgacgattcttttgac 1

tgacgacggttaactatatgac 1

tgacgactaagtcaagattgac 1

tgacgactcattttttaatgac 1

tgacgagctctataaacgtgac 1

tgacgagtgatccatttatgac 1

tgacgataaacgtagagttgac 1

tgacgatacaaaaccatatgac 1

tgacgatatgtcaatctttgac 1

tgacgatattttcacgtttgac 1

tgacgatgaagaagatgttgac 1

tgacgatggtcccatgcatgac 1

tgacgattttgtgacatatgac 1

tgacgcaaattttctcgctgac 1

tgacgcaacaagcattcatgac 1

tgacgcagcaagtgatggtgac 1

tgacgctagatctttcgttgac 1

tgacgcttagactaatattgac 1

tgacggacctcctccgtatgac 1

tgacggattaatattctctgac 1

tgacggcgaaggagatggtgac 1

tgacggcgagagagatgatgac 1

tgacggctatggtggtggtgac 1

tgacggctcaaaattgtgtgac 1

tgacgggacaaccctcgttgac 1

tgacgggagaacaactgttgac 1

tgacgggagctgtaacagtgac 1

tgacggtgaaggtgcctttgac 1

tgacgtaagcttagtcaatgac 1

tgacgtacaagtgataaatgac 1

tgacgtactaattgattgtgac 1

tgacgtataatacggtgttgac 1

tgacgtcatcagcgtcattgac 1

tgacgtcgtactggtacttgac 1

tgacgtctctctcttatgtgac 1

tgacgtctgtttattggttgac 1

tgacgtgagatttgtttgtgac 1

tgacgtgatcatgtgtcatgac 1

tgacgtggaagcgcgtagtgac 1

tgacgtggattatggtaatgac 3

tgacgtggcgtgttgccatgac 1

tgacgtggtaacaagtgatgac 1

tgacgtgtgcgagatatttgac 1

tgacgttaaagtcaataatgac 2

tgacgttatggaatgaaatgac 1

tgacgttctagaactgattgac 1

tgacgttctcgcagagtatgac 1

tgacgttgattattattttgac 1

tgacgttggttaggtaattgac 1

tgacgttttgaaagtcaatgac 1

tgacgtttttgttctctttgac 2

tgactaaaagtagatgaatgac 1

tgactaaaatgcgttgagtgac 1

tgactaaatatggtaacatgac 1

tgactaaatcatggaacatgac 1

tgactaataagtaataaatgac 1

tgactaatataccaaatgtgac 1

tgactaatcacattataatgac 2

tgactaatgaatttcttttgac 1

tgactaatgagtcacatatgac 1

tgactaatgtcaaataagtgac 1

tgactaattaataagatctgac 1

tgactaattccacttccgtgac 1

tgactacaaaatctcgattgac 1

tgactacatacataatattgac 1

tgactacattaatccttttgac 1

tgactacatttattccattgac 1

tgactacgatcgagtcattgac 1

tgactacttgtttaatgatgac 1

tgactacttttctctatgtgac 1

tgactagagtgagagtgatgac 1

tgactataacaggttacttgac 1

tgactataccaatgcgattgac 1

tgactatatacatcgaactgac 1

tgactatatagagaacagtgac 1

tgactatatgatatgtattgac 2

tgactatatttcccaaattgac 2

tgactatcaacatcttattgac 1

tgactatgacgaggtctctgac 1

tgactatgactgtgaccgtgac 1

tgactatgagacgaaaaatgac 1

tgactatgtagtatcttttgac 1

tgactattccagcaacaatgac 1

tgactattgaaatatttttgac 1

tgactattgtatcggttatgac 1

tgactattgttgtgagtgtgac 1

tgactatttagacaattttgac 1

tgactattttaattagtttgac 1

tgactcaaaccctagatttgac 1

tgactcaaactttttgagtgac 1

tgactcaagattaatctttgac 1

tgactcacttggaaagtttgac 1

tgactcagcgcctcatgttgac 1

tgactcagtccttttgtttgac 1

tgactcagtgacgactcatgac 1

tgactcatgactaatccatgac 1

tgactcattgaaagggtttgac 1

tgactccatgcaccgagatgac 1

tgactcccgcttttactttgac 1

tgactcctcaggacgagctgac 1

tgactcgcccaccatccatgac 1

tgactcgtaaaagacttttgac 1

tgactcgtggttcaggtgtgac 1

tgactctctaatggtctgtgac 1

tgactctgaggaagctgttgac 1

tgactcttattgagtatttgac 1

tgactcttcaccaatatatgac 1

tgactcttgaatttttactgac 1

tgactcttggaaagatagtgac 1

tgactcttggagggttagtgac 1

tgactctttaatagcctttgac 1

tgactctttgaagaattctgac 1

tgactgaaaagatgatattgac 1

tgactgaaactccccacgtgac 1

tgactgaaggaattcttttgac 1

tgactgactaagggtatgtgac 1

tgactgactgactgactgtgac 1

tgactgcaacagcttctttgac 1

tgactgcaataactgaaatgac 1

tgactgcccaataagtcttgac 1

tgactgcctgactggtattgac 1

tgactgctccttcgagcatgac 1

tgactgggattggtgcagtgac 1

tgactgggccaaacctgttgac 1

tgactgtaaattggaggctgac 1

tgactgtcgacaactgaatgac 1

tgactgtcgatgcaattgtgac 1

tgactgtcttaagtagtttgac 1

tgactgtgaccgtgactgtgac 1

tgactgtggtggtgattttgac 1

tgactgtgtcatattttctgac 1

tgactgttgcgtcatctttgac 1

tgactgttggcacatagttgac 1

tgacttaacatgtacgcttgac 1

tgacttaagccaaatgactgac 1

tgacttaagttataatactgac 1

tgacttaatgctatttcgtgac 1

tgacttactagaaacagttgac 1

tgacttaggttccgagtttgac 1

tgacttatcaaaatcaaatgac 1

tgacttatcatctggtgctgac 1

tgacttattttttcctgttgac 1

tgacttattttttttaactgac 1

tgacttcaagtcctttgatgac 1

tgacttcactttttaatttgac 1

tgacttccgacatattggtgac 2

tgacttcgttttgatttttgac 1

tgacttctctcttgtcgttgac 1

tgacttgaaaccaaattttgac 2

tgacttgaacaagttacctgac 1

tgacttgaacattcactttgac 1

tgacttgaatacttgatttgac 1

tgacttgccactttggtatgac 1

tgacttgcgatcctgagttgac 1

tgacttggaagctaaggttgac 1

tgacttgtcctgtcaccatgac 1

tgacttgtgagttctgactgac 1

tgacttgtgagttgtgagtgac 1

tgactttaatttttaaaatgac 1

tgactttacctgtcactatgac 1

tgactttagggtttgttctgac 1

tgactttagtcaatacattgac 1

tgactttagttatagctctgac 1

tgactttatttacaacgatgac 2

tgactttcgacctgagtttgac 1

tgactttgagtttgatgttgac 1

tgactttgctaattttaatgac 1

tgactttgctatacttgttgac 1

tgactttggtatacatagtgac 1

tgactttgtttcattcattgac 1

tgacttttaagttctttctgac 1

tgacttttctcgctactctgac 1

tgacttttgatggtgatttgac 1

tgacttttttataaattgtgac 1

15 : 329

tgacaaaaaaaattgaatttgac 1

tgacaaaaataaaatattctgac 1

tgacaaaattttataaatttgac 1

tgacaaacaacatttgatgtgac 1

tgacaaactattactggcatgac 1

tgacaaagatcatgccgcatgac 1

tgacaaagctgctgagtgctgac 1

tgacaaagtattttttcgttgac 1

tgacaaattaggcgcttattgac 1

tgacaaatttctatgtctatgac 1

tgacaacaaaaaattcatttgac 1

tgacaacaagtaaatgctatgac 1

tgacaacagattctttatttgac 2

tgacaacatttaaccattttgac 1

tgacaactagcatttctggtgac 1

tgacaactgaggaacataatgac 1

tgacaactttatttccttttgac 1

tgacaactttccatcttgttgac 1

tgacaagaaaaataacccctgac 1

tgacaagaaagtcaatcaatgac 1

tgacaagaagacagtgacttgac 1

tgacaagagagatgattggtgac 1

tgacaagcgtggccggagatgac 1

tgacaagctcttttgaaagtgac 1

tgacaagtcaaggaacaaatgac 1

tgacaagtttcgtttttattgac 1

tgacaatacgggagctatttgac 1

tgacaatctttatcatcgttgac 1

tgacaatggtagagaataatgac 1

tgacaattgtaatttgttgtgac 1

tgacaattttggtaacatatgac 2

tgacaccatataactcagttgac 1

tgacaccctgtctctaatttgac 2

tgacaccggggctagcgcgtgac 1

tgacaccgttcgtgtcgtgtgac 1

tgacacgcaagaacttggatgac 1

tgacacgtgtcgtttgttgtgac 1

tgacacgtgttcccttatttgac 1

tgacactatacttgtttagtgac 1

tgacactatattacagcagtgac 1

tgacacttgggaatcaacctgac 1

tgacacttttgtctccatttgac 1

tgacagaaaatgttattgttgac 1

tgacagaagagattcaaagtgac 1

tgacagagaaatagagttgtgac 1

tgacagagggagctgagggtgac 1

tgacagatttggattattttgac 2

tgacagcagttttctttgttgac 1

tgacagccaaggcagctaatgac 1

tgacagcgttcgaattccatgac 1

tgacagctcaaattaaagttgac 1

tgacaggtgatgtgaccggtgac 1

tgacagtaaatggctatagtgac 1

tgacagtgaagatctcgagtgac 1

tgacagtttttcaactggttgac 1

tgacataacgaacctaaaatgac 1

tgacataatgctcttgatttgac 1

tgacatagcaaaaggtgtttgac 1

tgacatatatcttatcgtttgac 1

tgacatattattctctggatgac 1

tgacatcacttgtatttcttgac 1

tgacatcatgaatttctcatgac 1

tgacatccaccgataagattgac 1

tgacatccattgaaacatttgac 1

tgacatgaaactgtagcattgac 1

tgacatgacatgagatctttgac 1

tgacatgacatgtattgattgac 1

tgacatgagcaaaaataagtgac 1

tgacatgagttaagttgagtgac 1

tgacatgcaagagccactttgac 1

tgacatggaatctctatgttgac 1

tgacatggatatatgattctgac 1

tgacatgtcgaccagagtttgac 1

tgacatgtcgcgattgttatgac 1

tgacatgtttgtatgtatttgac 1

tgacattaaaaacttgagatgac 1

tgacattaaatgacagatttgac 1

tgacattacatcttatcgttgac 2

tgacattagatacaaaacttgac 1

tgacattataatatattaatgac 1

tgacattatcagtgactactgac 1

tgacattattattgtgttctgac 1

tgacattcaattatatagctgac 1

tgacattcatataaatatatgac 1

tgacattccaaagaaaatatgac 1

tgacattgaaataaaacattgac 1

tgacattgactttgacttttgac 1

tgacattgcactaactgactgac 1

tgacatttaaaaactcatttgac 1

tgacatttaagtttttatttgac 1

tgacatttcaaaagtcccatgac 1

tgacatttccaaattttagtgac 1

tgacatttgactttttaagtgac 1

tgacatttgagattggttttgac 1

tgacatttgatattttgtatgac 1

tgacatttgtgactcagcgtgac 1

tgacattttatagtttagttgac 1

tgacattttgttttggatctgac 1

tgacatttttaaaactttttgac 1

tgacatttttgttacgttttgac 1

tgaccaaacaaacacaaaatgac 1

tgaccaaagagagaattattgac 1

tgaccaaatcccgaaacggtgac 1

tgaccaacaaaagaaaatatgac 1

tgaccaactaaaatcattttgac 1

tgaccaagaaagatatatttgac 1

tgaccaagatgtccaatattgac 1

tgaccaagattagaagttatgac 1

tgaccaagtcaacgaaacttgac 2

tgaccaatgaccaatttattgac 1

tgaccacatgacgtcatcttgac 1

tgaccacatggtatggagttgac 1

tgaccactgcgatgcaatgtgac 1

tgaccaggcgttgagtgggtgac 1

tgaccatagaaagtacccgtgac 1

tgaccatcgtcaagaactgtgac 1

tgaccatctatgtacttcctgac 1

tgaccatgaaccgcgactatgac 1

tgaccatgaatctcttcaatgac 1

tgaccattacaaattaagatgac 1

tgaccattctttattaatgtgac 1

tgaccattgttgagtagagtgac 1

tgaccatttactaaaattctgac 1

tgaccatttactctgattttgac 1

tgaccatttttttgttacctgac 1

tgacccaaaaaaaactctttgac 1

tgacccgaatcgaaccgtttgac 1

tgacccgtaattgatccactgac 1

tgaccgaaaatgataaatttgac 1

tgaccgaagaaattgtttttgac 1

tgaccgagatgaccggagttgac 1

tgaccgagtaaagagcgaatgac 1

tgaccgattctgatatatgtgac 1

tgaccgatttcttaaacactgac 1

tgaccggacagatggattctgac 1

tgaccggtgaacatgacggtgac 1

tgaccgtaaacagagaaattgac 1

tgaccgtactctgaccgattgac 1

tgaccgtataaaatacatttgac 1

tgaccgtcattgtctaaattgac 1

tgacctagagcagcccaaatgac 1

tgacctagatgcgaatatttgac 1

tgacctagttgagcatatatgac 4

tgacctagttttatccagatgac 1

tgacctatcggatcctaaatgac 1

tgacctatgaatgatactttgac 1

tgacctcaaatccagagcttgac 1

tgacctcgtggcttcatattgac 1

tgacctcttctaatttcattgac 1

tgacctgtggaggggtccatgac 1

tgaccttacagattttatctgac 1

tgaccttgtgatattgatatgac 1

tgacctttgtgttggtccctgac 1

tgaccttttgagtcaaccatgac 1

tgacctttttagtttcttatgac 1

tgacgacattatcttaaactgac 1

tgacgaccaatattttttgtgac 1

tgacgaccagattagcaagtgac 1

tgacgataaataaattagttgac 1

tgacgataattaaaagtgttgac 1

tgacgatcagcattgcatgtgac 1

tgacgattacgatttctgttgac 2

tgacgattgatgatatttctgac 1

tgacgccagctgtacaacttgac 1

tgacgccgtgtgtcaatcatgac 1

tgacgcgatccttggcaggtgac 1

tgacgcgtaaaactcagtttgac 1

tgacgctgaacatctgctttgac 1

tgacggaagatgttgatgttgac 1

tgacggatattccaaagtttgac 1

tgacggatcaatccacgaatgac 1

tgacggcaaaacgttatcttgac 1

tgacgggaaacgttaactctgac 1

tgacgggcaagtggaggcatgac 1

tgacgggttatacttggtatgac 1

tgacggtaagtaccagtcctgac 2

tgacggtcgacatgtgggttgac 1

tgacgtaattatttaacaatgac 1

tgacgtagttaaggaacattgac 1

tgacgtattcccagaatgttgac 1

tgacgtatttgtccctttttgac 1

tgacgtcaaattatgcctttgac 1

tgacgtcatataacatatatgac 1

tgacgtcgttttgtaataatgac 1

tgacgtctacattattttatgac 1

tgacgtctccatgtttctctgac 1

tgacgtctttacacacgtctgac 1

tgacgtgagaagtcgtcattgac 1

tgacgtggattatgtgtaatgac 1

tgacgtggccgatgagagctgac 1

tgacgttataccacttgaatgac 1

tgacgttcgcttaattttttgac 1

tgacgttcttgctttatattgac 1

tgacgttgacatagtccattgac 1

tgacgttttgaatgataagtgac 1

tgacgttttttcgtgtagttgac 1

tgactaaagctggaaaatttgac 1

tgactaaataaatatagtatgac 1

tgactaaatcatctattgatgac 1

tgactaacacccgttactttgac 1

tgactaacctttttgaatttgac 1

tgactaacgcgttagagagtgac 1

tgactaagaaccctataattgac 1

tgactaagacaaatggtgttgac 1

tgactaagcgggtcccttttgac 1

tgactaagggtccgaatagtgac 1

tgactaatagtgtggggcttgac 1

tgactacaagaaccgactctgac 1

tgactacatgagaccgtcctgac 1

tgactacatgttctgtctttgac 1

tgactacgttgtcttgtaatgac 1

tgactactagatatttacttgac 1

tgactacttatataagcgttgac 1

tgactagagtcaacaaaaatgac 1

tgactagcaacgataacattgac 1

tgactataatattcaatgttgac 1

tgactatacttagtaattctgac 1

tgactatcattcttggttgtgac 1

tgactatccgttaataacgtgac 1

tgactatggaactatggcgtgac 1

tgactatggatggacaaattgac 1

tgactatgggttggcgtggtgac 1

tgactatgttttcctgagttgac 1

tgactattctagtactatatgac 1

tgactattgtatgtctgtatgac 1

tgactcaagttttcagctttgac 1

tgactcacaagtcacaagttgac 1

tgactcacacgtcattctttgac 1

tgactcactatacaagtattgac 1

tgactcagacccataattgtgac 1

tgactcagatcatagttactgac 1

tgactcagccttagcattttgac 1

tgactcatcaactcgcacgtgac 1

tgactccaatcaaaatctttgac 1

tgactcctccaccaagatctgac 1

tgactctaaagattgtcgttgac 1

tgactctaatcaaaatctttgac 1

tgactctacaacatgcagatgac 1

tgactctatatgattggagtgac 1

tgactctcctagttacttgtgac 1

tgactctcggttaatccaatgac 1

tgactctcttgtttcctcctgac 1

tgactctgacaatacgttttgac 2

tgactctttcaagaaccgttgac 1

tgactctttgacgtatctttgac 1

tgactctttgtttttagtgtgac 1

tgactcttttggtttgatatgac 1

tgactcttttttctctttttgac 1

tgactgaaaatttactatttgac 1

tgactgaaatgtcatcgagtgac 1

tgactgaacacaaccctattgac 1

tgactgaattaaagttagatgac 1

tgactgagattagttaaattgac 1

tgactgagtatcaccacagtgac 1

tgactgatgggagcgaaattgac 1

tgactgatgggtcgacttttgac 1

tgactgattagagccgtcatgac 1

tgactgattataattacattgac 1

tgactgattctcactttattgac 1

tgactgattttgtgagccttgac 1

tgactgcggctcttgctggtgac 1

tgactgctggcttattcagtgac 1

tgactggagatggagtgaatgac 1

tgactgggtctcaacgtggtgac 1

tgactggtgagataaatgatgac 1

tgactgtaaaaccctctattgac 1

tgactgtgtcttcttcgtttgac 1

tgactgttattaggaatgatgac 1

tgactgttttgactatgattgac 1

tgacttaaaaatgcaatgttgac 1

tgacttaaggcaaaagttttgac 1

tgacttaataagaagagtttgac 1

tgacttacttagcatttgttgac 1

tgacttagtcataattcagtgac 1

tgacttatagaatagatcttgac 1

tgacttatggaaggcaacgtgac 1

tgacttattctataaaacttgac 1

tgacttcaacatcaccatttgac 1

tgacttcaccacatccacttgac 1

tgacttccattagagctattgac 1

tgacttcgagttcttggagtgac 1

tgacttcgcaactgaaaagtgac 1

tgacttctaaaatggaccttgac 1

tgacttctccgacagaacttgac 1

tgacttctcggtcatatcttgac 1

tgacttgatgacccatacatgac 1

tgacttgggttgtaacttgtgac 1

tgacttgtaatatctgaaatgac 1

tgacttgtcatgaactctctgac 1

tgacttgtcgtcatggtcctgac 1

tgacttgtgttgtgccttgtgac 1

tgacttgttttagattctctgac 1

tgactttaagatgagttcttgac 1

tgactttacctcttcctaatgac 1

tgactttataatggatacatgac 1

tgactttctcaggcattgttgac 1

tgactttcttctcttcctttgac 2

tgactttgacctataacaatgac 1

tgactttgaggtagagatttgac 1

tgactttggcacacaaccgtgac 1

tgactttggttgaagtgtttgac 1

tgactttgtcagttagagttgac 2

tgactttgtgtttacacaatgac 1

tgactttgttatccacttttgac 2

tgacttttacatcgtgatctgac 1

tgacttttattttcaaatctgac 1

tgacttttcgagggccatttgac 1

tgacttttctttctttttttgac 1

tgacttttgttttctttgttgac 1

tgactttttaaaacgttaatgac 1

tgactttttaatatataattgac 1

tgactttttctattgactctgac 1

tgacttttttcttgaaccatgac 1

tgactttttttttttgggttgac 1

16 : 391

tgacaaaaaaaaacaagggttgac 1

tgacaaaaataaaaaaaggatgac 2

tgacaaaaccaaaaagaccttgac 1

tgacaaaacggcacgtagtttgac 1

tgacaaaacgtttgcgaaagtgac 1

tgacaaaagtaatcaatttgtgac 1

tgacaaaataaataaacaagtgac 1

tgacaaaatcgtgaagaatatgac 1

tgacaaacaaatttatttagtgac 1

tgacaaacgaagagggaaagtgac 1

tgacaaagaaaaaaagactttgac 1

tgacaaagaaaatgtattgttgac 1

tgacaaagagtgccaatgcttgac 1

tgacaaagatcaatgactcatgac 2

tgacaaagatgccagtttcctgac 1

tgacaaagtgacaagtatagtgac 1

tgacaaatatatttttgggctgac 1

tgacaaatctaatgaatctttgac 1

tgacaacaaaaaaactatgttgac 1

tgacaacaaatttagaaggttgac 1

tgacaacaagtcaagaaaaatgac 1

tgacaacattttagagtttttgac 1

tgacaactaattcttgattttgac 1

tgacaagaccggccatttagtgac 1

tgacaagagagtgagagtgctgac 1

tgacaagagcagaagaatcttgac 1

tgacaaggctagcaagaatttgac 1

tgacaagtgacgatttttactgac 1

tgacaagttaattaaagaaatgac 1

tgacaataaataatctgtattgac 1

tgacaataacgtatgaggtgtgac 1

tgacaatagtttaactgaattgac 1

tgacaatattgatcagaagttgac 1

tgacaatatttagacttttatgac 1

tgacaatcagctttatttgatgac 1

tgacaatctccagtttcagttgac 1

tgacaatgaaatggaagtggtgac 1

tgacaatggtggaacctttgtgac 1

tgacaattaacagttacgactgac 1

tgacaattactaacacacggtgac 1

tgacaattctttgtgatgtatgac 1

tgacaattgacttgacaggttgac 1

tgacacaaacctaatcgtcttgac 1

tgacacaagaccaaccgccttgac 1

tgacacaataagagtttcactgac 1

tgacacacactgacacacactgac 1

tgacacacatccaccaaatatgac 1

tgacacatatatgaagatgttgac 1

tgacacatatcaacattgactgac 1

tgacacatatgcatcaatactgac 1

tgacacatctcagctctcactgac 1

tgacacatgtccgagacatttgac 1

tgacacattaaaaaattaaatgac 1

tgacaccaaaaagccaaacgtgac 1

tgacaccagggttcgacacctgac 1

tgacaccgttacgtttccgttgac 1

tgacacctaaaatactacaatgac 1

tgacaccttaaatgctgcaatgac 1

tgacacgattcttccttacgtgac 1

tgacacgcataatgacataatgac 1

tgacacggtttaaataaatatgac 1

tgacacgtcatcagtaataatgac 1

tgacacgtgtgcatggtagctgac 1

tgacacgtgtttaaagattttgac 1

tgacactaagtacatggaagtgac 1

tgacactaattaacgaccattgac 1

tgacactacgcacatcatcatgac 1

tgacactagaatacattgtatgac 1

tgacactcaaaaaccagatctgac 1

tgacactctagcggcttcaatgac 1

tgacactgaaacattcaagatgac 1

tgacactgaaccattggagttgac 1

tgacactgaaggacaaccactgac 1

tgacactgactatgattgcctgac 1

tgacactggcacaccaatgttgac 1

tgacacttgacaacattacctgac 1

tgacactttatataacagtatgac 1

tgacacttttcgttatcttttgac 1

tgacagaagtataggtgatatgac 1

tgacagaatgcatatggtactgac 1

tgacagacgaagaatacatatgac 1

tgacagagcatttcgattactgac 1

tgacagagggcgccaaagtatgac 1

tgacagagtttcattctctttgac 1

tgacagcaattcacgtgccttgac 1

tgacagggttagcgataagttgac 1

tgacagtaatggtgatagtgtgac 1

tgacagtaggtattagctaatgac 1

tgacagtcggttcaactcgatgac 1

tgacagtggctaacgtcatgtgac 1

tgacagttgtattaaatttttgac 1

tgacataaaaatgaagataatgac 1

tgacataaaatttcactaaatgac 1

tgacataaataaactcactttgac 1

tgacataaatactcttcccatgac 2

tgacataagcaatacatctatgac 1

tgacataatgtttcaactcatgac 1

tgacatacgagatggttttttgac 1

tgacatatatgatgagtatatgac 1

tgacatatattgtatggtattgac 1

tgacatatgactttttctattgac 1

tgacatatttcacacggtcttgac 1

tgacatcaacgtcacgttggtgac 1

tgacatcaactagtctaagatgac 1

tgacatcaatacatcatctctgac 1

tgacatcactctgtaatcaatgac 1

tgacatcattatctccaatctgac 1

tgacatccatcttaacagattgac 1

tgacatcttgaactcttccctgac 1

tgacatctttattgattttgtgac 1

tgacatgccaacagctcacctgac 1

tgacatgcccggaatatacatgac 1

tgacatgctattttattaaatgac 2

tgacatgctttctacattgctgac 1

tgacatggtacccacggtattgac 1

tgacatggtcgtcgaggtaatgac 1

tgacatgtaaaactttggaatgac 1

tgacatgttatttgagtttttgac 1

tgacatgttccttagccttttgac 1

tgacattaaagaaacattattgac 1

tgacattagcatttttacgatgac 1

tgacattaggcagtcacttttgac 1

tgacattagtaaatagcaaatgac 1

tgacattcatcattactttctgac 1

tgacattgtaacagtttctttgac 1

tgacattgtcttcaagttgttgac 1

tgacatttaagttcagattttgac 1

tgacatttagttgttaaaaatgac 1

tgacatttgtcaatccggattgac 1

tgacattttgtatattttgctgac 1

tgacatttttccttcctctctgac 1

tgacatttttgttcatgaagtgac 1

tgacattttttgggggtttatgac 1

tgaccaaaaacgatatatagtgac 1

tgaccaaaattaatcgattttgac 1

tgaccaaacacaacttaagttgac 1

tgaccaaacttaatatataatgac 1

tgaccaaatctacttcaaactgac 2

tgaccaacaaacgaggatattgac 2

tgaccaagatcatttctttttgac 1

tgaccaagcctagaatgccatgac 1

tgaccaagttcctgggaaattgac 1

tgaccaagtttatagaacaatgac 1

tgaccaatcaattgaaaatgtgac 1

tgaccacattggagcttaagtgac 1

tgaccaccaccactactttttgac 1

tgaccaccgaacaagaacgatgac 1

tgaccactcttttgagattttgac 1

tgaccagcaaacgaggatattgac 1

tgaccagctgcaaagaaatgtgac 1

tgaccaggccaaaatccacatgac 1

tgaccaggcgtaattggctgtgac 1

tgaccagtagacgatagttgtgac 1

tgaccagtcaaagcatgctttgac 1

tgaccataagccggttgcgttgac 1

tgaccatccatgcctctgtgtgac 1

tgaccattatctactttctttgac 2

tgaccattgacacttacctgtgac 1

tgaccattgaccaaacgttttgac 1

tgacccacggtttggcggattgac 1

tgacccactgcaatctgtaatgac 1

tgacccatcttctctgttcatgac 1

tgaccccaagaaaccctttgtgac 1

tgaccccgtcaaaatgatgttgac 1

tgacccgactaatgacttattgac 1

tgacccgagatcaaggtgtttgac 1

tgacccgttcgtggaggacatgac 1

tgaccctaaatgtcagaaattgac 1

tgacccttttcttctaccggtgac 1

tgaccctttttgttgatatttgac 1

tgaccgaaaattcttcaacatgac 1

tgaccgaatcagagaagttttgac 1

tgaccgaatggaaaaaaaaatgac 1

tgaccgaccaaaaaatacaatgac 1

tgaccgataaaaccgaaccatgac 1

tgaccgatcatacatcgcactgac 1

tgaccgcctccgacaaccattgac 1

tgaccggagggagacattcttgac 1

tgaccggctgagaagtgatgtgac 1

tgaccgtcattgtaagatgatgac 1

tgaccgtctcttctctcttgtgac 1

tgaccgtgtgagtcaacagttgac 1

tgaccgttaattttgttccttgac 1

tgaccgtttcactttctagatgac 1

tgaccgtttcgttacgccgttgac 1

tgaccgtttggtatattctgtgac 1

tgacctaaaacattttctagtgac 1

tgacctaataaattatgtgttgac 1

tgacctatgctatatttatttgac 1

tgacctcggtattgcattactgac 1

tgacctcgttgatagatgtttgac 1

tgacctctaataaatttctttgac 1

tgacctgaaaagtcaatggttgac 1

tgacctgacccactcaaaattgac 1

tgacctgtgatactagtgtgtgac 1

tgaccttagaaatggaggtttgac 1

tgaccttccattacaaagaatgac 1

tgaccttctcttctgtttgttgac 1

tgaccttggtgtcgtaaacttgac 1

tgacctttcgagtaatcagctgac 1

tgacctttgaacacatgtcatgac 1

tgacctttgtttccaacatttgac 2

tgaccttttttactgtgcattgac 1

tgacgaaaaaccgcgtctagtgac 1

tgacgaaaattgatttgatttgac 1

tgacgaaaatttagtattagtgac 1

tgacgaagaagaaagaagagtgac 1

tgacgaagttacgtatacggtgac 1

tgacgagacccttgagcgtttgac 1

tgacgagatccaatggccgttgac 1

tgacgagctgacttgccgattgac 1

tgacgagtatattgcgtgcttgac 1

tgacgagtttcgatcaatggtgac 1

tgacgatattataccagaagtgac 1

tgacgatcacattaaccacatgac 1

tgacgatctccatctcttcttgac 1

tgacgcaaattttgtaactttgac 1

tgacgcaacccgtgacgcggtgac 1

tgacgcacgttaaacgactctgac 1

tgacgcagacagaaagaacgtgac 1

tgacgcatcaaaaaagctaatgac 1

tgacgcattatgggcttaactgac 1

tgacgcatttgtcagaaacctgac 1

tgacgccaactttaaaattttgac 1

tgacgccgtattgattgtagtgac 1

tgacgccgtttcgccgcatatgac 1

tgacgcctcaacaactgccatgac 1

tgacgcggttccgagttttctgac 1

tgacgctgatacagatggcatgac 1

tgacggagcaccacgcgcagtgac 1

tgacggcaaagacgaatgcttgac 1

tgacggcaaattggagcaactgac 1

tgacggcagtgtgatcttaatgac 1

tgacggcatgagagttattatgac 1

tgacggcgttaactgttagatgac 1

tgacggcttttggctttcactgac 1

tgacgggctgacttgtcgattgac 1

tgacggtaaaatctacgtggtgac 1

tgacggtaatacgattgtagtgac 1

tgacggtaggggataccgtttgac 1

tgacggtgtttgggacgtgatgac 1

tgacgtagttaccacgctcttgac 1

tgacgtagtttctaagcttttgac 1

tgacgtataatatggaaaactgac 1

tgacgtcaaaaacaagtaaatgac 1

tgacgtcggtattttatacttgac 1

tgacgtggaacgttttgtgctgac 1

tgacgtggagacccatgggatgac 1

tgacgtggggagaaggaacatgac 1

tgacgtgtaatcttgactcttgac 1

tgacgtgtcgaatttaataatgac 1

tgacgttaagtagcagaacatgac 1

tgacgttgtgatggtagtggtgac 1

tgacgtttcaaaattttgattgac 1

tgacgttttgttgtgacttatgac 1

tgactaaaatttgttaatgttgac 1

tgactaaacttgtactgttctgac 1

tgactaactgagaaacagagtgac 1

tgactaagctcctctgataatgac 1

tgactaatgcgttggatagatgac 1

tgactaattactccgaaaactgac 1

tgactaattatttcaagagttgac 1

tgactacgcagacgattatatgac 1

tgactactcttctttcaaattgac 1

tgactactgatcccacaatctgac 1

tgactactgctacgtctgtgtgac 1

tgactagagacatttattggtgac 1

tgactagatactctttaaactgac 1

tgactagatctgttgtttgatgac 1

tgactagatgaattcaaacttgac 1

tgactagggttgaccggcgttgac 1

tgactagtgatgatgtctcgtgac 1

tgactagtggagatgtgttttgac 1

tgactagtggtgcaacctgctgac 1

tgactataaaatcataaaaatgac 1

tgactataacttcgaataattgac 1

tgactatcaaacaaagccaatgac 1

tgactatgatcttcccaaattgac 1

tgactatgattaaacgttaatgac 1

tgactatggttttaagttcttgac 1

tgactattatccacaacgcgtgac 1

tgactattgccaaccttatctgac 1

tgactatttgataaacaacttgac 1

tgactatttggaaccataaatgac 1

tgactattttgtgacgttcttgac 1

tgactcaaaaattgaattattgac 1

tgactcaaagaactcagacttgac 1

tgactcaaaggacgagagggtgac 1

tgactcaaatcccatctggatgac 1

tgactcaactcacatgtgtatgac 1

tgactcaatataattattattgac 1

tgactcactagctaatgcattgac 1

tgactcagcaaaaaagctaatgac 1

tgactcatactatcttctcctgac 1

tgactcatgagcagaccaattgac 1

tgactcccatcattaattagtgac 2

tgactcgtaactcgtaagcgtgac 1

tgactcgtagacaaccgctttgac 1

tgactcgttatgaagtgttttgac 1

tgactctatcatgtagtatctgac 1

tgactctcgtaattgatggctgac 1

tgactctgacaacaaaacagtgac 1

tgactctgtatattcaaggctgac 1

tgactctgtcactcttccagtgac 1

tgactctgtctcatattatttgac 1

tgactcttacgagatctctttgac 1

tgactcttataattgatggctgac 1

tgactcttgtgcttgaattctgac 1

tgactgaagaaattaggttttgac 1

tgactgatttcatcaactaatgac 1

tgactgcagggatcactccttgac 1

tgactgcgtgtaaagtaatctgac 3

tgactgctactatataagtatgac 1

tgactggccagtacacaagttgac 1

tgactgggaaggtacttgcgtgac 1

tgactgggtcaggagaatcttgac 1

tgactggtggtgtacaacaatgac 1

tgactggtgtaatggttctttgac 1

tgactgtaaatgttaagctttgac 1

tgactgtaggggtctttatatgac 2

tgactgtatgttctaattgttgac 1

tgactgtgacataagttaaatgac 1

tgactgtgactctcttttattgac 1

tgactgttagtgcattctgatgac 1

tgacttaaaacaatttgatgtgac 1

tgacttaaaccaagacatggtgac 1

tgacttaaatataaaattcctgac 1

tgacttaacatctctgtggatgac 1

tgacttacacccactaatattgac 1

tgacttacattcaaaaaatttgac 1

tgacttaggttgaaaaactctgac 1

tgacttagtcgctcttatattgac 1

tgacttatacaattcgtacatgac 1

tgacttatgatatgaataaatgac 1

tgacttattaagttcaattatgac 1

tgacttcagatgacgattaatgac 1

tgacttcctacattttaccttgac 1

tgacttcgtggatagatgtttgac 2

tgacttctagttgatattgttgac 1

tgacttctgagagaggaatctgac 1

tgacttctggttcatgagattgac 1

tgacttcttaacataaatgctgac 1

tgacttgaaaattctcattttgac 1

tgacttgaaacattaccaaatgac 1

tgacttgagaagaaggtttatgac 1

tgacttgatatttattctcttgac 1

tgacttgatttattagttgttgac 1

tgacttgcatattggcgagatgac 1

tgacttgccgattgacgagttgac 1

tgacttgcgcgtggtatagatgac 1

tgacttggccccttcgttcttgac 1

tgacttgtcgattgacgagctgac 1

tgacttgttcgcgaaggatctgac 1

tgactttaagcaaatacttttgac 1

tgactttcactgtctctttgtgac 1

tgactttcagccgtcgacgttgac 1

tgactttcatataaaaataatgac 1

tgactttctacaaaaaaatgtgac 1

tgactttctccagtttggattgac 1

tgactttgaaaaagtgaatgtgac 1

tgactttgaaagagttttaatgac 1

tgactttgaaatgcatttcttgac 1

tgactttgagatcaaaatcttgac 1

tgactttgagtcttctttgttgac 1

tgactttgcactattgaaactgac 1

tgactttgctatgctctgtttgac 1

tgactttgttcaatatttgttgac 1

tgacttttaaggaaatgaaatgac 1

tgacttttagcttttgacattgac 1

tgacttttgaaaaagaaagttgac 1

tgacttttgaaacaatatattgac 1

tgacttttgaaagcctaaattgac 1

tgacttttgagaaatcggtatgac 1

tgacttttgagcaatgatcttgac 2

tgacttttgattgaaaaaaatgac 1

tgactttttcttttttggtttgac 1

tgacttttttttggaagacttgac 1

17 : 402

tgacaaaaaatcatatgggcttgac 1

tgacaaaaacatagctatccatgac 1

tgacaaaactaaaattaaatctgac 1

tgacaaaactatagccatctttgac 1

tgacaaaagatccaaaagtgttgac 4

tgacaaaagatctaaaagtattgac 1

tgacaaaatatccaaaagtattgac 1

tgacaaaatgagattgtgttttgac 1

tgacaaacgcgagttcatttttgac 1

tgacaaagaaccaatatatattgac 1

tgacaaagaagatggcgatgatgac 1

tgacaaagagatgaagctaggtgac 1

tgacaaatatgaatcaaaatatgac 1

tgacaaatccacaccacatgttgac 1

tgacaaatgaattaagagtaatgac 1

tgacaaatttaatttatgtcatgac 1

tgacaacaaataattttattttgac 1

tgacaacaaatcaacaatatgtgac 1

tgacaacacctgagaaatttgtgac 1

tgacaacattgggaatgggagtgac 1

tgacaacccataacgcatgactgac 2

tgacaactcaacttcagtgaatgac 1

tgacaactcatccaagagatatgac 2

tgacaactgctttggatttgatgac 1

tgacaacttttcaggttcttttgac 1

tgacaagaaaaaaaaaacatttgac 1

tgacaagagtggacttagctctgac 1

tgacaagatcaaacttctctctgac 1

tgacaagatcagtttcagagttgac 1

tgacaagatgttgattttggttgac 1

tgacaagcaggatgtgatctctgac 1

tgacaagccaaactagatatctgac 1

tgacaaggacccgcaaggccctgac 1

tgacaataaaataatatgacctgac 1

tgacaatacataacaataatatgac 1

tgacaatataattatagtatgtgac 1

tgacaatattaaacatgaaattgac 1

tgacaatcatatggagagagttgac 1

tgacaatgacatttaccttgttgac 1

tgacaatggaggtttccaggttgac 1

tgacaatggtgaagtcaagactgac 1

tgacaattgagaaaaaaagtttgac 1

tgacaattgctctttattttctgac 1

tgacaattggcggcactgtgttgac 1

tgacaatttgggttaccaagttgac 1

tgacaattttttctcctcttttgac 1

tgacacaaaaaaaaatacatttgac 1

tgacacaaactcaggtgtttctgac 1

tgacacaatatataaaattaatgac 1

tgacacacacacaagacaatgtgac 1

tgacacacataagccgacatctgac 1

tgacacaggtaaaggaaaccatgac 1

tgacacataaataagtgtaattgac 1

tgacacattgggatgggctaatgac 1

tgacacatttttttttacatgtgac 1

tgacaccagatcttgtgtggatgac 1

tgacaccccctatagctgcattgac 1

tgacaccgaagctgatatttgtgac 1

tgacacgtcaccaccattctctgac 1

tgacactatgtcaaatcaatctgac 1

tgacactattattgaattgtttgac 1

tgacactattccagacgaatttgac 1

tgacactcctaacttcctatgtgac 2

tgacacttgtcacagtgctgttgac 1

tgacacttttctttttctagatgac 1

tgacagaagatctaaaagtattgac 1

tgacagacgatataaaagtattgac 1

tgacagagacagctttgtgtttgac 1

tgacagagttgttttatgttgtgac 1

tgacagattagtttctagtgatgac 1

tgacagcgattggtagcagaatgac 1

tgacagctccgacgatggcagtgac 1

tgacaggagcgaaattggtcttgac 1

tgacaggatcagtgccttgcctgac 1

tgacaggttacttgaccggagtgac 1

tgacagtgcttgtttcagatgtgac 1

tgacagttaaacagcttaaaatgac 1

tgacataaaaacgcagcaaaatgac 1

tgacataaacacataatttgatgac 1

tgacataaaccatggaagagttgac 1

tgacataacacgagaggagaatgac 1

tgacataagtttttttacatttgac 1

tgacataatggttttaagttttgac 1

tgacatacacaacaaaatatgtgac 1

tgacatagtcaccttacattgtgac 1

tgacatagtccattgacataatgac 1

tgacatagtttcgttcgtagatgac 1

tgacatatctatatgttttggtgac 1

tgacatatttgccttagtttttgac 1

tgacatcaccgccttactaattgac 1

tgacatcagtgggtctagatctgac 1

tgacatcccaattatcaattgtgac 1

tgacatccctaacgaagtatatgac 1

tgacatcgatgtatctgaggttgac 1

tgacatcggatgtaagtaaaatgac 1

tgacatctggtattgctcgcttgac 1

tgacatcttcaagaaccatgttgac 1

tgacatcttcctctttctctttgac 1

tgacatcttcgacgacaattttgac 1

tgacatctttgtttgttcatatgac 1

tgacatgagtaaaggataatttgac 1

tgacatgataagtccgattcatgac 1

tgacatgataatcggattatttgac 1

tgacatgatcatcagcccatttgac 1

tgacatgctcttcgagatatctgac 1

tgacatggagaagtatgcagatgac 1

tgacatgtataacatcatatttgac 1

tgacatgtcagcgtcagaagatgac 1

tgacatgtgacgcaaaatgcgtgac 1

tgacattaaaagcaaagaacatgac 1

tgacattattgaaccacagaatgac 1

tgacattccaattaagataaatgac 1

tgacattgatgttacatttgatgac 1

tgacattgattctcttttggatgac 1

tgacattgctatcttcatgtatgac 1

tgacattggagagaaagcttatgac 1

tgacatttaaaaccaaaagcttgac 1

tgacatttattctttggcttttgac 1

tgacatttccaactctacatgtgac 1

tgacattttataaatttttaatgac 1

tgacattttatattgcagatatgac 1

tgacatttttaggtacctttctgac 1

tgaccaaaaagtctctgaagatgac 1

tgaccaaacaaacaagacacgtgac 1

tgaccaaacacgccacacaattgac 1

tgaccaaacttgagaggagtatgac 1

tgaccaaagaaaaaaaaaaattgac 1

tgaccaaagttataggaaccatgac 1

tgaccaaagttttgggggaaatgac 1

tgaccaaatatgttttatttttgac 1

tgaccaaatattatctccaaatgac 1

tgaccaacgacttgaactccttgac 1

tgaccaactctacctcgacgatgac 1

tgaccaagaaaacaatcaggctgac 1

tgaccaagggccgatgatgagtgac 1

tgaccaataaactttattatttgac 1

tgaccaattaattatgcgaattgac 1

tgaccaattagatgtaaaaaatgac 1

tgaccacaatctcccactacatgac 1

tgaccacaatgggtcatacattgac 1

tgaccacacgacgttattaaatgac 1

tgaccacataagacgtcattgtgac 1

tgaccacatgaagaaaatgagtgac 1

tgaccactacatcatctattttgac 1

tgaccagaaaaatattcgacatgac 1

tgaccagaatctgttcatcattgac 1

tgaccagatacgtcgtcgtagtgac 1

tgaccagatacgtcgtcgttgtgac 1

tgaccagtaatatgtacactttgac 1

tgaccagtgatgtgacgattttgac 1

tgaccatagaggatggataattgac 1

tgaccatattaatttgtaaactgac 1

tgaccatatttatgaactatatgac 2

tgaccatcatagcatttcagttgac 1

tgaccatgaaaagactgacactgac 1

tgaccatgcagcatttgattttgac 1

tgaccatgctcggtgaaatactgac 1

tgaccatggactctatgcaattgac 1

tgaccatgtagcatttgattttgac 1

tgaccatgtgaccaacatttatgac 1

tgaccatgtttggtgaattactgac 1

tgaccattgaactttctatattgac 1

tgaccattgggtcaaattgagtgac 1

tgaccatttgcttattttgcttgac 1

tgacccaaacccaactggatctgac 1

tgacccaaaggaaaaaaattgtgac 1

tgacccaagaaaagtggaacttgac 1

tgacccaagctctgttgttgttgac 1

tgacccaattatgtgatcttatgac 1

tgacccacgaaaatgtggttttgac 1

tgacccatgtgcctgcaacgatgac 1

tgaccccaatattactccctttgac 1

tgacccccaaaaaaatatatttgac 1

tgacccgaatgatcctaattatgac 1

tgacccgctcatcaagccaaatgac 1

tgacccgggtccccaaccacctgac 1

tgacccggtgtacattaatcttgac 1

tgacccgtgatccaacctggttgac 1

tgacccgtttcaacatcaatttgac 1

tgacccgttttgtattatccatgac 1

tgaccctactttgtatgtttctgac 1

tgaccctgaacagttaagatctgac 1

tgacccttgagaggaattcagtgac 1

tgacccttgtaaagagacttttgac 1

tgaccgaagagcctttgcatttgac 1

tgaccgacggctccaagtgtgtgac 1

tgaccgattcgattttgggcgtgac 1

tgaccggaaaatgttcaggagtgac 1

tgaccggacatgagtataatatgac 1

tgaccggatattacccgaacttgac 1

tgaccggctccaacaaaactttgac 1

tgaccggcttggtttttcatttgac 1

tgaccgtatctatggagtctttgac 1

tgaccgttaagatcaattagctgac 1

tgaccgttaatatcttgcatttgac 1

tgacctaacctttttaaattttgac 1

tgacctaatgactatttcttttgac 1

tgacctaatttaacttcttactgac 1

tgacctaccttctctttttattgac 1

tgacctagtattttatagttttgac 1

tgacctccgctgctatatctttgac 1

tgacctctgcatccaaggatctgac 1

tgacctcttcatataaatagctgac 1

tgacctgagttagacgaagactgac 1

tgacctgttgttattagtaattgac 1

tgaccttatgtcttactcacgtgac 1

tgaccttcacctgtcttcttttgac 1

tgaccttgaattctaatactgtgac 1

tgaccttgtttggattcatagtgac 1

tgacctttgtaccctttttgctgac 1

tgacctttttctatcatgatttgac 1

tgacgaaaatatgtatgtctgtgac 1

tgacgaaacttttacttaacgtgac 1

tgacgaaagaacgttgatacttgac 1

tgacgaagacgatgaaatcactgac 1

tgacgaagacggtgaaaacaatgac 1

tgacgaaggagttgattcacgtgac 1

tgacgaatcaatccacacgaatgac 1

tgacgaatcgaaccgttggattgac 1

tgacgaatctaggtgagaaactgac 1

tgacgaatctttaagaaccagtgac 1

tgacgaattttttctgtgatatgac 1

tgacgaccaatattcttgaaatgac 1

tgacgacccaccttgcccttttgac 1

tgacgactaatgaatgatggatgac 1

tgacgactacagctctacttgtgac 1

tgacgagatcaaaaacaaaattgac 1

tgacgaggcagatgatatctgtgac 1

tgacgagggtttatcttcttttgac 1

tgacgagtagttgtctgatattgac 1

tgacgagtcttagctccgcattgac 1

tgacgatacatatacaattcctgac 1

tgacgatcattacactggttttgac 1

tgacgatcctcttgttacatgtgac 1

tgacgatgattggtttgcttttgac 2

tgacgccaagagaaatgctcgtgac 1

tgacgcccattagcatgttagtgac 1

tgacgcctccgatgatagacctgac 1

tgacgcttgctcacgacttgttgac 1

tgacggaaaacccaatgagggtgac 1

tgacggaatctgaaaagactatgac 1

tgacggagcgataagatggtatgac 1

tgacggatccttactgttggatgac 1

tgacggattagtattaggaactgac 1

tgacggcgctcgcacggagcctgac 1

tgacggcggcagtggtggtgatgac 1

tgacggcgtaaaccagtccaatgac 1

tgacggcgttaacaaaaatgatgac 1

tgacggcgttgcagacaacaatgac 1

tgacgggctagtgacttgctgtgac 1

tgacggtaataatatgtcaattgac 1

tgacggtataattatgtcaattgac 1

tgacggtcggttttgttgttgtgac 1

tgacggtgacggagaagatggtgac 1

tgacggtgcaattcttggtgatgac 2

tgacgtaaaacatcctagttgtgac 1

tgacgtaaagcctctcaattatgac 1

tgacgtactctaaaccttttttgac 1

tgacgtatcaaacgcttatcttgac 1

tgacgtattcaacctcctgtatgac 1

tgacgtcaaattttaaaattttgac 1

tgacgtcaatgatcgtggggatgac 1

tgacgtcatttcccttccaaatgac 1

tgacgtcgttttagcccacactgac 1

tgacgtgcattagttatgttttgac 1

tgacgtggcagagatagtagctgac 1

tgacgtgggacggttttcatttgac 1

tgacgtggtcaattggctatctgac 2

tgacgtgtggacagagtaccatgac 1

tgacgtgttaacacgagcctatgac 1

tgacgttagcaataagcttcatgac 1

tgacgttataatttttgctaatgac 1

tgacgttcagattgagtagggtgac 1

tgacgttggccaggtggtcgttgac 1

tgacgttggtctctaatctgatgac 1

tgacgtttccatgatgtactgtgac 1

tgacgtttgagaagaggataatgac 1

tgacgtttggacttgtcattgtgac 1

tgacgttttttattgatatgatgac 1

tgactaaaaagcctaaaacattgac 1

tgactaaaagaaaatcagatctgac 1

tgactaaaaggtctttagctttgac 1

tgactaaatatgtacatgttttgac 1

tgactaaattcctttaaatcatgac 1

tgactaacctagtcttccatatgac 1

tgactaagccaactaaaatattgac 1

tgactaagctaactaaaatattgac 1

tgactaatgaggcttgaattgtgac 1

tgactacacagtaatgcatgatgac 1

tgactacacttttcttacttttgac 1

tgactacatatacatcaaccatgac 1

tgactacatatacgaaatgtatgac 1

tgactacgaggatgaagatgatgac 1

tgactacggcgagagaatgcttgac 1

tgactagatacgtcgtcgttgtgac 1

tgactagatgtaaaaagtatatgac 1

tgactagccatacaagaatattgac 1

tgactagtacctcgcaaaacatgac 1

tgactagttacagctactcaatgac 1

tgactataacttttcttttactgac 1

tgactatacttgttgggcagttgac 1

tgactatgaaaaaaatattcttgac 1

tgactatgaaataataaagtgtgac 1

tgactatgatgtcatagttattgac 1

tgactatgattgatgactatgtgac 1

tgactatggtacgtacggtgatgac 1

tgactatgtacaaatgtgcgttgac 2

tgactattagcagcacaggtatgac 1

tgactattgatcgtgtacatttgac 1

tgactcaagttttacactttttgac 1

tgactcactaaaacccgcgcgtgac 1

tgactcagcaaccgtaacagttgac 1

tgactcagcaactgtaacagttgac 1

tgactcagtaatcattagcattgac 1

tgactcataatccctcgcacgtgac 1

tgactcatagtcaataggagttgac 1

tgactcatgtcatccatattatgac 1

tgactcattcaacaaaaagagtgac 1

tgactccacaagtcgtcgatttgac 2

tgactccaccacccatgcccatgac 1

tgactccaccacctatgcctatgac 1

tgactccatattttgacaacgtgac 1

tgactccgagctctgtttcgatgac 1

tgactcgtccaaatttccttttgac 1

tgactcgtggcgcacagtcaatgac 1

tgactctacctttgtaaatattgac 1

tgactctgctatttgttactttgac 1

tgactctgtttgaccaaataatgac 1

tgactcttgagcattagaatgtgac 1

tgactctttcgaatgtgtgtgtgac 1

tgactctttgtagttgaacattgac 1

tgactgaaagtgaatattggttgac 1

tgactgaagaaatggaacacttgac 1

tgactgaagctgatgtggcattgac 1

tgactgacacatctatcctcgtgac 1

tgactgacattcaaaaagaagtgac 1

tgactgaccattcttatcaactgac 1

tgactgagtaagattaggtggtgac 1

tgactgatacataacatgttgtgac 1

tgactgatgaaacctttcacgtgac 1

tgactgatgataggtataagttgac 1

tgactgattttgtttgcatgatgac 1

tgactgcttatttcgtccaactgac 1

tgactggtctgcaaagctcgatgac 1

tgactgtacgatctttttatttgac 1

tgactgtctattgagctcagttgac 1

tgactgtgtcacgaaagtctctgac 1

tgactgtttgttaccacgttttgac 1

tgacttaacagagaaaatggttgac 1

tgacttaatacatgtgagttgtgac 1

tgacttacgctgctagtgagttgac 1

tgacttactacagcagatttttgac 1

tgacttatatatacgttactgtgac 1

tgacttatatatcgttgagagtgac 1

tgacttatcatttacaagtaatgac 1

tgacttattaagctcatattttgac 1

tgacttcaaaaactttgttcatgac 1

tgacttcacaacgcatttcattgac 1

tgacttcagaagatctatctttgac 1

tgacttcgtaagttcattctttgac 1

tgacttctgcctatatatgcttgac 1

tgacttgaataccctccgttatgac 1

tgacttgagcttagccacacatgac 1

tgacttgagttcatattataatgac 1

tgacttggtgaatttaattagtgac 1

tgacttgtatgacttgtagtatgac 1

tgacttgtccgcatcatcatgtgac 1

tgacttgtcggaaataggagatgac 1

tgacttgtcgtcttcttcttctgac 1

tgacttgtgaatattcttttttgac 1

tgacttgtgagatctcatagttgac 1

tgacttgttagatgggatgcatgac 1

tgacttgttgcggcagagttttgac 1

tgacttgttgtatgttgttattgac 1

tgacttgttttttggtgtgtgtgac 1

tgactttagagatggtggggttgac 1

tgactttattcttgataaggatgac 1

tgactttcatgttcttatatctgac 1

tgactttctcatctcttctgttgac 1

tgactttctctagatattggttgac 1

tgactttgtgggtgatttgtttgac 1

tgacttttaagattgtgggattgac 1

tgacttttagttataatatcttgac 1

tgacttttgaagtgacctcgttgac 1

tgacttttgacatatgacaaatgac 1

tgacttttgagattgtaggattgac 1

tgacttttgagattgtgggattgac 1

tgacttttgtcgtgacgctcatgac 1

tgactttttgacttttgtcgttgac 1

tgactttttgttaatcaattttgac 1

18 : 352

tgacaaaaaaaaacaaaaaaaatgac 1

tgacaaaaaaaaagacatgacgtgac 1

tgacaaaaaaaaggaaaacttttgac 1

tgacaaaaaataaataaaagtctgac 1

tgacaaaacattcatatgataatgac 1

tgacaaaataattttattaaattgac 1

tgacaaaccaatcaattatttgtgac 1

tgacaaagaatttgacgaattgtgac 1

tgacaaagcaaagattggtatatgac 1

tgacaaatataacaattaattttgac 1

tgacaaatcaactgagttacgttgac 1

tgacaaatgcatttaggatttctgac 1

tgacaaattattggcgaaatggtgac 1

tgacaacaaatctgttacaatgtgac 1

tgacaacaaatttgttacaatgtgac 1

tgacaacatatatttagttgtctgac 1

tgacaaccacctcgactctctttgac 1

tgacaaccgaaacagaagcacatgac 1

tgacaacgaacaagtgttaagttgac 1

tgacaacgaggatgtcacacattgac 1

tgacaagatatgtaacgatatttgac 1

tgacaagattaatatatttagctgac 1

tgacaagcaataaatgaaatgctgac 1

tgacaaggaaagtatgtggtattgac 1

tgacaaggagaatttaacactttgac 1

tgacaagggttgtggatgattatgac 1

tgacaaggttttttgagtctattgac 1

tgacaagttatatcatttgctctgac 1

tgacaagtttgcactcactagatgac 1

tgacaataacaaattaggtaaatgac 1

tgacaatacctctctttttcaatgac 1

tgacaatcttcataactataattgac 1

tgacaatgaactagaatttagttgac 1

tgacaattattgataagcttggtgac 1

tgacacaaactaaaacgacacgtgac 1

tgacacaaactaattagaatgttgac 1

tgacacaaggtatctaagggcatgac 1

tgacacaaggttgttagagaaatgac 1

tgacacaatcttcatccatgaatgac 1

tgacacacatgatctcctttattgac 1

tgacacagcacttgctattccatgac 1

tgacacagctttgaatcttgcatgac 1

tgacacaggcacagctaataggtgac 1

tgacacatcatgaactcacttgtgac 1

tgacacatccgcttgatttaattgac 1

tgacacatcgacgctagattactgac 1

tgacacatctacttgaaattaatgac 1

tgacaccacattggttcattattgac 1

tgacaccgactttggacttagttgac 1

tgacacctgtcaagatctacaatgac 1

tgacacggttacaaactgacagtgac 1

tgacacgttctccttttttggttgac 1

tgacacttactcgtagatgaggtgac 1

tgacagactcttattggtaatgtgac 1

tgacagagaacggtcaagctgttgac 1

tgacagaggaaagacgtctctttgac 1

tgacagcatatgtttttttatctgac 1

tgacagcattcattaaatcaattgac 1

tgacaggatttggagtaatgtctgac 1

tgacaggtggttgtaatttcattgac 1

tgacagttcttacatatgtggttgac 1

tgacagtttggaattcctcatgtgac 1

tgacataaacaagaaacaattctgac 1

tgacataagttgtaaacagtaatgac 1

tgacataagtttccattcacattgac 1

tgacataatggatttgtctttatgac 1

tgacataatttcctttccttgatgac 1

tgacatagaatgtacattgaattgac 1

tgacatagacaaagtctacgcttgac 2

tgacatagttatggcattattatgac 1

tgacatattaattccaaatcattgac 1

tgacatatttttatttctgaattgac 1

tgacatatttttttcatttgcgtgac 1

tgacatcaggagaaacccccgatgac 1

tgacatccggagaaacccctgatgac 1

tgacatcttggattactacttctgac 1

tgacatgaaatttgcgaaatagtgac 1

tgacatgcttcagtatttgatatgac 1

tgacatggataaaacttaagtctgac 1

tgacatgtagcatctttctttgtgac 1

tgacatgtcaattattttgaaatgac 1

tgacatgtgaatttatatctattgac 1

tgacattaaatgtttcatattttgac 1

tgacattgaatttctggggttatgac 1

tgacattgtgaccactgaccagtgac 1

tgacatttaccattttcttgattgac 1

tgacatttcaatggcttttccctgac 1

tgacatttttctctctaaactttgac 1

tgaccaaaaaaatcaataatattgac 1

tgaccaaaactcaacagtaatatgac 1

tgaccaaagaattgagatctgatgac 1

tgaccaaatcagaatttgtttatgac 1

tgaccaactctctccatatgattgac 2

tgaccaagaactacgagtcctgtgac 1

tgaccaattctctccatatgattgac 1

tgaccacacatcttgtattgattgac 1

tgaccactatgaatgtattaaatgac 1

tgaccactatgcctgggcctattgac 1

tgaccactattttcatcatgcgtgac 1

tgaccagaactgtttgcatgtgtgac 1

tgaccagatttattcaatcccttgac 1

tgaccagcatactcattttaactgac 1

tgaccagctgctgtctgtacgttgac 1

tgaccagcttcaaacacatacttgac 1

tgaccagtgtgactttgcacagtgac 1

tgaccatatttgaaatatcaaatgac 1

tgaccatctatcatccaaccgatgac 1

tgaccatgattcttcccgatggtgac 1

tgaccatgctataaagctattatgac 1

tgaccattggaattgttgcgcatgac 1

tgaccatttcaacccgtgggtttgac 1

tgacccaaaatactcctattaatgac 1

tgacccaagtcgctgaaatgtttgac 1

tgacccaatatttctagaaagttgac 1

tgacccactaccatatacattttgac 1

tgacccatttattagtagttagtgac 1

tgacccggactaaccaagcccatgac 1

tgacccgttatagtttcatgcttgac 1

tgaccctcttacaggttagttttgac 1

tgaccctttcccaaaatattattgac 1

tgaccgaagtttattttgcggatgac 1

tgaccgagattgttcgcatgcttgac 1

tgaccgagtcttttattgacagtgac 1

tgaccgcctgtcgaccgtttgctgac 2

tgaccgggtcagaattgataggtgac 1

tgaccgtagaggctacaagcattgac 1

tgaccgtgacatttttttaatgtgac 1

tgaccgttaaactaagattttgtgac 1

tgacctaaattataccctaaactgac 1

tgacctaactcttaatgtcttgtgac 1

tgacctaggtcttggagtctcctgac 1

tgacctagtcaaagttaggcattgac 1

tgacctatacatagtcaagctatgac 1

tgacctcatccagttttgcatttgac 1

tgacctcatctcaatacgaagatgac 1

tgacctctattactacataaaatgac 1

tgacctgattatttattttggctgac 1

tgacctgcacatgtcaagtttttgac 1

tgacctgcatgacattgttttctgac 1

tgacctgtgactccaagttcaatgac 1

tgaccttacatgttcatgacgttgac 1

tgaccttagacattggaatgagtgac 1

tgaccttatacataacatcccatgac 1

tgaccttcacatgtcctagtgatgac 1

tgaccttgtagtaaagcattcttgac 1

tgacctttaaaactcatcactatgac 2

tgacctttcatttaattttcagtgac 1

tgacctttcttctacaaatctctgac 1

tgacctttggaaactggataaatgac 1

tgacgaaaaaaaatatatatattgac 1

tgacgaaaaatgtccctttcattgac 1

tgacgaaacatttatttgacattgac 1

tgacgaaatctaataatcttggtgac 1

tgacgaaatgttttgcaatctttgac 1

tgacgaactggactcttaactctgac 1

tgacgaagaagatatgaaatgttgac 1

tgacgactctactgcccaatcttgac 1

tgacgactgatagccacaaaattgac 1

tgacgagaaacaaaattgattttgac 1

tgacgagaaatttcttaagagatgac 1

tgacgagtctccatcacttcgttgac 1

tgacgagttcataaacgaatattgac 1

tgacgagttttttcctcgagtctgac 2

tgacgataaacaaactcaattttgac 1

tgacgataagtgaagatacgagtgac 1

tgacgatacaaaaagaagactttgac 1

tgacgatagatgacaatgcccatgac 1

tgacgatggtccaaagatgccgtgac 1

tgacgcaagagttccacagttgtgac 1

tgacgcataatcatttaaaaaatgac 1

tgacgcctagctgctacggtcatgac 1

tgacgcctccaaaattagaatgtgac 1

tgacgcgtgtaatcaacgcgcatgac 1

tgacgctaacattcaaaataagtgac 1

tgacgcttgaagtatgggaccatgac 1

tgacggtaacggtaacggcaattgac 1

tgacggtagtaatagtccttaatgac 1

tgacgtacatgaattttaaggttgac 1

tgacgtacttatatgatgcatgtgac 1

tgacgtacttgattatccatagtgac 1

tgacgtagaccatcatgtagtttgac 1

tgacgtatgattcccttgaacttgac 1

tgacgtatttctttgaaccgtttgac 1

tgacgtcaaaaaaactgtgaggtgac 1

tgacgtcacctgttaggttgcgtgac 1

tgacgtccttctcaacgtatgttgac 1

tgacgtcggttcttattgaccttgac 1

tgacgtgacaaatcatacttggtgac 1

tgacgtgagaggaaaatccaaatgac 1

tgacgtggactttttgggctattgac 1

tgacgtggccgatccctcaacgtgac 1

tgacgtgggacctttaagattatgac 1

tgacgtggttataaagcagtaatgac 1

tgacgtgtaaaacatgccccgttgac 1

tgacgtgtactgtttaatataatgac 1

tgacgtgttattttcaagagtatgac 1

tgacgttaaggtaaaaataaactgac 1

tgacgttcataaaccttttcattgac 1

tgacgttcattggatgatcccgtgac 1

tgacgtttctaatgcttcctaatgac 1

tgacgtttgtagcccttacgtttgac 1

tgacgttttttaacattttgagtgac 1

tgactaaaaaactgttagacaatgac 1

tgactaaaaacttttgaaatcatgac 2

tgactaaagagaaaatcaatgttgac 1

tgactaaagagataaaaatatgtgac 1

tgactaaatatcaatcaaaacatgac 1

tgactaacctagattatgtaaatgac 1

tgactaacgaatggctgaggcttgac 1

tgactaactttctccatatgagtgac 1

tgactaatagtgtgtttgggtatgac 1

tgactaatggacaaattggatgtgac 1

tgactaattatcagttacgatttgac 1

tgactacaaagtctaaaacttttgac 1

tgactaccttttgatgcttggctgac 1

tgactactaaaaaaaaaatctttgac 1

tgactactagcatataacatcttgac 1

tgactacttgactggtgttcgttgac 1

tgactagaagtctcaagttaattgac 2

tgactagaataagtgttggttttgac 1

tgactagagtcgttttcgtctttgac 1

tgactagtaagtaactagtttttgac 1

tgactagtattttaatttattttgac 1

tgactagtggacgaagtttgaatgac 1

tgactatacatatattactctgtgac 1

tgactatctaaagaaattatcttgac 1

tgactattaaaaaatctatatttgac 1

tgactattatactccccaaatttgac 1

tgactattatgacgtaagagattgac 1

tgactattgactaaaagattattgac 1

tgactcaaacagagataagatatgac 2

tgactcaataatttaacataagtgac 1

tgactcacacatcttatgcagttgac 1

tgactcagagatttctgcagattgac 1

tgactcatatattgtagacttatgac 1

tgactcatcatcgcgtgggctttgac 1

tgactcatgaccattacaaccatgac 1

tgactcatgttaaactaatatttgac 1

tgactccattgcgttggccatgtgac 1

tgactccggcgttgactagggttgac 1

tgactcggaaccctgtgactcgtgac 1

tgactcggtttggtttggtgggtgac 1

tgactcgttgtgttttgtaatctgac 1

tgactcgtttatttaagtacactgac 1

tgactctaacggaaaccagaattgac 1

tgactctacatgcaataaggtttgac 1

tgactctatattagcctaattttgac 1

tgactctattttaatccaattttgac 1

tgactctattttagcctaattttgac 1

tgactctcaaacgcgttttcgttgac 1

tgactctctcattggtcaaacctgac 1

tgactctcttccatctcttgtttgac 1

tgactctgttttatgttaattgtgac 1

tgactcttaccttcatgagagctgac 1

tgactcttcacacatttcaatatgac 1

tgactcttgtgtaaccatcatatgac 1

tgactgaaaataatcaatttagtgac 1

tgactgaaagataggaacactatgac 1

tgactgactatgacgaggtctctgac 1

tgactgactgggattggtgcagtgac 1

tgactgagtttggtttatgttttgac 1

tgactgatctggaccaacccggtgac 1

tgactgatggatgatgatatattgac 1

tgactgcaaagtaggtgtactttgac 1

tgactgcagagtgcaaactgtgtgac 1

tgactgctagtgtttacccaaatgac 1

tgactggaaatgaaataatttgtgac 1

tgactggaactcttaaaggctatgac 1

tgactggccaatttaatgactatgac 1

tgactgggcctttcaatgttagtgac 1

tgactgggctaatgggccggtgtgac 1

tgactggtaatcagctgagcagtgac 1

tgactgtccattaaaaatttgatgac 1

tgactgtctattgtaccttttttgac 1

tgactgtgagttggtgtctttatgac 1

tgactgtttactacaacgaccttgac 1

tgacttaaaaggaagaatgatctgac 1

tgacttaactcagtacacatcctgac 1

tgacttaatgatgatacgactttgac 1

tgacttacacgcttcaaaacgttgac 1

tgacttacatgcccagacccggtgac 1

tgacttagatgattatatacattgac 1

tgacttagttttctcatgatgatgac 1

tgacttataggacatgtacacttgac 1

tgacttatcatcttcacggagttgac 1

tgacttatccaaaatagtttgatgac 1

tgacttatggattaggctaatttgac 1

tgacttcaatgcattagctatctgac 1

tgacttcagcaaatatgcaccatgac 1

tgacttcataggttcttagttctgac 1

tgacttcatatacagaatttaatgac 1

tgacttcatgttcagacgtccctgac 1

tgacttcggtgtttgaaatgtatgac 1

tgacttctaacttgactgtctttgac 1

tgacttctagaccactttttagtgac 1

tgacttctcaatttttctttattgac 1

tgacttcttattacagtcttcttgac 1

tgacttgaaaataaaatacatatgac 1

tgacttgaaagcatacaatctttgac 1

tgacttgaacgggtcgctcttatgac 1

tgacttgacccatagctttgcttgac 1

tgacttgactcattgcttgacttgac 1

tgacttgacttgactcattgcttgac 1

tgacttgattgctttctttttttgac 1

tgacttggcttaggtgcaagactgac 1

tgacttgggcgtcgagtggagctgac 1

tgacttggttggtaaaccgagttgac 1

tgacttgtagcgaatgaacacatgac 1

tgacttgtatagttgtattgcttgac 1

tgacttgtatgatcgttttgcctgac 1

tgacttgtcgatttcaatttgatgac 1

tgacttgtctcttctttgtcgttgac 1

tgacttgtgtacgaatgtaatatgac 1

tgacttgttctgcttccttgggtgac 1

tgactttacttgggactaatgatgac 1

tgactttagtttaggaggaccttgac 1

tgactttatgcatgtgaaaagatgac 1

tgactttatttctccaagtgcttgac 1

tgactttattttagcctaattttgac 1

tgactttcacaataccattcagtgac 1

tgactttctcgtagctgtaacatgac 1

tgactttgaaagtgtatgtacgtgac 1

tgactttggtaactaaatcatttgac 1

tgactttgtgcaggccagaagatgac 1

tgactttgttatatgatgatattgac 1

tgactttgtttttcgtaagctctgac 1

tgacttttaacattgccttgtttgac 1

tgacttttactagtctacaagatgac 1

tgacttttagatagagagccattgac 1

tgacttttagtttttgataaaatgac 1

tgacttttcataattaaagagatgac 1

tgacttttctagactgaataaatgac 1

tgacttttcttcttaacttgcatgac 1

tgacttttgacactcgttttcgtgac 1

tgacttttgaccattaatgaaatgac 1

tgacttttgtagtttttatttttgac 1

tgacttttgtgcatagaactagtgac 1

tgacttttgttgaactttgtagtgac 1

tgactttttatatgtttttcattgac 1

tgactttttctttttgtacatttgac 1

tgactttttgctgttgagtttctgac 1

tgactttttgtatcgatgattatgac 1

tgactttttgttgttaagacggtgac 1

tgacttttttaaaaccaaattctgac 1

19 : 349

tgacaaaaaaatagtggtatttttgac 1

tgacaaaaagacaagtttctaagtgac 1

tgacaaaaatatataaatagacatgac 1

tgacaaaatgagttctagaaagttgac 1

tgacaaaatgtcatgtgctgatgtgac 1

tgacaaaattctctgtaccattttgac 1

tgacaaactagtccaatggaatttgac 1

tgacaaagatgtgaaactgcaagtgac 1

tgacaaagcacgtgcgcctctcgtgac 1

tgacaaagttcaattaaaaaacttgac 1

tgacaaataaaagagagagagagtgac 1

tgacaaatacaagactccttttctgac 1

tgacaaatgttaaagcgatgatgtgac 1

tgacaaatttctattggtcgttgtgac 1

tgacaaatttggtttcaccaagatgac 1

tgacaacaacaaaaagtcatgtttgac 1

tgacaacaattttatttttctgttgac 1

tgacaaccgaaaaaatgatacattgac 1

tgacaacgatttgtggtggattgtgac 1

tgacaacggatgcactaaccatatgac 1

tgacaagaactggttgaatttgatgac 1

tgacaagacaaagagagagaggatgac 1

tgacaagacacaccgacttttattgac 1

tgacaagattttgttacactatctgac 1

tgacaagctccaaacaggaaaactgac 1

tgacaagtgaaaaacagaatggatgac 1

tgacaataaataatttttgatgttgac 1

tgacaataccaaataagtttttttgac 1

tgacaatacggtcatcattactctgac 1

tgacaatattctacataaataaatgac 1

tgacaatcactaagacatggagatgac 1

tgacaatcaggtgagagggcctttgac 1

tgacaatgaggctataatacagctgac 1

tgacaatggggtggtggctccggtgac 1

tgacaatggttcccaagttctcatgac 1

tgacaatgtagctgaaaaaaaactgac 2

tgacaattactcaattagtgacatgac 1

tgacaattatacaagatatggggtgac 1

tgacaattatatatatacacatttgac 1

tgacaattatttttctgccaccttgac 1

tgacaatttgccagcagtattcatgac 1

tgacaatttgtgtagaatcgttatgac 1

tgacacaaaatagttgtaatccatgac 1

tgacacaacgacgacgacgccattgac 1

tgacacaatatgtaattgttcaatgac 1

tgacacaccatacacgttatacttgac 1

tgacacacccagacagttatcaatgac 1

tgacacagacatatcatatttaatgac 1

tgacacataatcactttagcatttgac 1

tgacacatcatcacacaacaaagtgac 1

tgacacatcatcacacaagaaaatgac 2

tgacacatcatcactttagcatttgac 1

tgacacattatcacacaacaaaatgac 1

tgacacattatcatacaagaaaatgac 1

tgacacattcctttcgacgactatgac 1

tgacacattcgatcgcagggtcatgac 1

tgacacattcggtcgcaggatcatgac 1

tgacacattcttctttgtaacattgac 1

tgacacattggatgacgaaagtttgac 1

tgacaccaactgagaaaaccatatgac 1

tgacaccaatagtactcgcaagctgac 1

tgacacgtgtataaattgccacgtgac 1

tgacacgttgtagtcttgtaggatgac 1

tgacactaaagagtagaagcaattgac 1

tgacacttagagccagtgatagttgac 1

tgacacttcatcgaaaccaaccttgac 1

tgacacttttctccagagaattgtgac 1

tgacagaagctgtctgtcgcttttgac 2

tgacagaagtctatttccctcggtgac 1

tgacagaattttgtttttgttagtgac 1

tgacagactacttaaatccaagttgac 1

tgacagatcagttacaattatgctgac 1

tgacagatgctcaactgatccaatgac 1

tgacaggtttataacaatttgtgtgac 1

tgacagtaattggtatttgaaaatgac 1

tgacagtatcacagatttgcacttgac 1

tgacagtgatatgaccgttgagatgac 1

tgacagtgtatgatttggttacatgac 1

tgacataaaaaaattaaaaaatatgac 1

tgacataaaatccatttttttcttgac 1

tgacataaaatttaaaacaaatatgac 1

tgacataaaattttaaacaaatatgac 2

tgacataaatgtcgtgatatatttgac 1

tgacataagtcgtataaaaattgtgac 1

tgacataattaacctagaaaagctgac 1

tgacataattggtgaagaagatgtgac 1

tgacatagcaatgtcattttcattgac 1

tgacatatacagattagatttgttgac 1

tgacatatacatacgtgtatacatgac 1

tgacatatagactgattcaaagctgac 1

tgacatatcatcacacaagaaaatgac 1

tgacatatgtcatcttctttacatgac 1

tgacatattatgtattggccatttgac 1

tgacatattcccgcgtgcgtcattgac 1

tgacatattcggtcgcaggatcatgac 1

tgacatcaaatctcattattgtatgac 1

tgacatcactggccgaagaacgttgac 1

tgacatcattgttcagaaagacttgac 1

tgacatgaaaaataatatggttgtgac 1

tgacatgaaattaaagtggagtttgac 1

tgacatgaagagacaagtgcgattgac 1

tgacatgatcatgatgtcgaaaatgac 1

tgacatgcacagtaacgtgtaattgac 1

tgacatgctttttgtttttgtaatgac 1

tgacattaaataaggttcatgtttgac 1

tgacattaagtgagattcatgtttgac 1

tgacattataagctacacacttttgac 1

tgacattctccttatgggttctttgac 1

tgacattgacaatacgagctccttgac 1

tgacattggtgaatacagacggatgac 1

tgacattgtttttttttagactatgac 1

tgacatttatccttttctgtagatgac 1

tgacatttatgcccctaaggttttgac 1

tgacatttcaaagctgacttttttgac 1

tgacatttgacaattttttggtatgac 1

tgacattttctagaaaggttcagtgac 1

tgacatttttatcttgtcactgctgac 1

tgacatttttcccattaataaattgac 1

tgacatttttttttttgttcaaatgac 1

tgaccaaaaactaatgtttattttgac 1

tgaccaaagcaaaatcggatatttgac 1

tgaccaactacagttttaagttttgac 1

tgaccaactatataagtatgttatgac 1

tgaccaagaagattcttactatttgac 1

tgaccaagactatagtctatacgtgac 2

tgaccaagtctattcactgaaagtgac 1

tgaccaatgtttgtccgataatgtgac 1

tgaccacaccaaccacctagttttgac 1

tgaccacagctagtttgaacttttgac 1

tgaccacatggagaccgaaaggttgac 1

tgaccacgaccaggccatgaccatgac 1

tgaccactatttttttgttcttctgac 1

tgaccactccaccttggtgacgatgac 1

tgaccactcgctcccttatatggtgac 1

tgaccactgctgccccttgtttatgac 1

tgaccagaaaaaatgtcaatcgctgac 1

tgaccagaagatttggttgcttatgac 1

tgaccatagatacgtcatacgtgtgac 1

tgaccatcatattagtattatattgac 1

tgaccatgaattttgacccattttgac 1

tgaccatggttttcaataatatgtgac 1

tgaccattacaaccatgactcaatgac 1

tgacccaaaagccgaactcagattgac 1

tgacccaatctgactaccttaaatgac 1

tgacccacaaatagaaaaacacgtgac 1

tgacccatctcaatctcttgtcttgac 1

tgacccatctctttatccacatatgac 1

tgaccccaaactatatatgaaaatgac 1

tgaccccaaagaggttcgtgctttgac 1

tgaccccaacttaggataaattttgac 1

tgaccccagtcaaacaaaaatattgac 1

tgacccctatatagacacacatttgac 1

tgacccctttcccaaaattttattgac 1

tgacccgaccccgacagatcctctgac 1

tgacccggacgtgtacctaaaaatgac 1

tgaccctaatccacgtgggattttgac 1

tgaccctgagaacattaaaattatgac 1

tgaccctgggatcttttgagttttgac 1

tgacccttttatttgaataatcttgac 1

tgaccgaaaaataaaacaataagtgac 1

tgaccgaccaccattacgtatgttgac 1

tgaccgaccaccgcatcctcagttgac 1

tgaccgagaccctcacaagccattgac 1

tgaccgatataatgtatttcccgtgac 1

tgaccgattctttttcttcgatctgac 1

tgaccgcttttggtttcgggttatgac 1

tgaccggaatctcgagactcgtttgac 1

tgaccggcaatagtaagttaagttgac 1

tgaccggcgagtttatctctagctgac 1

tgaccggtaaatctcaattcaaatgac 1

tgaccgtgctaaagcatacctcctgac 1

tgaccgttaaagcgtggtaaacgtgac 1

tgacctaaaatgggtttaaactatgac 1

tgacctaatagaaaagattccactgac 1

tgacctaatcttatcctcaaaaatgac 1

tgacctagagcctatagctttgatgac 1

tgacctataagtattgattaaattgac 1

tgacctcacgcgttttttttttttgac 1

tgacctcatgaatgattttgtgctgac 1

tgacctctatagatttaggaaaatgac 1

tgacctgatccacaagttgaccatgac 1

tgacctggtcccattttcttaattgac 1

tgaccttaatatagtttttttgttgac 1

tgacctttacacaccacacatgttgac 1

tgacctttggtattttactattatgac 1

tgacgaaaaaaagattcccctcgtgac 1

tgacgaaacatgcctaaagtgtatgac 1

tgacgaaagaaaaaggctgtttgtgac 1

tgacgaaagcaagaaaagaatagtgac 1

tgacgaaagtgaaatagaatatatgac 1

tgacgaacacttcactgaaatgatgac 1

tgacgaagtaatatatataagattgac 1

tgacgaatgtagtagaactgtgatgac 1

tgacgaccagcctaaccaagacttgac 1

tgacgagacggcatgatattcgatgac 1

tgacgagaggaatggattccatatgac 1

tgacgatgcacaacacattctcttgac 1

tgacgatgctcgcggacagacagtgac 1

tgacgattgagtgctttcctttgtgac 1

tgacgattgcaacaaaaattatttgac 1

tgacgcaaaaataaaagtaaaaatgac 1

tgacgcacacacacatgtatgtatgac 1

tgacgcataagaattactatagctgac 1

tgacgcattgcatatgattagcatgac 1

tgacgccatctaagaaaataatatgac 1

tgacgcgtgtcgtttaccttatttgac 1

tgacgctaaaggtcaaaatattatgac 1

tgacgctaacgtcgttcagccaatgac 1

tgacgctccgtgtcttttttttatgac 1

tgacgcttctagtttagacgtcgtgac 1

tgacggaaaacgctaaaagaaagtgac 1

tgacggaaagaagacggaattgttgac 1

tgacggacttaaaacccaaattttgac 1

tgacggttaaaatccattgtttatgac 1

tgacgtaccaacagtgaatgatttgac 1

tgacgtaggaaattatatgttaatgac 1

tgacgtcacaaatcacaatgcactgac 1

tgacgtgaacttccattggtcagtgac 1

tgacgtgatttaaaaacgtataatgac 1

tgacgtgcggcgtaagtaagcagtgac 1

tgacgtgctcgacgttaaggagatgac 1

tgacgtggacacgtcgttgtcaatgac 1

tgacgtggcataatttaattgggtgac 1

tgacgtggcatcagccattatgttgac 1

tgacgtgggtaacgttgtgatggtgac 1

tgacgttgacgatggtaagattctgac 1

tgacgtttataagccaacgttcttgac 1

tgacgtttctgattggttaacgatgac 1

tgactaaaaaatcaccaaaaatctgac 1

tgactaaaactacaaacttttcttgac 1

tgactaaaatgacgtcttcttcttgac 1

tgactaaaattttaaattcaaattgac 1

tgactaaacaggatatatattgttgac 1

tgactaaagaaacacagtttttctgac 1

tgactaaatcatgtttgttttgttgac 1

tgactaaatttacagaagcagtttgac 1

tgactaagtattttatattgttttgac 1

tgactaagtccactccaatccagtgac 1

tgactaataagagaattattatttgac 1

tgactaataatattaagcctcactgac 1

tgactaatatgtcataattaatttgac 1

tgactaattgatgtcgagtggattgac 1

tgactaatttatgtcttaaattatgac 1

tgactacaacaccaagttggccatgac 1

tgactacagtaaatacaatcaattgac 2

tgactagactagaccactgcttttgac 1

tgactagatatgcacttggagactgac 1

tgactagctacatcccttgatgatgac 1

tgactaggattctattacgtagttgac 1

tgactaggattggtgtagtcacatgac 1

tgactagtaaatcgtgagtatattgac 1

tgactagtatgtgaggtgactgatgac 1

tgactatagatttgtgtgcattgtgac 2

tgactatcaacgaactccaaacgtgac 1

tgactatcaatttttttatctggtgac 1

tgactatgttgatgatcacatgatgac 1

tgactattgacattgaattttaatgac 1

tgactcaaacctaagaaaatctttgac 1

tgactcacgaagataaaacggcatgac 1

tgactcagaacgagcattaacggtgac 1

tgactcagtgagtttgtaaaatgtgac 1

tgactcatcaagctttctcaatttgac 1

tgactcatcagtgtcgtttggtgtgac 1

tgactccaaaaatattaaattaatgac 1

tgactccagatcagtctaaagcctgac 2

tgactccgcgtacaagctatcattgac 1

tgactccttcgctaagtccgacgtgac 1

tgactcgaacaagagtaggtacttgac 1

tgactcgattgattctcttagtatgac 1

tgactcgttgttatatctaaaaatgac 1

tgactctaaagtctgaataaagttgac 1

tgactctaatcaaataagaaaattgac 1

tgactctacgatgatagcttatttgac 1

tgactctattgatctagtatcgatgac 1

tgactcttaaatttctttttggttgac 1

tgactcttgaagatttggttttttgac 1

tgactcttgaagctttgtttttttgac 1

tgactcttttgtcacacatgcgttgac 1

tgactgaagatttttcatttgattgac 1

tgactgagaagaaagaaagaagttgac 1

tgactgagcaatactaattaagctgac 1

tgactgatacgctcgagctcgactgac 1

tgactgattcatctaggtgcagatgac 1

tgactgcagtaataaagatttattgac 1

tgactgctaattagatttatatttgac 1

tgactgttaaatacagggggagctgac 1

tgactgttttacataaaaagttctgac 1

tgacttaaattaaattatatttgtgac 1

tgacttaacaagaaaaatgtttatgac 1

tgacttaagccaaataacaaaattgac 1

tgacttaagccaaataataaaattgac 1

tgacttaatatttgaaagctccatgac 1

tgacttagatttgctaagtgcgttgac 1

tgacttagatttgtagatagtcttgac 1

tgacttataaattcattttttagtgac 1

tgacttattgaatgttattagcttgac 1

tgacttcaatgggatattttatttgac 1

tgacttcatgagctcgtcagctttgac 1

tgacttcccggatcatgtaaagatgac 1

tgacttcgtctccaagactcagctgac 1

tgacttctgacgtaataatccattgac 1

tgacttctggtgcgtgtatttattgac 1

tgacttgagtaacaactttgtaatgac 1

tgacttgataatgttttcagaattgac 1

tgacttgattcttgagttgattttgac 1

tgacttggtgagtgactaatttttgac 1

tgacttgtgggaactatattctgtgac 1

tgacttgttcgacccattctctttgac 1

tgacttgtttttgagtgatttcttgac 1

tgactttaaatctagcattttgctgac 1

tgactttacccttttgaggtctctgac 1

tgactttagcattataatgaaattgac 1

tgactttaggagaatttcaacagtgac 1

tgactttatttcaaaaacttgtttgac 1

tgactttcaggcaagttcttgtttgac 1

tgactttcttatttattgttgtttgac 1

tgactttctttcttatttaggcatgac 1

tgactttgagtatacattattattgac 1

tgactttgataaatttgccttcgtgac 1

tgactttggcaagtatgtggtgttgac 1

tgactttggcctttctgttctaatgac 3

tgactttggtaacttactgtgtgtgac 1

tgactttgtatttcaagttgaagtgac 1

tgactttgtgatggtgatggtgatgac 1

tgacttttaaattttatattatatgac 1

tgacttttaataaatgttaaaattgac 1

tgacttttagatttgtttcttaatgac 1

tgacttttcgctagtcttatgtttgac 2

tgacttttcttttatttctattttgac 1

tgacttttgggaactctgaactctgac 1

tgacttttgggttcatctccaattgac 1

tgacttttgttgttgtcaacacatgac 1

tgactttttatcttcattgtttatgac 2

tgactttttcagcttcatgaatatgac 1

tgacttttttaatctcagttagttgac 1

tgacttttttttgtagcagctggtgac 1

tgacttttttttttttttgattatgac 1

20 : 417

tgacaaaaaaacaaatggacctcgtgac 1

tgacaaaaaaataaaaaaaatcattgac 1

tgacaaaaaaatcataaaataggttgac 1

tgacaaaaacaaaattacatacagtgac 2

tgacaaaaattagtgaaactttactgac 1

tgacaaaaattggactttaagtggtgac 2

tgacaaaacaagtataattaatattgac 1

tgacaaaacctgtagattttctgatgac 1

tgacaaaactataaagtgaaaacttgac 1

tgacaaaagaaaaaaaaaaaaggttgac 2

tgacaaaaggtgttttcgtaaatttgac 1

tgacaaaataaaaatgtttgttgttgac 1

tgacaaaataatactatgttgaattgac 1

tgacaaacaaaatatatataattttgac 1

tgacaaactttagacttgcgttgatgac 1

tgacaaagcataatatatagcatatgac 1

tgacaaagcctgtgaactaaaaattgac 1

tgacaaaggtactggttgttttgatgac 1

tgacaaagtaattagcaaagttagtgac 2

tgacaaagtccttgcagcagctgctgac 1

tgacaaataagttggaggaagatttgac 1

tgacaaatacactgtcatgagtcatgac 1

tgacaaatcacgtacgattcactctgac 1

tgacaaatgaaacattcaagtacttgac 4

tgacaaatgcttctagactaatattgac 1

tgacaaatgtatgttccaatcatgtgac 1

tgacaaattcatataattgttttctgac 1

tgacaaattgattataacttccgttgac 1

tgacaaattgccttctgaaaatgttgac 1

tgacaaccaagtctgtgaactatgtgac 1

tgacaacgaagatggcgattgcgatgac 1

tgacaactcgtgttatatccaaaatgac 1

tgacaactttccattaaactaaaatgac 1

tgacaagaacgaacaatcctccattgac 1

tgacaagaagcttaatctagagaatgac 1

tgacaagacatgttacttgtttgatgac 1

tgacaagtaatcaaaagtattgtatgac 1

tgacaagtcataatcaaaatcagatgac 1

tgacaagtgggatacttgattctttgac 1

tgacaagttaggtataaagtgttttgac 1

tgacaataacgaagaaggtgacggtgac 1

tgacaataattatattgactagcatgac 1

tgacaatagcgatggtagtagtggtgac 1

tgacaatctaaattccattttatttgac 1

tgacaatctagttacatgcatatatgac 1

tgacaatctcaccctctggacttctgac 1

tgacaatgacattgtacatgcccttgac 1

tgacaatgactcagtccttttgtttgac 1

tgacaatgatgtcagcatgacacttgac 1

tgacaatgtcatgaatatttcccttgac 1

tgacaatgttcatcagtagttttgtgac 1

tgacaatgttggtgagagatttcatgac 1

tgacaattacatcgacgtgattggtgac 1

tgacaattatattgaactcggatgtgac 1

tgacaatttagagattaaaaaacatgac 1

tgacaattttagttataaactatgtgac 1

tgacacactagatttcagtttctttgac 1

tgacacatcatcacacaagaaaaatgac 1

tgacacatcatcacttttagcatttgac 1

tgacacatgtcatttcttgtgtaatgac 1

tgacacatttgatgaaaagagttgtgac 1

tgacaccatcacccctaggaaaaatgac 1

tgacacctttagcaactttcttcttgac 1

tgacacgcaataacgcgttttaactgac 1

tgacacggaagatgatgagagtgctgac 1

tgacacgtagattaagtgaggacgtgac 1

tgacacgtctgatgtttacttagatgac 1

tgacactactcatacatgcatacatgac 1

tgacactcacctacagtggttaattgac 1

tgacactgatggagactatcacattgac 1

tgacactgttctcgttgttgcaagtgac 1

tgacactttacccaattttaaccatgac 1

tgacagaacaatgtactttgggaatgac 1

tgacagaagccgcatgatagtgtatgac 1

tgacagaagctatgaaggttcgtttgac 1

tgacagagaaactttttgaaatgatgac 1

tgacagagacgaagaaatcttgcgtgac 1

tgacagagcgatggaagctttacgtgac 1

tgacagatctactagtatgtccaatgac 1

tgacagatttgttgatcatttgtttgac 1

tgacagcgttttaaatattagtactgac 1

tgacagctaaaataaagattgttgtgac 1

tgacagcttacctcattggaaatatgac 1

tgacagcttatttagtatggacggtgac 1

tgacaggaaatggagtccagtgagtgac 1

tgacaggacatcacagaccaaacatgac 1

tgacaggttagcaatttgtcacattgac 1

tgacagtaacaacaaaaatatacatgac 1

tgacagtcccatcaatatatatgctgac 1

tgacagtggtggcccccaccttactgac 1

tgacagtgtcaaaagagatttgtgtgac 1

tgacagtttcgtgacataaaacattgac 1

tgacataaaaaacaagaacaatgttgac 1

tgacataaatttagaaaggacccatgac 1

tgacataacagataaacaatgtagtgac 1

tgacataagatgcatttagggaattgac 1

tgacataatttttagatcataaggtgac 1

tgacatactgataaagtcattgtttgac 1

tgacatagaatgcacgcaattctatgac 1

tgacatagtgtgatttaacataattgac 1

tgacatatgatacttatgtgagtgtgac 1

tgacatcaaagattgaatattcgatgac 1

tgacatcaactagtctaagatgactgac 1

tgacatcaagcacacctcttctggtgac 1

tgacatcactaaaagacaaacctttgac 1

tgacatcccctctctggtttcatctgac 1

tgacatcgtctagtttccttcccatgac 1

tgacatctgtatggcaacttttcttgac 1

tgacatgaattttttgttcacttttgac 1

tgacatgatcaaacgccatcaacttgac 1

tgacatgcaaatgttttcatgtaatgac 1

tgacatgcaacattctcttcccaatgac 1

tgacatgggtgacgcgatccatattgac 1

tgacatggtgtctccggatcttgttgac 1

tgacatgtaaagaacatacattaatgac 1

tgacatgttatctgaaaaattatatgac 1

tgacatgttctttgtggacagccttgac 1

tgacattaatggttctgatatatgtgac 1

tgacattagaaaccaaaccaaatctgac 1

tgacattatcaagtcaattcctggtgac 1

tgacattatcgttgtttgtgtgtgtgac 2

tgacattattattacagtaataattgac 1

tgacattccgatccgtttacattttgac 1

tgacattccggtattggtgggagatgac 1

tgacattgaggctatggtaactcttgac 1

tgacattgatggaatcaagccatttgac 1

tgacattggactacttcactaccatgac 1

tgacatttactctattaggaactctgac 1

tgacatttcttgaaaagcacgcagtgac 1

tgacattttgtttcttaatattactgac 1

tgacatttttgcatgtggtggtggtgac 1

tgacatttttttataacaaaatattgac 1

tgaccaaaaaaggctgcaaaaaggtgac 1

tgaccaaaaacctaccactttttatgac 1

tgaccaaaagagcggtgaagacgctgac 1

tgaccaaaaggaaaaaaatcttggtgac 1

tgaccaaaaggaatatgtgactcgtgac 1

tgaccaaacaattaggtatattcgtgac 1

tgaccaaacacaaaagctctcttatgac 1

tgaccaaacgtatctgttgactattgac 1

tgaccaaagaaaaaagaggagtgatgac 1

tgaccaaagacatcaaccacagtttgac 2

tgaccaaataaataaaaactaccttgac 1

tgaccaaataacgagtgtcttgcgtgac 1

tgaccaaatctctctggaggaaggtgac 1

tgaccaacatgcaaatagtcaaaatgac 1

tgaccaaccgacaataagaacagctgac 1

tgaccaactagtcaaacccgtttctgac 1

tgaccaagaaatccaatgagacggtgac 1

tgaccaataaaaaagatttagaaatgac 1

tgaccaatattactgaaatccaaatgac 1

tgaccaatgaaaactcgtgaagtctgac 1

tgaccaatttatataaaatgaccatgac 1

tgaccacaccaaatggtcgatttgtgac 1

tgaccacctctttaactgcgaccatgac 1

tgaccactcaagatcaaaacttattgac 1

tgaccactttttcgtctccatgtatgac 1

tgaccagctcaatgttgcattcattgac 1

tgaccatacgaaccattttccctctgac 1

tgaccatccagagtgcgaggcacttgac 1

tgaccatctgtagtgcctccacactgac 1

tgaccatgaggaagaaatctacgatgac 1

tgaccatggagattgatgagccgatgac 1

tgaccatgttgaactaattatttatgac 1

tgaccattaaactttagaaaaaaatgac 1

tgaccattagggacttagaaaaactgac 1

tgaccattgataaaagacatgaaatgac 1

tgacccaatcgttgtggaattttgtgac 1

tgacccacaaaatgtgagagaatgtgac 1

tgacccacgctctctctcacacgctgac 1

tgacccactaatcaaatggttcattgac 1

tgacccacttttaatttcaaattttgac 1

tgacccataaattttgacccattttgac 1

tgacccataaattttgatccattttgac 1

tgacccatattcgagtttcaatgatgac 1

tgacccatgaaacttgatccattttgac 1

tgacccatgaattttgacccattttgac 9

tgacccatgaattttgacctattttgac 1

tgacccgaattaaacaatccgacttgac 1

tgacccgcataagggcattaggcatgac 1

tgacccgccaaatttcggattatttgac 1

tgaccctcgtcgtctaaaaaccggtgac 1

tgacccttagttaatttctagttttgac 1

tgacccttattgtttcttcacttttgac 1

tgacccttttattttcctattttatgac 1

tgaccgaaataagaacaaaatttatgac 1

tgaccgaaattcttctgtgctaattgac 1

tgaccgagttctttaaggctcctatgac 1

tgaccgcaatactaaaaaaacaattgac 1

tgaccgccgagaaggccttcgtagtgac 1

tgaccggagcaagttaaatctcgttgac 2

tgaccgtaaaatctagttccaaagtgac 1

tgaccgtaattttctttgttttcttgac 1

tgaccgtttcttttgcgtcctcattgac 1

tgacctaacaaaagaacctatatatgac 1

tgacctaacgttttctccaacttgtgac 1

tgacctaaggaaagatgctttgggtgac 1

tgacctaattttcgttttgttttttgac 1

tgacctacaaaggtctcgtctcactgac 1

tgacctacaataaaactcaattggtgac 1

tgacctagaagtaaattacaattttgac 1

tgacctagaagtggtctttggatgtgac 1

tgacctagacggtggtggtggtggtgac 1

tgacctatgtgaacaagaaaatgctgac 1

tgacctccaaggcagttctacaaatgac 1

tgacctctactctcgcacggtttgtgac 1

tgacctctcctttcctgataatgatgac 1

tgacctgaaatttttggggaacagtgac 1

tgacctgattatgattacgtgaaatgac 1

tgacctgcttgctcttcctgctgttgac 1

tgacctgtggatctccagaaattatgac 1

tgacctgttaatggaagacaactatgac 1

tgaccttaccttaatctaataagttgac 1

tgaccttatccctttctgttttgttgac 1

tgaccttccctcttgcacatttaatgac 1

tgaccttgcatcatgtaatgttgttgac 1

tgaccttgtatgtagccatggctgtgac 1

tgaccttgtttattatatgtgttttgac 1

tgacctttttaaaaggcactgttgtgac 1

tgacgaaaaaagaaattttaaaagtgac 1

tgacgaaattatgacagaaaaaaatgac 1

tgacgaaattgagatcacgaaatatgac 1

tgacgaacacaagtcaaagaccattgac 1

tgacgaaggactgttataccactatgac 1

tgacgaatgtcaacaatctttagttgac 1

tgacgacaaagaagctaaccgtcttgac 1

tgacgaccttgcaagtgatgattatgac 1

tgacgacgaagtttccgttgacgttgac 1

tgacgagatacgttgaaatcagcttgac 1

tgacgagcgacgagtgacgagcgatgac 1

tgacgagtgaatggcctttttttgtgac 1

tgacgataaaatgtcaaaggaatgtgac 1

tgacgatagcgataatggtggggttgac 1

tgacgatccttgtagaaacctttttgac 1

tgacgatctcttcaagttagatattgac 1

tgacgatgatgacggtgaggatgatgac 1

tgacgatgggacaatgaagtatggtgac 1

tgacgatgggccatggtacatttatgac 1

tgacgatttttggtctcgttttggtgac 2

tgacgcacgagccacgttctacggtgac 1

tgacgcaggatctcccacttggggtgac 1

tgacgcatctgtatcttcaatcaatgac 1

tgacgccatctgcgatcgttcccatgac 1

tgacgccgacactgtgagaattgatgac 1

tgacgccggtcgttcatcatcggctgac 1

tgacgcctaactacccgaaatcaatgac 1

tgacgcgcttgaaggcgcgtgtgatgac 1

tgacgctaaatgccattgtgatattgac 1

tgacgcttctcatccatgaaggtttgac 1

tgacggatgagattcttcttgtgatgac 1

tgacggattactctatgcctgggatgac 1

tgacggcgacagccgtggtggtggtgac 1

tgacgggagctgtaacagtgacggtgac 1

tgacggtaaaatctacgtggtgactgac 1

tgacggtaaaggtggtggtggtggtgac 1

tgacggtaatgatggtggtgatgatgac 1

tgacggtggaggagtagtagtaggtgac 1

tgacggttatactaacgacttttatgac 1

tgacggtttgtcgtcttgtttatctgac 1

tgacgtaaaagctacgtattattatgac 1

tgacgtaaatgaataatttggaaatgac 1

tgacgtacaactaaatctggaccctgac 1

tgacgtagaagaagttcaaatgattgac 1

tgacgtagaagctatctggtttcttgac 1

tgacgtataaacgtaatgatcacttgac 1

tgacgtatctaaaaatttgctttatgac 1

tgacgtattcgttagatctgtatatgac 1

tgacgtcaaaagtctccctatttatgac 1

tgacgtcaccgtgtgatacaagattgac 1

tgacgtcatctgtttcggttttgttgac 1

tgacgtggcgaaaaggtactggtatgac 1

tgacgtgtgaaattgtgtgaaagttgac 1

tgacgtgtttttaaagctctttactgac 1

tgacgttgctgataacatcctgcttgac 1

tgacgtttctgaatattcgagatgtgac 1

tgactaaaaagtaaacgtaagtgttgac 1

tgactaaaaattgttttctgtagatgac 1

tgactaaacacactcaattataattgac 1

tgactaaacttgaaccgattggtttgac 1

tgactaaagaggaaatgaatgttttgac 1

tgactaaagtcagttttaattctatgac 1

tgactaaatcattcttaagaatcttgac 1

tgactaaccgtacaattcaggctttgac 1

tgactaacctagtctatctcacggtgac 1

tgactaagtacttttttttttaattgac 1

tgactaataaacagaggatcatggtgac 1

tgactaatagttttacaagattagtgac 1

tgactaatgaaattcattattcattgac 1

tgactaattctatatttcttatactgac 1

tgactaatttgagtaaatattaattgac 1

tgactacagatttaataagcacattgac 1

tgactacgaaatcccaagacagtttgac 1

tgactagaaaatatacaaataatatgac 1

tgactagaataatttttttagtgatgac 1

tgactagataaatttccaaagacatgac 1

tgactataaaaataatattataaatgac 1

tgactataagaatagtatagacgatgac 1

tgactatataaactagctagagaatgac 1

tgactatataatttgtaacaaatttgac 1

tgactatgactgtgaccgtgactgtgac 1

tgactattattgtaactttacaaatgac 1

tgactattattgttcaaaggacaatgac 1

tgactattcatccattatcacaattgac 1

tgactatttgagataattccatggtgac 1

tgactatttgatgtcatttttaaatgac 1

tgactcaaatataggttattttcatgac 1

tgactcaactttttcggtcttcgttgac 1

tgactcaattttggttgtggaagatgac 1

tgactcacccaaatacatttaatttgac 1

tgactcatatattcaaaattgatgtgac 1

tgactcatgctctaaataagttattgac 1

tgactccaagtgagtatttaaatatgac 1

tgactccgtgcataaactacaagttgac 1

tgactcgtatatgactcatcatgctgac 1

tgactcgtattatgctatatagagtgac 1

tgactcgtttaaactataggtagatgac 1

tgactctggtttccctctgcatactgac 1

tgactcttgcaattttgtttaaattgac 1

tgactctttcaaaatctggttcattgac 1

tgactgaagctaccaaaaactcggtgac 1

tgactgaccacattggagcttaagtgac 1

tgactgagagaccggcaatggctttgac 1

tgactgagtaggaaaaaggaatagtgac 1

tgactgatgacgaaaaagtaaatatgac 1

tgactgatttacaaagctcaaagatgac 1

tgactgatttatatttttttaaattgac 1

tgactgcacagtctcgggcaccgttgac 1

tgactgcggtaatcagcttaagcatgac 1

tgactgctccctatgtgataaatttgac 1

tgactggaaacctccggccgcttgtgac 1

tgactggataaagaaacgattgggtgac 1

tgactggcagtcaggcccggcccatgac 1

tgactgggcatgtgcactgcaacgtgac 1

tgactgtaatttaatatccgattttgac 1

tgactgtattaccacatgtatcgctgac 1

tgactgtgacggctatggtggtggtgac 1

tgactgttcatgccacacttatattgac 1

tgactgtttctattgtcgactgtttgac 1

tgacttaaataacctttttagtaatgac 1

tgacttaacataaaatggaaagattgac 1

tgacttaacgaggaaaatgatgtttgac 1

tgacttacggagtcacgtgagacgtgac 1

tgacttactacagcgatcacaacatgac 1

tgacttagatggaaatgatgatgatgac 1

tgacttataagagtaaaaaagctttgac 1

tgacttatctaaaccaagttttaatgac 1

tgacttatctcatgaaaagccaattgac 1

tgacttatggttatgggtgatggatgac 1

tgacttattgaagtacgaaattattgac 1

tgacttatttttgcctagtttcattgac 1

tgacttcaccaccaacaccatcactgac 1

tgacttcatcaacgtacaaaattgtgac 1

tgacttccaagatggaaaactttttgac 1

tgacttcctctcagcatattacaatgac 1

tgacttcgaagagtctcatagagatgac 1

tgacttctcatgttatcaacctcctgac 1

tgacttctccagatacaaaacaattgac 1

tgacttgatccacacctgaagccttgac 1

tgacttgatgtggtcctacttcgatgac 1

tgacttgcagatcacgaaaagtgatgac 1

tgacttgccttgtcctttcttccttgac 1

tgacttgccttttttaataattcttgac 1

tgacttgctgaccgggacacaatctgac 1

tgacttgcttttattgttaaactatgac 1

tgacttggacttctgagttagacttgac 1

tgacttgggcagattcttgaagtatgac 1

tgacttgtcttttttaataattcttgac 2

tgactttaattatatatggacaattgac 1

tgactttagtatttgtttagggagtgac 1

tgactttagtgggtgaatttcttgtgac 1

tgactttatgcaaatttgaactaatgac 1

tgactttatgctagcttcttccggtgac 1

tgactttattagtattttaaaaaatgac 1

tgactttcccccattttccttggatgac 1

tgactttccggttccaaaagctattgac 1

tgactttccttcttactctgtcgttgac 1

tgactttctcgttggcattagttgtgac 1

tgactttctgcccaatggaagccttgac 1

tgactttctttaaacgcttggaattgac 1

tgactttgaagacgatgaagattctgac 1

tgactttgaccagctccttttcattgac 1

tgactttgagtttgatgttgaccatgac 1

tgactttgtacccttgaccttccgtgac 1

tgactttgtcaaacagaaacagagtgac 1

tgactttgttattcctaaattatgtgac 1

tgacttttaaaatcgtttttctcctgac 1

tgacttttaaacaataagctataatgac 1

tgacttttagaggatgaactaacatgac 1

tgacttttatctacgtagcgtctttgac 1

tgacttttcaaaagtatgcgacgttgac 1

tgacttttcatgaattatattagatgac 1

tgacttttgataaaatttcccctttgac 1

tgacttttgtttcttgcgtccttttgac 1

tgactttttaaaaagtaaagtaactgac 1

tgactttttcgttttgatctgttttgac 1

tgactttttgttcattttatcacatgac 1

tgacttttttttcttctctttgattgac 1

21 : 379

tgacaaaaacaagttaaacaaccaatgac 1

tgacaaaaatgaccaagccaaagagtgac 1

tgacaaaacaaacactgttcgaaactgac 1

tgacaaaacacacgtgaaaaatgaatgac 1

tgacaaaacagaacatacaaaaaagtgac 1

tgacaaaacatatatttatatattctgac 1

tgacaaaagaaggcagattgagacttgac 1

tgacaaaagagtacagagagattgatgac 1

tgacaaaagcaaatgtgattactgatgac 1

tgacaaaaggttaacaaatgtcatttgac 1

tgacaaaagtaaaggaagaaactcgtgac 1

tgacaaaataaaataatattgtaaatgac 1

tgacaaaatcattaatatattttagtgac 1

tgacaaaatcctaaaaataattatatgac 1

tgacaaaatgatgtttaattccacgtgac 1

tgacaaaattatgtttaatttcacgtgac 1

tgacaaacaaacaaaaagtctaatatgac 1

tgacaaacgatataaataaaagttttgac 1

tgacaaaggaataagccaaataagttgac 1

tgacaaaggagtcagaaaggacacttgac 1

tgacaaaggtattaattctataaattgac 1

tgacaaagttgaaaagaagaaatattgac 1

tgacaaagttgcaatttgataatggtgac 1

tgacaaataaaaaggaactttttggtgac 1

tgacaaatctattgatcttatattttgac 1

tgacaaattagtacttattacattttgac 1

tgacaaattatttaggaatattatgtgac 1

tgacaaatttacatccttttaaatatgac 1

tgacaacagttagaagacccaaacatgac 1

tgacaacatgatgactcgcataatatgac 1

tgacaacgaaaagtcgtagtatcagtgac 1

tgacaactcataaacgttaataacatgac 1

tgacaactgacaagcatatattagatgac 1

tgacaactggtttccgtgtacggaatgac 1

tgacaagaagatgatagaatcaaaatgac 1

tgacaagatcaaacttctctctgactgac 1

tgacaagattgattttgcctctccatgac 1

tgacaagcttctcttggagtttagttgac 1

tgacaagctttgatagtatatttaatgac 1

tgacaagtttcaaaagtttttacaatgac 1

tgacaataacaaaagatatattttttgac 1

tgacaatcgactttaaaaattatcatgac 1

tgacaatctttatcatcgttgactttgac 1

tgacaatggagtatcatggacgtggtgac 1

tgacaatgtgttattcaactgataatgac 1

tgacaattcaggggcatagaatacgtgac 1

tgacacaaacaaaactcatcctttatgac 1

tgacacaaccaaggaagatgattcatgac 1

tgacacaagtgagatcacagattgttgac 1

tgacacactactactctttccgtattgac 2

tgacacatgccaatctatctactgctgac 1

tgacacatgtaaacacctgatcgtttgac 2

tgacacatgtaaacatctaatcgtttgac 2

tgacacatgtaaacatctgatcgtttgac 4

tgacaccaaatgtttttagaattagtgac 1

tgacaccaatagctccttgcttccgtgac 1

tgacacccatggtcaatacattggttgac 1

tgacacccgtggtcaatacattggttgac 1

tgacacctgttttaagtcctaattttgac 1

tgacacgatcacggtattactaatttgac 1

tgacacgcgtcctcgaagtccaacttgac 1

tgacactaggcccacgtcctctgtctgac 1

tgacactagtttacatttgacaaagtgac 1

tgacactatttgcaatagtaaaatttgac 1

tgacagactttttagaagatagtgatgac 1

tgacagagctaaagttaataatgggtgac 1

tgacagataaatgcggggatgcttgtgac 2

tgacagattctgtttttaacaccgctgac 1

tgacagatttacccttcgtggaacttgac 1

tgacagatttgttcaaaaatctttatgac 1

tgacagcgccatatataatgaagtgtgac 1

tgacaggaggaaagagaaatatatatgac 1

tgacaggtagttcttaatttaataatgac 1

tgacaggtcataaagaaatcaattctgac 1

tgacaggtgtcaatatatgattagttgac 1

tgacagtatcaaaaagtccaagtaatgac 1

tgacagtttaagatcataatgctactgac 1

tgacataataaagaaaaatatatattgac 1

tgacataattctgcctagtgtggtatgac 1

tgacatacacgttatgatatatgcatgac 1

tgacatagaattctaaacatattagtgac 1

tgacatagtaggcccatattctttttgac 1

tgacatatcaagccatttaaatgtatgac 1

tgacatattaatacgtcgccgaaattgac 1

tgacatatttgagttcaacattgtgtgac 1

tgacatattttaacattcctaaatatgac 1

tgacatcaactcagctgattctagttgac 1

tgacatcagagcgtcttcaggaatatgac 2

tgacatcagtcatttaacctaaatatgac 1

tgacatcatctccacaaagtctatatgac 1

tgacatctataatttagagttatcttgac 1

tgacatctcctaaggttaaatcaagtgac 1

tgacatctggttccaccatcgaccgtgac 1

tgacatgaacttttgacgcctaacctgac 1

tgacatgaagttcaatacgatttactgac 1

tgacatgaattaatgataaataatatgac 1

tgacatgagaaaaaaaaagaccacttgac 1

tgacatgagtaaatctgttatgctttgac 3

tgacatgcaacgagtctccctgctgtgac 1

tgacatgcaacgggtctccctgctgtgac 1

tgacatgcaacgggtctcgctgctgtgac 2

tgacatgcaagcgacctaatagttttgac 1

tgacatgtgatagaatcagagaagatgac 1

tgacatgtggcatcattctgatcgatgac 1

tgacatgtgttgcaccgaaaaagagtgac 1

tgacatgttccttagccttttgacttgac 1

tgacattaacaagcaggcgtccagctgac 1

tgacattacattacattaaagtatttgac 2

tgacattatatattaaatattatcatgac 1

tgacattatatcatctatacatgaatgac 1

tgacattatttgaatcacaaagagttgac 1

tgacattgaagaactttgaaggtcttgac 1

tgacattgcagattattaacattgctgac 1

tgacattgccttaaccacaacctattgac 1

tgacatttctaaggacagtcaaatatgac 1

tgacatttgataccattaggagtcatgac 1

tgacattttcttcttttaatttacatgac 1

tgacatttttagattttgctttttttgac 1

tgacatttttatttttcaatgcgcttgac 1

tgacatttttcacgtgatttgcttgtgac 1

tgacattttttttttataatctctttgac 1

tgaccaaaacaaatgtattgagtattgac 1

tgaccaaaacttattaggtcagccgtgac 1

tgaccaaaagctatttgagttattttgac 1

tgaccaaaatggtaattttatcaaatgac 1

tgaccaaatattctcagccattcgttgac 1

tgaccaacgtaaacaaaaaagatgttgac 1

tgaccaattcaacgtctaaaatttgtgac 1

tgaccaatttaagactttgaacatttgac 1

tgaccaccactccttccgatcatcatgac 1

tgaccaccgttgttgattcagtcattgac 1

tgaccactgcggttgcggttgcgtctgac 1

tgaccactttgttgttgcgtgaaaatgac 1

tgaccagcatagaccttttacatagtgac 1

tgaccataactaaagtaagcgattttgac 1

tgaccataataaccgatcctagtcatgac 1

tgaccatagataggcagcgagccagtgac 1

tgaccatatatacaagtactaatggtgac 1

tgaccatcattccaattccaaaatttgac 1

tgaccatcgagcagtgtttttttggtgac 1

tgaccatctaaagtggtccccgtgatgac 1

tgaccatcttgcgtagagctggctgtgac 1

tgaccatggctcgggatgaagatgatgac 1

tgaccatgtaaacaggaacaacacatgac 1

tgaccatttaaccaaatttaaaagttgac 1

tgaccattttatttaatgtagtcgttgac 1

tgacccaaaaaaaaaaaaaagagtttgac 1

tgacccacaagccacaacaattttatgac 1

tgacccatcagattcttagtccatgtgac 1

tgacccgaatacccattttaaaatctgac 1

tgaccgaaataactaagtccttcgttgac 1

tgaccgatttcctttggtaaatctctgac 1

tgaccgccgatcaaattctgttttctgac 2

tgaccggaggtcaaagtaatttaattgac 1

tgaccgtagtgaaactggtgctctttgac 1

tgacctaaaactgcatatgaataattgac 1

tgacctaaagtaaacgagtacaatgtgac 1

tgacctaaatattagattgtgggcttgac 1

tgacctaagtaactcgaatataaattgac 1

tgacctaattctccggaaattaagttgac 1

tgacctatagaaacccaaagtaacttgac 1

tgacctccatgatggacctttgctttgac 1

tgacctctcaagatttaggctaacctgac 1

tgacctctctctatatacagaagcttgac 1

tgacctctggaaaacgaaaggtgggtgac 1

tgacctgaacatatgaaagtatgaatgac 1

tgacctgctttcatttatgagtctctgac 1

tgacctggctctgtgttcatacatgtgac 1

tgacctggtatggttctagatcttctgac 1

tgaccttaaaatcaaattttactaatgac 1

tgaccttaaccaaatgagcaaatattgac 1

tgaccttgctaaaacgaaaggtatatgac 1

tgaccttgtttatagtaaggactaatgac 1

tgacctttcggcgggaaattcgaaatgac 1

tgaccttttgtagtctttagaaatttgac 1

tgacgaaaattgtagaaaaaaggagtgac 1

tgacgaaatatattaaatgttattgtgac 1

tgacgaacatatgggccatagcccgtgac 1

tgacgaagacgtgactttcttattttgac 1

tgacgaagcagatgacggcaagacgtgac 1

tgacgacaactcaatggaagaattgtgac 1

tgacgacaggggaagaaaacatgagtgac 1

tgacgacatacataacacatttcactgac 1

tgacgaccctttttgttagtcatattgac 1

tgacgactcaacagaaattggctaatgac 1

tgacgagatacacacagagatgagatgac 1

tgacgagccttggtgatatagtaattgac 1

tgacgagctggttagacgggagagctgac 5

tgacgagtgcttaccatccccagagtgac 1

tgacgatagaagatattaatcgagttgac 1

tgacgatagaataagattgtaaaaatgac 2

tgacgatgataacaattatattttctgac 1

tgacgatgcgtgatgaccagcacactgac 1

tgacgattgagacaaataattattttgac 1

tgacgcaaaatttcgcgatcatgcatgac 1

tgacgcccggatgtcatagtctaaatgac 1

tgacgccttccatgatccatcaaactgac 1

tgacgcgactgattaatgttatgaatgac 1

tgacgctactgcccttttaaggctttgac 1

tgacgctgaggagtgtcaaaacgcttgac 1

tgacgcttcttcatgacttcatcactgac 1

tgacggaaaaatgtgaaatcttttttgac 1

tgacggaagctgccactagagcatctgac 1

tgacggaggcgggtccacgggaaggtgac 1

tgacggatgaagttccggcgagtagtgac 1

tgacggcagttgagatactcaatgatgac 1

tgacggctccaccggggaatagatctgac 1

tgacggtcacgccgacactttgttttgac 1

tgacggtccagttagaattgggctttgac 1

tgacggtgaccgattctgatatatgtgac 1

tgacggtttggtatagcatatagtttgac 1

tgacgtaaacatttctttcaatatttgac 1

tgacgtaatctcgttcctcacattatgac 1

tgacgtagaggtcaagtgtaaaagttgac 1

tgacgtagctcgaaagagtcgaaggtgac 1

tgacgtatgacgtctgtttattggttgac 1

tgacgtatttgtgcgtttctcatagtgac 1

tgacgtatttttacctttgtttttttgac 1

tgacgtcaagtaaaagctcgtttgatgac 1

tgacgtcaatcggtttcttactaaatgac 1

tgacgtcaatttcttcacttctctttgac 1

tgacgtcaccagtctcaattttaggtgac 1

tgacgtcattaacaaaatcatttaatgac 1

tgacgtcgtccgacttaaataccgatgac 1

tgacgtcttacttaatttaatgagctgac 1

tgacgtgaatgaatcgtcaaggcagtgac 1

tgacgtgacagaatgcatatggtactgac 1

tgacgtgagattacgattgacccattgac 1

tgacgtggaagtgagtgacgtcatctgac 1

tgacgtggccaaaagtgtcaagatttgac 1

tgacgttttataatgtacgaagttgtgac 1

tgacgttttttcacgtaatgctaattgac 1

tgactaaaaaatgatgtaattaaattgac 1

tgactaaaaatattaattagtggaatgac 1

tgactaaaactttcatcagttacattgac 1

tgactaaaccccttgtcaaaacagctgac 1

tgactaaattgggtcccaattattttgac 1

tgactaacacgaagaatgaaaaaagtgac 1

tgactaaggcgattggttgacaaaatgac 1

tgactaagtaaggtacatggtgccatgac 1

tgactaataagctggaaagcaaaactgac 1

tgactaatctatgtgctcaatcttgtgac 1

tgactaatctgactcaacttgtatttgac 1

tgactaatgtcgtttctgttgtagatgac 1

tgactaatgtttcaccaaaaaaaaatgac 1

tgactaattatattgatctcattcatgac 1

tgactacacaatttgatgcactaagtgac 1

tgactacagctaaggagttctatactgac 1

tgactacatctacaacactcaaagatgac 1

tgactacatgcaattttcaagacattgac 1

tgactacattattacaagatatacatgac 1

tgactactgactatttagacaattttgac 1

tgactaggcgacaacaagagacaagtgac 1

tgactaggtgaggaattggacgaactgac 1

tgactagtcaacgaaaatgagtaaatgac 1

tgactataaaagacttcaaatgttttgac 1

tgactataaaatgtcccatattcagtgac 1

tgactatagaagtagtaagtaactttgac 1

tgactatataactgggagttatagttgac 1

tgactatatattaattgggaaactttgac 1

tgactatcacctccaattccattgatgac 1

tgactatctaactttctaagttttgtgac 1

tgactatgatgattagtggctagactgac 1

tgactatggatacgatttttgaaaatgac 1

tgactattaatttggtcttcctttttgac 1

tgactattcaatataaggtaataaatgac 1

tgactattgactaatataccaaatgtgac 1

tgactatttttataaccggcgggtctgac 1

tgactcaacctataaggaaagtgcatgac 1

tgactcacaagtacaactcaagaattgac 1

tgactcacaataacgttgagattaatgac 1

tgactcacacggtctatgctcacaatgac 1

tgactcaccagtcacccctattatctgac 1

tgactcagaaaatcattataccgcatgac 1

tgactcaggcccatttaaagaatgatgac 1

tgactcaggttagagtgaagttcactgac 1

tgactcatatccaatttctaatatgtgac 1

tgactcatatggtttggtagagacttgac 1

tgactcatcagccgacccttaacaatgac 1

tgactcccaaatagtaataaatatatgac 1

tgactcccaagttcccaattaaacctgac 1

tgactccgaagcttcatcatcgtgatgac 2

tgactcctagcgaactaaaagacaatgac 1

tgactcgggtttctgagatcaatcttgac 1

tgactcgtgactcagtgacgactcatgac 1

tgactcgttatatatcctaaccatgtgac 1

tgactctatataccttaagaaaatatgac 1

tgactctccacttctcattgctcaatgac 1

tgactctgaaacgttctttgatttttgac 1

tgactctggaagtcctttttgttgttgac 1

tgactctgttttttccatcggaatttgac 1

tgactctttcaaaaaagacaacctttgac 1

tgactgaaaagagtaaaaaattgaatgac 1

tgactgaaatttatggagcattgtatgac 1

tgactgaaccggtggctgattaggatgac 1

tgactgaagtagttagtatgttttgtgac 1

tgactgaatagtatctgaacactaatgac 1

tgactgacctaatttaacttcttactgac 1

tgactgagacttgcacacgcatcggtgac 1

tgactgagctgaacaaacctttctttgac 1

tgactgatacaaaaataaaattaaatgac 1

tgactgatgctatcacatgttttcttgac 1

tgactggctgataacaacaacttcatgac 1

tgactggtccaagtcaaggttattatgac 1

tgactggtctttgtttgaaactttttgac 1

tgactggtttgccctgtgtttgaattgac 1

tgactgtaaaaaacgttacgatttgtgac 1

tgactgtctaggatcaatagagacctgac 1

tgactgtctcagtctgaaagtcacgtgac 1

tgactgtgggatgatgaattggatttgac 1

tgactgttaaagtacatatatgtaatgac 1

tgactgttgagctccattgtgaacctgac 1

tgactgtttttaatattctctaaaatgac 1

tgacttaacattctgcttaggcctttgac 3

tgacttaatatatataagtgtaaaatgac 1

tgacttaccaagaaaaggaccaaattgac 1

tgacttaccgatgaattttttttactgac 1

tgacttagaaatatatatatattagtgac 2

tgacttagctcaccatttcataaaatgac 1

tgacttagtcctcaagcatagtgggtgac 1

tgacttagtgaatgttaattacagttgac 2

tgacttattatttttattagtttattgac 1

tgacttatttgttgttaagattgtgtgac 1

tgacttattttctccccaatttagttgac 1

tgacttcactgacgtgtgaatgtgatgac 1

tgacttcatcaaccgctcctccgcttgac 1

tgacttcgtgcaatatcataaaatttgac 1

tgacttcgtttttttcattggagtttgac 1

tgacttctaacaatcattattcaaatgac 1

tgacttctattattctataaactattgac 1

tgacttctgagaatttttgaaagtctgac 1

tgacttcttcggcggcgatcagatctgac 1

tgacttcttcgtctcgaggcaaacttgac 1

tgacttctttcaaatcgatgtgccttgac 1

tgacttgaggtcttgtttactttcatgac 1

tgacttgattctcttcgatcttctgtgac 1

tgacttgcacttgaatctattgctttgac 1

tgacttggcaggtacctttcttttatgac 1

tgacttggcttattggtctctttcttgac 1

tgacttggtaaaacaacacgcaccttgac 1

tgacttgtgagaatctgagtcctaatgac 1

tgacttgttaaaaaattattcattttgac 1

tgacttgtttattgaataaacgcaatgac 1

tgactttaacgcaagtaaaggacgttgac 1

tgactttacggaaagtttctatatctgac 1

tgactttatttcttgttgacaaaattgac 1

tgactttcacttgtcctttgatccttgac 1

tgactttgactagagtcaacaaaaatgac 1

tgactttggacgatatcaacaactttgac 1

tgactttgggtcaactaactttccttgac 1

tgactttggttgcatttgaatctcgtgac 1

tgactttgttgcaatattttatgcatgac 1

tgacttttgcttaatatatataatttgac 1

tgactttttgaagcatttctcgaattgac 1

tgactttttttcttatgtcgttgtttgac 1

tgactttttttttgaagttaacagatgac 1

22 : 376

tgacaaaaaaaaagaagataaaaatttgac 1

tgacaaaaaaattagatgaaaagaagtgac 1

tgacaaaaaagagtaaaatacagttatgac 1

tgacaaaaagaatctaatgtatatcttgac 1

tgacaaaagaaaaacacactattcgttgac 1

tgacaaaagcaacaaagaaaagtgtttgac 1

tgacaaaatcatataattaccgtgtgtgac 1

tgacaaacaaaaatttaaccgcgtgttgac 1

tgacaaacgtatcattacttcacatatgac 1

tgacaaagacaactaccaatcgagcctgac 1

tgacaaagagatatgatgttagtcactgac 1

tgacaaagagatgaagctaggtgacttgac 1

tgacaaagcaatgctgtggaaatttttgac 1

tgacaaagtctacacttttaaggtgatgac 1

tgacaaatgaaagaaaataagaattgtgac 1

tgacaaatttcacatcattttaaatatgac 1

tgacaaatttgtgttctagtactctatgac 1

tgacaaattttaccgctggattgcattgac 1

tgacaacagcgtgatgaacaggactgtgac 1

tgacaacatatccaacaaattacctttgac 1

tgacaacgtaagactggccctcgttctgac 1

tgacaagaatacaacgtaattggaaatgac 1

tgacaagagtctgcctccattggagctgac 1

tgacaagatgttgattttggttgacttgac 1

tgacaagcccatttttaaattttgtctgac 1

tgacaagttggttgatgtcaagacaatgac 1

tgacaataatggaaaatatgcaaatttgac 1

tgacaatatatacacaagaaacaggttgac 1

tgacaatcaaggatatcaattgttactgac 1

tgacaatcccaaatgatgaagaattctgac 1

tgacaatctattaagaggttgatttttgac 1

tgacaatgagtgcaagtggcaagccatgac 1

tgacaatgagtttaaaggctttaatatgac 1

tgacaatgatggcggaattgtgctggtgac 1

tgacaatgtagtatttgtgacttttatgac 1

tgacaatgtgaaacaagcctgaacattgac 1

tgacaatgtgcatgcaatagtgagagtgac 1

tgacaatgtgtgtatgattttcgaagtgac 1

tgacaattttcaggatataaatattatgac 1

tgacaattttttctttctgcgtgttttgac 1

tgacacaaactaaaggaataaagccatgac 1

tgacacaaattttatagaagagaatctgac 1

tgacacaacctcttacgccatgtccatgac 1

tgacacacagtcacactcacacacactgac 1

tgacacatacctgcatgatcactttttgac 1

tgacacatcatactcctaatctagtttgac 1

tgacacatctactgttaagtcgacattgac 1

tgacaccatagtctaaccttgcaaattgac 1

tgacacctgcctcttagtcacgtcattgac 1

tgacacggcctctaataccttggacttgac 1

tgacacggtgtcgtaattcagagacgtgac 1

tgacacgtgactcatcaactcgcacgtgac 1

tgacactaacccgatagctacaagagtgac 1

tgacactagtgaataagggagaaatatgac 1

tgacactagtgaatgagggagaaagatgac 1

tgacactgactgttagtgcattctgatgac 1

tgacactgtcattcatgactttgcgttgac 1

tgacactgtgggactttataagtggatgac 1

tgacacttcgatttggtaggctaatctgac 1

tgacactttgatcaaatctcgttggatgac 1

tgacacttttcactgcttaaaaaagatgac 1

tgacagaaaccctttatctcaatttgtgac 1

tgacagaagctgctttaaataatcagtgac 1

tgacagaatgagccaaaaacaagagttgac 1

tgacagacaaactctcctttgagttttgac 1

tgacagacaataaaccaatgaaaatatgac 1

tgacagacatgatttcatcttgtatatgac 1

tgacagacccacaattatctcagttttgac 1

tgacagatctacaaaacttcttacagtgac 1

tgacagctctacttattgtaaccacatgac 1

tgacaggaaaaaataatgagattatctgac 1

tgacaggaggagactgatagcagctgtgac 1

tgacaggatcagagagaaaatcttactgac 1

tgacagggagaatctgagaacctgcatgac 1

tgacagggagccgagtttgagtctcttgac 1

tgacaggtaagaggttgttttcgtggtgac 1

tgacaggttacttgaccggagtgacctgac 1

tgacagtgattttgttaaaatagcgatgac 1

tgacagtgcggtcaaagaattagctttgac 1

tgacagttatgttcagaacaatcaattgac 1

tgacagtttaaccgttgatcgtgcaatgac 1

tgacataaaaaattgaaggtccaaattgac 1

tgacataaatatttaataggttaagttgac 1

tgacataattaatagtgatagtgatgtgac 1

tgacataatttacgacaaaaaaaaaatgac 1

tgacatacagatgggcccattgtttatgac 1

tgacataccaaacccaaaaacaaagatgac 1

tgacatataaatggattagtttttgctgac 1

tgacatatatactttttggtttaatttgac 1

tgacatatatattcactatagtttattgac 1

tgacatatattgacaaaaaaaaataatgac 1

tgacatatgactttttctattgactctgac 1

tgacatcaacaacgattttccccagctgac 1

tgacatcaatacatcatctctgacgatgac 1

tgacatcacgttatacaacatactcttgac 1

tgacatcactttttatatcaacttgatgac 1

tgacatcatccaaaagagccgttgtgtgac 1

tgacatcttttcttacaaagactttttgac 1

tgacatgatgatgttgatacaagagttgac 1

tgacatgattaaaaaccttatactgttgac 1

tgacatgtatgcgaaatgtgactgtttgac 1

tgacatgtcccacacagacacacatttgac 1

tgacatgtcgaccagagtttgaccaatgac 1

tgacatgtggctagagtagcatcatttgac 1

tgacattaaatattcgaggtgaaatgtgac 1

tgacattagatgtttatatctatttttgac 1

tgacattcataaacaaaacggtccagtgac 1

tgacattcctcttcagataaatgagttgac 1

tgacattgatatcaaggagcaaaaggtgac 1

tgacattgcatgtttagggaatccagtgac 1

tgacattgtttgttgcgtctaacttgtgac 1

tgacatttgacttcgcaactgaaaagtgac 1

tgacatttgattctaaaagcattatatgac 1

tgacatttgtccacgtttaataagagtgac 1

tgaccaaaacgagaaaagtttttcgttgac 1

tgaccaaattaaaattgtatatttcttgac 1

tgaccaacggaaatagctgaaaacgatgac 1

tgaccaagaagaagaaaagtccgatatgac 1

tgaccaagctttctgcgttaaaactttgac 1

tgaccaatctggtgagagagagactctgac 1

tgaccaatgtgcacgtgccatcggtgtgac 1

tgaccaatttttcactaaagtctcaatgac 1

tgaccacaaacctgaacacaagaaattgac 1

tgaccacactcttgattttactttcttgac 1

tgaccaccgtcttaatcggtggctattgac 1

tgaccagaaaaagctgacaaataaattgac 1

tgaccagaaaattaaaatagcattagtgac 1

tgaccagacgacttttgtcaaagtgttgac 1

tgaccagatattaaagtgcctatagttgac 1

tgaccagcaactaatgggtagtgaagtgac 1

tgaccaggtcgaggtgttattatttctgac 1

tgaccagtaagtaatgagagttaaattgac 1

tgaccatataaacaaagaaccaaaattgac 1

tgaccatatggagtaatggtgaaacatgac 1

tgaccatatttatagcaagtacatggtgac 1

tgaccatgattgttagttttgagttttgac 1

tgaccatggaagactcattatttaaatgac 1

tgaccattacttacacggtaaactaatgac 1

tgaccattgacagagaaatagagttgtgac 1

tgacccaaaaaaaaaaaatcaacaattgac 1

tgacccaattcttgttccgtggattttgac 1

tgacccagctgggtggtccggtcagctgac 3

tgacccatatttatgttgtccatccttgac 1

tgacccattgactcgcccaccatccatgac 1

tgacccgatataccctgcaccaccagtgac 1

tgacccgtcaaagtttaacggaaacttgac 1

tgaccctgaacagttaagatctgacttgac 1

tgaccctgaacagttaagatctgccttgac 1

tgaccctgatcagttaagatctgccttgac 1

tgacccttgttaaagcattcacattttgac 1

tgaccgaaaagataaatgcattttagtgac 1

tgaccgaaacaataaagtaaatacattgac 1

tgaccgaaacgaacttttatataaaatgac 1

tgaccgaatattttttgtataataggtgac 1

tgaccgagaaacaaaaaggaacgttgtgac 1

tgaccgagtcttaatttgtgatgtaatgac 1

tgaccgatatctgaactattatgtgctgac 1

tgaccgatgcagaatcattaacttagtgac 1

tgaccggctatctgaccaaaatcctttgac 1

tgaccgggaaatataaggggtcttattgac 1

tgaccgtgtttctcaatggtaccatttgac 1

tgaccgttaattttaaaaacacttattgac 1

tgaccgttaggcataactttagtaggtgac 1

tgacctaaaaaaaaagtgtattttagtgac 1

tgacctaaaaaggtaaaaaagacatttgac 1

tgacctaaactaattattaattaaattgac 1

tgacctaacaaattataaataatctctgac 1

tgacctaaccaaattcttcagcttggtgac 1

tgacctacactaagaaaactcaccaatgac 1

tgacctacttcttttgtttttttgtttgac 1

tgacctcattcttctgttcttaatattgac 1

tgacctcctttttccagtatacaacctgac 1

tgacctcgataaagtatgtgagcctatgac 1

tgaccttatcttgcgcctgttcattatgac 1

tgaccttcactcggataaatgccctttgac 1

tgaccttcatccttaatgtatatagttgac 1

tgaccttgttgctggaactagaaatttgac 1

tgaccttttccgtagtaaaattgttttgac 1

tgaccttttgttggcccatgaggccatgac 1

tgacgaaactccggcgaattttccggtgac 1

tgacgaaagtcatgtctaaacttttttgac 1

tgacgaatcgacggaagtgagttaagtgac 1

tgacgaattcgtattttagaattgtctgac 1

tgacgacaacgacgactatccaattgtgac 1

tgacgacagcaagacataaaagcagttgac 1

tgacgacgtcatatttcgatcacccctgac 1

tgacgactagattttcatgcaatctttgac 1

tgacgactgatatgatgatgcattagtgac 1

tgacgagacgcttcagacgggtgagctgac 1

tgacgagagactgagtttaagagttgtgac 1

tgacgagatgcttcagacgggtaagctgac 3

tgacgagatgcttcagacgggtgagctgac 1

tgacgagattatgtgaaatcaagttttgac 1

tgacgagctacttgagacggaccggctgac 4

tgacgagctgattgagacgggtgagctgac 1

tgacgagctgcttgagacgggtgagctgac 4

tgacgaggcgcttcagacgggtgagctgac 1

tgacgatactaattcaaggcattgtttgac 2

tgacgatcccatgaatttcacctaattgac 1

tgacgatccgagagcacgctttagggtgac 2

tgacgatctctaaacaaaaaaaaaattgac 1

tgacgatctgcttgagacgggtgagctgac 1

tgacgatgactcaaagaactcagacttgac 1

tgacgattaaatatattagatggaattgac 1

tgacgatttaaaattttagagagttttgac 1

tgacgcatgatggtgacgaagatccgtgac 1

tgacgcccatatcctagtcagggacgtgac 1

tgacgccgtcgcgtcccatcggatgatgac 1

tgacgcctccatgccatggacaattttgac 1

tgacgcgatgactttgtttcattcattgac 1

tgacgcgttttgttgacatgtcttgatgac 1

tgacgctgacacgtcatcagtaataatgac 1

tgacgctgcaagaatgaacttgattttgac 1

tgacgcttaaaaatgattcttcaaattgac 1

tgacgcttatactatgcgcattgctctgac 1

tgacggaaacaaatttatggttgaactgac 1

tgacggatcaaatatgggtttaacgatgac 1

tgacggatccgaatctttttctcgtttgac 1

tgacgggaaactacggaatcactttttgac 1

tgacgggcaaaattgggattggtaggtgac 1

tgacgggcattgttgttgagggagaatgac 1

tgacgggccaccaattttcaaaacattgac 1

tgacgggttttttattggaccgacgatgac 1

tgacggtagaacattctgttttcagatgac 1

tgacggtcgtgtcctttttttaatggtgac 1

tgacggtgatatttcttttataaaattgac 1

tgacggtgattattcttgcaatttgttgac 1

tgacggttaaatatttttcacggctctgac 1

tgacgtagaagacagaagcatcgttgtgac 2

tgacgtataaaatctcaaagtcttcttgac 2

tgacgtataattaataatggtaattttgac 1

tgacgtatttcttatcaaatttagattgac 1

tgacgtcaacactagaaattagattgtgac 1

tgacgtccccacttgactcaacaaagtgac 1

tgacgtcctttaacaacaacaaaatatgac 1

tgacgtggcggtggtggtggtggttgtgac 1

tgacgtggggacaattatcttattactgac 1

tgacgtgtgtatgtgtatattgagactgac 1

tgacgtgttaagtcgtgactggtgaatgac 1

tgacgttaaacccagaatccaacatttgac 2

tgacgttaaatgacgagacaaaattctgac 1

tgacgttaattaatctgactgcagtttgac 1

tgacgttgcaccggatctccggtggatgac 1

tgacgttgggtttgccttttctcacttgac 1

tgacgtttaaaatttggacaaataaatgac 1

tgactaaaaatctgctttcaaaattttgac 1

tgactaaaatgcgttgagtgacgtcatgac 1

tgactaaatatagctcatacctttgttgac 1

tgactaaatcttcaagatcaaggtattgac 1

tgactaagaaaacttatctaaaggcttgac 1

tgactaagaaattaagagcctagacttgac 1

tgactaagccaagaaagttgagtagttgac 1

tgactaagcgtatagtctcgtataattgac 1

tgactaagtaagtgcaaacaaatgtttgac 1

tgactaagttataactaatttgtggttgac 1

tgactaatcataccaaaaattttaagtgac 1

tgactaatcttcaaaaatccatctaatgac 1

tgactaatgtaaagattacatgttactgac 1

tgactacaaatcttgactcaggaacctgac 1

tgactacaagaaatgaaattaagctttgac 1

tgactacaggtggaaaattcgaccagtgac 1

tgactacatgataacaataattggtctgac 1

tgactagaggttttgtcgccgaggcatgac 1

tgactagcgatatctaaagccagcaatgac 1

tgactagcttcgacgttaaccaacattgac 1

tgactagtagctaatcgtattagcgttgac 1

tgactagtctttgtctgcagacaacttgac 1

tgactataataaactttttctttgcatgac 1

tgactatacattttcgattaatctggtgac 1

tgactatatatattatcattttttgttgac 1

tgactatattcctcaacgttactctttgac 1

tgactatcctcaatatactcggagcgtgac 1

tgactatgatgcaaagactctcagaatgac 1

tgactattaaaagtcatatttgctcttgac 1

tgactattaaaattaatatttgctcttgac 2

tgactattacataacgtactaccgtttgac 1

tgactattctccataatgcccacatatgac 1

tgactattggaaaaggaaagaaagcctgac 1

tgactcaacaagaacatatccaccactgac 1

tgactcaagacaaagccattgattattgac 1

tgactcaccagaggcattcgctcctttgac 1

tgactcagcattgcgtaagcagaagctgac 1

tgactcattgttattttaagcatttttgac 1

tgactcattttttggtatttcttgtttgac 1

tgactccattaacagggtaagatttttgac 1

tgactcccttattatgtcttcccatatgac 1

tgactcctctggagcaaaagctgttctgac 1

tgactcgagcaaagtgtttgttaagttgac 1

tgactcgcttctgtttcaacctccgttgac 1

tgactcggattgggcgtcttgtcctttgac 1

tgactcgtgattatggagctgtcttatgac 1

tgactctaaccttctttttttttaaatgac 1

tgactctagggttttgtgaagcatcatgac 1

tgactctcacacaatttgcattaagatgac 1

tgactctccgatgaaagcgatttatatgac 1

tgactcttctaaccgctccacgtgagtgac 1

tgactcttgtatgtcgttgatggacatgac 1

tgactgaataatcaatagttagtacatgac 1

tgactgacgtgtactgtttaatataatgac 1

tgactgatgctattacaaaaattgtttgac 1

tgactgcaataacatgaagaggaatctgac 1

tgactgcattgacatctctattactttgac 1

tgactgcttttagtcctagtttatcatgac 1

tgactggcatcaacgatgcctttgtctgac 1

tgactggtacggatctaatagtaacatgac 1

tgactgtgaaaactttatgaatcttttgac 1

tgactgtgaatttagggaaacaagaatgac 1

tgactgtgtatgaccttgtcaacgtctgac 1

tgactgttaccgaatcagaaagtcaatgac 1

tgactgttcactctttgaggctttgatgac 1

tgactgtttctatcactatcagcatatgac 1

tgactgttttactgacttgtgagttctgac 1

tgactgttttggccactccctttgagtgac 1

tgacttaaaacactgtctaataatcgtgac 1

tgacttaaacttaataacataaactttgac 1

tgacttaataagaagagtttgacttttgac 1

tgacttaattttggacacctttaccttgac 1

tgacttaccatctgtactttaagaagtgac 1

tgacttagtcataattcagtgacagttgac 1

tgacttattacaaataacgatatgaatgac 1

tgacttcaagaagatcgaattggtcctgac 1

tgacttcacataggtagacatctctctgac 1

tgacttcgagcaaaaaaaattaaacatgac 1

tgacttcttttttttttttttttgcatgac 1

tgacttgaatatatggacaatatggttgac 1

tgacttgctgctgtgtacttcatagctgac 1

tgacttggttgaccggtaaaggagtttgac 1

tgacttgtattttaattgccacacattgac 1

tgacttgtcattgtgcaataatgttctgac 1

tgacttgtgactatggatggacaaattgac 1

tgacttgttcaatatttacataatcctgac 1

tgactttaaaactagtgactagaagctgac 1

tgactttactttatttctatgttagatgac 1

tgactttagccctaaaatctattacgtgac 1

tgactttatgagattgagattagttttgac 1

tgactttattctttggacaaattatttgac 1

tgactttcaaggtactgatcattctttgac 1

tgactttcacatttaagtttgttagatgac 1

tgactttctacattacattatagtattgac 1

tgactttgaccaaacacaacttaagttgac 1

tgactttgataacgcacactaataagtgac 1

tgactttggcataagcttaaataagctgac 1

tgactttgtcttaatatcaccgacggtgac 1

tgactttgttcttgggcttgaagagatgac 2

tgactttgtttgtatggttcgggcattgac 1

tgacttttagcttttgacattgactttgac 1

tgacttttaggtttgtaaatgataaatgac 1

tgacttttctgaatgtttttgttgtatgac 1

tgacttttcttgttgttcctctcatgtgac 1

tgacttttgagataaagaatatcaaatgac 1

tgacttttgtagtaatttttatatactgac 1

tgacttttgttgttaataaaaaggaatgac 1

tgactttttaagtgacaccaagattctgac 1

tgactttttcaaaccctaaaccaaattgac 1

tgactttttgagactatcgattattttgac 1

tgactttttgtatacttaaaaaaatttgac 1

tgacttttttaataattgtgaatttttgac 1

tgacttttttcggtcggagaaaaatttgac 1

tgactttttttcttttatttgaaaattgac 1

23 : 410

tgacaaaaaaaaaactaaatatttttatgac 1

tgacaaaaaaaaatacagcgaatgcgttgac 1

tgacaaaaaatgatataattttacttttgac 1

tgacaaaaaattgccagtcaaaaaccatgac 1

tgacaaaaaccctcgatatgaagcatctgac 1

tgacaaaaataaggtattctaacactatgac 1

tgacaaaaatacccttcttctgtaacgtgac 1

tgacaaaacaagcgattataaatttattgac 1

tgacaaaactattacaacttagatctttgac 1

tgacaaaatatactgaacaacattctttgac 1

tgacaaaatatgtgtttcttttaacgatgac 1

tgacaaaatatttgagtattcagattttgac 1

tgacaaaatgtcgaattagaacaaacatgac 1

tgacaaaatgttatcaatgtttacatttgac 1

tgacaaaccaacgttaagctctaaagttgac 1

tgacaaactaaaataaaatctcgttgctgac 1

tgacaaagaaaagtgagaaaacagttatgac 1

tgacaaagatttaaacgacgattattttgac 1

tgacaaagccgcggtggttatagaaggtgac 1

tgacaaagtatcacggtgtccatcatttgac 1

tgacaaagttcaattaaaaaacttgactgac 1

tgacaaataagtttgatcgacattgtgtgac 1

tgacaaatatgctactacgtactaatctgac 1

tgacaaattcatccactgtgttaattatgac 1

tgacaaattgaattccgttaaaattattgac 1

tgacaaatttaggatcaattatgggattgac 1

tgacaacaaaggtgatgatggtgatgatgac 1

tgacaacaagaagttggtctctgatagtgac 1

tgacaacataacgtaactatgagtctgtgac 1

tgacaacataatgttgggtagagatgatgac 1

tgacaaccagctaaagatattattgattgac 1

tgacaaccgttgaggagacttattctttgac 1

tgacaacgaagatgacaacgaagatgatgac 1

tgacaacgacaaaaccgactataagagtgac 1

tgacaacgatggcaaagtagaaaagcgtgac 1

tgacaactatcaactatgtgaagcatttgac 1

tgacaactatttaaaacaaaaaatgtttgac 1

tgacaactcactcccacttatagtcactgac 1

tgacaactttattcgcaaatattcttatgac 1

tgacaagaaagctgcaagagactctgatgac 1

tgacaagagtcgagcagctcaagcttctgac 1

tgacaagcaatggaactcgaaagtttctgac 1

tgacaagggagctttgcctgtaaagtttgac 1

tgacaaggtatgtaagtttttctccaatgac 1

tgacaagtagactcagattctataacatgac 1

tgacaagtatgatttattgtatatagttgac 1

tgacaagttgtcctctttttgtattattgac 1

tgacaagtttttccactaaaaaacatatgac 1

tgacaataagagacagaacactggatatgac 1

tgacaataagagacggaacactggatatgac 1

tgacaataagagatggaacactggatatgac 1

tgacaataccatttactttacgatttatgac 1

tgacaatactaggatctatggaccttttgac 1

tgacaatatatacaaatcaagaggttgtgac 1

tgacaatcgattttcatggacgacttttgac 1

tgacaatctattaaaaaatattcttcatgac 1

tgacaatgaattcggcattgatggatttgac 1

tgacaatgatgctgaaaaccatataaatgac 1

tgacaatgattgttttgggcttcaacgtgac 1

tgacaatggaatgcttttctaaagagttgac 1

tgacaatgttctcatataatgtaaatttgac 1

tgacaatttaccctttgatttggaccatgac 1

tgacaatttggaatctttagctcagtttgac 1

tgacaatttggtaaatgggatatgatttgac 1

tgacaattttatacatgaactaaatgttgac 1

tgacacaaaaatattttgtattaagtttgac 1

tgacacaagaaaccaataaaagtcttgtgac 1

tgacacaagagaacaaaccttttttcttgac 1

tgacacaagttgggaaagtaatgagtttgac 1

tgacacacaataaaatatgaggaaatttgac 1

tgacacatctacgacaaaaactatctgtgac 1

tgacacatgattttactaaaaggatcctgac 1

tgacacatgtcattttatggagagagttgac 1

tgacacattagtgaatagtgataatcttgac 1

tgacaccacatatttttttagagaaattgac 1

tgacaccataacgaacaaagtatagtttgac 1

tgacacccactcaatttcgagtttttctgac 1

tgacacctgttcgtcttggctgtcttgtgac 1

tgacacgtgttcttcttggctctcttgtgac 1

tgacacgttgttttaattaaaaacatttgac 1

tgacacgtttgcactctttcaggcggttgac 1

tgacactacaaaaattcttggaacacttgac 1

tgacactcgaacaaatttctggaagattgac 1

tgacactgctaatccggattgggatgatgac 1

tgacacttgcgggccgatagctctcgatgac 1

tgacactttactttcttcactcttatttgac 1

tgacagaaaaatgtcgacattggatattgac 1

tgacagaatcttcatcaacgctcgatttgac 1

tgacagaattatatccaatcttttaactgac 1

tgacagagattgaagtgtgaagccatctgac 2

tgacagagcagctagagaatttgtttatgac 1

tgacagatacatacgggtaagaaggcgtgac 1

tgacagcaaacgatttgtaccatgagatgac 1

tgacagcattctttactcttggacaggtgac 1

tgacagctacataagcaccattggtgttgac 1

tgacagcttgtaagaagcataactccttgac 1

tgacaggagttagatgcgtacttcttctgac 1

tgacaggtatctctttaattcttctagtgac 1

tgacagtaaaataatttttttgtttattgac 1

tgacagtacacgagaggaagatgaagttgac 1

tgacagtagatgaagaacaaagagcgatgac 1

tgacagtaggtattagctaatgacaagtgac 1

tgacagtgaccactacatcatctattttgac 1

tgacagtgaccagtaatatgtacactttgac 1

tgacagtggttatactttaaatctttttgac 1

tgacagtttcaacagaaaaaatatgattgac 1

tgacagtttgttagaaaaaataatggatgac 1

tgacataaaacagaagtactaggtgtttgac 1

tgacataatagtttatcttataaaaaatgac 1

tgacataatttacctcgttcggatcgctgac 1

tgacataatttgtaaaagttagctttgtgac 1

tgacatagaaaaaagtcagaatccgattgac 1

tgacatagaacaaaaactaaatttccttgac 1

tgacatagggtgaatctacacctaaattgac 1

tgacatatacttaagggtccctatatttgac 1

tgacatatataatttcctatttttctctgac 1

tgacatatataggtccaaatgaatttgtgac 1

tgacatatattaaataaaaaaatgcaatgac 1

tgacatatattgtagcacaagttgtggtgac 1

tgacatatccatatatattagtcgtcctgac 1

tgacatatctgatcaacatgcaaaatatgac 1

tgacatattatttatgccactatattttgac 1

tgacatattcatggagctaaagaaatatgac 1

tgacatattgttcacgcaaaaaagaaatgac 1

tgacatcaaattaagaaattgtctgcatgac 1

tgacatcatcacccaaaaacatgcgcctgac 1

tgacatctctaagataagcaatactcatgac 1

tgacatctgttaaaaagataaaaaaaatgac 1

tgacatgactagtggacgaagtttgaatgac 1

tgacatgacttcatgttcagacgtccctgac 1

tgacatgagaaaagtgtgataaaatgttgac 1

tgacatgatagatttgtaatttgtttttgac 1

tgacatgatgaataagatatttatagatgac 1

tgacatgcatttatttacgtgtgatgttgac 1

tgacatgccgtcagcgtcggagattgttgac 2

tgacatggattttatgacattagatattgac 1

tgacatggtaaaagttgtgcagtaccttgac 1

tgacatgttgtaagaatgatttagcgttgac 1

tgacatgttgtggtattttgattggtttgac 1

tgacatgtttataatagctaaagacactgac 1

tgacattaaaagtataacaaaattctctgac 1

tgacattattttgtgttcttgttcagttgac 2

tgacattccaccttgaattattgtttctgac 1

tgacattccgcgacatttatattatagtgac 1

tgacattccttaacaaaaaatccactatgac 1

tgacattgaaccggataaactcggttatgac 1

tgacattgatgcaaatttcagggagtgtgac 1

tgacattgctaaaaatcttcttggggttgac 1

tgacattgtcaagtctactactccatatgac 1

tgacattgtgaaatgcattatgagtcatgac 1

tgacattgtgtgatctaatgtagaaactgac 1

tgacatttccaactctacatgtgactttgac 1

tgacatttctaaactcaggatgtgtagtgac 1

tgacattttcgaactgtgttacgttcttgac 1

tgacattttgtccctgtggattctgtatgac 1

tgaccaaaaaacacggcatatgagagttgac 1

tgaccaaaagtaacaattgaaaaacaatgac 1

tgaccaaaggtgcaccaatagttggtctgac 1

tgaccaaatttgagtcaaataggctaatgac 1

tgaccaacaacatcttcatagaaagtctgac 1

tgaccaactgaatgtggaaattatatttgac 1

tgaccaataaaattggtagaaattctttgac 1

tgaccaatggcctaaaagtcaaacctttgac 1

tgaccaatgggctttggacagcccaattgac 1

tgaccacaatacttgcaatccgaataatgac 1

tgaccacactcacactatgattaattctgac 1

tgaccacactgacaactatcggatcgttgac 1

tgaccacgcaaaagacgaaattgagtttgac 1

tgaccacggagctcaagtggttgtagttgac 1

tgaccactgacacggtttaaataaatatgac 1

tgaccagaagagtaaattaggctgagatgac 1

tgaccagatgtagtaatttgttatgcttgac 1

tgaccagtgcaacacaatctcaaagaatgac 1

tgaccatccacacatttgaatattgtctgac 1

tgaccatgatgtaggatcattaagtcatgac 1

tgaccattctctaatctttatcttttttgac 1

tgaccattttaaacataaactaagcgttgac 1

tgacccaataagcccagcagccggatttgac 1

tgacccagatttggtacagcccataaatgac 1

tgaccccacaagcaagcagtagtaatgtgac 1

tgaccccgatttcgaacctccagagagtgac 1

tgaccccgttacgattaaggcggttggtgac 1

tgacccctattaaatacctctatctactgac 1

tgacccgagagggagacatcaatttaatgac 1

tgacccgcaaaatcttgtatttatttttgac 1

tgacccgccttggtccgaacaagtaactgac 1

tgaccctgaacagttaaagatctgccttgac 1

tgaccctgaagatcgaaagcttagatatgac 1

tgaccctgaggcttctttactatgggctgac 1

tgaccgaaaaagataactactctttggtgac 1

tgaccgcgttcgagagctcagtttcaatgac 1

tgaccggctccaacaaaactttgactctgac 1

tgaccgggatctcctgcgatatctggatgac 1

tgaccgtcgaaaacaaatttattaaagtgac 1

tgaccgtcgatagacggatgataactatgac 1

tgaccgtctgatctaagccaccttctttgac 1

tgaccgtggacgctacagttacttacctgac 1

tgaccgttttaactctcaacaaaaagttgac 1

tgacctaaatactaacatcaaattttgtgac 1

tgacctatacatagtcaagctatgacatgac 1

tgacctatagagttataaccttctctatgac 1

tgacctatagttacttgtcgaatactgtgac 1

tgacctatatcgtgcataagtattctctgac 1

tgacctatcaaatgagtccaaaacggatgac 1

tgacctcaaaccaggaaacatcttgcttgac 1

tgacctcacttgctcttggcatcgacttgac 1

tgacctcattttattgggtaaactgaatgac 1

tgacctcgaaaaagaggcaatggcagatgac 1

tgacctgtccaccgacgaattcggttgtgac 1

tgacctgtctgtatacttccatcataatgac 1

tgaccttaacttaacttcttctgatgatgac 1

tgaccttaatggaaacatcttcccgtatgac 1

tgaccttccaggaagctctaatgcatctgac 1

tgaccttttcgtaacctcccctcctcgtgac 1

tgacgaaactaatctgttaaaattatgtgac 1

tgacgaaagatcatttggtgatctcactgac 1

tgacgaaattattcgcgtatgatgcgatgac 1

tgacgaaattgttttgcgggaaaagtttgac 1

tgacgaaattttaaaattatttgagaatgac 1

tgacgaacatttcgctttgtacacttctgac 1

tgacgaagaagttggcgaagatgaaattgac 1

tgacgaagatgatgaggaagaggaggatgac 1

tgacgaagtagaatatttgtctcactgtgac 1

tgacgaatttgccctggaacgagaaactgac 1

tgacgacataactcagccgcaatgatatgac 1

tgacgacgcgagaaggtggtgaatctatgac 1

tgacgagacatatgaggaatcaacggctgac 1

tgacgagactgaagctaaacataactttgac 1

tgacgagggtttatcttcttttgacaatgac 1

tgacgatcagtgcaagatgtttcttaatgac 1

tgacgatgacagattagtttctagtgatgac 1

tgacgatttactaaaatgcccccttcatgac 2

tgacgatttttatttttatttttattttgac 1

tgacgcaattagtgtaacaaaatgtaatgac 1

tgacgcacatccttacgtaaccatcgttgac 1

tgacgcagttttgcatccacattgtgatgac 1

tgacgccatattcagagacggcggaggtgac 1

tgacgccatttgtaaacgtcagaatcatgac 1

tgacgcgaacctaggatgcacccagattgac 1

tgacgcgatgtctagatcttccgtctgtgac 1

tgacgcggttttgtctgtgttcaatgttgac 1

tgacgctccactctaaccctcttaatctgac 1

tgacggaaaaacatgattttgcggttttgac 1

tgacggaaggtgggtctctgtctcccctgac 1

tgacggagataacttttttcacttgggtgac 1

tgacggatctgaccggtcgaaccggattgac 1

tgacggcaatggtggcggtggagattctgac 1

tgacggcggcagtggtggtgatgactgtgac 1

tgacggcggtaggtttgtcaccggagatgac 1

tgacggcggttagagctaggccggcggtgac 1

tgacggcttggaagggtcttcaaccgatgac 1

tgacgggaaaacatgattttaccgttttgac 1

tgacgggtttaggttgataataatacatgac 1

tgacggtcctaccttaataaacaaaaatgac 1

tgacggtgtatgaattgccatgtgtattgac 1

tgacgtaaagcttctggttaattgctttgac 1

tgacgtaaggaaaacaatcattacaagtgac 1

tgacgtacacatttcaaagtacacacatgac 1

tgacgtacatagaaatctataaattaatgac 1

tgacgtacatttcagactaatatggtttgac 1

tgacgtcaagtcttttagccatctttttgac 1

tgacgtcagctcctcctcacgcgtccgtgac 1

tgacgtcatctgacagtgaagaacacgtgac 1

tgacgtcgtttctagttgggatgagactgac 1

tgacgtggaggttctaggtcagaacgttgac 1

tgacgtggttgtataacgtgagtcacgtgac 1

tgacgttatcgatagtgtctgagcttttgac 1

tgacgttgacatagtccattgacataatgac 1

tgacgttgacccacgaaaatgtggttttgac 1

tgacgttttagatctagttctatcagttgac 1

tgacgttttgtttttgacaaaatacgatgac 1

tgactaaaagcattacaaacttatcaatgac 1

tgactaaaagtgaaatgatatcaacggtgac 1

tgactaaaatgaattatttgaaaacgttgac 1

tgactaaacaggacaaatatatattgttgac 1

tgactaaataaaaagcaattaagaagttgac 1

tgactaaatgttttctaacactatgagtgac 1

tgactaaattaataatttaaaaagtattgac 1

tgactaaattattatttttggttcagttgac 1

tgactaactaatggagtttgaataaaatgac 1

tgactaattgctgatgattttttacaatgac 1

tgactacgaggatgaagatgatgaccttgac 1

tgactacgatttggcaacagaaacaattgac 1

tgactactaagtaaaatatatagtcattgac 1

tgactactaccaaggcaccaatcaccatgac 1

tgactagaaagacactgagactgagtctgac 1

tgactagaagagcgacatgtatagatgtgac 1

tgactagaagcagaatacatattagcgtgac 1

tgactagacaaggttcgatatactcgttgac 1

tgactagaggttttaaagaataaaatttgac 1

tgactagctttttgagctcattaattatgac 1

tgactaggctaatgggccggtctattgtgac 1

tgactagtaacaggtacaatttagctctgac 1

tgactatacaatttaaggttatgtgcgtgac 1

tgactatacatcaagagttggcttaaatgac 1

tgactatatatcttttttttttttttgtgac 1

tgactatattttagtatcactttctcttgac 1

tgactatcaaacaaagccaatgacatgtgac 1

tgactatcttgactctttgaagaattctgac 1

tgactatgattttgtgctgatccagagtgac 1

tgactatgtggttccatgacttccatctgac 1

tgactattacacactttaaactaaatatgac 1

tgactattactaactagttcttggttatgac 1

tgactcaaaatagaaatttctgcagattgac 1

tgactcaaatgagttaggtgcacgaaatgac 1

tgactcataaaaacacttgagctcatttgac 1

tgactcatcatactatgaaaatatcgttgac 1

tgactcatctcttccacaaaaagtcattgac 1

tgactcatgaggcaggtttgaatgttatgac 1

tgactcattaatggaaaataaatgttgtgac 1

tgactcattttctaaaaccatcaaggctgac 1

tgactccaattacattcgtcggcgtattgac 1

tgactccattgcgttggccatgtgacatgac 1

tgactccatttacacattcccttgccctgac 1

tgactcgaagatggtcgaagaaattactgac 1

tgactcgatcagagataatctcgtccttgac 1

tgactcgtgttcttatcatctttaagctgac 1

tgactctagcagctcggattctgaatctgac 1

tgactctcacttccttacaggttcacatgac 1

tgactctcttaatatagattaataatatgac 1

tgactctctttccgtaacggagaagcgtgac 1

tgactctgttccgcctattgttcaccgtgac 1

tgactcttattaggatccactcatggatgac 1

tgactcttcaaccaacttatatacgtatgac 1

tgactcttcttcttcttcttcttcctctgac 1

tgactctttagctgttgagcatgaaactgac 1

tgactcttttcatcacactatatatattgac 1

tgactgaaatgaaatatttgagagggttgac 1

tgactgaatatttatgagattggactatgac 1

tgactgaattttcctatactcatgcgttgac 1

tgactgacccggacgtgtacctaaaaatgac 1

tgactgagaacacacattgtgttgtgatgac 1

tgactgagaagaaagaaagaagttgactgac 1

tgactgagcaatactaattaagctgactgac 1

tgactgatcaaacgaacagggccttagtgac 1

tgactgatctaacaactttaattatgttgac 1

tgactgatcttgtgtcacattgaagattgac 1

tgactgatgaaacctttcacgtgacagtgac 1

tgactgattggttttgaccaaaaaaattgac 1

tgactgcgatatatcaatattataaaatgac 1

tgactgctttttccactattataaccttgac 1

tgactggaaaaccaaaggagacaaggttgac 1

tgactggagaatcaatggagaaaaggttgac 1

tgactggagagtttggaaagcatgtaatgac 1

tgactgggcctattaaaggtttctctttgac 1

tgactggttttatagagttgggtctcatgac 1

tgactgtaacgtggaccttgtttttgctgac 1

tgactgtccttaactcttttaccaacttgac 1

tgactgtgatggcgtctatttgttagctgac 1

tgactgttcatgttcaaggaagagacttgac 1

tgactgttgaccaatcaattgaaaatgtgac 1

tgactgtttataatcataacgtacaggtgac 1

tgacttaaaaattcttaaaatagatcatgac 1

tgacttaagtttcttcattgttgtttctgac 1

tgacttaataaaatttgatccaacatatgac 1

tgacttaatgttatagtctgtatatattgac 1

tgacttagactttgtttccagctcccttgac 1

tgacttagatagactttgttatagacatgac 1

tgacttatatatcgttgagagtgacattgac 1

tgacttattaaaaaactaataatgtattgac 1

tgacttattgatctttgttttgtgttatgac 1

tgacttattttattacaaatttatatatgac 1

tgacttcaaaaagaaaaggagggagagtgac 1

tgacttcactaaatgaagttgatttgctgac 1

tgacttcagccccaactcagtgtgcactgac 1

tgacttcatcaagacaaagaccacttttgac 1

tgacttcatttggtttaagagtaaaagtgac 1

tgacttcatttgtcttcagaatagttatgac 1

tgacttcattttattgggtaaacgttttgac 1

tgacttctccacctcctaaaatgagaatgac 1

tgacttctcttttttttatcgtagttttgac 1

tgacttctgatcgagattgaaggacaatgac 1

tgacttctttaagagatatggacgttatgac 1

tgacttgaaatctgttgttgagccgcgtgac 1

tgacttgacttgactcattgcttgacttgac 1

tgacttgatcgttcacatgaaatcgcttgac 1

tgacttggcacaatttaagataagttttgac 1

tgacttggccgcttggtattgtagctatgac 1

tgacttggtgaatttaattagtgactatgac 1

tgacttgtagtcttgtaccattgaatttgac 1

tgacttgtcaatttgttaaggttaatttgac 1

tgacttgtcagtagattgacctctcaatgac 1

tgacttgttcttctcctccttggtcgttgac 1

tgacttgttgggaaaacaaaactctaatgac 1

tgactttagggaatggcctacctaatttgac 1

tgactttattctaagttcaaatttagatgac 1

tgactttcaaattaatcttcatttttatgac 1

tgactttcacaagccactctgctaagttgac 1

tgactttcacttttgccttaaagtgattgac 1

tgactttccccaatgtcttgtcttatttgac 1

tgactttccctggctgtggtcttttgttgac 1

tgactttccgcttcctcctttttctggtgac 1

tgactttcctcttgggtttaggctgattgac 1

tgactttcgtggaaatttctacttagatgac 1

tgactttcgttgttgagtgtctcttactgac 1

tgactttgaaaatttcaaatacgacaatgac 1

tgactttgccggagaagattggtcagatgac 1

tgactttggggacttttagttgtccgctgac 1

tgacttttaaaaaaattgtaatcgatgtgac 1

tgacttttcttcttaacttgcatgacatgac 1

tgacttttgatacaaccattaatagtatgac 1

tgacttttgcctctaaatatctggttctgac 1

tgactttttattattattattgtagtatgac 1

tgactttttcgataaattaatcgaaattgac 1

tgacttttttgtgcgaaaatatactattgac 1

tgacttttttttttctctgaatgtttttgac 1

24 : 339

tgacaaaaaaaaaagaaagaaagctacttgac 1

tgacaaaaaaataaaataaaaaatggcatgac 1

tgacaaaaaacaattttttaatttttattgac 1

tgacaaaaattacagataaaagagtaactgac 1

tgacaaaacacaaacccatttggtttgttgac 2

tgacaaaacatgttcacaagattttgtttgac 1

tgacaaaatcaagaaacagacttgagcttgac 1

tgacaaaatttaaaagtaaatatgaggttgac 1

tgacaaaattttgtaggccgagttagattgac 1

tgacaaacccataagaggtcgataggaatgac 1

tgacaaatctttaaaattgaataagtgatgac 1

tgacaacaaatctctcacgcgtcagagatgac 1

tgacaacaagtgagaaaaaaattataaatgac 1

tgacaaccacgcgatctcttttataatttgac 1

tgacaacctcgttcttactcttctcgtatgac 1

tgacaactactattgcagacaaggcttttgac 1

tgacaactagaacctcatgagtggaggttgac 1

tgacaactattaatttccaaataagtcgtgac 1

tgacaactatttaaaaaaaaaatttatttgac 1

tgacaactatttaaaacaaatatttgtttgac 1

tgacaactgagtcaacatatatcccgattgac 1

tgacaagaaaagataagtttttttattatgac 1

tgacaagaaataagaggctaatttcacttgac 1

tgacaagcagcctgtctttcccttgttctgac 1

tgacaagcatgggggtgaccaaaagttttgac 1

tgacaagcgacgagtgacgagcgacgagtgac 1

tgacaataaaacaatttaataatttacgtgac 1

tgacaataacccgtcgccaagcggactgtgac 1

tgacaataataaatacaactaatatgtgtgac 1

tgacaataatccaccaactcgtttctcctgac 1

tgacaataatctggtctttcaacatgcatgac 1

tgacaatacaaaccaatgtgctctgcattgac 1

tgacaatagtagtagttcgatacgctcgtgac 1

tgacaatattgcatgaactgggcaatcatgac 1

tgacaatatttgtctctaaaacttatagtgac 1

tgacaatcactcatggcttagtgaatagtgac 1

tgacaatcagtatatatgttacctcgcttgac 1

tgacaatgtattcgaaatttggaagcattgac 1

tgacaatttcataaagaaattagttgtttgac 1

tgacacaaaagaagaggaaacttctgtttgac 1

tgacacaaagtcaagccaaccccaactttgac 1

tgacacaattcaatatacatcaaattaatgac 1

tgacacattattattatgatatacggtttgac 1

tgacacattctcaaaagtaattcaatcatgac 1

tgacacctaaaattaaccaaaacaaaagtgac 1

tgacacgattaaaaaacattgttgtatatgac 1

tgacacgcagtaaacgcgtgatcgtcattgac 1

tgacacgtcaactaatctctgaggcctatgac 1

tgacacgtccggccgttgacaaacaaagtgac 1

tgacactacacaaaacggttgcaatgtatgac 1

tgacactctgtcccgatataatctcttttgac 1

tgacacttttttggtacaaatcaaatagtgac 1

tgacagacacaaaaacacaaatgaatgttgac 1

tgacagatcttagtcgtacataaatggctgac 1

tgacagattactgcagattaactaacactgac 1

tgacagcattaaacagattattattttgtgac 1

tgacagccaacgcttaccaacacaagtatgac 1

tgacagctgatgcatgattctcaaatcctgac 1

tgacaggaaaagaaaaaaaaaggaagtttgac 1

tgacaggagcgaaattggtcttgactcttgac 1

tgacaggagtggtgggaaatatgagtgttgac 1

tgacaggcgataatgggttattctttaatgac 1

tgacagtgactgctagtgtttacccaaatgac 1

tgacagttatcaaacttttgagagtttgtgac 2

tgacagtttaaattttgcgatgcaaatgtgac 1

tgacataaacacattattatgtgttcaatgac 1

tgacataacattcatatatagttaacaatgac 1

tgacataatctttttcgactgctataattgac 1

tgacatactacactacttgattatggtctgac 1

tgacatagaaatacaaatacatatcgtttgac 1

tgacatagtcttctatagaaattaattttgac 1

tgacatatataatataattcaaataaaatgac 1

tgacatatatggcaaaccagacgaaagctgac 1

tgacatatggttttattactgattttcttgac 1

tgacatattttaccaattaccaccatgctgac 1

tgacatcaatatcaaatgttgatttttttgac 1

tgacatcataatgttttctttaaaagtttgac 1

tgacatcctactaaaattagaaacaaaatgac 1

tgacatcgttttgcttatgtcacgtttatgac 1

tgacatcttcggcaatgttgactgtctctgac 1

tgacatgaagagttaaaaccatcaaatttgac 1

tgacatgaatatttggcttcagaagatttgac 1

tgacatgaatcttctgtttccacttacttgac 1

tgacatgactcttaaatttctttttggttgac 1

tgacatggatcatgaattcaaggttccctgac 1

tgacatgtaaacaaaagatgcaacattctgac 1

tgacatgtgaatttatatctattgaccctgac 1

tgacatgttaaatacagttgttttctagtgac 1

tgacatgtttattacctttgcaatattatgac 1

tgacatgtttgttaataatataggtctatgac 1

tgacatgttttcaagttgtcttgatccatgac 1

tgacattaaatttattatctaatttttgtgac 1

tgacattacggctgagttgtgaattaaatgac 1

tgacattagatattgaccaattaaaacttgac 1

tgacattattgtattgcaaaccaattgatgac 1

tgacattgccagtcacgtgcctcttttttgac 2

tgacattgtatcaaacatttgtaataagtgac 1

tgacattgtcactcacgtgggcctcttttgac 1

tgacattgttgaaatggtaaataaaaaatgac 1

tgacatttgaaaataatttgttgaattttgac 1

tgacatttggcatttgaggatccttgtttgac 1

tgacattttatcaccttgatatgtattttgac 1

tgacattttcacaagactgaatcaaatttgac 1

tgacattttccaacgtaaaaaagatttatgac 1

tgacattttgttgaaacttgaaaagacttgac 1

tgaccaaaaaaaaaacaataaaatctgatgac 1

tgaccaaaaacacgacgacaaaaaaagatgac 1

tgaccaaaaacgagcatgactgtggtaatgac 1

tgaccaaaaagttaaaatatgaaaattgtgac 1

tgaccaaaaggaatatgtgactcgtgactgac 1

tgaccaaacaaagtcttaacatgcgttttgac 1

tgaccaaacgatatctgaattcttattttgac 1

tgaccaaacgcaaaagtcactaaaacagtgac 1

tgaccaaagcataactaaatagttttcttgac 1

tgaccaaaggtggtacataacacacgtatgac 1

tgaccaaatacaatatgttaagttcatgtgac 1

tgaccaaatcatggaggccgataccttttgac 1

tgaccaaatgtatcatactgcattgtaatgac 1

tgaccaacaacattacaaaattaataagtgac 1

tgaccaacgattttcacatagtaacaggtgac 1

tgaccaagaagatgatcatatagtattatgac 1

tgaccaagctacttgattcatatccatgtgac 1

tgaccaagttaaaatatttaaaatagtttgac 1

tgaccaataccagtgtgacctccacacttgac 1

tgaccaatatggtatatgttctttttactgac 1

tgaccaatattgttttgttttgaatgtttgac 1

tgaccaatgacggcgctcgcacggagcctgac 1

tgaccaattttttccaatgatatttgcatgac 1

tgaccacactaaaaatgcctatgttcgttgac 1

tgaccaccccctcacatgattcacttactgac 1

tgaccacctgaaacctctctttgcacattgac 1

tgaccagttacgtaaatatggacttaattgac 1

tgaccagttcaaccggttagcagattaatgac 1

tgaccagtttaaccatctcaaaggtatttgac 1

tgaccataaaagggtttgagaacttgcatgac 1

tgaccataacaagccaattgctttaaaatgac 1

tgaccatactcttcttttcttggtggattgac 1

tgaccatatataatgatatagacaacgatgac 1

tgaccatatttgaaatatcaaatgacagtgac 1

tgaccatgactttgaaagtgtatgtacgtgac 1

tgaccatgtgatcgtacgtggctcctcatgac 2

tgacccaaaattgttgaataagtaaatttgac 1

tgacccaatttaagcccacagcagactctgac 1

tgacccactaggacgaaacattgttgcgtgac 1

tgacccactcttttgaattttcacaacatgac 1

tgaccctatcctctcgatctcttacttttgac 1

tgaccctctcctaaatgtttttgttctttgac 1

tgaccctctcctggaggcgcaaagttcatgac 1

tgacccttgtctggtcttttcattcgactgac 1

tgacccttttgtgtcactgtaatttgagtgac 1

tgaccgatataatgtatttcccgtgacttgac 1

tgaccgcacgctcgtgtaggggtccttctgac 1

tgaccggactggcttgtggcttcctcgatgac 1

tgaccgtaaatatcacattaaaatagaatgac 1

tgaccgtctttcaaacctttgtaccatttgac 1

tgaccgtgaaatgtagtttctttacatttgac 1

tgaccgtggagaggcagccttggaatggtgac 1

tgaccgttattacgcagtaggagaaatatgac 1

tgaccgtttgtgaattgtcggagaaatatgac 1

tgacctactaatattatcaaattgatgttgac 1

tgacctagaccactctcattggtttgtatgac 1

tgacctataaatcggcaaatctgtatcgtgac 1

tgacctctgtacaagattaaatccaatttgac 1

tgacctgaagaaaacgttgaggtctctctgac 1

tgacctgctctgtctatatgtgatattgtgac 1

tgacctgtctcctaatttgttaattaattgac 1

tgacctgtgaattcaccaaagagagtcatgac 1

tgaccttacacacactaacacactcatgtgac 1

tgaccttatcaattctagtagcggttcctgac 1

tgaccttttttttgtcaaagaaaatttgtgac 1

tgacgaaaaagtggcttaacttaactaatgac 1

tgacgaaaaccaacgacagccgtttgattgac 1

tgacgaagtctcggctgaaactataaaatgac 1

tgacgaatcccatcatatccaaaaccattgac 1

tgacgacgactttgtctttccgattccgtgac 1

tgacgagatatttcttttgttcagtccatgac 1

tgacgagcgacgagtgacaagcgacgagtgac 1

tgacgagcgacgagtgacgagcgacgagtgac 2

tgacgagctgacttgccgattgacgagttgac 1

tgacgagtaggtgaggaattggacgagctgac 2

tgacgatagttttgcaggtaaaatatggtgac 1

tgacgatataaagatgacctaattggcctgac 1

tgacgatgaacaataaccgaggaaaaagtgac 1

tgacgattatacggataagaaagatcgctgac 1

tgacgatttttgtgtaaacgcgttcccgtgac 1

tgacgcagaattaaaggaaatacgtgtgtgac 1

tgacgcaggcgacgctgagaaaaaggcctgac 1

tgacgcatattacaacattgcattaagatgac 1

tgacgccaggaggcaaatctctctgtcatgac 1

tgacgccattgtcgtcgccggaaaatgatgac 1

tgacgccgtgaaataacggtgttgggcttgac 1

tgacgctcatgaaaaacgcatctctttctgac 1

tgacggaaaaaaaaaacaaaattaaaactgac 1

tgacggaaaaaaaggaaataaatatcgatgac 1

tgacggaaacaaatagaaattataaatttgac 1

tgacggacaccaacaagcataagttatatgac 1

tgacggacacggcaagaagaagaatagatgac 1

tgacggattaattgcgaatttaagtttttgac 1

tgacggatttgttcaaaggaagcttctttgac 1

tgacggatttttttttgtgagaaaattttgac 1

tgacggcccaattgatgcgaatatggattgac 1

tgacggctctttgacctcttcgcctagttgac 1

tgacgggctgacttgtcgattgacgagctgac 1

tgacggtatttttttactaagattattttgac 1

tgacggtgtagtgcaaactaacaaattatgac 1

tgacgtacatgaattttaaggttgactctgac 1

tgacgtcactgctgtgacgctagaatgatgac 1

tgacgtcatcacttcttcgtcatctccttgac 1

tgacgtgaaaaaaaaagtaaacatataatgac 1

tgacgtgaagagaaaacctctctgagtttgac 1

tgacgtgaagcaccgatttaggtggatttgac 1

tgacgtgaagcaccgattttggtggatctgac 1

tgacgtgcagcttggtggtcttcaagtttgac 1

tgacgtggctaaagaacatccttatttatgac 1

tgacgtgtccaactttaattggataagctgac 1

tgacgttaaaaatatatatataagttcttgac 1

tgacgttcctataaggtaaataacatggtgac 1

tgacgtttaaatttcaatcgcagatccctgac 1

tgacgtttgtagcccttacgtttgacattgac 1

tgacgttttcttgtcttcgaatagtatttgac 1

tgacgtttttacaaatgtgttaaatgtttgac 1

tgactaaaaaagtttcctttcggttatgtgac 1

tgactaaaaacttcttaagcgcaatcactgac 1

tgactaaagcattgggtcgtcgtgagtttgac 1

tgactaaatgtggtcgcatctgtgctattgac 1

tgactaacatttactataacttggtatgtgac 1

tgactaagatgaagtcatctattctcaatgac 1

tgactaagggttaattactaatattttctgac 1

tgactaagttgtgattcttgcatcatcatgac 1

tgactaatacgtaatttaatttctctcgtgac 1

tgactaatatcatgtaacaatagacttgtgac 1

tgactaattaaacgtttggttataatagtgac 1

tgactaattaacactttatgatctatattgac 1

tgactaccaaaggtatgtcgagtaaatgtgac 1

tgactacgcgcatcttatttttttattttgac 1

tgactactaattttagaatgatttttattgac 1

tgactagaatttgtatttcagtatttcttgac 1

tgactataaagccaaaggatttaacatgtgac 1

tgactatattgtctcattgattcaacagtgac 1

tgactatctattgaatatgcagtgtttatgac 1

tgactatgtcaagtttaggttgatgttgtgac 1

tgactattaagtagccactaataatatgtgac 1

tgactattattggaaacaactgttttgatgac 1

tgactattgaccaataaactttattatttgac 1

tgactatttttagagagttgttgtttaatgac 1

tgactcaaaaaatatttcgacgttaccgtgac 1

tgactcaaaaatagagatttctacagattgac 1

tgactcaaaacatatgacaccctgaagatgac 1

tgactcaacacgtatgttatcatgaggatgac 1

tgactcaatcatagcttagttctgtaaatgac 1

tgactcaatctgtcttaaaccggaaagttgac 1

tgactcagaagatctttagaatcaacggtgac 1

tgactcagcagaaagattaatctacctgtgac 1

tgactcatctatagtgtaatcatatatgtgac 1

tgactccaacgtccttaactgtttttgctgac 1

tgactccacattttatcaatttttttcttgac 1

tgactccatctggttttaagagtaaaagtgac 1

tgactcctgaatgtgccacggttatgcctgac 1

tgactcgtgcgatagactcctgagacaatgac 1

tgactcgtggcgcacagtcaatgacttatgac 1

tgactctaattgattaatataattgttttgac 2

tgactctcccggctgtgcaagcatcagatgac 1

tgactctcgattagagaggaaattaaactgac 1

tgactcttctgtgactcttttcaacttctgac 1

tgactctttcgaactaggtattgttcagtgac 1

tgactgacgacaaagaagctaaccgtcttgac 1

tgactgacttttagaggatgaactaacatgac 1

tgactgagactacatcttatgatttatttgac 1

tgactgagcaactgtagagtcaagctattgac 1

tgactgatagacagaggctcaaagctagtgac 1

tgactgctaagaaattttcattagtttatgac 1

tgactgggaaggttgttagatttgattttgac 1

tgactggtaatcatttcaaaggtaagcttgac 1

tgactggtgcgaacagggtcattatctttgac 1

tgactggtgtttctaaagtaatcagagctgac 1

tgactggttattgtttcaaatgaaagcttgac 1

tgactgtacttttatcacaaatattatttgac 1

tgactgtctttgactcccgcttttactttgac 1

tgactgttgactccgagctctgtttcgatgac 1

tgactgttgatagataatatggtaagattgac 1

tgactgttgttaagctttcattatcagttgac 1

tgactgttgttgatgttggaccaccagctgac 1

tgactgtttgttttttcttactaagttttgac 1

tgactgttttcaatcttcaattttggtatgac 1

tgacttaaagattttagataattactgatgac 1

tgacttaaagtttttgaacaaaaaaagatgac 1

tgacttaacatgcggctgttgccaacaatgac 1

tgacttaactctaaaagtctaatattggtgac 1

tgacttaagttattatcggatacggtaatgac 1

tgacttaattattctaatttttgaaagctgac 1

tgacttaatttagacgactgtgagtctctgac 1

tgacttagggatacgagtatatatcccttgac 1

tgacttataatcatataggcacaatttgtgac 1

tgacttattatttggaagaggagcacattgac 1

tgacttattcttcaccgtctgatatagttgac 1

tgacttattgaccagtcaaagcatgctttgac 1

tgacttattggtaaaagaatgtagggattgac 1

tgacttatttatttaattcagtatttgatgac 1

tgacttatttgtaactattatgagtttatgac 1

tgacttcaaaatttgtatgttattatattgac 1

tgacttcaaaatttgtatgttattttgttgac 1

tgacttccgtccctcgtgtattctgttctgac 1

tgacttcgacagataaaacggacgcgtttgac 1

tgacttctacctttcttaatttccagcttgac 1

tgacttctctcgactaatatcgctaatatgac 1

tgacttctctctctaccctgtttctccatgac 2

tgacttctgttagggtttcttatgtgaatgac 1

tgacttgagacaacgagtacacactacttgac 1

tgacttgcatccagtttaatcccgtggatgac 1

tgacttgctcgggtaatccaaccgcaactgac 1

tgacttgtaaagcagacctatttcgttttgac 1

tgacttgtcgctttgacaatatccaaaatgac 1

tgacttgtgtaagaatattagacttacgtgac 1

tgacttgtgtcttcctcctcttaatcgttgac 1

tgacttgttacagtttttatatatatgatgac 1

tgacttgtttcatcaaaaatattcaacttgac 1

tgactttaagatatatatacctttcacatgac 1

tgactttagtctttggttcattgtaacatgac 1

tgactttattatgactaacaaaagatcgtgac 1

tgactttcatgccactacttaatagttatgac 1

tgactttctccaaacaacttgagtgtagtgac 1

tgactttgacttggttggtaaaccgagttgac 1

tgactttgactttgtttttcgtaagctctgac 1

tgactttgacttttgacactcgttttcgtgac 1

tgactttgatattttcttggttacaagctgac 1

tgactttggactcgcttctaaaggacactgac 1

tgactttgtcaaccccataaaaaaaagttgac 1

tgactttttgttgtgatagtataaaaaatgac 1

tgactttttttcgatgatataaaatttatgac 1

tgacttttttttagaggacgggttggcatgac 1

tgacttttttttttttttaaatgcattgtgac 1

25 : 364

tgacaaaaaaaaattgtttggaaattacatgac 1

tgacaaaaaaaacaaagcttgtgagtctgtgac 1

tgacaaaaaatgaggtttgtattggacagtgac 1

tgacaaaaaattagtaagtgtgatggaaatgac 1

tgacaaaaacacaaaaacacaaaaacgaatgac 1

tgacaaaaacatcatactccacctgtatttgac 1

tgacaaaatctacagcttaagttatttcttgac 1

tgacaaaatttcctcaaatagcttggtattgac 1

tgacaaaccgatcggtttgttttaattcttgac 1

tgacaaactaattcataaatcttccaaattgac 1

tgacaaactagtccaatggaatttgactatgac 1

tgacaaagaaattaaaataattcatttggtgac 1

tgacaaagaccaagcaccagacaaaacgttgac 1

tgacaaattcataatcatattggtttcgttgac 1

tgacaaatttgcgtatcttctagttgggttgac 1

tgacaaattttttttgttgagtatttttctgac 1

tgacaacaaatggaatggagcattaaaaatgac 1

tgacaacctcttgaagctaatatcatacgtgac 1

tgacaacgatatttttttggatcctccgttgac 1

tgacaacgtataaagaaaaacgaacagcctgac 1

tgacaactgtatcacagaggaaaaaatcttgac 1

tgacaagaattgaagaaactatcgaggcttgac 1

tgacaagattacactttttcaaaaatttctgac 1

tgacaaggctgcaaaaccagagatttccttgac 1

tgacaaggcttcaaccacgctcaaggaaatgac 1

tgacaagtgagaacgtatacaaatggtgttgac 1

tgacaagttgagctaattaataattagtgtgac 1

tgacaatactccggagctccgatgattcttgac 1

tgacaatattagtgatatgcttaatttgatgac 1

tgacaatcacgagaaagagatttatcatgtgac 1

tgacaatgacacacccagacagttatcaatgac 1

tgacaatgctggactcatcattgaggctttgac 1

tgacaattaaaagaactttgtctttgtattgac 1

tgacaattagtatttacagtttatagttatgac 1

tgacaattatgatttaagagcaaagatagtgac 1

tgacaattggcttgaagatacaaacacactgac 1

tgacacaaaaagagtctaaatttacacactgac 2

tgacacaaaatgtactgtaggagaactagtgac 1

tgacacaaacaatacatatgtcctacatttgac 1

tgacacaagagatgaaagttgtctaaacatgac 1

tgacacaattatgatgtcaagcgggcttctgac 1

tgacacagcttttggacacttctgatgagtgac 1

tgacacagtttattttcattttaatagattgac 1

tgacacataacagtaatttatgttttacttgac 2

tgacaccaaggaatggttggacaatacaatgac 1

tgacacccgaaaagataaatgtattttagtgac 1

tgacaccctgtttttgtggggggtgtgagtgac 1

tgacacccttgcaatgcaaatctctgttatgac 1

tgacaccgactttggacttagttgactaatgac 1

tgacacgaatattcttcatggtgttgcaatgac 1

tgacacgcgtcaataggaaaaatatagtttgac 1

tgacacggcaccagtttattggggttttgtgac 1

tgacacggcactcttaaaaataaacacggtgac 1

tgacacggtgccgtttaacttggcatctttgac 1

tgacacgtcttaagcttatctcttttttgtgac 1

tgacacgtgcgtcaatccaaccgttaaattgac 1

tgacacgtgtcggtaatttagctccggtttgac 1

tgacacgtgttcgtcttcaaacggttcgttgac 1

tgacacgtgttgtactcttggggtcgagatgac 1

tgacactattgattttctgattttttttttgac 1

tgacactcactggactacttgtagaagagtgac 1

tgacactgtttgtataagacttttgattatgac 1

tgacagaagttcattaaatcctaaattcgtgac 1

tgacagagaaaatgtatgatccatgagagtgac 1

tgacagagaagctcggatctaaatctacatgac 1

tgacagagacggggttgaccttcgggtcctgac 1

tgacagataagtggaacaaaatatgaggatgac 1

tgacagatactatattgcagcaaatttggtgac 1

tgacagatatcgaaagcccaataattcgttgac 1

tgacagatcagcaaggtaaaaaagcattctgac 1

tgacagatctatcgattccacgaatttggtgac 1

tgacagatttggaattgttgagggtcttatgac 1

tgacagcagaactaaacttgttgcttgcttgac 1

tgacagcatcataagtagacgttgaattatgac 1

tgacagcgtaatggtagtttctatggtggtgac 1

tgacagcgtaatggttgtttctatggtggtgac 1

tgacagctgacgactctactgcccaatcttgac 1

tgacagcttttaactgatgtgatgatacatgac 1

tgacaggagattttggaccagatgatttctgac 1

tgacaggatgaggatttgtattggagacatgac 1

tgacagtatatgaaacccgtactaggccttgac 1

tgacagtgtaaattaagtccactcttctttgac 1

tgacagttgagtaaagaaaccagagacggtgac 1

tgacagtttaaattttaagtttggattattgac 1

tgacagttttcaaaaataaaaagaaatattgac 1

tgacataaactgttctcttgtttgtttcttgac 1

tgacataaagattagccactctcctaccgtgac 1

tgacataacatcagcgtcccaaaataacttgac 1

tgacataacggggaacttgaagtagattatgac 1

tgacataagatattatatagatgtgagcttgac 1

tgacatacatatttgcatctttgaaatgctgac 1

tgacatacctaattcaatctacatttgtttgac 1

tgacatagcattcaaaatgatgttagcaatgac 1

tgacatatttttagttcaattaaatgaaatgac 1

tgacatcacattaaaagatgtcactgggatgac 1

tgacatcatcttcagccttatagatcatgtgac 1

tgacatcatgctagatagagcccgaactatgac 1

tgacatcattacctctcaacatatgtatgtgac 1

tgacatctttacacttcttgaactcgatttgac 1

tgacatctttctgcttattcccatatctatgac 1

tgacatgcatgacttgcgcgtggtatagatgac 1

tgacatgggctttgttattacttattaagtgac 1

tgacatgtactactactgtatagagcacatgac 1

tgacatgtattaactaatttattatattttgac 2

tgacatgtcatctcattatctcaacactatgac 1

tgacatgtgtagtgtccacttttgaactttgac 1

tgacattaaattcgtgttagtctttttcatgac 1

tgacattaactacaacactctcattgtgttgac 1

tgacattaatctaaacaacgatatattaatgac 1

tgacattaatttgtgtagatatgtatgtatgac 1

tgacattacacatcctcaacaaatcatcatgac 1

tgacattactttatcgggattgattcatttgac 1

tgacattagaaacagcactagtgtttgattgac 1

tgacattagcaacatatacattatttgtgtgac 1

tgacattccatacttgaccgttagagatttgac 1

tgacattggtagttcctgtgctccctctttgac 1

tgacattgttatcagaaatcttggtggattgac 1

tgacatttaaattgtgtatataaatgtcctgac 1

tgacatttaggataaaccaccggtccatttgac 1

tgacatttcacttttgttcctcttaaatatgac 1

tgacatttggttttatactttagttccgttgac 1

tgacattttagaataaatagtcagcttgttgac 1

tgacattttagtctgttgtgatgaaagtttgac 1

tgacattttcaaaatcgtctgtgcttcattgac 1

tgacattttgttttaagaaacttcctagttgac 1

tgacattttttttttttgaacatatcgtttgac 1

tgaccaaaaacaaaaacttagaggttatttgac 1

tgaccaaaaacaaaaagagcacgattaaatgac 1

tgaccaaaaagttgaaaatgacattttcttgac 1

tgaccaaaattgtcattgttaaagaaatatgac 1

tgaccaaaggcccaccaaatcatgggccttgac 1

tgaccaaattgtgacttgtgagttgtgagtgac 1

tgaccaacttgtgattcttgcatcaccaatgac 1

tgaccaagactatggatacaacgtcgttatgac 1

tgaccaaggcaagataagtatgctttttatgac 1

tgaccaagtaattctttatgattctatattgac 1

tgaccaataaaaaccaaacacgagacttctgac 1

tgaccaatagtgtattgtcacgtatcatgtgac 1

tgaccacatactttcttaaaactatttgttgac 1

tgaccacatgttacaactaatgaatttattgac 1

tgaccacatttgtagcaagatacaacccatgac 1

tgaccacctcgagtcacatcacagagccttgac 1

tgaccacgtcttatatgatatggtttctatgac 1

tgaccactagtatttatttagggggtgattgac 1

tgaccagaaaaactaacaaactaggttggtgac 1

tgaccagccgtccgggcacgtgatccatctgac 1

tgaccagctaaatacaagtcaactacggttgac 1

tgaccagctcgaagagaagatttctactttgac 1

tgaccagggaacatatcaaattaccatcatgac 1

tgaccaggtacttctttatgattctagattgac 2

tgaccagtgccacttgattgatatatagttgac 1

tgaccataatccagattttaatttatctttgac 1

tgaccataattaatttttattttcatatttgac 1

tgaccatatatgcatcaaaaggaacttcttgac 1

tgaccatatctaagtgcatctgctagttttgac 1

tgaccatctctgtttttagattgtgtcaatgac 1

tgaccatgaccttaatatagtttttttgttgac 1

tgaccatttaatccgctaattaatcaacttgac 1

tgacccaaaaaggttgtggtcctgtaacttgac 1

tgacccaaatttattcagtaataaacttttgac 1

tgacccaacgtcccaattcatccgtgttctgac 1

tgacccacactcatatggaccctacataatgac 1

tgacccaccattagagagcttgtgagttgtgac 1

tgacccagcttcgggcaagttttcgcttttgac 1

tgacccatctctaaaacataagatatcaatgac 1

tgacccgcctgagagatcttctgaagctgtgac 1

tgacccggcttcataaaactcattacaattgac 1

tgaccctactgtttatgactcattcatgatgac 1

tgaccctataaataatatgataatttccttgac 1

tgacccttgaaatttcatttattatatattgac 1

tgacccttttacagttttcttctgcatgttgac 1

tgacccttttgtgtctcacaagcttgcactgac 1

tgaccgatacattattgtggaatgtggagtgac 1

tgaccgattgacgaattttttctgtgatatgac 1

tgaccgccatttaacacaaatcaaaatgttgac 1

tgaccgtaccaactaccaaaccaacgaattgac 2

tgaccgtctagactgtttttaagaacatatgac 1

tgaccgtgaagacacaatattacgttttctgac 1

tgaccgttattatctgtctcgtctctctctgac 1

tgaccgttcaagcaaccccgtttcccctttgac 1

tgacctaaaaccacgtacgtgtaggatgatgac 1

tgacctaacttgggaagactacaaatcaatgac 1

tgacctaagcttccaacaacaacaaaaactgac 1

tgacctagagagtagagactttgtccagttgac 1

tgacctggggcagtttcgagaattccttttgac 1

tgacctgttttacatgatttcaagaggcttgac 1

tgaccttagaacaacaagggacacatacctgac 1

tgacctttacgatagattccaactcattttgac 1

tgacctttcctttttagagagtccttctttgac 1

tgaccttttctgtatgctattttttacgttgac 1

tgaccttttcttacaaaaatggtcggttttgac 1

tgaccttttttttttttcaacattagctgtgac 1

tgacgaaaatacagagccgttggatttgatgac 1

tgacgaaacgattctcggcccattggatttgac 1

tgacgaagatgtgaagtagacctagaagatgac 1

tgacgaatcgaacggtttatcagctcttctgac 1

tgacgaattttttctgtgatatgaccgattgac 1

tgacgacatgtctatttgggaaacaccaatgac 1

tgacgacattccctctccgccacaacatttgac 1

tgacgacctaaaatctgctttagttaatttgac 1

tgacgacttggtttgcatcaagataaaaatgac 1

tgacgagagaatacaggagaagtgggaaatgac 1

tgacgagagcctctttcatttctcaacgatgac 1

tgacgcaaaagacaatattgtgatagacgtgac 1

tgacgcaaaagacagtattgtgatagacgtgac 1

tgacgctaagtgattcgtccaatcatgagtgac 1

tgacggaccaatcggtaggaaacacgtggtgac 1

tgacggagctgcttgattgagatctactttgac 1

tgacggatttttagtcaaatcctcaaccttgac 1

tgacggcccatttaaccttttttttttcctgac 1

tgacggcggagcattagggttttagaggatgac 1

tgacggctatttcttaatattaatcttcttgac 1

tgacgggacaatgtccccaagaaagaatctgac 1

tgacggtagatgaaacgtccttgatgctatgac 1

tgacggtcaaagacaaaaatggttccatttgac 1

tgacgtaaaccataatgctaagtgatttctgac 1

tgacgtatatacccctgtaacaaactttctgac 1

tgacgtatatttccaccttcacgaaaaaatgac 1

tgacgtcagcatatttgaactctctctcatgac 1

tgacgtcgttcaacggcgggttgatcttatgac 1

tgacgtctatatacgcgcataaaatcacgtgac 1

tgacgtggaccaactggtatgggtagtggtgac 1

tgacgtggatatgccgccaaataaacgaatgac 1

tgacgtggcttcttctgattggtcacatttgac 1

tgacgtggttataaagcagtaatgactcttgac 1

tgacgttaaataacaaaccctggagtacctgac 1

tgacgttaaggttgagaatatgcaaaggttgac 1

tgacgttttacactctgactctccatagttgac 1

tgactaaaaaaatatgtatagtaagctagtgac 1

tgactaaaaatggaaaagcatgcaccatgtgac 1

tgactaaaactaaaaccaaaagatcaaagtgac 1

tgactaaaactaaacccaaaagatcaaagtgac 1

tgactaaaataccccttgccacgtcagtctgac 1

tgactaaaccaaaaactatgtcaacagattgac 1

tgactaaactaaatctgatgaaatatttttgac 1

tgactaacttttaaaacataattattcgttgac 1

tgactaagaagaagcaggcacatccgccttgac 1

tgactaagagcatctgtgagagtaaacattgac 1

tgactaatcctgcgtactaattaaaccaatgac 1

tgactaatccttctcggaaaatcatttgctgac 1

tgactaattttaaaaacgtgattattcgttgac 1

tgactaatttttaaaacatgatctttcgttgac 2

tgactaattttttaaacatgatctttcgttgac 1

tgactacaaagtctaaaacttttgacttgtgac 1

tgactacaacaagagtgggccgaggccgatgac 1

tgactacaattacgattaaaataagcttatgac 1

tgactacattcacgcttttggatataatttgac 1

tgactaccatttgaaacacgagaatccgttgac 1

tgactacgtggactcactagacttgccattgac 1

tgactactgagtgtataaatattactagttgac 1

tgactagaaaatatagattatatactatgtgac 1

tgactagaagaaatatagcttgatggaattgac 1

tgactagagttttaaccaaaattcaatactgac 1

tgactagcatacaattatctaaatgatgatgac 1

tgactaggttgatgaattttaattgttactgac 1

tgactagtagacaaacacgtctaattgggtgac 1

tgactatatcagtaatcttatcagcaatatgac 1

tgactatcattctcctaagtcttgaacgttgac 1

tgactatcattttataaaaaaaatactattgac 1

tgactatgatgtattgtcaaaaaaaaaaatgac 1

tgactatgattggcgattgcaactttaaatgac 1

tgactattcccactaaaactacatataagtgac 1

tgactattgtattcttaggaattaatgcgtgac 1

tgactatttattgtgcatgggaaagattttgac 1

tgactatttgccttggtcaacgatgctattgac 1

tgactatttttaggaatcatctagacttttgac 1

tgactattttttttgtaaaactttatgcatgac 1

tgactcaaaagaaagatttgtaatagacgtgac 1

tgactcaaacatgtaaattctcggtaacttgac 1

tgactcaacccttagcttgggagactctctgac 1

tgactcaatttcaacaacctcttgagtcttgac 1

tgactcaccaaataaaacgcgtcaaccgttgac 1

tgactcactcacaaaaaactacaaaacaatgac 1

tgactcagctgggttctgtggggaaagcgtgac 1

tgactcatcgtccaactgcttcaatctagtgac 1

tgactcatcttttcatttactgctatcattgac 1

tgactcattgccatatctgggaagagaattgac 1

tgactcatttgaacattaatataatttgatgac 1

tgactccatttagcaatgacgaactaaaatgac 1

tgactccgtccacgttcttcttcactgtttgac 1

tgactcgaaaaggttgttatacgtggagctgac 1

tgactcgccaggccaggaagaaaccggaatgac 1

tgactcgttatctttggtaagtcctcgtatgac 1

tgactctaatactaatgcactaggacacttgac 1

tgactctaatggtggagctacatatatcgtgac 1

tgactctcaactagtcatttcaaaataattgac 2

tgactctcgtaattgatggctgactagaatgac 1

tgactcttataattgatggctgactagaatgac 1

tgactcttctttccaaaaaggattagatctgac 1

tgactcttgattacagaagaataagaaactgac 1

tgactctttagtctgaaaaacaaaatacatgac 1

tgactgaaacaccaatgaacttgtgtgtgtgac 1

tgactgacaagctttgatagtatatttaatgac 1

tgactgagatgcacaaaagtgggaaaacgtgac 1

tgactgatgaggctaacgaaagtctaagatgac 1

tgactgattcttccgggaagcaatcgatctgac 1

tgactgcatcgttttacttttgttctgggtgac 1

tgactggttattgaaattgaatttgatcctgac 1

tgactgtagatataaacttttattttttctgac 1

tgactgtcacaattttgattgttgataagtgac 1

tgactgtcattacaacataattccaaatgtgac 1

tgactgttttccatttcaagtaaattaaatgac 1

tgacttaaataaggtagttgaaaatatcttgac 1

tgacttaactccattccattctcctgctatgac 1

tgacttaattagacacttgatttcatatttgac 1

tgacttacatcttgttccatttttctttttgac 1

tgacttacgattatgtttgggtagaatgttgac 1

tgacttactccactattgtcgctcatgagtgac 1

tgacttagctattcagagttcataattagtgac 2

tgacttataccaaattagatttgaatatttgac 1

tgacttatccatctcatcatcagaatcaatgac 1

tgacttatggtccaacctggttaacccgatgac 1

tgacttcaaataaaagctataactttagttgac 1

tgacttcaaataaaattaataactttagttgac 1

tgacttcaattgcttttccgattcagatctgac 1

tgacttcacacgcaagaagctaaaatacttgac 1

tgacttcatgataatttcatatcatctaatgac 1

tgacttcgagttctccaaatcctatagattgac 1

tgacttctatgtgtattgtccactgtgtttgac 1

tgacttcttaaaactctcataagttttcatgac 1

tgacttgagagaagaagagtgaaatccagtgac 1

tgacttgaggatataataaataaatagattgac 1

tgacttgatccacaagccattgtcttgggtgac 1

tgacttggagctatgagggaacactaatgtgac 1

tgacttgggagaagatagtgaaggcacggtgac 1

tgacttgtatacggctgttcaacgactcttgac 1

tgacttgtgtggcgcgggtaacataaatttgac 1

tgacttgttttagcttaccaataatattttgac 1

tgactttaataaattagcaaaaataagtatgac 1

tgactttaggtggtagctgtgaagcaacttgac 1

tgactttatgtaatttctaaattttactatgac 1

tgactttatgtgatattaaatatgtcatttgac 1

tgactttcaaattaatcacaaattcttgttgac 1

tgactttcaataaaatagcaaagtttccttgac 1

tgactttcaatttaatcacgaattttcgttgac 1

tgactttcaatttaatcataaattttcgttgac 1

tgactttgaaccggtctctcactctttgatgac 1

tgactttgctaaatgataattaatcttcatgac 1

tgactttgctgctgcttcatagacttagctgac 1

tgactttggtttgttttgatgtcagccgttgac 1

tgactttgtataatgtgataggttgaaattgac 1

tgacttttatcttttttggtcaaattatatgac 1

tgacttttgaggaggcgagattgcggcagtgac 1

tgacttttgataaaatgatcaagttttgttgac 1

tgacttttgataaaatgattaagttttgttgac 1

tgacttttgattaaatagtcaagtttcgttgac 1

tgacttttggattaacacatactacctattgac 1

tgactttttaatttttctgtgtggccaggtgac 1

tgactttttatttaatctcgaattctggttgac 1

tgactttttcgtggaaatgacgtaactattgac 1

tgactttttggccttatagggttccaatttgac 1

tgacttttttgtgtagaaaatggtaaaattgac 1

tgactttttttcaactacgtctttttatttgac 1

tgactttttttgttgttaaaataaattgttgac 1

tgactttttttttttttttaattattttttgac 1

tgactttttttttttttttttaagaatattgac 1

26 : 523

tgacaaaaaaggtttagatgtgtcattagttgac 1

tgacaaaaaatatggatgagagaatgcctttgac 1

tgacaaaaactcatctaattgtttgaatggtgac 1

tgacaaaaagacacctcatgggcttactgatgac 1

tgacaaaacccaaaaaaaaaagtactcacgtgac 1

tgacaaaacctattttatactatagttttttgac 1

tgacaaaagatccaaaagtgttgacttcaatgac 2

tgacaaaagatccaaaagtgttgacttcagtgac 1

tgacaaaagccggtttatttgtgaaattaatgac 1

tgacaaaagtcccatactttgaacaatctttgac 1

tgacaaaatatccaaaagtattgacttcaatgac 1

tgacaaaatcaaactcgttgaactctaacatgac 1

tgacaaaattcttaattacttacagatccgtgac 1

tgacaaacaaacttcaaaacactaaaaacatgac 1

tgacaaaccatgtatatatactataacatgtgac 1

tgacaaacctaggtagtatgacagaccttgtgac 1

tgacaaactctttagttttttggtcaaccatgac 1

tgacaaactttccagtagcatcgaggacagtgac 1

tgacaaagatatatacgcgtctgtttttgttgac 1

tgacaaaggggtccggtcccaatgaaaatatgac 1

tgacaaatagtgttttattctaattttggatgac 1

tgacaaatatcttgacatacaaaaaagttttgac 1

tgacaaatgatattagagtatcggtaaaattgac 1

tgacaaatgattcattattctcgtcgtatttgac 1

tgacaaattcttttcttttggacagatatgtgac 1

tgacaacaaaatgttgattcacttgaaaaatgac 1

tgacaacaacaatttcaaggcaagtcaagttgac 1

tgacaacaatatcaaaatcttgagtgcaaatgac 1

tgacaacaatcaaattttacaaaatgtgtttgac 1

tgacaacccccaaaaaaagaaaatgtaacgtgac 1

tgacaacgaagatggcgattgcgatgacgatgac 1

tgacaacggcgtgccgtgatgtcggagggttgac 1

tgacaactaaagctgaagatatcctagtattgac 1

tgacaactgcggaaagtctgtagtctggtgtgac 1

tgacaacttgacgtatttctttgaaccgtttgac 1

tgacaactttccactggcactaagataaagtgac 1

tgacaagaaagaagatgttaatgtagaacatgac 1

tgacaagaatttatgctaatgttaaatgtttgac 1

tgacaagaccaccttcatcatttattgtgctgac 1

tgacaagacgaacttattgtacctccctgatgac 1

tgacaagcattttttttttcatgaaacatttgac 1

tgacaagccgtttaagacatgagaatgtgttgac 1

tgacaagttgagattgagttgccgacgacttgac 2

tgacaagtttcttttcattttgagataaaatgac 1

tgacaataatctctatcatctactcaaatgtgac 1

tgacaatagaaaattcaaagtaaacctatatgac 1

tgacaatcccgcatgcagtcatatgcaccatgac 1

tgacaatctttttagttgttcaaaattgtatgac 1

tgacaatgagcaagttttgctcaaatattttgac 2

tgacaattatattcgagtgaacttaatcagtgac 1

tgacaattgtctttcattcatttcatcatttgac 1

tgacaatttatttgtcgacgtaactagagatgac 1

tgacaattttaaatggagacgtaagatgtatgac 1

tgacaattttaaggtcagtcccatcttttttgac 1

tgacaattttgtacaaaaaaaaaaagggattgac 1

tgacacaatattattagtccacatcgtatttgac 1

tgacacaatcttctatcatcttttgaagattgac 1

tgacacagaaatgttatcttatagtcaatttgac 1

tgacacagataccatgtttctggcagaatttgac 1

tgacacatatcaacatctgaacgatagacttgac 1

tgacacatccttttttttttgtcaaacatttgac 1

tgacacatgtaaacacctgatcgtttgacttgac 2

tgacacatgtaaacatctaatcgtttgacttgac 2

tgacacatgtaaacatctgatcgtttgacttgac 4

tgacacatgtaaacatctgatcgtttgatttgac 1

tgacacatgtcaacaattgatcgatataattgac 1

tgacacatgtcaacatctgatagataaatttgac 1

tgacacatgtcaacatctgatcgatttatttgac 1

tgacacatgtcaggtatatcattgagatgttgac 1

tgacacatgtttttcttgtgaacaattaactgac 1

tgacaccttggtagtcaccaagggcacgcatgac 1

tgacacgtctcacatctgtcagttagattatgac 1

tgacacgtgtcaagtttatcgttcagatattgac 1

tgacacgtgtccataaggtttcgttctccgtgac 1

tgacacgtgtttggtgagaaactgaactcctgac 1

tgacacgttttttgagcaatgccatagagttgac 1

tgacactaaaaaggtttattattgcaaaaatgac 1

tgacactagttataatacacgttaaatatttgac 1

tgacactcaaattctgttaaaatatacacatgac 1

tgacactcaggatttgaaaattaaaagtagtgac 1

tgacactgaaatgtcttgcgaagaaggaggtgac 1

tgacactgatatatgtagtcataatgatattgac 1

tgacactggtaaacatctaatagataaacatgac 1

tgacactgttctttcttaatattaatgttatgac 1

tgacagaagatgctccgagtatgaaggatttgac 1

tgacagaagcatatgacttccacatatatttgac 1

tgacagagaagagccttaaagaacctaaggtgac 1

tgacagataagaataaataatgaaacattttgac 2

tgacagcagcgaggagtctgattttggagatgac 1

tgacagcatttattttgtgcaacaaataattgac 1

tgacagccatccatttggaagctcacatgttgac 1

tgacagcgccgtttaccatttttataatactgac 1

tgacagcgtagctgatatgtgttcgtttgttgac 1

tgacagctatacatcttactctcgccattgtgac 1

tgacagctccgacgatggcagtgactccagtgac 1

tgacagggcttatgttcttgaattgcttcgtgac 1

tgacagggtttggaggaagatccttggctttgac 1

tgacaggtgcagtagtgggtcctaccaagttgac 1

tgacagtaatttactattatattttgctattgac 1

tgacagtatatgatatagtgatactcaatttgac 1

tgacagtgagtttcgtcttcgtgcttatagtgac 1

tgacagtgattgggtatgtcaaaacaggggtgac 1

tgacagttgaacagtatattctcaaaatcatgac 1

tgacagtttttcaactggttgacaattctatgac 1

tgacagtttttttttcattttagcttgttatgac 1

tgacagttttttttttcttttttttttttttgac 1

tgacataaataggaagttgggtacgctttttgac 1

tgacataacaaatctattaatatatttagatgac 1

tgacataactttttgttttttgtcaaactgtgac 1

tgacataagaattgatgttcaattgattcatgac 1

tgacataagtagcttttgacttttgatgaatgac 1

tgacataataatggtttggttctcccaacatgac 1

tgacataattacggcattgcgtagccaccatgac 1

tgacatactatagcaaatgataataattgatgac 1

tgacatatgcaattcgatcatgatggtttttgac 1

tgacatatgtcaacatctcaatgatagacctgac 1

tgacatatgttaacatctcaatggtatacctgac 1

tgacatattgacatgcttcagtatttgatatgac 1

tgacatatttcattttcttgtgtgatgatgtgac 1

tgacatcactggccgaagaacgttgactgttgac 1

tgacatcgatgtatatttgtgatgcaggtgtgac 1

tgacatcgggtagtatatcttgaacatccatgac 1

tgacatctagtatatacacttgggttgtgatgac 1

tgacatcttttaatgatgtagtttatgcaatgac 1

tgacatgacattatatattaaatattatcatgac 1

tgacatgatcagatgtggaggagatttatttgac 1

tgacatgcggaagatcgataaatggcatcatgac 1

tgacatgcttagaagacaatctctccgccctgac 1

tgacatggaacagagaggagaaggtgaagatgac 1

tgacatggcagattttgtttcgaatcgtcatgac 1

tgacatggcgggtttggaaattatattacatgac 1

tgacatggcttacttgctgctctacgttgatgac 1

tgacatgggttgggcttcatgaaccctgcttgac 1

tgacatggtcgtcgtattctttacaatctttgac 1

tgacatggtgagacatagctgtctctcagttgac 1

tgacatgtgcgaatatcttattaataaatttgac 1

tgacatgtgtacttatcattaattatagtttgac 1

tgacatgttatacttcagtgaccggttgagtgac 1

tgacatgttgatcactactgatttttccgttgac 1

tgacattaaggcccatgggcgtttttctaatgac 1

tgacattacatagtgtggtcacgatacctatgac 1

tgacattatcatcatttgctgcaggccagctgac 1

tgacattatgttctcttttaagattctatatgac 1

tgacattattaatttgtttggtaaaagctttgac 1

tgacattattagaacattagacttctaatatgac 1

tgacattatttatgaagcaaaagataattttgac 1

tgacattcacaagcattctaaacatatctatgac 1

tgacattctatttgattttatcctacggcctgac 1

tgacattctctcccaaagaagaagaattaatgac 1

tgacattcttttgaagtaaacagaaataattgac 1

tgacattgaattgtattgtattgattgcattgac 1

tgacattgttcttgttttcctcagaggatgtgac 1

tgacattgttgtttttgaataaatcagaggtgac 1

tgacatttaccttgttgactttgtaagcaatgac 1

tgacatttctccagctatattaagagcttatgac 1

tgacatttcttttcaaactgaaattggagttgac 1

tgacatttgtcacatttgtttgaaaatccatgac 1

tgaccaaaatgtagcgtttctagatgggattgac 1

tgaccaaacaaatggaaaatcttttggccttgac 1

tgaccaaactctttttatttctttaaactatgac 1

tgaccaaagaaacagtacgattttgcaagctgac 1

tgaccaaagatagagagctccggagctaagtgac 1

tgaccaaagatgcaacctagtctcatcacctgac 1

tgaccaaagattccacaggtcggacagatatgac 1

tgaccaaagcctcctagctcctcctatttttgac 1

tgaccaaatcaaacaacctcttaacttctttgac 1

tgaccaaatcgacatttgactacttgagcgtgac 1

tgaccaaattaaatcgcattgcattaggaatgac 1

tgaccaacaagggcaatgaacccatcaatatgac 1

tgaccaaccattttacacatagaaaaacgttgac 1

tgaccaaccggactatccaaccaagacatttgac 1

tgaccaagaccattcgaatattggaagaagtgac 1

tgaccaagcaacaaagacaaatctattagatgac 1

tgaccaataaccattaacgaattgaattactgac 1

tgaccaatgcttaaaaaaactaaataattgtgac 1

tgaccaatgttgatcgacgtttaccgatgttgac 1

tgaccaatttgaagtgggtattagaaaggttgac 1

tgaccacaaacactcatttccaacagaacatgac 1

tgaccacaatctcccactacatgacacagctgac 1

tgaccaccggttcatagtagcaccctaacttgac 1

tgaccacttaccaggtgtgtttgctacgcgtgac 1

tgaccagaccgtacacctacaggacttgcttgac 1

tgaccagactagaagcctttgaagcgactatgac 1

tgaccagcacaaatctgatgaaagggctagtgac 1

tgaccagccattttacaaatacaaaaacgttgac 1

tgaccagctgaaccttttagtttagtgttttgac 1

tgaccaggccaaaatccacatgacgttatctgac 1

tgaccaggttaaaattcacacgacgttatctgac 1

tgaccagttctacaagaagacaaggcaacatgac 1

tgaccataaccatgagcgttacccacctggtgac 1

tgaccatacagtatagtcactatctgagattgac 1

tgaccatattttggtcaaaaataatgagaatgac 1

tgaccatccattttaacaaaaacaaaacgttgac 1

tgaccatgtataatactataacaacacaagtgac 1

tgaccattcaatacctggttgagggattcttgac 1

tgaccattggattagcgattagcgtgtaggtgac 1

tgaccatttttcttcttacaaaggttgagatgac 1

tgaccattttttgaaagttgggggattaattgac 1

tgacccaaagttctaaatctcactaaatattgac 1

tgacccaagtcgctgaaatgtttgaccatatgac 1

tgacccaattctttttgtcgcgatgatttttgac 1

tgacccataatctcttcttcttcttctttttgac 1

tgacccattaaactcatactttttttgtaatgac 1

tgacccccttaccctccatgtaaatcttaatgac 1

tgacccgagtgttataaccatcaattgtcctgac 2

tgacccgatttgatttttcagcctcaaatctgac 1

tgacccgccatttaacacaaatcaaaatgttgac 1

tgacccgcggacttatctgggcctgaactttgac 1

tgaccctaaatcttttaagttttaactagttgac 1

tgaccctctttctcatttatttgcttaagatgac 1

tgaccctttctcttctttagctcattgccgtgac 1

tgaccctttttttttggtgaaccaaagctatgac 1

tgaccgaagtttcgggtccagcgagtgggttgac 1

tgaccgaccattttatacatctcaaaacgttgac 1

tgaccgatcatttcacacaaatgttattgttgac 1

tgaccgattttattgttatcttcttcatgatgac 1

tgaccgcctctcattctctagctttgtccatgac 1

tgaccggatattttacacatatataaccgttgac 1

tgaccggccatttcacataaacttaatcgttgac 1

tgaccggccgtttcacacaaatattattgttgac 1

tgaccggcgttggagtatctggtggaggcttgac 1

tgaccgggtttatgtgttgttggctccgtgtgac 1

tgaccggtcattttatacatataaaaacgttgac 1

tgaccggtcgtttcacacaaatgttattgttgac 1

tgaccgtggagtctcttacacgtttggccctgac 1

tgaccgttggatgagaaaacgatgttcatatgac 1

tgacctaaaactgctacgacatcgttttagtgac 1

tgacctaaaccatagccgtaaatcggaaaatgac 1

tgacctaattactttggttgcctcacgttctgac 1

tgacctaatttaattacacacaaatttatttgac 1

tgacctagctttgactgagtatcaccacagtgac 1

tgacctagttcgataataaaacgaagcttatgac 2

tgacctatattttgtgttttattactagaatgac 1

tgacctctctctgacatgaaactgtagcattgac 1

tgacctgaatgtttgcttcgatttcccgattgac 1

tgacctgaccaccactccttccgatcatcatgac 1

tgacctgcttgctcttcctgctgttgactgtgac 1

tgacctgtcacgtcataacaaggtcgtttttgac 1

tgacctgtttagatgtaattctttggttgatgac 1

tgaccttctgctgaaaagtggttcggatagtgac 1

tgaccttgatccatccaacgttccctatactgac 1

tgaccttggttagatttagctggccaaagttgac 1

tgacgaaaattctacacgcgaaagctgttttgac 1

tgacgaaattcacacgtgtgtcttaaacagtgac 1

tgacgaagagatactcttccaactcccacgtgac 1

tgacgaagatgtgggttttcatattgcccatgac 1

tgacgaatcgagactcacctttttgttccttgac 1

tgacgaatttctcaacgaaacacggtagtgtgac 1

tgacgacaaaagcaaatttcttcttcttattgac 2

tgacgacacacttttgtttgttatgagttatgac 1

tgacgacataaatttttcaaaacaaaaaaatgac 1

tgacgacttcgatcctgctgcgtcttagcctgac 1

tgacgactttatcgaatgttttgtagttaatgac 1

tgacgagaatcgctgatcacagcaccagagtgac 1

tgacgagagggagagagcttgaagatccggtgac 1

tgacgagatctcggatgcttcgattacacctgac 1

tgacgaggttcgggttatagtcacgaatattgac 1

tgacgagtaagaactgagatgagacagaactgac 1

tgacgataaaacgacggcgtatttgcggtttgac 1

tgacgataactagcagagaatgtaaggatttgac 1

tgacgatagcaatagtttagatgaagaaattgac 2

tgacgattacacacattcttaaagctgtgatgac 1

tgacgcaaagtgatgaatttacataaaatatgac 1

tgacgcacgtgaatgtgtgtgtaaatactttgac 1

tgacgcacttggataaacatacatgtgcaatgac 1

tgacgccaaaaaaaatccaatctgtcagagtgac 1

tgacgccatgcatggtgggtacagtaactatgac 1

tgacgccccgactattctagctgttacaagtgac 1

tgacgcgaaaatgttgatacacttgaaaaatgac 1

tgacgcgagtcttacccacgaccctttctttgac 1

tgacgcgattcatatactatcttttttctttgac 1

tgacgcggaacagagcaaaataatgatttttgac 1

tgacgcttactatatatgttttcggatacgtgac 1

tgacgcttttacactttttcaaaagtgatttgac 1

tgacggaagtgttcgtaacaaacgacgtcgtgac 1

tgacggaataattcttcgatgttttcgttttgac 2

tgacggaccaaaataaaccagagattttcctgac 1

tgacggactgtggtgtgatggtggcggcagtgac 1

tgacggattcaatcttgcatgcaaaccacttgac 1

tgacggcatgaaaaggaaacatcgaagtgatgac 1

tgacggcgaaaactcctgatttgtttattttgac 1

tgacggctttccaaccaaaatatttcgttgtgac 1

tgacgggaacctcgaataatttgaaaaaattgac 1

tgacgggacccttagcataaccaaaatcactgac 1

tgacgggcctccagcattttgtacagtcgctgac 1

tgacggtaacaggcctgggcggtgaatgcatgac 1

tgacggtaatatctcctttaccggctgtcatgac 1

tgacggtagcggttgcggctcctgtgctcgtgac 1

tgacggtagtaatagtccttaatgacatattgac 1

tgacgtaagcgcttttgtcatgtacatcattgac 1

tgacgtacatcaatgttttctgatttttactgac 1

tgacgtactctaaaattcagaaaaaagaaatgac 1

tgacgtactgacatagacaaagtctacgcttgac 2

tgacgtatctaaaaatttgctttatgactttgac 1

tgacgtcaacactagaaattagattgtgactgac 1

tgacgtcaccgactctgttcaaaatgtcgatgac 1

tgacgtctcgtttgatctacacatattgtttgac 1

tgacgtgcttattagtcttgtacaaacccttgac 1

tgacgtggcagattataacggctactttgatgac 1

tgacgtggcagtgcatttaattatttgatttgac 1

tgacgtgtggctgttctgacattaacacattgac 2

tgacgtgttttaatgtgattggatggaaaatgac 1

tgacgttacttgtaatcattgtccaatcagtgac 1

tgacgttacttgtagtcattgtccaatcagtgac 1

tgacgttcttgtggactccattagagaaaatgac 1

tgacgttgacgatagcgataatggtggggttgac 1

tgacgttgactttgaagacgatgaagattctgac 1

tgacgtttagttgtttaagtatgaacgacatgac 1

tgacgttttaaaattacttcagtatatatatgac 1

tgacgttttccttagccgtctttgtaactctgac 1

tgacgtttttggtggctggagatccgatcgtgac 1

tgacgttttttttagctaatggtgatctcttgac 1

tgactaaaagagtcaaaggatgcaatcttgtgac 1

tgactaaagttgatggaaagaaggaaatggtgac 1

tgactaacaaataaaacatgaactttgcgttgac 1

tgactaacaatcattaacaatcataatgaatgac 1

tgactaacatatttaaacaataaaaatgtatgac 1

tgactaacatttaaaacacgaactttgcgttgac 1

tgactaaccgacctcgtaatccgatgaacatgac 1

tgactaagaagagaagaaaatgaagtttgttgac 1

tgactaagtggctgtcaaaaatcaactagatgac 1

tgactaataaaaccctacaagtccgttctttgac 1

tgactaataaaattcattaaaattcaaatatgac 1

tgactaatcagatcttgggcacaaaaccattgac 1

tgactaatgaaaatcaaccactatttattttgac 1

tgactaattttctaaatatgttctgaaaactgac 1

tgactacaagagagcccggcatttcgaggttgac 1

tgactacacatttatggcataacaatagattgac 1

tgactaccatttaaaacatgaaaaattcgttgac 2

tgactaccatttaagacataaaaatatcgttgac 1

tgactaccatttaagacatgaaaaattcgttgac 1

tgactaccatttaagacatgaaaaatttgttgac 1

tgactaccatttaagatatgaacaattcgttgac 1

tgactactattaaataacacattattttcttgac 1

tgactactttacaaattatgaatagtaaagtgac 1

tgactactttcctaattaagtttgaaaaaatgac 1

tgactactttcctaattaagtttgaaaaagtgac 3

tgactactttcttaattaagtttgaaaaagtgac 1

tgactactttgcaaattatgaatagtaaaatgac 1

tgactactttgcaaattatgaatagtaaagtgac 3

tgactactttgcaaattgtaaatagtaatatgac 1

tgactacttttccaattaagtttaaaaaagtgac 1

tgactacttttccaattaagtttgaaaaagtgac 1

tgactacttttccaattaagttttaaaaagtgac 2

tgactacttttctaattaagtttaaaaaagtgac 1

tgactaggattatcacgtttcgaagatgcttgac 1

tgactagtggtgcaacctgctgactccatatgac 1

tgactagtgtttacataattatgaaatacgtgac 1

tgactagttttctctgccacacgaccaaagtgac 1

tgactatagactttcaaacaagttgagtgatgac 1

tgactatagagattttcttcgaagacaaaatgac 1

tgactatatatctatcaccttcaagactcgtgac 1

tgactatattaaactctaactaatatcggttgac 1

tgactatcataaatcagttttcaattctgttgac 1

tgactatcctcaaattgacgattttgcccctgac 1

tgactatccttgtgcagatattaaaatgcctgac 1

tgactatgcttggaaaaactaagaacggtatgac 1

tgactatgtgatgacgttttttcgtgtagttgac 1

tgactatgtgttattgtgttgttgatttgctgac 1

tgactatgttcgctcagacgacgacggagatgac 1

tgactattgagtttgaagtgaaattggaattgac 1

tgactattgtttttgttttggtagtttatatgac 1

tgactatttaagtctaaagaaacgcgtctttgac 1

tgactatttcgtgtatctgaagtcctcacttgac 1

tgactatttggtagatatacacaaataacgtgac 1

tgactattttacaaattataaataataaagtgac 1

tgactattttcctaattaagtttgaaaaagtgac 1

tgactattttgcaaattataaatagtaaagtgac 2

tgactattttgcaaattatgaatagtaaagtgac 4

tgactatttttccaattaagtttaaacaaatgac 1

tgactattttttcaattaaattttaaaaagtgac 1

tgactcaaaactggataatagtaactaaaatgac 1

tgactcaaagacctatcattgtctttagtgtgac 1

tgactcaaataccacaaagcataaaccttttgac 1

tgactcaatgactcatgaccattacaaccatgac 1

tgactcactcacaaagaactacagaaaaaatgac 1

tgactcataacttaggattaataacttaaatgac 1

tgactcataatgaaacgtatcataatggattgac 1

tgactcatgaccattacaaccatgactcaatgac 1

tgactcccatacatttagatttattcgcattgac 1

tgactccttccactgttttaatccaacaggtgac 1

tgactcgaatgtagccaaatttagtttggatgac 1

tgactcgactcatctctagatcaacaatagtgac 1

tgactcgagtaaacaaccactactagtcgttgac 1

tgactcgcttgagatttaattaaaatcggatgac 1

tgactcgggcgactcagacccgtgccgagatgac 2

tgactcggtattgattgaaaatgatgaagatgac 2

tgactcgttcacacgattgtgcaggtatactgac 1

tgactcgtttcaattataaaaaaaaactcttgac 1

tgactctaactaaagtagaaggaaaatatttgac 1

tgactctaatcttaacaaaagtaactagaatgac 1

tgactctacttttaacaaaaataactagaatgac 1

tgactctagcagaaacccactcactgatattgac 1

tgactctagtatctttatctttagctctgttgac 1

tgactctatttagaaaagttgtgtctacagtgac 1

tgactctccaagccccagtcttcatccatttgac 1

tgactctctcttgttacgaccacaaatttttgac 1

tgactctgaccacctctttaactgcgaccatgac 1

tgactctgttccggttctaggagtcaattctgac 1

tgactcttgtttggtcaacttgcagaaaaatgac 1

tgactctttaaagcgtcctctcagaatctctgac 1

tgactctttaaccatgaaaaacgatcgacttgac 1

tgactcttttgagctctaatttatttcggatgac 1

tgactcttttgaggtgtaaataaaatctgatgac 1

tgactcttttgagttataattaaaagcagatgac 1

tgactcttttttttttgcatgtataaactctgac 1

tgactgaaagtgaatattggttgacagacctgac 1

tgactgaagaacatataaagcaagcccacatgac 1

tgactgactaatcttcaaaaatccatctaatgac 1

tgactgagcttgtaagtagtagtattgtggtgac 1

tgactgatcctatcctcattgctcaaactctgac 1

tgactgatcgagttgttggttctacacatgtgac 1

tgactgatctggaccaacccggtgacctaatgac 1

tgactgatttatataacagataaaaccctctgac 1

tgactgcacatactttcaatatcccatagatgac 1

tgactgcagtatgtgaaaaactcttaactttgac 1

tgactgccactctgtctccacatgaaaatctgac 1

tgactgccatttaagacatgaaaaattcattgac 1

tgactggccatttcacacaaatgttatcgttgac 1

tgactgggataccaaaaaaaaactaaacactgac 1

tgactggttgaaacgcagtgcctacatttgtgac 1

tgactggtttgcgcgtttgaactgatgtcatgac 1

tgactgtaaaaattttggttgtaaaaattttgac 1

tgactgtagtataagcaattaagcatctcttgac 1

tgactgtagttaactcttgtagaacccatatgac 1

tgactgtatctccgtcatcaagactcatgctgac 1

tgactgtcgatgaggtaatctcctggtccatgac 1

tgactgtcttttacaaattgggttatcacctgac 1

tgactgtggttctaggagaacatttgcatttgac 1

tgactgttatatccctcaatccttctcgcgtgac 1

tgactgttgatttttctgctgtgaatgtgatgac 1

tgactgttttactgacttgtgagttctgactgac 1

tgacttaataataaaatggttaagttttgttgac 1

tgacttagaccaaaaaaaaggagaagttgttgac 1

tgacttagatcggcttacactgcttagcgatgac 1

tgacttataacaaatttttaaatattgatttgac 1

tgacttatcaatttaatcgctaacttatgttgac 1

tgacttatcgatatcacccatttggtggcctgac 1

tgacttatgaaacttccataaaaatttcattgac 1

tgacttattagaagcaagtccatccctcagtgac 1

tgacttcaaactaatcataaccaaaagttttgac 1

tgacttcaagagcagccaagaacaatcctgtgac 1

tgacttcagcgtggaggttctcgagaactctgac 1

tgacttccacgaaaaggcaaggaagagcgttgac 1

tgacttcgatacaaacctctctttcctccgtgac 1

tgacttcgcatttttaatcgctcattttgttgac 1

tgacttctaatttaacctataagttatgattgac 1

tgacttctaatttaatctataagttatcgttgac 1

tgacttctcaaccacaaataatagcaacaatgac 1

tgacttctgttttaacctttaagtttgcgttgac 1

tgacttctgttttaacttttaagtttgcgttgac 1

tgacttgacttctattattctataaactattgac 1

tgacttgatctctataaaaaatatgagatttgac 1

tgacttgatggtgtgtatttgcctttcgtttgac 1

tgacttgattttttatatgatcaataaggctgac 1

tgacttgctccgatgttgttatagacaagttgac 1

tgacttgcttgtgtgctgcagagctgagtatgac 1

tgacttggccggagattttcttccgacttttgac 1

tgacttggctcttccttcttcacataaagatgac 1

tgacttggtatctagttttgggatttttcctgac 1

tgacttgttagtcccaaaaagataaaatattgac 1

tgacttgttagttcaaaactcacttgatcctgac 1

tgactttataataaaatggttaagttttgttgac 1

tgactttattgtcaatgattattgtcaatgtgac 1

tgactttcatataaagcttgataattttgttgac 1

tgactttcatattaacttatcagaattgtgtgac 1

tgactttcatgatggatgaatccagttgtttgac 1

tgactttccaattcaatgattacactatactgac 1

tgactttccactgccaccgccaccacctagtgac 1

tgactttcgaaataaatgacaagtttttgttgac 1

tgactttctaaataaatatctaatttttgttgac 1

tgactttctgacatttttctctctaaactttgac 1

tgactttctgatatatatatttcacagatttgac 1

tgactttcttataaaacattaactaagatttgac 1

tgactttgattgcgatgattttgtaatgtttgac 1

tgactttgcatatggactcactcttgcctatgac 1

tgactttgctcggcacacaaaagaaaaggttgac 1

tgactttggcggcgggttattagagcttgttgac 1

tgactttgttccttcctcttcatacgtcgttgac 1

tgactttgttttcgaggactatttaggcgatgac 1

tgacttttaatttaacctataatttatcgttgac 1

tgacttttacatctcatgaatgcattgccatgac 1

tgacttttatcattacgtggaaaattagcgtgac 1

tgacttttcatgtattacggctccaccacctgac 1

tgacttttcatttcaatagtcaacttttagtgac 1

tgacttttccagaaaatacaaaagtttggttgac 1

tgacttttctaataaatcttaaacttgtgttgac 1

tgacttttgaaaaaaatgatcaagttttgttgac 1

tgacttttgtcgtcactaaacgaagagacttgac 1

tgacttttgttaagtggtacaaagtggcattgac 1

tgactttttaaaaaaattgtcaagtttcgttgac 3

tgacttttttataaattgtgactagtaaaatgac 1

tgacttttttttaaataaaaagaatctaactgac 1

27 : 385

tgacaaaaaaaaaaggatagagacattgtcttgac 1

tgacaaaaaaattaacaatattaaacgaaaatgac 1

tgacaaaaaatggcgtcttattataaaacattgac 1

tgacaaaaactagtcctctcacgtttcaccgtgac 1

tgacaaaaaggcactaaaaaaactctccttttgac 1

tgacaaaaattaaaaagaaagaaaagactgctgac 1

tgacaaaacatgcaaacacatctcttctttttgac 1

tgacaaaacgtcgtcgttaactttagattaatgac 1

tgacaaaactgtgaactttaattttccatcttgac 1

tgacaaaataatatgattgaaagttgataaatgac 1

tgacaaaatactttgctcaaaaacaaatcgttgac 1

tgacaaaattgtataatttgattttaagaaatgac 1

tgacaaacaaacaaactaacactccaaccaatgac 1

tgacaaacaacattaaaatactatataattgtgac 1

tgacaaacagagaccacattaaacagttacttgac 1

tgacaaacatattcaagtttattctacacagtgac 1

tgacaaacgtatgcgtttgacataatcggaatgac 1

tgacaaactcaaatttgaatgtaggtaccagtgac 1

tgacaaacttaaactattacataagattgtttgac 1

tgacaaagaacgattcaaattctggacgatatgac 1

tgacaaagattgttgtataatcttcatcagctgac 1

tgacaaagttaaaatagtcgttccaataaattgac 2

tgacaaataaattgttttcagattttttttttgac 1

tgacaaatacatatatatattattttatttttgac 1

tgacaaatactagaatctatttcattactcctgac 1

tgacaaatatttgtctcatttatatttactttgac 1

tgacaaatcaaagaatgaatgattaattctatgac 1

tgacaaatcatttcttcttcctttactgatctgac 1

tgacaaatgaataaaagctgttgttatcatatgac 1

tgacaaatgtggatttgcaagagagctgcattgac 1

tgacaaatttttttcgttctgtctacacttttgac 1

tgacaacaaaggctgtaatggtgttcaagcttgac 1

tgacaacaattagaataagtatgctattctttgac 1

tgacaacactgcacgtggcatttccccacagtgac 1

tgacaacgaaatttggtatttttccaaaagttgac 1

tgacaacgccaggctggtgctgcggcgataatgac 1

tgacaactgtatcatcgtccatggcttctggtgac 1

tgacaacttcataatgtaaaattatccaaattgac 1

tgacaacttccaaggataacaaaaaatctgctgac 1

tgacaagaaatatattaatatgaatgagcattgac 1

tgacaagagcccatgtacactcatgagcgattgac 1

tgacaagattaaaatatccttatcaaatgtttgac 1

tgacaagcacgaaaacgttttaagcaacatttgac 1

tgacaagcatatattagatgacgtggcccattgac 1

tgacaaggatatggaaaaatgaaggtgttactgac 1

tgacaaggtttgtgatgacattgatggcttttgac 1

tgacaaggttttggtctctgcaggttgccattgac 1

tgacaataataagaaaaagaatcatttactttgac 1

tgacaatacgggagctatttgacttcatagttgac 1

tgacaatccacagaaaaccaaagagagacaatgac 2

tgacaatcttagtactacaacaaaaagattttgac 1

tgacaatgtatgatgatatatttttatgtgttgac 1

tgacaattaacacaaccaaaaataaacaatatgac 1

tgacaattactctttacgattgtcgtcgtcatgac 1

tgacaattatacccctggcactcgacatgtttgac 1

tgacaatttaaaagagaaaaactgaaaaaagtgac 1

tgacaatttcacaaaaatggttcgttagttttgac 1

tgacaatttcggtttagtgtggtttaatctttgac 1

tgacacaaaaacacaatcttatttcacagcttgac 1

tgacacaacgcaataacgcatgcatgcattttgac 1

tgacacacaaaagtactcatactagatagtatgac 1

tgacacagaaataatattgaatttttttttttgac 1

tgacacagagaaaggacagcagtacaaccattgac 1

tgacacatgtcagttaactcctttgaacaattgac 1

tgacacatttcctttaactctccattgatgttgac 1

tgacaccagatttgtgaatactactaaccgatgac 1

tgacacctgtcaagatctacaatgacggaattgac 1

tgacacggatccacgccgtagaagataggcatgac 1

tgacacgtggcagtttatcaatctaagattttgac 1

tgacacgtgtcatgatgttaatggttaagcttgac 1

tgacacgtgtcgtacacatgttagtaggtgatgac 1

tgacacgttcatgtgcataggtgttttaacatgac 1

tgacactaaattatgaattaatttaagtgattgac 1

tgacactatttgacacacacacaagacaatgtgac 1

tgacactgaaactcatgcggtcaccagaaagtgac 1

tgacactgcagaggtgtcagtgtttgcattctgac 1

tgacacttatatcctcaatatttgagaactatgac 1

tgacacttcccaaacacataacatcaacacttgac 1

tgacactttttttcattgattgagtaatatatgac 1

tgacagaaaaagaaaatttcttggagaacattgac 1

tgacagaaacaaatggagagcttgagtcttttgac 1

tgacagatacgtggcacataaaactaggtggtgac 1

tgacagatggttggtgagaccaattactagttgac 1

tgacagatttattcaatttttaatagattggtgac 3

tgacagcactcgcaatgtcatgtaagagttatgac 1

tgacagcccttgcaactattcaattcaaagttgac 1

tgacagctacgttgatatgtattcgtttggttgac 1

tgacagctattctcgagtcttattcttccaatgac 1

tgacaggtaatggaaaaacagttgtaagttttgac 1

tgacagtctcaagggagtccttatgatatcatgac 1

tgacagtttctgaaccgttgagatgtttggatgac 1

tgacataaataccctcattacttgcacccattgac 1

tgacataatgtttttttccaccactatattttgac 1

tgacataattggcgcggatcctcatttgtaatgac 1

tgacatacaatatggtggtggctcactaaactgac 1

tgacatacctcaagttggtcaccaagattgatgac 1

tgacatagttatggcattattatgacataagtgac 1

tgacatatacatacgtgtatacatgacacattgac 1

tgacatattaattgacaaaattttataaatttgac 1

tgacatattatttgttcaaattttataaatttgac 1

tgacatattgttttgcagttttattgtcatttgac 1

tgacatcaattcttcatattaatctagttattgac 1

tgacatcaccttttctagaataatatagttttgac 1

tgacatcattttaaaatatcaagttgtcggatgac 1

tgacatctctcgttcagacatcctcaccttttgac 1

tgacatctgactccgtgcataaactacaagttgac 1

tgacatgaatttcattctctcttgataaaggtgac 1

tgacatgaccgaaacgaacttttatataaaatgac 1

tgacatgatcaagagaacgatgtataattattgac 1

tgacatgcatcatcaacaaacatatgttatatgac 1

tgacatggaaaccaagcagcatttggtgcagtgac 1

tgacatgtagagtgtaactattaatgtctattgac 1

tgacatgtcatttaaatctcaaagtttaatttgac 1

tgacatgtctgctctaatgataaagccactttgac 1

tgacatgtggagtttgaacccaccagataaatgac 1

tgacattaacatcaccgccttggggacactttgac 1

tgacattggtacgtattcagatggtttggtctgac 1

tgacatttaaagaatcatcacatcaaacacttgac 1

tgacatttacgatttgtccattacatgatattgac 1

tgacatttccaactctacatgtgactttgactgac 1

tgacatttgagagagagcttcttgaacacattgac 1

tgacatttgtcttggcataataatgttttactgac 1

tgacattttcttcttaatttatttacaagtttgac 1

tgacattttgaaaacatatgtatattatttgtgac 1

tgaccaaacattagttacgattttctttatttgac 1

tgaccaaactcaatagcaccgtatccaaatatgac 1

tgaccaagaaaatcctcttcttaaacttctttgac 1

tgaccaagaataaaagaaatgtggcagataatgac 1

tgaccaagcccaaagtgaccaagcccaaaagtgac 1

tgaccaatcacttgatataaattaaagaatatgac 1

tgaccaatcttatgcatgttaattaaatggttgac 1

tgaccaatgtctcatatcatcttccctcctgtgac 1

tgaccaattacagaaatattgttttgaattttgac 1

tgaccaattattacgacgaaaaaaaaaaggttgac 1

tgaccacaacatgcatatctaaaagctagtttgac 1

tgaccaccacatatcggcatggcttcgagtttgac 1

tgaccaccattcacttctccacaacttttaatgac 1

tgaccagagctctgttttgtttctattttcatgac 1

tgaccagagcttgattaacggtgtaatagactgac 1

tgaccagttttgtccacggataatatgggcttgac 1

tgaccatcacttcaaattctccggcttcacttgac 1

tgaccatcattcatgagtatttcaactctgatgac 1

tgaccatgcacagaacatttactgaaccatttgac 1

tgaccatgtgttctgctattattcaggtacctgac 1

tgaccatgttgttgttagaaaactatttcgctgac 1

tgaccatttatatgtttcgtaattaataacctgac 1

tgaccatttcttttcttttcttattaaagattgac 1

tgacccaatgaagacccgacccgtctggtaatgac 1

tgacccaattggtcctctaggtagactgcagtgac 1

tgacccacacaccacaaaaatttgttcttaatgac 1

tgacccacagactttggcccatatagtaccatgac 1

tgacccaccttttctcttaatgggtccgctttgac 1

tgacccagtgttgttacccggagatcctagctgac 1

tgacccataaattaggcttgtgatgtggagctgac 1

tgacccatgcgatcccaaaatgaatggagaatgac 1

tgacccatgctgacgaaaatatgtatgtctgtgac 1

tgaccccctaattacacactgcctacgcccatgac 1

tgaccccctctctgtttttatagccgagagatgac 1

tgacccctatgactcatgttaaactaatatttgac 1

tgacccctctagagaccatgtatatatatggtgac 1

tgaccctaccaggtgctccacgtgtaagacctgac 2

tgaccctccagaggaagagagctagaattgctgac 1

tgaccctctttaatccttctattaacgggcctgac 1

tgaccgatatagtggagggtggcagtggctgtgac 1

tgaccgatttcctttggtaaatctctgacgatgac 1

tgaccggagagattttgcatgtaaatggatgtgac 1

tgaccggccatttaatacatataaaaatcattgac 1

tgaccggccatttaatacatgtaaaaattgttgac 1

tgaccggcggaagatttctccggcgaagcagtgac 1

tgaccggtcaaagtttcacacaacacaacaatgac 1

tgaccgtttcattttatatttagtcacacgatgac 1

tgacctaaaagatgattgatggatccatagttgac 1

tgacctaaagaggtgctttttgcatcgaaagtgac 1

tgacctcaaaaactctatttggcaatttatctgac 1

tgacctctacaaattattaccgtacgtatactgac 1

tgacctctggacagattcttaaacaaagctttgac 1

tgacctcttaattaaagtgaaatccggactctgac 1

tgacctgcgccgttggatctgtttacttctttgac 1

tgacctggcagtgttgactccttgttttaaatgac 1

tgacctgtctttgtcgatgtggctgttccagtgac 1

tgaccttacctttttgtttctcgtgtctctctgac 1

tgaccttacttttgtttgcgtgtgattttattgac 1

tgaccttagaagagacgatagtagtaactagtgac 1

tgacctttaatttcatcattttgattgtacttgac 1

tgacctttataaagataaaaaaaaaagaatgtgac 1

tgacctttccgtttagaaccatcgttagggttgac 2

tgacctttcgaaaggatgcaaagaacggatgtgac 1

tgacctttcggaaggatgcaaggaacggatgtgac 1

tgacctttgtgttggtccctgacttctcctatgac 1

tgacgaaaaaacacatgaggagcttagctattgac 1

tgacgaaaatatgtatgtctgtgactcataatgac 1

tgacgaaaatattcgttttagtatactagcctgac 2

tgacgaaaccagcttttaatagccccttcgttgac 1

tgacgaaacgagcttaatccacccatttcgttgac 1

tgacgaactccttatccaacactctctccggtgac 1

tgacgaagcagttttgtattcacttatattttgac 1

tgacgaatctttcgctgttggccataatttatgac 1

tgacgacgtcgacggctgtggtctgtctcgttgac 1

tgacgagactccctatgggccatggcccatgtgac 1

tgacgagcatggtataatcgttgattgaggctgac 1

tgacgagtggctttttttctttcttccctcatgac 1

tgacgataacgacgagagagggacgaggaaatgac 1

tgacgatcgtatcactataagccaatattaatgac 1

tgacgatgaccgatttcctttggtaaatctctgac 1

tgacgatgggacaatgaagtatggtgacgtatgac 1

tgacgattatatatataacacacataaattatgac 1

tgacgattcaagatcttgatgatgaagcttatgac 1

tgacgcaaaagaaaatctgcttgtctatgagtgac 1

tgacgcacgtgatacagaatgtatcagactttgac 1

tgacgcagcaactagctgggttttcccgctatgac 1

tgacgcctgttgctatgattgatgcgataattgac 1

tgacgcgatctatatacataacatacgctcatgac 1

tgacgcgtgaattgatgtggacctacttacctgac 1

tgacgcgtgtcgagagtactcaccattactctgac 1

tgacgctgagcgattcctttttctatttcagtgac 1

tgacgcttaaaaatgattcttcaaattgacctgac 1

tgacgcttataatttatgatttgtggaacaatgac 1

tgacggaaggttcctagatatgttagaatcatgac 1

tgacggatacgtgtagatccgccgatccctatgac 1

tgacggatgaagacctactgtctcggctgcttgac 1

tgacggatgagaggtataagttgagggttaatgac 1

tgacggcaaaattgacaaaaataaaatattctgac 1

tgacggcgattagggttttcgtgaaagatgatgac 1

tgacgggcctcaactctccatagtgaaagcatgac 1

tgacggtccacaagcctgacagccctcgagctgac 1

tgacggtttcaagggcaatttaggaatctcatgac 1

tgacggttttcgcctcaaattaaacatacattgac 1

tgacgtatatatagccaacattcatatgtaatgac 1

tgacgtatggtattttgcttgatggcttgtgtgac 1

tgacgtcacctgtgcctaccaaaaaaaacactgac 1

tgacgtcagaaaaatggatataagtcgcatttgac 2

tgacgtcagcagcgtcgttgccgccggctgttgac 1

tgacgtccacacgcatcggatcggagaggcctgac 1

tgacgtcgtacgccgaattacagaaaccaattgac 1

tgacgtcgtttattggttcttttactactcttgac 1

tgacgtcgtttctagttgggatgagactgactgac 1

tgacgtcgttttgaagtcatccgagagaaaatgac 1

tgacgtctttcaccgccgtaaatctttggattgac 1

tgacgtgattgatatatttatttgataaatttgac 1

tgacgtggagaaaatagtcccacctaagtgatgac 1

tgacgtggattatggtaatgacatggaataatgac 1

tgacgtggattatggtaatgacatggaatgatgac 2

tgacgtggccgatgagagctgaccagtacactgac 1

tgacgtggtaatatatgctaggattgaaggatgac 1

tgacgtggttttacgtcagcggaggcgctgatgac 1

tgacgtgtaccaacaaagttttttgcatggatgac 1

tgacgttacggtgacactgaaggacaaccactgac 1

tgacgttatgcggtactaagcctctgtaccttgac 1

tgacgttcatacaatttattcttgttttaaatgac 1

tgacgttcttagcatatgatttcgtatatggtgac 1

tgacgtttaaaaaagaaacaaaaactttagttgac 1

tgacgttttctgcacgagctgtcacgcaaggtgac 1

tgactaaaaatcattctgttggatagttctttgac 1

tgactaaaatgcgttgagtgacgtcatgacgtgac 1

tgactaaatagtctaaatatcaatcaaaatatgac 1

tgactaaatattttatttactaattcctgtttgac 1

tgactaacgtgtatactttcacacgtgtgcatgac 1

tgactaagggtgttttttttgggttaaaatttgac 1

tgactaatctaattcccaaaagttttatatttgac 1

tgactaatgtcaaataagtgacgccttttgatgac 1

tgactaatttacaaaaaaaaaaaaaaaatgttgac 1

tgactacaactaatttttttttttttttttttgac 1

tgactacaatagccaccttccctatacattatgac 1

tgactactgaccgaaattcttctgtgctaattgac 1

tgactactgcgaataatggatactgtgaacatgac 1

tgactactttacgttgcatttttgaaaccattgac 1

tgactagacgaccactgcacctgcaattctgtgac 1

tgactagaggaataaaaggcaatgcgttaggtgac 1

tgactagagtcgatatccaaatccaacgaactgac 1

tgactagagtctccatccaagtggggggagatgac 5

tgactagatgcatcctagctagtttttctactgac 1

tgactagattattgggaaattagttcctgtatgac 1

tgactagcaaaaaacaaatatcgttcacatttgac 1

tgactagtttctctaaacttaataaattcagtgac 1

tgactatacatacacatacaatcaaatgttgtgac 1

tgactatactatatatgcttatacgaaaagatgac 1

tgactatacttgttgggcagttgacctaagttgac 1

tgactatggtaaatcgtttcagcaatgcacatgac 1

tgactatggtgagcccaataataaaagtccatgac 1

tgactattgggtaacactaaaatactgtatatgac 1

tgactatttgattattcttcccttttattattgac 1

tgactattttgtaccaatttaaaatagtgtatgac 1

tgactcaaccatcactatctctctctctctctgac 1

tgactcaatacattcaaagaaggacaaatgttgac 1

tgactcaatagaaaaaaaagacaaagatgcatgac 1

tgactcagcttgcagcacaatgccttagccgtgac 1

tgactcattcattgcattcagtttcaagttgtgac 1

tgactcatttatttacagaaaccactgagtttgac 1

tgactccaatcatcctataaactaagagggttgac 1

tgactccattcaattcactcttttatagcaatgac 1

tgactcctaaactccttttaatttatttgattgac 1

tgactcctctgttgaggttgatgtctctctctgac 1

tgactccttataagtagagagtgaaacaccttgac 1

tgactcgaacccactaaaccaagacgagccctgac 1

tgactcgtattatgctatatagagtgaccactgac 1

tgactcgtttgagtcctaattaattttcggatgac 1

tgactctaaataaattgctatacactaaaattgac 1

tgactctaagatctcaccaaaaataacaacatgac 1

tgactctaataataaatttgattaaattaaatgac 1

tgactctcaactaccaaacgacaatagtctctgac 1

tgactctcgcttgagaagtgcattctcatattgac 1

tgactctgaaagactttcaatgtttgatctttgac 1

tgactcttttagtcataaaacattcaaattgtgac 1

tgactcttttgagctctaaataaagatagattgac 1

tgactgaaaagacccttaaaagcaatgcacctgac 1

tgactgagtgagatttaactttcaaatattttgac 1

tgactgataacgtaacgtaatttcacacgagtgac 1

tgactgatgataggtataagttgacggttaatgac 1

tgactgatgcaaagcttgccaagagatatattgac 1

tgactgattgaggaatttggatccgaaacgatgac 1

tgactgattttccttttttttaatctctttctgac 1

tgactgcaatatagaccgggtttcatgtaattgac 1

tgactgcgtcaaccatataagaaccaaaaaatgac 1

tgactgctatacctgcacaagatgaaatctttgac 1

tgactggctatgtcctgagtaagtctaaatctgac 1

tgactggttattcataaacaactacaaaatttgac 1

tgacttaacaccgttaattttttcaattagttgac 1

tgacttacgaaattatcaacttgaaatgaattgac 1

tgacttagatataataatattcaaattaaaatgac 1

tgacttagcctaaccgatctgagccggtttatgac 1

tgacttagtcatgtagccggtctcccatgtttgac 1

tgacttagttggtgtatctttatttcaattttgac 1

tgacttatcaagatttgagtgaaggcagaattgac 1

tgacttatcaccatttttttttttgttccattgac 1

tgacttcaatatccgcttattataatgagtttgac 1

tgacttccccaccaacaaattgagtttaaaatgac 1

tgacttcctaaataataaataaagtttttgttgac 1

tgacttcgagaatagagtattgtcaaatgcatgac 1

tgacttcgatttgtttggtgagacgtgtgggtgac 1

tgacttctagactcttttcttgcaagtagcttgac 1

tgacttctgcactaataggccgtctctgtggtgac 1

tgacttctgttttaaactttcaagtttgcgttgac 1

tgacttctttatgagtttctctgcacatgagtgac 1

tgacttctttggctcctaaataatcatgatttgac 1

tgacttgactctcacacaatttgcattaagatgac 1

tgacttgagaagacgaagaaggtacaaagcgtgac 1

tgacttgatggtctaaccattaacattagaatgac 1

tgacttgattttgttctttgttttattttactgac 1

tgacttgcatgttatgtactaaccaagaagttgac 1

tgacttgcgcaagtctttccaatgctctctttgac 1

tgacttgcttcagttccaagttcgttgtacatgac 1

tgacttgcttcgtcactttcgtaaaaccccttgac 1

tgacttgtcggagaagaagcaaactctctgctgac 1

tgacttgtgaggaaaacgaaaacacgggcgttgac 1

tgactttaaatttaaatcgcaaagtttcggttgac 1

tgactttacccaatatgtcacgtagttaagttgac 1

tgactttagaaagatgatattgtttcttttctgac 1

tgactttagacagctagagtttcaactcaattgac 1

tgactttataattaaaatggttatattttgttgac 1

tgactttatttcttatatcaatatacttgtttgac 1

tgactttcatgtaaaaaaaaaaaaactcaattgac 1

tgactttcgaaataaataacaaagtttttgttgac 1

tgactttcgaaataaatgacaaagcttttgttgac 1

tgactttcgaaataaatgacaaagtttttgttgac 1

tgactttcgaaataaatgagaaagtgtttgttgac 1

tgactttctaaataaatgacaaagtttttgttgac 2

tgactttctatataaatgacgaagtttctgttgac 1

tgactttctcgttggcattagttgtgacttgtgac 1

tgactttgaaatttaatgcctttcgttttcatgac 1

tgactttgaatccgttcttcaatatattcattgac 1

tgactttgactgacctaatttaacttcttactgac 1

tgactttgagagttttaattcttaagaatcttgac 1

tgactttgcaactttattaacatatatggtttgac 1

tgactttgtaccgttaaattgatctgttaattgac 1

tgactttgtttgtttggagaacaagctaagctgac 1

tgacttttaccaaaaaaatttttttgaactttgac 1

tgacttttaggaagaggcgctttggctgtggtgac 1

tgactttttcttttttggtcaaatgtataaatgac 1

tgactttttttataaaatgatcaagttttgttgac 1

tgacttttttttatttgtggtgcaccatttatgac 1

tgacttttttttccccgatcaattaaaactttgac 1

28 : 360

tgacaaaaacttaatgagaacgatcttgcacgtgac 1

tgacaaaaattaattaaatagtattgtaggcctgac 1

tgacaaaacacaaaccaacatggttgaaaggttgac 1

tgacaaaacacacatgttggattgagattcgatgac 1

tgacaaaagaaaatatgttattatcttttgcatgac 1

tgacaaaagctccttctatcttgtattgaaggtgac 1

tgacaaaataaataaatacctacaagattcgttgac 1

tgacaaaataattgtttagctacctaacaaattgac 1

tgacaaaatcatatcctagtcatttccatctttgac 1

tgacaaacgcgagttcatttttgactaggcaatgac 1

tgacaaacttataaagtttgatttttcgtatgtgac 1

tgacaaactttttcttcgttttctgctattattgac 1

tgacaaagaaaaaaaaaaagagtcacttttgatgac 1

tgacaaagaaatatagagagaaagaaacttgttgac 1

tgacaaagacaacataatccaaaccaggtatctgac 1

tgacaaagataattgcaaagtttcacttgaaatgac 1

tgacaaagctaacggataaacccttattattttgac 1

tgacaaatcctcaagattttagtaaaaactaatgac 1

tgacaaatctcgttattacatgtggtggtccatgac 1

tgacaaatctcgttattatatgtggtggtccatgac 1

tgacaaatggaggtctaaagggagaccatacttgac 2

tgacaaattcaacaaagaccattctttttggatgac 1

tgacaacaacaacaacataaaacagcagtgagtgac 1

tgacaacaagggtttcaagaatttgaaatctctgac 1

tgacaacctctttttttggttcaaccaaaaattgac 1

tgacaacggttttagcaaaaggattgtatccatgac 1

tgacaacttagtgtccagaccacacagtgatctgac 1

tgacaagatactgataaaaaccatttatacaatgac 1

tgacaagattattgagtgtggtgtacagatcatgac 1

tgacaagatttatgaaaagttaaagtctaaggtgac 1

tgacaagcgaaaacgacgttgtattgaattcgtgac 1

tgacaagcttattgtcaccttttatgttaaagtgac 1

tgacaagcttctcttggagtttagttgactattgac 1

tgacaagtaaagagtcgtgacaaatcagtatttgac 1

tgacaagtatccttgcctttttttccagatggtgac 1

tgacaagtcaaaagagagatagttttgagttttgac 1

tgacaagtgcaagggaccggttgatgtccacttgac 1

tgacaagttgacaactcgtgttatatccaaaatgac 1

tgacaagtttatagtccaatcacttcgtacagtgac 1

tgacaataagtcatcatatctaatttagtgcatgac 1

tgacaatacatgtggttgcaggaagtggcaaatgac 1

tgacaataccttctggtttttgcttctctaagtgac 1

tgacaatactaattaattgcaaaaattttgtttgac 1

tgacaatagttggtgccgacaaaaaataataatgac 1

tgacaatatattatagtataagtttttggtattgac 1

tgacaatattacagcatcaaggaacatgttcgtgac 1

tgacaatcacaattaatattgaaaattaagattgac 1

tgacaatctgcaagtcatgaagatttttatgttgac 2

tgacaatcttagtactagaaaacaaaagactttgac 1

tgacaatgaagaaaatggaaataagttaataatgac 1

tgacaatgatctaaatatacaagttgtcaaaatgac 1

tgacaattagatttgaatgattttgatctctttgac 1

tgacaattccgacgacctaattttcctctcgttgac 1

tgacaatttgaccaaaaggaaaaaaatcttggtgac 1

tgacaattttactttttgaatgatcacactcgtgac 1

tgacacaaaaacaaaatctatagaaaactgcatgac 1

tgacacaaaacattttctttcttctaagaatatgac 1

tgacacaaacatgcaaacctagaaaatagctttgac 1

tgacacaaattgcgtggatggtgccgattttgtgac 1

tgacacaagtctagctagattagtgttcttgctgac 1

tgacacaatactttattgatgttcatgagtcatgac 1

tgacacaatctaactcaaaccaaccttgtctttgac 1

tgacacaatctataaatatgtatatcacacactgac 1

tgacacaattgtatatcttcttgaatagaatatgac 1

tgacacacaggttagtgataactacaagatcatgac 1

tgacacacccaagtgcaaagcacttattcgtgtgac 1

tgacacatcaacctttttatgtgatgagaagctgac 1

tgacaccataccaaaagaaagtttttttatattgac 1

tgacacctaattaatccttacatattccattatgac 1

tgacacgtcagtaactttatgccaatagggtctgac 1

tgacacgtgcacagattataagtaaccacacgtgac 1

tgacacgtggtttggtgactcgcccatactcatgac 1

tgacacgtgtacataaagaaaacaaagtgggatgac 1

tgacacgtgtcagtataacactcgtgcataattgac 1

tgacacgtgtcttataatcaacaaggcttttctgac 1

tgacacgtgttctccaatattcttatactcattgac 1

tgacactctgattatagtgaccagtgaggcagtgac 1

tgacacttcgatttggtaggctaatctgactctgac 1

tgacagaaaggtccaaaacaaactttgttcaatgac 1

tgacagaaagttttgataaaacatgttgaatatgac 1

tgacagaagcttattggagtgtttgtctagtatgac 1

tgacagcagaggattttgaagagcacagaatctgac 1

tgacagcctcaagatttgtcgttgtaagagattgac 1

tgacaggtattgagaattttctggtctttctgtgac 1

tgacaggttaccccaataatagaccaaatatgtgac 1

tgacagtaatttttgtcatgaaattaataaaatgac 1

tgacataaattcattaaaaggttacgtttgagtgac 1

tgacataacagccatgtactttaaatactctgtgac 1

tgacataactttatataagcctttttttcgtttgac 1

tgacataagaaatcttatgttctcaatggtaatgac 1

tgacataatagtgtatatatacgtgtaattagtgac 1

tgacataatatttagtctgtgatcgactatattgac 1

tgacataatgctattgttgttaactttttgtttgac 1

tgacataatttatttatattaaatatattgtgtgac 1

tgacatatacagtcctttggaatctgaggaattgac 1

tgacatattttgatcgccagttgttgtgcacttgac 1

tgacatcaactcctaatataataataaaaagatgac 1

tgacatcagccttatttgtctatatgtatgggtgac 1

tgacatccttttttttttttgggcaagtattatgac 1

tgacatcgcactgtcaagactttaaaagcatttgac 1

tgacatcgcttttgtcgacaaatcatcattggtgac 1

tgacatctaaaattctaaaagcaacaatagcgtgac 1

tgacatgaaacgactttttgtaccgtcgcgtatgac 1

tgacatgaaagaggccattggaggactgaacctgac 1

tgacatgaatactgagttgcacaccacaagtttgac 1

tgacatgaatgtgacgtcaatgatcgtggggatgac 1

tgacatggaagaggccattcgaggattgaacctgac 1

tgacatggttaaatttgaagttattattgttttgac 1

tgacatgtaacaaaattgttttgtaaagcaattgac 1

tgacatgtaccgccgcccataccacatatggttgac 1

tgacatgtatatgtaaattgtttgtcatccattgac 1

tgacatgtgccaagaatacaaatgtacatgagtgac 1

tgacatgtgtcaaatctattgatcttagattttgac 1

tgacatgtgttgcaccgaaaaagagtgactagtgac 1

tgacatgtttcaagaatagggtttttaattgttgac 1

tgacattatataatcatttggaccacgtgaaatgac 1

tgacattcgattaattaattatagatttcggatgac 1

tgacattcttacgttgaagtttactatgaagttgac 1

tgacattggactgttaagactttaaaagcatttgac 1

tgacattgtagaggctctacaagccggtgctttgac 1

tgacattgtttcagttttgtaggtgaaaagcctgac 1

tgacatttctaatcacaatcatcaccctcgtgtgac 1

tgacatttgcatgtggtgaagagtgattgaaatgac 1

tgacattttcttcatgatgttcaacctcggtttgac 1

tgacatttttttaatgtgacagtaatatcttttgac 1

tgaccaaaaaaaaaaagatcatttactatatttgac 1

tgaccaaaaacaaacaaacatactatacatgttgac 1

tgaccaaaagtaacaattgaaaaacaatgacttgac 1

tgaccaaaattttggttcaaatgtagttttgttgac 1

tgaccaaacctcttttataatcaaactatccatgac 1

tgaccaaataactagcttagtttggaaactcctgac 1

tgaccaaatctctctggaggaaggtgacagtctgac 1

tgaccaaatctttcagttattggttctaattttgac 1

tgaccaaattggtcgagaattgttatcgtcattgac 1

tgaccaagaggtaaaagaaaacaacttttgtttgac 1

tgaccaagccaaagagtgaccaagttcaagagtgac 1

tgaccaagcccaaaagtgaccaagtccaagagtgac 1

tgaccaaggaaccatgacttgtcctgtcaccatgac 1

tgaccaataatatatattattattatttgttttgac 1

tgaccaccactcttttaccaaaaaaacaaaattgac 1

tgaccaccatgcactttcgttcgaacagtttttgac 1

tgaccaccgtttgttagctaggttataaactctgac 1

tgaccacggaaaaagggcatgggacgaagaagtgac 1

tgaccagaagatttggttgcttatgacgaaaatgac 1

tgaccagatgtcttcattattagataaactgatgac 1

tgaccagattccacgaagagacttgcattcgttgac 1

tgaccaggaataattcaatggaagtatgagtttgac 1

tgaccataaaagccataaccacgggtcccacgtgac 1

tgaccataacataattaagaaaattcaaattttgac 1

tgaccataatagttataaccaaaaacaaatcctgac 1

tgaccatatatcttttcttttatctcaacaagtgac 1

tgaccatatctgtatactagaaacagttcgtctgac 1

tgaccatgactcgtgattatggagctgtcttatgac 1

tgaccatgtctaaaacaaagaagtgtttggtatgac 1

tgaccatgtgagactataacgtctaacgatattgac 1

tgaccatgtttgattagagtaaaactaaatattgac 1

tgaccattagagacgtaatatttacgaagatgtgac 1

tgaccattgattttctttatttttttagaagatgac 1

tgacccatagctttgcttgacttgtacatatgtgac 1

tgacccatatgatatgaaaacactctttccattgac 1

tgacccctttatcgccgacgcttcaaactcggtgac 1

tgacccggatattcggatccgtattgtcgtattgac 1

tgaccctcgaaagtttcaagtactttaggatctgac 1

tgaccctgtacaaaactatgaaataatttaattgac 1

tgacccttcttctctttgtgggtccgcccaattgac 1

tgaccgaagcttgttggcctctgagagacccttgac 1

tgaccgacaaaattaaatcacacggccagttttgac 1

tgaccgagtcagcacacataaaatctggtacttgac 1

tgaccgatcgaagggaaagatggatcaaaaggtgac 1

tgaccggcacaacaataattctggtggagatttgac 1

tgaccgtgcggcgaattgaccgagtagctacttgac 1

tgaccgttaagtgtttcttgtcaaatattatgtgac 1

tgacctacactaagaaaactcaccaatgactatgac 1

tgacctactctataaaatttaacagctacatatgac 1

tgacctagggagcatagaaccaggaactaccgtgac 1

tgacctcaagagtttggcttgtgaaaagttgatgac 1

tgacctcatgttatgtgatttatgcttgtctttgac 1

tgacctccaacgtcggcatatatttgagtcggtgac 1

tgacctcgatgaataggtggtgaatacgtctgtgac 1

tgaccttaagaaataatttctcagccaactgttgac 1

tgaccttagattttgtgatttaaaatcgatcatgac 1

tgaccttcccttcactatacggtttcctctggtgac 1

tgaccttcttacttgcacctcaggcctcagcttgac 1

tgaccttgcacgacggtctacactgagcttgttgac 1

tgaccttgtagttatgtaattagtcacgctaatgac 1

tgacctttgtattgtacaaaactaaacttacgtgac 1

tgacgaaactcattcaggcgaacgattacgtctgac 1

tgacgaaatggcaagaaacacgtcgaaattattgac 1

tgacgaactaaggtttcgtcaaaaaaattgtgtgac 1

tgacgaagcagagcctgcaaaggaagatgaagtgac 1

tgacgaaggagttattttcaggcgtaagcatttgac 1

tgacgaatgtgtcctctcaaagccagcttatttgac 1

tgacgacaaataaaaagaagaaagtattaagctgac 1

tgacgacgacgtccaccatttgtatttcaaaatgac 1

tgacgacgataatgacgtggaacgttttgtgctgac 1

tgacgacgtctttacgatactctctctaaatgtgac 1

tgacgagaggaaaatcaagcttcgtgatgttttgac 1

tgacgaggtagatttgaaaaagttcttaactttgac 1

tgacgataataaccctatcccgacacaatctctgac 1

tgacgatagagtgaatgatgcaggagcagcaatgac 1

tgacgatagcatgcaatacaaataaagaacactgac 1

tgacgatattttcacgtttgacatattcatcgtgac 1

tgacgatccatcatgttgttgttccggcccattgac 1

tgacgatctatggcatgtcgatataatctatgtgac 1

tgacgattgcagtggtgatggtgatgatggtatgac 2

tgacgatttcgtaaactcagcttaacatttattgac 1

tgacgatttctaaatcaaattgtataaaaccctgac 1

tgacgatttcttctttttttgagcaataattgtgac 1

tgacgatttgtgtgtatagcaatcgtctgttatgac 1

tgacgcaccatgtcaacaacaactcactcacgtgac 1

tgacgccgactcggcgggaccagaagagacgttgac 1

tgacgctattaatacactcatacaattaattatgac 1

tgacgctgcggcggcggcggcggtggtggaagtgac 1

tgacgctttaacatccttgtcccctagagaaatgac 1

tgacggacaaacaaaatacggaaacacatttttgac 1

tgacggagcaggatttgtgaggaaaggaggagtgac 1

tgacggagtattgtttttaatcaatctgtgattgac 1

tgacggatagatgttagattagttataatccttgac 1

tgacggatcagaaacgaagaggacaatggtgatgac 1

tgacggcgctcatgtttactctttttgttaaatgac 1

tgacggtaacggtaacggcgtttgagtgctcttgac 1

tgacggtcgacatgtgggttgactttgatgtatgac 1

tgacggttcatgaaattgttaagcatgtaatgtgac 1

tgacgtagagagatccgttgcttaaaaaagtatgac 1

tgacgtattaccaacatctattaccggcaaagtgac 1

tgacgtcacacgaaaaaaacagacaccagacctgac 1

tgacgtcacttttgattgcactaaaaaaaaggtgac 1

tgacgtcgaaaaatagctttataacagtgttttgac 1

tgacgtcttcgcagaagaaaatatcctatgtatgac 1

tgacgtcttcggaaatgtaatgaggctacatttgac 1

tgacgtcttctcctgaagttgaaaattgtgtttgac 1

tgacgtgaatttgattttaatgaattttttaatgac 1

tgacgtgagttttcatataatttgtaactttttgac 1

tgacgtggattatgtgtaatgacgtggattgatgac 1

tgacgtggcacttatgacctcttctacgcttttgac 1

tgacgtggcgtgtaacgattggaataattagttgac 1

tgacgtggggacaattatcttattactgacattgac 1

tgacgtggtagacgattgtaattcgttatctttgac 1

tgacgttagctgaatctctccatggattaatatgac 1

tgacgttcttttaatttagaaaacaaaaactctgac 1

tgacgttgcatgtcattctttaatgttgttgttgac 1

tgacgttgtgttctttcctttttccccatatttgac 1

tgacgtttcagggattaataatttagcgttaatgac 1

tgacgtttcctttccttataataaagcaaagttgac 1

tgactaaaaagacgatactgatcggacaatcttgac 1

tgactaaaatacccctccgtcttcaacctctctgac 1

tgactaaaatgctcgttgttgttctttaactgtgac 1

tgactaaatccatcaaataaagtaataaatagtgac 1

tgactaacagaactatagaagacgcatggggttgac 1

tgactaagcgtatagtctcgtataattgacattgac 1

tgactaatacataacttacacacttttttaattgac 1

tgactaatataacatttttttaatcttctttttgac 1

tgactaatataattgcaaataagacacactattgac 1

tgactaatcaacagcgtaaattttaaataatttgac 1

tgactaatgagtgatagtctgataggtgttaatgac 1

tgactaatttcgtataatcatgatgatgattatgac 1

tgactacatgaattgaaaatcatcattatttgtgac 1

tgactactaaaataggaggatttatctttacttgac 1

tgactacttagtgtttggcttttgtgaattagtgac 1

tgactacttgattcataaatcgaacaagcacatgac 1

tgactagaaaaaagttacaacggatgtatatgtgac 1

tgactagaaagaaaactctttaaaaaatatgttgac 1

tgactagaaccttatatttattttagataacttgac 1

tgactagccaataaacgaagccaattagtgagtgac 1

tgactagtattttaatttattttgacagcaattgac 1

tgactagtcggaaaactacacgttcagtatgctgac 1

tgactataccgaagggactgcgatgaatacattgac 1

tgactatatacatccatgcatgccacatcacgtgac 1

tgactatatggtagggatgagaattacattgttgac 1

tgactatcagaaagataataaaaagtggaaggtgac 1

tgactatctaaccaaatgtataattcgtaaattgac 1

tgactatgagcatgaaactgcaagtcaaatgctgac 1

tgactatgctgtgcatctcaagacctcggttttgac 1

tgactatgtcgtgttttagtactggtcagagatgac 1

tgactattagtatagtttgttcatataccatatgac 1

tgactatttaatcagctttaagtatcatcatttgac 1

tgactcaaacgatgatccactttgaaaagaaatgac 1

tgactcacaagtggaaaggttcaagtcgatattgac 1

tgactcacaccatgtcacatgtgaagtgtagttgac 1

tgactcacatactctcaatagattccttaatttgac 1

tgactcagctttctcaagatccgaaaacacactgac 1

tgactcagtaggatttcgacattttgtaatgatgac 1

tgactccaaaaacaaaatcatcagatacatgatgac 1

tgactccggcgttgactagggttgaccggcgttgac 1

tgactcgaatggtgatgagatacctctagccttgac 1

tgactcgcacacatatcaatggttcaaaacgttgac 1

tgactcggttcaagaactaaaaagaacaaaagtgac 1

tgactcggttttgttctacttgtaatgggttatgac 1

tgactcgtctgatctcaaaggctttcaactcttgac 1

tgactcgtgattatggagctgtcttatgaccctgac 1

tgactctaactccgttaaattagtccgttagttgac 1

tgactctaagcaaaagagagggtttggtcctatgac 1

tgactctcacgttataaaagccttgtgtcacgtgac 1

tgactctgaaacgttctttgatttttgacccatgac 1

tgactctgaagtttctcgttcttcttgatcattgac 1

tgactctggaaaaccattttgtggtcctctcttgac 1

tgactctggccgtggctggtgcaattgcaatgtgac 1

tgactcttgagtgttttcgggaatatatagattgac 1

tgactcttgttaatacattccttgttcgatgctgac 1

tgactcttttaacaaaaaaaactttattacattgac 1

tgactcttttttcttttggtttaacctttagttgac 1

tgactgaaatatcgccgtttaccaatgtaacatgac 1

tgactgaacatattgcgtatattgcatgttgttgac 1

tgactgatgtcacaagaaaaacaaatcaaagttgac 1

tgactgcggaaaatgtaaacttgtaaaggaaatgac 1

tgactggtaaccgaactcttcattcttataaatgac 1

tgactgtcatccacatgatctctaattcaacttgac 1

tgactgttcgacgacaaagagcacaaatccgttgac 1

tgactgtttgactccaagtgagtatttaaatatgac 1

tgacttaaaaaggtataatattatacaatcgttgac 1

tgacttaaacatctacttggatcaaacaactttgac 1

tgacttaacacacgctcctaaatccaagtacttgac 1

tgacttaacgagtaagttattagatgttggtctgac 1

tgacttaagttataatactgacataacatatttgac 1

tgacttaatgaaaccgtctcaatgtcacattttgac 1

tgacttactaataaattattgggcttcataattgac 1

tgacttactattactgtttttggggtcagtgttgac 1

tgacttatatataatttgtagtcaataacaaatgac 1

tgacttattaagattaaaaatgttaaaagtagtgac 1

tgacttattaagttcaattatgacttaaaagctgac 1

tgacttatttcgagtcgagtcctacctcattgtgac 1

tgacttattttcaacctttttggaaaatgtgttgac 1

tgacttcacgtgatttgcggtgtgtgatcagttgac 1

tgacttcgtcgtttttttttttttcgtcaacctgac 1

tgacttctttaaattccaacaaatttaaatactgac 1

tgacttctttttcttataatatcagctgcaattgac 1

tgacttgacaagtatgatttattgtatatagttgac 1

tgacttgacgcaattagtgtaacaaaatgtaatgac 1

tgacttgagaagtgtagaattagatattgagctgac 1

tgacttgcaactgagttgcttacgggcaaacctgac 1

tgacttggcggctagtgtaatagttttaaaaatgac 1

tgacttgtcgattgacgagctgacttgccgattgac 1

tgacttgtgcattaatgcacgtatccactacttgac 1

tgacttgtgcgagaattctgtgtatatatggttgac 1

tgacttgttcgtctctcaaaaagacaactacctgac 1

tgactttaacacatatagtttcgaattttgaatgac 1

tgactttaagttaattaatgtcggttaataagtgac 1

tgactttagtgcctagaagaaagggatgataatgac 1

tgactttagtggaatagatacgcgatagactttgac 1

tgactttagtttaggaggaccttgacctatgttgac 1

tgactttatggtagggatgaaaattacagtgttgac 1

tgactttattttaattgtttcaggacaaagcttgac 1

tgactttattttcttgttcaaataaatatgcttgac 1

tgactttatttttataaacaaacaaaataagttgac 1

tgactttcacttcttgtctctcagatctgttttgac 1

tgactttctttattaagcaagttgcctaatcatgac 1

tgactttgaattttgcaccaaaccggttcttctgac 1

tgactttgattcctccttgggtattctctacatgac 1

tgactttgttatgagccactaatttattattttgac 1

tgacttttaaatgtgtacattaacagagactttgac 1

tgacttttccgtactcactcaccacgcgagcgtgac 1

tgacttttcgtgattgttgaatcgtctatatatgac 1

tgacttttggtcaagtcgagtagctgagattgtgac 1

tgactttttaaattttaaacttctgagggttatgac 1

tgacttttttgccaatgaaagcataaaatacttgac 1

tgactttttttttttcttttttttaatatatatgac 1

tgactttttttttttgtgtgtttgaaatcagttgac 1

29 : 387

tgacaaaaagagatgagagagttggtgtggtgatgac 1

tgacaaaacctcgatcaaataaagaaattaccatgac 1

tgacaaaactcagaaacagtggttaggattgtatgac 1

tgacaaaactttaatctaatttttaaaatcatatgac 1

tgacaaaataaaaacaacctacagtgcacaaaatgac 1

tgacaaaatttcatataacaaataaaataattatgac 1

tgacaaaccctttgttttctttttgtaacaaagtgac 1

tgacaaactaaatattagttcttaaacagttgttgac 1

tgacaaactcgtggctgaatgcaaagatagccatgac 1

tgacaaagaaaaaataaattaaaaatacaaaggtgac 1

tgacaaataacaaaataactaggcatttgtagttgac 1

tgacaaataataaatagtctgtttttatgtttttgac 1

tgacaacaaaaaaaggtaaataatataaaacagtgac 1

tgacaacacaactgctataactcaaaagtcaaatgac 1

tgacaacacgacgagacgaaacgatgtcgttcatgac 1

tgacaacatacaacaactaatttctcactgcagtgac 1

tgacaacattcctactgaaattaaggacaatgatgac 1

tgacaaccaccgccgcgaccacctttaccgcgttgac 1

tgacaacgcatgtctatgtgacttctccttgtgtgac 1

tgacaacttttgaagaacaaatctcgcctaatatgac 1

tgacaagatatgtataacaatatcaatgtctaatgac 2

tgacaaggaatacaaaatgaagaaagaaaaaaatgac 1

tgacaagtcatcaccttggctttgcggagttcgtgac 1

tgacaagtgagaagtgagaactgttgcatgcagtgac 1

tgacaagttattaatgaaagacacatcttgcaatgac 1

tgacaagttgggcatcaataatacgttacgtgttgac 1

tgacaataaacgataacgagattaattctagagtgac 1

tgacaataacgtctccaacgtgtcaaaactatatgac 1

tgacaataagacattaagaccccttccatatcatgac 1

tgacaataatcccatgtagtatatatgcgataatgac 1

tgacaatctcacgtgagacaatgtatcattcggtgac 1

tgacaatgaaccaaaccaaagagccaccacacttgac 1

tgacaatgagcgaaagtaaaatagtaaaagaaatgac 1

tgacaatgatgaagatgataatggcaatgccgttgac 1

tgacaatgatgttaaaacgatatggattttgtttgac 1

tgacaatggaagctccggtgacaatggaaaccctgac 1

tgacaatggtagtgatggcactaacggtgaagttgac 1

tgacaatgttaaaatgtgattctttcattaatttgac 1

tgacaatgttatacttcaaaccacacgaatacctgac 1

tgacaattttctatgatggtctcaggtacgtattgac 1

tgacaatttttaaaaatacttttttgaacaaaatgac 1

tgacacaaagtaatatatattcttaatacgtactgac 1

tgacacaatattattaacgggccatgggcttaatgac 1

tgacacaatattattaacgggtcatgggcttaatgac 1

tgacacagaatcaactgatgatgctttggatggtgac 1

tgacacagttgtgaccagatttattcaatcccttgac 1

tgacacatataatcacaaacaaaatcatttttttgac 1

tgacacatgacacatctactgttaagtcgacattgac 1

tgacacatggtccgtggtgatgaggcgtggcgatgac 1

tgacaccaatcatttacatattacaccaagaattgac 1

tgacacctaaacagccttaatctggacccaatctgac 1

tgacacgattagatatgtgtctaaaccaaataatgac 1

tgacacggaagaaggcaaggatgagggagatgctgac 1

tgacacgtggatttgtcacgtggacactttttatgac 1

tgacactaaagagaagcttgagtatgttcattatgac 1

tgacactagtctctgtatctttccaaattccggtgac 1

tgacactgccgaagaattatttgtccatgagaatgac 1

tgacactggcaagcctcgcataaagctatacagtgac 1

tgacacttgagttttgattccaaatctatcccatgac 1

tgacactttgagtggacatggatgggtcctagttgac 1

tgacagaaattctagggagcataagagagaaattgac 1

tgacagaggcaacttgaaaagactcaccagacatgac 1

tgacagatgcagaagaattggtcaaaatcattttgac 1

tgacagcatttttggagttattcaatagggaattgac 1

tgacagctctaaatgtgattgggtaagaatacgtgac 1

tgacaggcagtacaataatgataagtagtgtcatgac 1

tgacagtaaattacattttgtcatttacgattctgac 1

tgacagtcagagttcatcgccgtggacttcatttgac 1

tgacagtccaagatcttttgagcttttatgtgttgac 1

tgacagtggctgtaagaaaaacaaacaaatcattgac 1

tgacagtgttgcgttattgactgggtaaaaagttgac 1

tgacagttataggttgtgaaaattttgtggtattgac 1

tgacataaacacggccagtgagaacttagttgttgac 1

tgacataaccctaaagaaaattacagtatataatgac 1

tgacatagtagaaacaggaagatctcatggccttgac 1

tgacatatagtatttatgtttactgaatatatttgac 1

tgacatatgacttgttcaatatttacataatcctgac 1

tgacatatgttacttgtaaacatatgaaacacttgac 1

tgacatatgtttgggtaagatttaaaaaacctatgac 1

tgacatcaaagaacccaacctccaccatgtgggtgac 1

tgacatcaccctgcgctacgatgcaaccgctgatgac 1

tgacatcatcaattggcttgaccttcgaaccgctgac 1

tgacatcgaaccttgtaagttagctcttcttcttgac 1

tgacatctatagacatatttgatatatatttcttgac 1

tgacatgaacttctcctttaatcgcttcgatcttgac 1

tgacatgagcccattctgaaagctaataatgcttgac 1

tgacatgattatactataacattaatttacatttgac 1

tgacatgccgtctgatttaatgctatgtgtaactgac 1

tgacatgcttatggtattgatgttgagtctttttgac 1

tgacatggaaatactcaaatggatattgttttctgac 1

tgacatggcttaattttgtctcttttatttatgtgac 1

tgacatgggatcaatccaagaggcccatccaattgac 1

tgacatgttcaataaaattcccatttcatagtttgac 1

tgacatgttttgatcaaatagctaaacgccacgtgac 1

tgacattaatttgtagtttaattcatgaattaatgac 1

tgacattataaagccggagtcgccggcaattaatgac 1

tgacattattgaccaaataaataaaaactaccttgac 1

tgacattccattccattcatgaatgcggttagttgac 1

tgacattccttatgactacgaggatgaagatgatgac 1

tgacattgcataaacagagagcgtcatgccgattgac 1

tgacattggaggtgatacatcatcagagggaggtgac 1

tgacattgttcactcgatcacggatttgatatctgac 1

tgacattgttggtccacatacagtacccacttatgac 1

tgacattgtttctccacaatttttgggtttgagtgac 1

tgacatttacatttgaacaaagaaataaacacatgac 1

tgacatttgactttttaagtgacaccaagattctgac 1

tgacatttgtacagtttttgtctgagtctataatgac 1

tgacatttgtccacgtttaataagagtgaccagtgac 1

tgacatttgtggaaataatggtggatgcgatgatgac 1

tgacattttaaagatattttaaagcgatgattttgac 1

tgacattttcgataacgtggtgaggtcactgtgtgac 1

tgacattttgttttggatctgacattgctaatttgac 1

tgaccaaaaacgaagtatcatgtccttcttaattgac 1

tgaccaaaataaaatgaaatcataacagccgtttgac 1

tgaccaaaattaacaccacaattaaactcaaagtgac 1

tgaccaaaattatctttgatttcacattaggcatgac 1

tgaccaaacatctcttgcgccttctcagtgttgtgac 1

tgaccaaataacatctataggcttctgcggatatgac 1

tgaccaaatcagaccgagaagtagtaattctggtgac 1

tgaccaaattaaaaaggataaataaatattgtttgac 1

tgaccaaccatcttcattgatccctcccgatcctgac 1

tgaccaacgcataaatgaaagtgtatataaagatgac 1

tgaccaacgtatgggcacgtctctatgtcccgatgac 1

tgaccaactttcatttatatgatttgattcaactgac 1

tgaccaataacagaataatttatcgtttaattttgac 1

tgaccaatatatcacactcagaatcaacatttttgac 1

tgaccaatcaaacgatcatactcgagccacttgtgac 1

tgaccaatcttttggaaaactaacactaatcgatgac 1

tgaccaattcatatacaaaagcatctcaagaaatgac 1

tgaccacccattttgtgtctttattttatacagtgac 1

tgaccacctctttaactgcgaccatgacagcgatgac 1

tgaccacttttaaatccgtcaataaatgttacatgac 1

tgaccagaataaacaatggatactattccaaactgac 1

tgaccagaatttttgttgattctttaaaatttctgac 1

tgaccagataatataaaatgatgaaatctagcttgac 1

tgaccagatctccataagaaacacgttaccatttgac 1

tgaccagatttcttcctaaatataagctttaaatgac 1

tgaccagcttatccatgtagtactgaaagtatgtgac 1

tgaccagtactcagcaaggcttcttaagaagcctgac 1

tgaccataaaaatggagcatatgtttctacagctgac 1

tgaccataatattggcatgagacatgagtcttttgac 2

tgaccatacacttcagaaaatcaaaatgcaatttgac 1

tgaccatcatagcatttcagttgacttggagagtgac 1

tgaccatgtaatatttttcaattgtcctatctatgac 1

tgaccattaacataattttggttcgaacgaaagtgac 1

tgaccattaattctaaccactttttttggtccttgac 1

tgaccattgtctattgacggatattccaaagtttgac 1

tgaccatttaaccaaatttaaaagttgaccatttgac 1

tgaccattttattttatttgcaaaaacaattgatgac 1

tgacccaaagaaaatgatatttaatagaccaactgac 1

tgacccaaatataagccttactgccttagagcttgac 1

tgacccaagcaaggcactcgacactaattggtatgac 1

tgacccacaaaactgtaagaaacatctaaatgatgac 1

tgacccacctgacccacgctctctctcacacgctgac 1

tgacccagcatattaacgttcgcaaatgattggtgac 1

tgacccagtcagttgctccaggattgcaagtactgac 1

tgacccattgctttggctattttcacacccatgtgac 1

tgaccccaaactatatatgaaaatgactctttttgac 1

tgaccccattctaaagctttttatgagctttcatgac 1

tgacccgaaacattttattttatatcgttgaaatgac 1

tgaccctatttgcaatagtaaaatatctataaatgac 1

tgaccctcgggtgcacgaggcagttccacttcctgac 1

tgacccttacttgaggcaaaggctgcagcttcgtgac 1

tgaccgacagacgacaggatatgaaatcttatttgac 1

tgaccggaagacaagaacaatgcttcctcctcttgac 1

tgaccggctccaacaaaactttgactctgactctgac 1

tgaccgtaattttttcgtaagaatatcgatttctgac 1

tgaccgtagaggcaaattatgtactatccacgttgac 1

tgaccgtccactaccatccataagtatgtataatgac 1

tgacctaaaacattttttgtatatataattggttgac 1

tgacctaaggggaatctcgtgagattcgttttttgac 1

tgacctaataatctctgggatttctgttgattttgac 1

tgacctataacaatgaccgtttcgttacgccgttgac 1

tgacctcagtttcattttgacatgtcttgttcttgac 1

tgacctgaactatttttgccccttttggctacttgac 1

tgacctgacctgctctgtctatatgtgatattgtgac 1

tgaccttgaacagtgtcaactagttggggaagctgac 1

tgaccttggtgtcgtaaacttgaccgaacactttgac 1

tgaccttgttcgtcttgactcttgaatttttactgac 1

tgacctttaaaactataaatatatatatatatatgac 1

tgacctttaacttattatatagaaaataaaagttgac 1

tgacctttactcaaattagtaaaagtttggtagtgac 1

tgacctttcccatttcaacatccaaggccaagttgac 1

tgacctttgtgatttttccccatatagacgatttgac 1

tgaccttttagtgattaccgtgggatctaccaatgac 1

tgacgaaaaaacaaaaaaacatcaatttttcattgac 1

tgacgaaaaagataaatccacggtggcgggaactgac 1

tgacgaaaacaatcctattactcaagatagaaatgac 1

tgacgaaatgttacacgtattaaaattgttaaatgac 1

tgacgaaattgaccttccctcttgcacatttaatgac 1

tgacgaagacgatgatgatctttgcggcggcgatgac 1

tgacgaatgatatcactagcagcctctttggtttgac 1

tgacgacaaaaaatagtacaaataaaatttacatgac 2

tgacgacgaagacgagatttttttttttttttttgac 1

tgacgacgaaggttggtggtttgggaacaaatttgac 1

tgacgacgactcgtggggtcgtgtacacattggtgac 1

tgacgacgatacggtttccatcaacaaaaactctgac 1

tgacgacgtcgtttgcttccttgagaagctcgctgac 1

tgacgacgtctagttacaggaagatcgcctaaatgac 1

tgacgactgacgagagactgagtttaagagttgtgac 1

tgacgatatattgaacaattttagtacaccaattgac 1

tgacgatcatgagtaacgcataatacattcagatgac 1

tgacgatgatggctctgctcttgaactcgacgatgac 1

tgacgatgcattttcaaagttatatgaacaagttgac 1

tgacgattcacaaaactctgtaatcttgctttttgac 1

tgacgattctcactgtggttcatgtacctatattgac 1

tgacgatttagacactcagaattttgagaagtttgac 1

tgacgatttgtttctgttactttcactttcatatgac 1

tgacgcaaatatttaagtgtattccgtagaatgtgac 1

tgacgcaagttatccacgtgtcttatttaaatttgac 1

tgacgcacaacagagatcagtttcttatttatatgac 1

tgacgcataaaattcaactataatctacaaaaatgac 1

tgacgcatagtaacctttctcatattccatttctgac 1

tgacgcccggatgtcatagtctaaatgactgtgtgac 1

tgacgctaagcataaatagttcgtctgtttcattgac 1

tgacggacgaagcggacggtgtgaagtttaagctgac 1

tgacggagctagagcattaggaggggggtcacttgac 1

tgacggatatggagtggttcttcttggtttccatgac 1

tgacggatgaagcagacggtgttagatttaagctgac 1

tgacggcccatggttttttaaacgtcgtcgtcttgac 1

tgacggcgacttttattacgcttagcgtaaaactgac 1

tgacgggaatcttggcttctggtttgtttgggttgac 1

tgacgggcgatgtctcaagggaaatcttgaatgtgac 1

tgacggtaaaatctacgtggtgactgactcgagtgac 1

tgacggtggaagagcattaacgacatttggtgatgac 1

tgacggttgttcccgtcaagctcaaccctgccgtgac 1

tgacggtttgattatttgatatgtttcaatttatgac 1

tgacgtaaataagtcaattggtaattaatagggtgac 1

tgacgtagatgacaaaaaaataaaaaaaatcattgac 1

tgacgtaggtttacatgtaccaaaattattaattgac 1

tgacgtatttgtccctttttgacaattctttcatgac 1

tgacgtcaaatgaccaagactatagtctatacgtgac 2

tgacgtcattagacagtgataatatggtgaaaatgac 1

tgacgtcgagtatcatataattaccaaatttcatgac 1

tgacgtctccactaaaccacacaacatagcgcgtgac 2

tgacgtgaagctttttaaattgaagaccatcggtgac 1

tgacgtgagtcatcctattaaaactcatggtggtgac 1

tgacgtggaaagcccacgtagatataatggtgttgac 1

tgacgtggagtgtcgttttctttctattcgaggtgac 1

tgacgtgtactcaccttatcttacattatttgatgac 1

tgacgtgtcgatacaccattggtgggattataatgac 1

tgacgtgtgtatgtgtatattgagactgacatatgac 1

tgacgttaacttgtgcataggcgtttaatgcggtgac 1

tgacgttttgtggaatcttgccaaaaatgtagttgac 1

tgactaaaagactaataatgtctcccatgctagtgac 1

tgactaaaatacccatgatataattttaacaaatgac 1

tgactaaaatacccatgttataattttaacaaatgac 1

tgactaaaatacccattctataattttaacaaatgac 1

tgactaaaccaagacacatttgggtccaggaagtgac 1

tgactaacataaatttccagagtagatgagtgatgac 1

tgactaagaatacaagaaaaacttcgagcatgatgac 1

tgactaagacatagctggagtttgtatgtacaatgac 1

tgactaagtggtaaatttgttgtaaaaaaattatgac 1

tgactaatgaattatatatgatataacctttgatgac 1

tgactaattgaataaacaacaagtctaagctaatgac 1

tgactaatttaatttgcattgtttaggataatttgac 1

tgactaattttcctcaaccaaagagtacgggtttgac 2

tgactacacaagcaaacgaaaatgttctgattgtgac 1

tgactacactaaatgtatattaaatgctttgtttgac 1

tgactacatgtcatcatatagttcgtatatgtgtgac 1

tgactacgttcctatatcgctctccaaagcatttgac 1

tgactacgttgttgagtctactggtgtcttcactgac 1

tgactactacacatctatttgtggtatttcatttgac 1

tgactagacaagttggattatagtttcgattagtgac 1

tgactagatctgacgcttctagtttagacgtcgtgac 1

tgactagatttctgttataattagctgaatatttgac 1

tgactaggtgaggaattggacgaactgactagctgac 1

tgactagttgctttgcgatgctatcataaaacttgac 1

tgactatacaaatatagtgacagtcatgattcatgac 1

tgactatatacaattgtgaaggcttgagggaattgac 1

tgactatatttaaagtgtgattagagtctcctatgac 1

tgactatcaagctgccatccatctctggaaagttgac 1

tgactatctcacggtttcaggcggcggcatccttgac 1

tgactatcttaagaaagaaatattaattcactttgac 1

tgactatcttataaaaactaaaaaaaattctcgtgac 1

tgactatcttcgattttcagtcgactgctcacgtgac 1

tgactatggttggtcaatgcattcatgctttgatgac 1

tgactattaaaagtcatatttgctcttgaccactgac 1

tgactattagaggtgttctcgtcctttttttggtgac 1

tgactatttctcaaaataagttggaaacttatttgac 1

tgactatttgttgccatgggcgaatgaagcatttgac 1

tgactcaaaggggtttgagctaaaacgacctaatgac 1

tgactcaaatttgaatcgaatatattcataatctgac 1

tgactcagacaattaacacttgtttgtacattttgac 1

tgactcagcatgccatgtcatcgttgtataagctgac 1

tgactcaggggccaaatatagacacaatttaattgac 1

tgactcatattttagtgttatgcattcattacatgac 1

tgactcatcataacttattaacttgtgcatatctgac 1

tgactcatcctatggtcagggccttgctgtctctgac 1

tgactcattggccgacgtcggaacttaaaaaattgac 1

tgactccactctgtgaccaccgaacaagaacgatgac 1

tgactccagaagccctgaggcatcgcaggacattgac 1

tgactcgattcccggtttgtagccaaccaaagatgac 1

tgactcgcccatactcatgacacgtggacatgctgac 1

tgactcgctcgtgaaaggttattcgactagccgtgac 1

tgactcgctctctctctctcgctggctcgcttctgac 1

tgactcggtctttctggaaaacgtgttcctgattgac 1

tgactcgttgagctaagtatctattaggcattgtgac 1

tgactctagcagctcggattctgaatctgactctgac 1

tgactctatcgtgtccaaaatcaaaacttctcttgac 1

tgactctcataagttgtttttttgcaatatttatgac 1

tgactctcattctttctgaggtctgcttcttcttgac 1

tgactctcttctcgctagacgctgtgccaactgtgac 1

tgactctgtttgcatgaagggttttggtttagctgac 1

tgactcttatttatactaatacaacgaatcaaatgac 1

tgactgaaaaagaaaaacttgtagtttggtttatgac 1

tgactgaaaaggtcaaagtctttgctatctttttgac 1

tgactgataaaacattacaacatgtgattttgatgac 1

tgactgatactcggctcccgaagagacgacccatgac 1

tgactgatatgtcagattaacaactgaaaagtatgac 1

tgactgatgacaatatatacacaagaaacaggttgac 1

tgactgcatataaaaccaagacatgtgtgagtgtgac 1

tgactggagcagtagaaaggaccctacttcacctgac 1

tgactgggattccaatgatgtgtatttaaagcgtgac 1

tgactgggtaaaaagttgactttttcgtggaaatgac 1

tgactggtactaagatgtatttagacataaaactgac 1

tgactggtgacagacccacaattatctcagttttgac 1

tgactggttgctgatattttctacttttaatcttgac 1

tgactggtttttttacttgattggaattacaggtgac 1

tgactgtaaagaaataagagaaagaaagagatttgac 1

tgactgtcattacaacataattccaaatgtgactgac 1

tgactgtggatgacgaatgtagtagaactgtgatgac 1

tgactgtttaagtgagaatgaaatcggttagactgac 1

tgactgtttccatgaaacaagcttcgtggatattgac 1

tgacttaactttatgttttctcaggctggagtttgac 1

tgacttacaagaatcagaataacaagaacagattgac 1

tgacttacattatcccttcttaacaacctcaagtgac 1

tgacttacattatctcttcttaacaacttcaagtgac 1

tgacttactcgtggaagatcattcgactagccatgac 1

tgacttacttggagtgatttggatagaaaatggtgac 1

tgacttacttgttgttgaagggaaggctgggggtgac 1

tgacttataacaccctcattgatgggttgtccatgac 1

tgacttatggtattcgctttggcattttcagtttgac 1

tgacttattcttctaaaaatgtaacaatttcgctgac 1

tgacttcaaaattgtattggttctcactacagttgac 1

tgacttcaccacatccacttgacactaataaattgac 1

tgacttcagtcacttaatgatcctgagtccttttgac 1

tgacttcatggaggttgataatgttgtttaggatgac 1

tgacttcgagagtagcgagagagagagacttattgac 1

tgacttcgttcccgacagcaatgaacgtgatgttgac 1

tgacttcgttggcgacaacaggtcgagcatttttgac 1

tgacttctaattgtaaccataggaaaaattagatgac 1

tgacttctttttttttttttccacaaacaacattgac 1

tgacttgagacaacgagtacacactacttgacttgac 1

tgacttgatagtgatgtgtaaaaagatgagtgctgac 1

tgacttgattctggcttcggacgtggtgtatcatgac 1

tgacttgattgctttctttttttgacataacgatgac 1

tgacttgccttgtcctttcttccttgacctaaatgac 1

tgacttgcgatcctgagttgactgatttacctttgac 1

tgacttggatgctaataaaaattgcttaagcaatgac 1

tgacttgggcattgagaatttccaaactttctctgac 1

tgacttgtaacaaaaaaaaaagaataatgtaaatgac 1

tgacttgtaaggtggttgtggtaggtggagaagtgac 1

tgacttgtgaaagttgtatcatgcactttagcatgac 1

tgacttgtgacagggagaatctgagaacctgcatgac 1

tgacttgtgatagaatataaaagtttttgttgttgac 1

tgacttgttatgaaagggtgtgagtaagagttgtgac 1

tgactttactttgtgagatcatattcatatgtatgac 1

tgactttagttaattaaaattaaataaaaaatttgac 1

tgactttcaggataatcaggctcttcatcaccttgac 1

tgactttcttaaccctaatattttgtgcaagtttgac 1

tgactttgacttgtagtcttgtaccattgaatttgac 1

tgactttgtacagcgtagtatatgatgaaatattgac 1

tgacttttaaacacgtccgtgagattttcagattgac 1

tgacttttagcttttgacattgactttgacttttgac 1

tgacttttagggatcttttgttttgtggttttgtgac 1

tgacttttatgttttgtttttacaattaattattgac 1

tgacttttcgaatctaacaaaaaaagaaaaagttgac 1

tgacttttctctctattaattttcttaatttcatgac 1

tgacttttcttgttgttcctctcatgtgacgagtgac 1

tgacttttcttttttaataattgattatctttctgac 1

tgacttttgagaaagagagacttgtcaaaaaagtgac 1

tgacttttgctgtcaaaaagaaataaagcacactgac 1

tgacttttggtagaatagtcacaaacgtcggtatgac 1

tgactttttatgggaagatgatctagaagcacttgac 1

tgactttttcagacttctttctagctgatatcctgac 1

tgactttttctctgagatcatggttacatcatctgac 2

tgactttttttttcaatattaaaacattgttgctgac 1

tgactttttttttccttggggcaaaaaacattatgac 1

tgactttttttttgccggcgatgccgacgccggtgac 1

30 : 346

tgacaaaaaaaaatatgcaatttttttcgaaatctgac 1

tgacaaaaacataaatggagtacttgttccaacttgac 1

tgacaaaaacttctacaataactttgttttgctttgac 1

tgacaaaaagttattagttcataatgctatcagatgac 1

tgacaaaaatgttttattatatttgtcaatgttatgac 1

tgacaaaacacacaataaatgggccttttgggcctgac 1

tgacaaaataatgtgtatgtttaatgttccacattgac 1

tgacaaaatacagatcgatctatatccgatctgttgac 1

tgacaaaatgatgacgcaaaaataaaagtaaaaatgac 1

tgacaaaccttttaactaaagaaaatcataaggctgac 2

tgacaaactacgagtaaagtttaatcggaagaattgac 1

tgacaaagtgctaacccaacaacaatgggacttgtgac 1

tgacaaatattaatttgataagtatttgtgtctttgac 1

tgacaaatccgtgtgttgcctttgtgtcctcccgtgac 1

tgacaaatgtaaaataatagtataaaaaaatctgtgac 1

tgacaaatttactcaatttatcatgtgtctttagtgac 1

tgacaacaaaaaaactatcagtcttagtaatatgtgac 1

tgacaacaaagatttaataataactcgcatcatttgac 1

tgacaacaacagacaaacggctccgtcaccaaattgac 1

tgacaacaaccattaagtcttcatagcttttgtatgac 1

tgacaacagaataataaatagatagaattattattgac 1

tgacaacattttcacttctgcccttaaatcaaaatgac 1

tgacaaccaaacggtccaaacttccacgtaagattgac 1

tgacaacccaaataaccaaggtaaaccgtagcggtgac 1

tgacaacgctttttttttttttttgtgtgcaaaatgac 1

tgacaactcaaaaacaaaaacaaaaaagcaaaggtgac 1

tgacaactctagcattgaagattagtagtaactctgac 1

tgacaagacactcaaattttccaggacttatgtttgac 1

tgacaagtattaccaaacttgtgtttaatttggttgac 1

tgacaagtcagttatatgatgatcataaactttttgac 1

tgacaagttcgaatggtttctaggtaaattaaaatgac 1

tgacaagttcttttgaagtaaattgcatattgtctgac 1

tgacaagttgatgcatgcaaagaagtaagaagaatgac 1

tgacaataactaatctcatgcccttgtttcactttgac 1

tgacaatacccattatatagatatctaccatgcatgac 1

tgacaatagcacatacattgtttatgccagctaatgac 1

tgacaatagtaacggttccgtaacaatttacatttgac 1

tgacaatatatattctacaacacttcaaataatttgac 1

tgacaatcaatgtgtactttatttgatataaatatgac 1

tgacaatccataaaaatatttgaatagtaattattgac 1

tgacaatctcaaacaagaaagattcaagcttaattgac 1

tgacaatctgaggataaagtttgagaaggtaatgtgac 1

tgacaatgaaccttttaactctcaacagtactcgtgac 1

tgacaatgatggatgatgatcataaagtttccaatgac 1

tgacaatgatggcacgggaatgtggatggatgcttgac 1

tgacaattattcattcaaaaaaaaatgtcaattttgac 1

tgacaatttatttatttttggtataaagaatggttgac 2

tgacaatttgattaggcattagaaaactaacctatgac 1

tgacaatttttgtcttgttgtgtaatcttgtcagtgac 1

tgacacaaccttatgataatttatatatcatatatgac 1

tgacacaagaaaattagctaaatgtcatgtttattgac 1

tgacacataaagaaagaaatctcttaatttatcttgac 1

tgacacatatgaaaagtataattttctcgctctctgac 1

tgacacatttcaatactttttgagacataagatatgac 1

tgacaccatatatgacaatacctctctttttcaatgac 1

tgacacctgcctcttagtcacgtcattgacaaaatgac 1

tgacacctggcgatagatattgatttttattgtttgac 1

tgacacctttagttgacgagatcaaaaacaaaattgac 1

tgacacgagattacgttcataatcttttgattagtgac 1

tgacacgatgagctggttgtgtcggtaaggtcaatgac 1

tgacacgattcggtgcatcgactcagctgttatttgac 1

tgacacgtaatatatataatcaaaattaacgcaatgac 1

tgacacgttgtcggttcgcatcattattttctcgtgac 1

tgacactatagcaggaggcttgaaagctgccttttgac 1

tgacactatttataaagtgtatgttccgaaattatgac 1

tgacactatttgcaatagtaaaatttgacttttctgac 1

tgacactggactcatagaactacttcctcgtccatgac 1

tgacacttttattcttttctttttttttaatcattgac 1

tgacacttttgtatatacttacattttctaattttgac 1

tgacagatggaacataacactgatattcatggtatgac 1

tgacagattgttttcggatcaatctgaaattgcttgac 1

tgacagcatcaaattcaatgtgcatcaaacgttttgac 1

tgacagcattggccgccgttgaagatgtgtcccgtgac 1

tgacagccatttggcgcatatagtgcaagatctttgac 1

tgacaggattttatttgggtattgaattgatttttgac 1

tgacaggttaaaaaattatatcgatctaatttgatgac 1

tgacaggtttgccctcgtcctttgcctataaatttgac 1

tgacagtcatggatgaatgctttctgcccattattgac 1

tgacagtctgatcatacataaaacattccctttatgac 1

tgacagtggaaacccttaaaaacctttaatctaatgac 1

tgacagtgttttgagttttgactaggggagatgatgac 1

tgacagttgcataaacccaaaagagatttgggagtgac 1

tgacataaatgcccccgaagacgaaactgtaatatgac 1

tgacataactcttaagaaagtgtgccaaaacgtgtgac 1

tgacataagtttctaaacaaataaagatatctcttgac 1

tgacataagttttagtaagatcccacaataattttgac 1

tgacataataacttgaatagctagcatcatctcttgac 1

tgacataggatgataaaaatcgattttagtgaaatgac 1

tgacatatatataattcgtacgaatgtacttcgctgac 1

tgacatattgatgtgaacaaagaaaacaaaaacttgac 1

tgacatcaaagagaggcatttcaacaaatatcattgac 1

tgacatcaccgccttactaattgacgtttccttgtgac 1

tgacatctttaattgcgtccaacaacattttttttgac 1

tgacatgacaacctcttgaagctaatatcatacgtgac 1

tgacatgactattcccactaaaactacatataagtgac 1

tgacatggtcaatttgatgtatataaactaaagatgac 1

tgacattcagaaagacaaagcatgttatcccatatgac 1

tgacattcttcaagctctcgaagccatctgaaaatgac 1

tgacattgactttgacttttgacactcgttttcgtgac 1

tgacattggccgctaatcctttgattggcggcggtgac 1

tgacattgttcattgacggaaaacccaatgagggtgac 1

tgacattgttcccgttacacattacataagaaattgac 1

tgacatttcccacggtcctatgtgttcgggattttgac 1

tgacatttccggcagagagtagtttcactgaacatgac 1

tgacatttcgttttgtaaccagttagattacaattgac 1

tgacatttgtcaacaagttaattacttttctttatgac 1

tgacattttatttttagaagctatacttacacagtgac 1

tgacattttgcatttactttctttataatttggttgac 1

tgacatttttgtatatcttgttttctccgccaagtgac 1

tgaccaaaaaacacggcatatgagagttgacgtgtgac 1

tgaccaaaaaataaatgtgatttggcgatataagtgac 1

tgaccaaaaaatgtcattatgcttattaagttgttgac 1

tgaccaaaaatgaagtatcatgtcctttcttgattgac 1

tgaccaaaacatttagcatagtaaattgtgttagtgac 1

tgaccaaaatgtcttgaattttctggtgtgattatgac 1

tgaccaaaattgaatcaatggttaagatctcttgtgac 1

tgaccaaaattttcatgtcaacccatttaaaatctgac 1

tgaccaacatcatcatccacggtgttaacatccatgac 1

tgaccaatcagtcaacccaaccatcctctcccgttgac 1

tgaccaatccaaatgtctgagagattctcccttttgac 1

tgaccacattttggctaacatggcgtgcaaacgatgac 1

tgaccacccttggtttgagttataaccaactcaatgac 1

tgaccacgcgctctctctcacagccccacttgtttgac 1

tgaccactagctttaaacctaacgtcttcatcaatgac 1

tgaccactgctcttctcagtaaattcaattccattgac 1

tgaccagacgctcttcaatatccgttagtagttgtgac 1

tgaccagaggttctttttgattaatttcaaattttgac 1

tgaccagttgactcattttttggtatttcttgtttgac 1

tgaccataatccagattttaatttatctttgacttgac 1

tgaccatacgccatttctcacatccaaaacacaatgac 1

tgaccatgtaaaaaaagaaaattgatgattggggtgac 1

tgaccattacacaatacttaaatactattcgcaatgac 1

tgaccatttagattagtaatcgcgctcaaatatttgac 1

tgaccattttcgtttctaacgatgtatgttgtaatgac 1

tgacccaaagaagaagaataaattattgaactattgac 1

tgacccaaagcaattgagttgtttcttttataaatgac 1

tgacccaactcattttgttaaatgagttatgggttgac 1

tgacccaatcgaatgtaggctaagagatatattttgac 1

tgacccagtcaaataactatttcatctaattttttgac 1

tgacccagtgttttagtaaacttttacacacaaatgac 1

tgacccatttagtttgtagagttaatgtaaatgttgac 1

tgaccccagtgagtcattgttctggctccgccactgac 1

tgaccccataatcaaaattttgttttagggtttatgac 1

tgacccctgacttttaaaaaaattgtaatcgatgtgac 1

tgaccccttctaaacaaaccgactcttgtgttcttgac 1

tgacccgagcgattgacaaagagatgaagctaggtgac 1

tgacccggcctaccaaaaccgccaaatccattattgac 1

tgaccctattttttctcgttgcctttttttggtatgac 1

tgacccttatcttttatttttatggtaagaaaattgac 1

tgacccttattgtttcttcacttttgacgaaacatgac 1

tgaccctttcaattaatttattttatgcacaaattgac 1

tgaccgatgtctttttttttttttttttcttttttgac 1

tgaccggctggcgagatacttcggacgggtgagctgac 1

tgaccgtacataaacatgtggttggagtcagtactgac 1

tgaccgtcaattattccatcgacggtctataacgtgac 1

tgaccgtctttcaaacctttgtaccatttgactttgac 1

tgaccgtttggggcaaaatcgagaaaatgatatttgac 1

tgacctaaccatggttaactctggtatatatgaatgac 1

tgacctccactgaaaagctaactcaagtttctattgac 1

tgacctctacgagcagcaagcaatgcagcttcattgac 1

tgacctctactgtatatttgaatctcaatgctcttgac 1

tgacctcttttcgaagcttccgtgcagaaatatttgac 1

tgacctgcttggtcggaccagtgaaaactcgacctgac 1

tgacctgtcttttgccaaactgatgtggaaattgtgac 1

tgaccttttataaaaagaaaaatagatttcacgttgac 1

tgaccttttctctctccgttaattccattgcggctgac 1

tgacctttttgtcgtttttttaatccttttttaatgac 1

tgacgaaagtgtcactagtagccctctcttattttgac 1

tgacgaagatgttgaactttaaaaactccataactgac 1

tgacgaatctcaacaagcttttcagaggcagccatgac 1

tgacgaattaaagatatcataggtcggcataaaatgac 1

tgacgaatttcatggttttaaaaaatttaggttctgac 1

tgacgacagatagacatctaggagctgctaaaattgac 1

tgacgacctactttctttaattaattttctgtcgtgac 1

tgacgacgaaatcctataattgtcacagttgatttgac 1

tgacgacgactttgtctttccgattccgtgaccgtgac 1

tgacgacgacttttaagcgctcttccgacgacgatgac 1

tgacgactttactttgtttaattagtctgtcacttgac 1

tgacgagacgggcgaactaagagctacttgagattgac 1

tgacgaggcaaagaatgttaggtaggtttgttattgac 1

tgacgagtgaattgtctttttacaccgtcagatttgac 1

tgacgatcataacatcaaatgtggtcgaaccgagtgac 1

tgacgatgctagttcagttcttttggcattcagctgac 1

tgacgatgtggcgtgaagagatccacagagaaactgac 1

tgacgatttagatacaataatattaggttcaaaatgac 1

tgacgcacctacgaaaaaggtactacgttgtttgtgac 1

tgacgccttttgatgacatggagaagtatgcagatgac 1

tgacgcgtaatatatccattattcattaacaatttgac 1

tgacgcgtggcaaccggagatgtcgcataagcggtgac 1

tgacgcttttgcccctagcaaagtgatgagacattgac 1

tgacggaaggagaaagaggaggaggagagagccttgac 1

tgacggagactaaggcaaaatctatcgaaagtggtgac 1

tgacggatcaaatggaaacgttacaatggttgattgac 1

tgacggatttcaaccttaagagtgaatctgaaactgac 1

tgacggcaagaacacgacaacggagaagtcgtgttgac 1

tgacggcggaggagacggcgggcggggattctggtgac 1

tgacggcgttagatttttgtggaatctaagtttgtgac 1

tgacggctagagaaagacatgttgaattaactagtgac 1

tgacggtatccaaatgaaggaacggctttggtagtgac 1

tgacggtcacgtaacaggaaaaatagatgtaacttgac 1

tgacggtgaaggctccatcaacactcactccccatgac 1

tgacggtgtgataaacgaaaaatcgtgaaagacatgac 1

tgacggttaatgtttacccaggacgtgatcacgctgac 1

tgacgtaaacagcaccatcacccatagcttcctgtgac 1

tgacgtaagaggctacgtatgttcgcttaaaatttgac 1

tgacgtaatggtttaatggtttaattggtctgtttgac 1

tgacgtatggtgctattgtgcgtcaattgcttactgac 1

tgacgtcataagaaattagggaaggaatcagttctgac 1

tgacgtcattttctaattccattttgttggtttttgac 1

tgacgtcgaggtttttcgtatcgtgtgtttcatttgac 1

tgacgtcgatgcgtcaatgtgtctgtatgtgatctgac 1

tgacgtctaaccgtttcttaaactacacgcagtgtgac 1

tgacgtggctttacaatactttgctaataaacattgac 1

tgacgtgggcttcgaagctctcagccgtaacacgtgac 1

tgacgtgtaataggtaagtcgcacgttttcgtattgac 1

tgacgtgtaatgactttttcgttttgatctgttttgac 1

tgacgtgtaatttttccattggtaaaacatgttttgac 1

tgacgtgttgcgattctaagaatctaatggaaaatgac 1

tgacgttaacgttaggagtaactgtcacatcacatgac 1

tgacgttataccaattcagtggataaataacccatgac 1

tgacgttgaaaccacctatatatatatgcatagttgac 1

tgacgtttacctcggagatgtggcatcctaatggtgac 1

tgacgtttgccatgcatagatttttcaatttttttgac 1

tgactaaaaccaagagtctcaatttttaattttttgac 1

tgactaaaagtggaagtttctttagtaaagtgtttgac 1

tgactaaaggattctttctatactgctcccaagatgac 1

tgactaaattagcagagagatttggtgccaagaatgac 1

tgactaacagcttgattgacatgtatgcgaaatgtgac 1

tgactaacccatattttatttgctaatgaaaaattgac 1

tgactaactaatttatcggcggaagattttccgttgac 1

tgactaaggtataatgttgtttaatcaaatcttttgac 1

tgactaataaagccagaaacatttgctcacatcatgac 1

tgactaatggctacacatcatttccttcttgatttgac 1

tgactaatgtcaaaaaaaaatgaaataaaaatcttgac 1

tgactaattaaggcgacatacttattttgaaatttgac 1

tgactaatttgttcaggagttccaggtcagactctgac 1

tgactacaccagagatcaatcttagatggtaccttgac 1

tgactacatatgcttacatgtacatatatatgcgtgac 1

tgactacatgtttctcaactatcaactaatgtattgac 1

tgactacattttgtaacatcaatattattgtatatgac 1

tgactaccccaactatgaatatgcaataaatcagtgac 1

tgactaccttactggttaagggagtcaacaaggttgac 1

tgactacgcaaaatagttaatggaaaaggcagagtgac 1

tgactagaataacgtaaatccatcaccttgagattgac 1

tgactagagagagtcatgattagtttaacgataatgac 1

tgactagcttttcacttggtattaaactgtagaatgac 1

tgactaggccacaccacaacgaatctttcactcttgac 1

tgactagtgactaaaatgaattatttgaaaacgttgac 1

tgactagtgtcatcttttaacaaagttgatttgttgac 1

tgactataaacgtgaggaaagtattatgcgagattgac 1

tgactataattaattttcccaaatcgaaactaattgac 1

tgactatagaaaacaaactataataagcttaaggtgac 1

tgactattcttagccaagcttcagctcagtggaatgac 1

tgactatttcctctgccgttgatcccttctcagatgac 1

tgactatttgctgcataaacaaaaaggtatgtcatgac 1

tgactattttttccccatattgagagcccaattctgac 1

tgactatttttttctttatttttggttttaaaagtgac 1

tgactcaaagtgagtcccactctaactatcatcttgac 1

tgactcaaatataagaccgtgtcggtaggtgatttgac 1

tgactcacacggagaaagtgattgtgattggatctgac 1

tgactcatcaaaccaattgacccaataactcatttgac 1

tgactcatcaaaccaattgactcatcaactcatttgac 1

tgactcatcaactcatttgacccatcaactcatttgac 1

tgactcatgagagtatatgaatttatatctaccttgac 1

tgactcattcttcaccaaataaaaataaaaatagtgac 1

tgactcattttttggtatttcttgtttgaccagttgac 1

tgactccaaatgcacggtggtgatctctgatctgtgac 1

tgactcccactttttcagttttcaatgtgtattttgac 1

tgactcctgacgagactgaagctaaacataactttgac 1

tgactccttcactagaattaacatccattaaaagtgac 1

tgactcgacgttagtgttctttttttgttttcgttgac 1

tgactcgatcctctctcttctttttcgtccgttatgac 1

tgactcggagcattgttctactcttcaattttcatgac 1

tgactcgtcaattagtttaatattcacttaatcttgac 1

tgactctaatatatattcgtttttgttctcagtttgac 1

tgactctgctatttgttactttgacgtgattgcatgac 1

tgactctggtcgtatcataaactaaaagttgttttgac 1

tgactctgtaaccatgcgcagaccccaaaatatatgac 1

tgactctgtctaaagccaaaatgggtttattatatgac 1

tgactcttaatttgcttaacattacactatgctttgac 1

tgactcttcttcatttggcaattgttggcgactatgac 1

tgactcttcttctcaccataccaactggtctctctgac 2

tgactcttcttgaggctcaattatttgggcgtgttgac 1

tgactcttgacaaaccaacgttaagctctaaagttgac 1

tgactctttggggtctctaggtcaatgtagcatgtgac 1

tgactctttttttcttttgaaccaataacctgcatgac 1

tgactgaaatgaaatatttgagagggttgactactgac 1

tgactgaacacagcaagttacaagagatgctaagtgac 1

tgactgaagttttaaacacgtctcttgtatataatgac 1

tgactgacattctatttgattttatcctacggcctgac 1

tgactgacttataacaaatttttaaatattgatttgac 1

tgactgagaacacacattgtgttgtgatgactgatgac 1

tgactgagattctattgtattttggcatatggactgac 1

tgactgaggtgatataatgtaaagtagagagtgttgac 1

tgactgattcacaagttggttgtaactaatattttgac 1

tgactgccctcgttttgttgagagtgattgttcatgac 1

tgactggtaagcacttgagactcttttttgtttgtgac 1

tgactggtttgcgcgtttgaactgatgtcatgactgac 1

tgactgtaactatctcaggaactgaaacgtttcttgac 1

tgactgtatgtatattcagaattatgtgattgattgac 1

tgactgttgattggtaccggtcaatcgtgtggaatgac 1

tgactgttttagagacttacattattctggactttgac 1

tgactgttttggttatgttgggagttcgagtcgttgac 1

tgacttaaattcataacattcacaatcaataacatgac 1

tgacttaattggtttgcagttgtacatacccacatgac 1

tgacttagcacgcaagaaacgaagcataattgggtgac 1

tgacttagtcgcgttttataaaagaaattattgttgac 1

tgacttatgatgacagctaaaataaagattgttgtgac 1

tgacttattaaacttattttttattttttcactttgac 1

tgacttcaaaaaaatactagaacgaattgatccatgac 1

tgacttcacgataatttcaggtttttgtgttggctgac 1

tgacttcagcacacggcacaaacgaaagaccaaatgac 1

tgacttcattaatcaaaatcaatttatctcttggtgac 1

tgacttccaaattaacaaatctttgattttgcattgac 1

tgacttccaagaaaagttcccttgcacataaatatgac 1

tgacttccagctatcttaggaagtttgattttcttgac 1

tgacttccatctttattcttgtgctatggttccatgac 1

tgacttctctaactaattatagtcagacagtcaatgac 1

tgacttctctgcttaatcgtcgcttacttacccgtgac 1

tgacttcttcaataaatattcaatttattggtattgac 1

tgacttctttaatttattttttggcaatatcttgtgac 1

tgacttgaagttaatgttgtgttaagtgtcaacatgac 1

tgacttgatatttttatacaactgagtcggtttttgac 1

tgacttgcttgtaagttgcttctctaaagtcttttgac 1

tgacttggcacaatttaagataagttttgacttttgac 1

tgacttgtgtttcctaatttcagatcgttctagatgac 1

tgactttagaaatttatttaaattcaaaatttaatgac 1

tgactttatagaaaggacactatgatgatgcaattgac 1

tgactttatgattaagcaattgagaccaactggatgac 1

tgactttatttttttaaagagaaaagctgaggcatgac 1

tgactttcatctctgtctccatcgtcttctagattgac 1

tgactttcgtattcgggtcggatccacccggatttgac 1

tgactttctacattacattatagtattgactgtttgac 1

tgactttgaatacaaattggtaaccgaattactttgac 1

tgactttgctatatcttcaaattaattcatgttttgac 1

tgactttggtcaacggacgttaagaattaagaattgac 1

tgactttgtcaagtcctaacttgccgaatttagttgac 1

tgacttttaactggcacaacccaaagtctcaaattgac 1

tgacttttcacttccatctccaattggaaacttctgac 1

tgacttttctgactgatacaaaaataaaattaaatgac 1

tgacttttcttggtctcggtaatgttttcatctttgac 1

tgacttttggatcacttccataaatgtcgtttgctgac 1

tgacttttggtatatagcattattcctttcctcttgac 1
